# Influence of the polar light cycle on seasonal dynamics of an Antarctic lake microbial community

**DOI:** 10.1186/s40168-020-00889-8

**Published:** 2020-08-09

**Authors:** Pratibha Panwar, Michelle A. Allen, Timothy J. Williams, Alyce M. Hancock, Sarah Brazendale, James Bevington, Simon Roux, David Páez-Espino, Stephen Nayfach, Maureen Berg, Frederik Schulz, I-Min A. Chen, Marcel Huntemann, Nicole Shapiro, Nikos C. Kyrpides, Tanja Woyke, Emiley A. Eloe-Fadrosh, Ricardo Cavicchioli

**Affiliations:** 1 School of Biotechnology and Biomolecular Sciences, UNSW Sydney, Sydney, New South Wales 2052 Australia; 2 Department of Energy Joint Genome Institute, Berkeley, CA USA; 3 Institute for Marine and Antarctic Studies, University of Tasmania, 20 Castray Esplanade, Battery Point, Tasmania, Australia; 4 476 Lancaster Rd, Pegarah, Australia; 5 Mammoth BioSciences, 279 East Grand Ave, South San Francisco, CA USA

**Keywords:** Antarctic microbiology, Polar light cycle, Metagenome time series, Host-virus interactions, Meromictic lake, Microbial food web, Green sulfur bacteria, Phototroph

## Abstract

**Background:**

Cold environments dominate the Earth’s biosphere and microbial activity drives ecosystem processes thereby contributing greatly to global biogeochemical cycles. Polar environments differ to all other cold environments by experiencing 24-h sunlight in summer and no sunlight in winter. The Vestfold Hills in East Antarctica contains hundreds of lakes that have evolved from a marine origin only 3000–7000 years ago. Ace Lake is a meromictic (stratified) lake from this region that has been intensively studied since the 1970s. Here, a total of 120 metagenomes representing a seasonal cycle and four summers spanning a 10-year period were analyzed to determine the effects of the polar light cycle on microbial-driven nutrient cycles.

**Results:**

The lake system is characterized by complex sulfur and hydrogen cycling, especially in the anoxic layers, with multiple mechanisms for the breakdown of biopolymers present throughout the water column. The two most abundant taxa are phototrophs (green sulfur bacteria and cyanobacteria) that are highly influenced by the seasonal availability of sunlight. The extent of the *Chlorobium* biomass thriving at the interface in summer was captured in underwater video footage. The *Chlorobium* abundance dropped from up to 83% in summer to 6% in winter and 1% in spring, before rebounding to high levels. Predicted *Chlorobium* viruses and cyanophage were also abundant, but their levels did not negatively correlate with their hosts.

**Conclusion:**

Over-wintering expeditions in Antarctica are logistically challenging, meaning insight into winter processes has been inferred from limited data. Here, we found that in contrast to chemolithoautotrophic carbon fixation potential of Southern Ocean Thaumarchaeota, this marine-derived lake evolved a reliance on photosynthesis. While viruses associated with phototrophs also have high seasonal abundance, the negative impact of viral infection on host growth appeared to be limited. The microbial community as a whole appears to have developed a capacity to generate biomass and remineralize nutrients, sufficient to sustain itself between two rounds of sunlight-driven summer-activity. In addition, this unique metagenome dataset provides considerable opportunity for future interrogation of eukaryotes and their viruses, abundant uncharacterized taxa (i.e. dark matter), and for testing hypotheses about endemic species in polar aquatic ecosystems.

Video Abstract

## Background

Life in cold environments dominates the Earth’s biosphere [1, 2]. The cold biosphere includes distinct biomes: the depths of the global ocean, alpine regions, and polar regions. Each of these cold biomes are characterized by specific abiotic factors. Polar regions are not only perennially cold but also subject to a polar light regime where summer brings 24-h sunlight, winter no sunlight, and autumn/spring occurs within a compressed time frame. In contrast, alpine regions such as parts of the Tibetan Plateau exhibit a similar range of annual temperatures to parts of Antarctica, but are exposed to a light cycle reflective of their latitude. Understanding food web structures and biogeochemical cycles in cold environments therefore requires an appreciation of the specifically relevant environmental factors [2, 3].

**Fig. 1:**
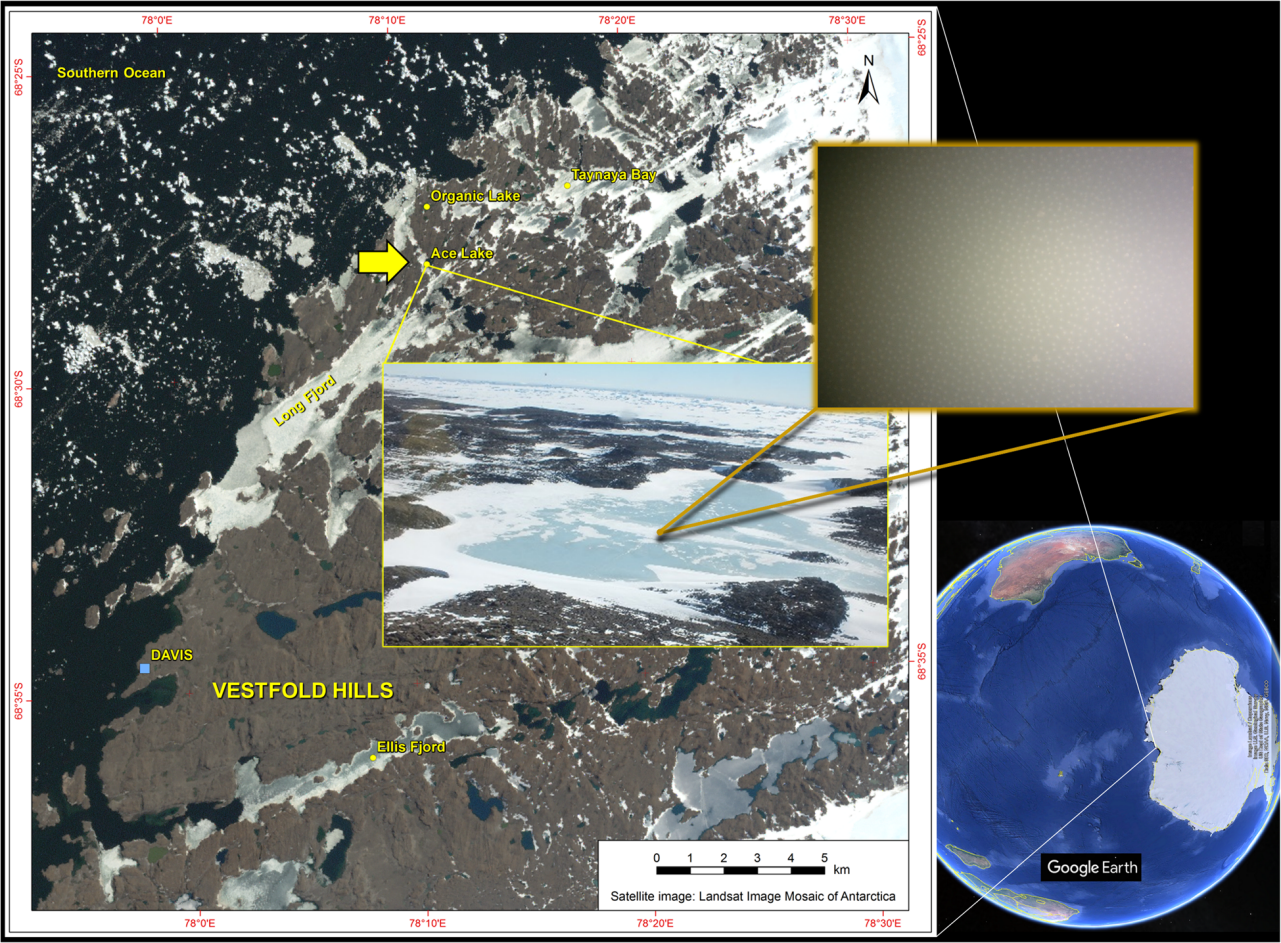
Location of Ace Lake, Antarctica, sampled for metagenomics. View of the Earth showing Antarctica with an inset satellite image of the Vestfold Hills, aerial image of Ace Lake, and underwater image of the top of the green sulfur bacteria layer at the Ace Lake interface. Credit to Google Earth (Image Landsat/Copernicus; Image US Geological Survey; US Dept. of State Geographer; Data SIO, NOAA, US Navy, NGA, GEBCO). Credit to the Landsat Image Mosaic of Antarctica—the map was produced by the Australian Antarctic Data Centre. Photo credits: Rick Cavicchioli (Ace Lake aerial photograph; underwater image of the lake interface)

In Antarctica, terrestrial [4] and aquatic [2] food webs are dominated by microorganisms. Antarctica’s aquatic environments consist of the Southern Ocean plus continental, surface, and subglacial lakes and ponds [2]. Antarctic lake ecosystems tend to support minimal or no higher trophic life forms, such as fish [2]. In Ace Lake (Vestfold Hills; Fig. 1), the calanoid copepod *Paralabidocera antarctica* is the only aquatic metazoan zooplankter known to inhabit the water column [5]. While protists can be common, the reduced level of higher trophic predators means that viruses potentially play a particularly important role in regulating cellular turnover and impacting nutrient cycles [2].

Despite recognizing that the polar light regime and other polar-specific factors are likely to influence Antarctic microbial communities, due to the challenges of working in Antarctica in winter, few meta-omic studies have been performed [2]. A western Antarctic Peninsula, metagenomics study of summer and spring communities identified temperate viruses dominating Southern Ocean waters and predicted that lysogeny enhanced the survival of hosts and viruses through winter [6]. A separate western Antarctic Peninsula study, utilizing both metagenomics and metaproteomics, identified a major shift from photosynthetic carbon fixation in summer to chemolithoautotrophic carbon fixation by Thaumarchaeota in winter [7, 8]. An analysis of pyrosequencing taxonomic data from Lake Fryxell and Lake Bonney (McMurdo Dry Valleys) also predicted the potential importance for chemolithoautotrophic processes to sustain communities during winter [9].

Ace Lake is a marine-derived, 24-m-deep, meromictic (stratified) system [5, 10] (Fig. 1). The surface is frozen for most of the year (up to 2 m thick) with the upper zone (mixolimnion) mixed by wind, and salt extrusion resulting from surface water freezing. The ‘interface’ occurs at a depth of 12–15 m and is defined by a strong halocline and oxycline where sufficient light penetrates to enable dense growth of phototrophic green sulfur bacteria (GSB). Below the interface (monimolimnion), the lower zone is stabilized by a continuing salt gradient (~ 4.2% at 24 m) that minimizes mixing and supports the growth of anaerobes, including methanogens.

The lake was sampled in the austral summer 2006/2007 for the purpose of metaproteogenomic analyses, which revealed the presence of key taxa supporting nutrient cycling and overall ecosystem function [10, 11]. Important members of the community included *Synechococcus* (a genus of cyanobacteria) performing photosynthetic carbon fixation and oxygenation of upper waters, and *Chlorobium* (a genus of GSB) at the interface performing major roles in carbon, nitrogen, and sulfur cycling. As both members of the community are phototrophic and chemolithoauotrophic microorganisms (e.g. Thaumarchaeota) were not abundant in the lake, questions are raised about how the community in the upper zone progresses through winter. Moreover, the *Chlorobium* was hypothesized to be resistant to viral infection but potentially susceptible to the introduction of invasive viruses [10], and little is known about its seasonal population dynamics.

In order to assess the stability of the community and learn how community taxa and function change seasonally, microbial biomass was resampled from the lake in summer 2008/2009, and summer 2013/2014–summer 2014/2015. The time series spans 10 years (2006–2015), and the 2013–2015 sampling includes eight time points representing a full Antarctic seasonal cycle (Additional file 1: Fig. S1). Here, we describe the nutrient cycling capacity of the most abundant taxa focusing on the dynamic of the population in the photic zone.

## Results and discussion

### Overview of metagenomic data

Biomass from Ace Lake was collected from the upper oxic zone (U1, U2, U3), the interface (I), and the lower anoxic zone (L1, L2, L3) (Additional file 1: Table S1; also see the “Methods” section). The precise depths sampled varied marginally due in part to differences in the water level in the lake at the time of sampling; the sampling depth ranges were as follows: U1, surface; U2, 5 m; U3, 11.5–13 m; I, 12.7–14.5 m; L1, 14–16 m; L2, 18–19 m; L3, 23–24 m. A total of 120 metagenomes were analysed (Additional file 1: Table S1). Abundance calculations are described in the “Methods” section. Briefly, for operational taxonomic units (OTUs), ‘relative abundance’ describes the sum of coverages of the contigs assigned to an OTU in a metagenome relative to the sum of coverages of all contigs in the metagenome; ‘peak relative abundance’ describes the highest relative abundance of an OTU within a set of metagenomes being considered (e.g. all 120 metagenomes, a season, a lake depth); and ‘total abundance’ describes the sum of the coverages (contig read depth × contig length) of the contigs of an OTU. OTUs with relative abundance ≥ 1% in at least one metagenome were considered abundant OTUs. For KEGGs, the abundance of a pathway or an enzyme in a metagenome was calculated using the sum of the read depth of contigs corresponding to predicted genes assigned KO annotations, and normalized to all 120 metagenomes. The relative abundance of viruses represented in OTU analyses were calculated as for other OTUs. The read depth of contigs corresponding to viral clusters or singletons were summed to obtain their total read depth (e.g. for a time period), with those having a total read depth > 4000 being considered abundant.

Taxonomy and taxa abundance were determined from the protein-coding genes (~ 40 million) present on ~ 25 million assembled contigs (20 Gbp); choosing contigs rather than individual proteins reduced incorrect assignments arising from horizontally transferred genes. A total of 17,157 reference-based OTUs were identified of which 117 had relative abundance greater than 1% in at least one metagenome. A total of 3.5 billion (0.5 Tbp) reads from the metagenomes were aligned against the OTU bins to refine them, and Blast was used to align the filtered OTU bins against metagenome-assembled genomes (MAGs) that were generated using MetaBAT [12] (hereafter referred to as MetaBAT MAGs), resulting in 51 high-quality OTU bins (Additional file 1: Table S2). The relative abundances of the 51 OTUs in each of the 120 metagenomes were compiled (Additional file 1: Dataset S1). A large number of low abundance contigs (< 1% relative to the sum of coverages of all contigs in a metagenome) could not be confidently taxonomically assigned, including those with best matches to uncultured bacteria, archaea, eukarya and viruses (see Additional file 2).

### Overview of seasonal and depth variation of taxa and function

**Fig. 2:**
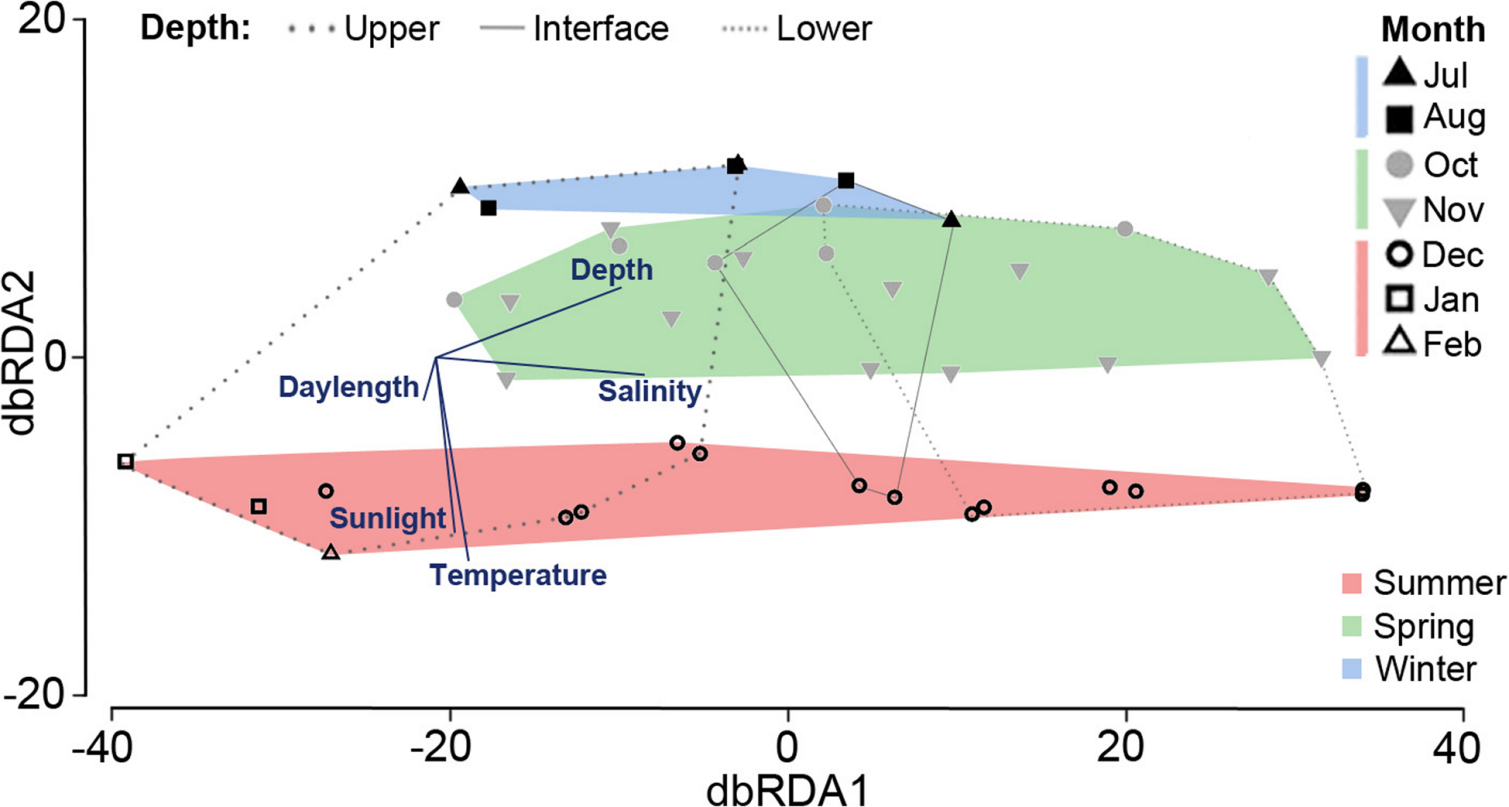
Environmental factors affecting species abundance in Ace Lake. dbRDA ordination plot showing the relationship between species abundance variations and changes in season and lake depth. The *x*-axis (dbRDA1) explains 77% of the fitted and 21% of the total variation. The *y*-axis (dbRDA2) explains 13% of the fitted and 4% of the total variation. The data points representing the three filter fractions from each depth and time point, overlap in the plot thereby reducing the 120 metagenomes to 40 data points (i.e. each data point represents the three filter fractions for a specific sampling date and depth). Included are vector overlays showing the contribution of lake depth (depth), salinity (salinity), monthly average day length (daylength), monthly average sunlight hours (sunlight), and monthly average air temperature (temperature) to variance. The season-based variations are highlighted by grouping samples from: December (white circle), January (white square), and February (white triangle) as summer (red shading); July (black triangle) and August (black square) as winter (blue shading); and October (grey circle) and November (grey triangle) as spring (green shading). The depth-based variations are highlighted by grouping samples from upper 1, 2, and 3 as Upper (thick dotted line); interface as Interface (solid line); and lower 1, 2, and 3 as Lower (thin dotted line)

The effects of a number of lake (depth and salinity) and external (daylength, sunlight hours, air temperature) parameters were used to assess the effects of environmental variables on the microbial community. The overall microbial population of the lake exhibited seasonal patterning (Fig. 2). A seasonal progression from summer through spring to winter was evident from a distance-based redundancy analysis (dbRDA) (Fig. 2, dbRDA2), in addition to separation by lake depth (Fig. 2, dbRDA1). The specific external factors, daylength (*P* = 0.001), sunlight hours (*P* = 0.002), and air temperature (*P* = 0.001) were significant explanatory variables for seasonality, with lake salinity (which increases with depth, *P* = 0.001) contributing to differences by lake depth (*P* = 0.001) (Fig. 2).

**Fig. 3:**
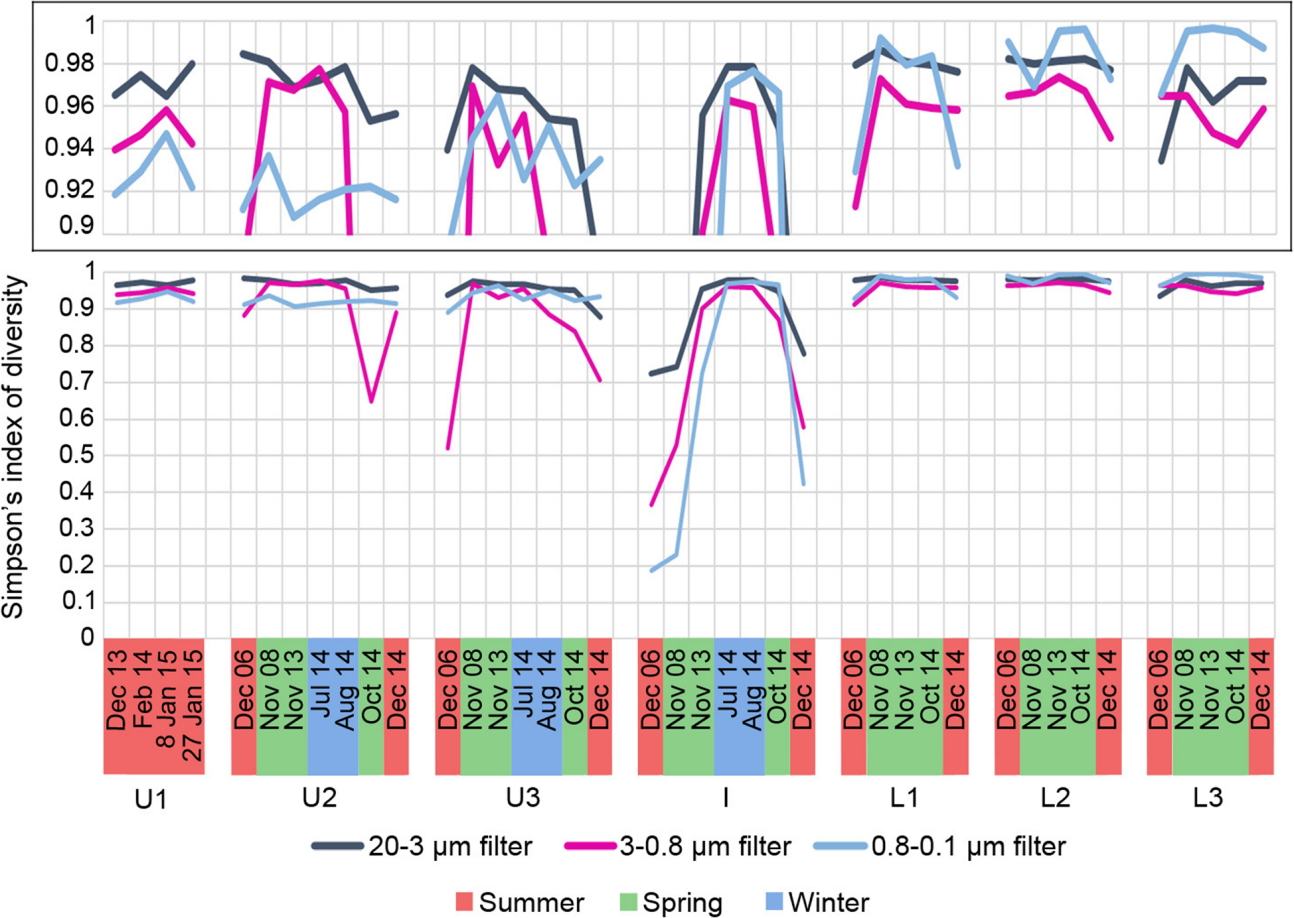
Seasonal changes in alpha diversity in Ace Lake. Line graph depicting Simpson’s index of diversity (alpha diversity) for the 120 metagenomes obtained from the three filter fractions (20–3 μm, dark blue line; 3–0.8 μm, pink line; 0.8–0.1 μm, light blue line) collected between 2006 and 2015 during summer (red shading), winter (blue shading), or spring (green shading). The data on the *x*-axis are arranged (from left to right) depth-wise from the upper to lower zone. The boxed area at the top of the figure provides an expanded view of the alpha diversity occurring between 0.9 and 1.0. Depths: U1, upper 1; U2, upper 2; U3, upper 3; I, interface; L1, lower 1; L2, lower 2; L3, lower 3 (see Additional file 1: Table S1 for specific information about sampled depths)

**Fig. 4:**
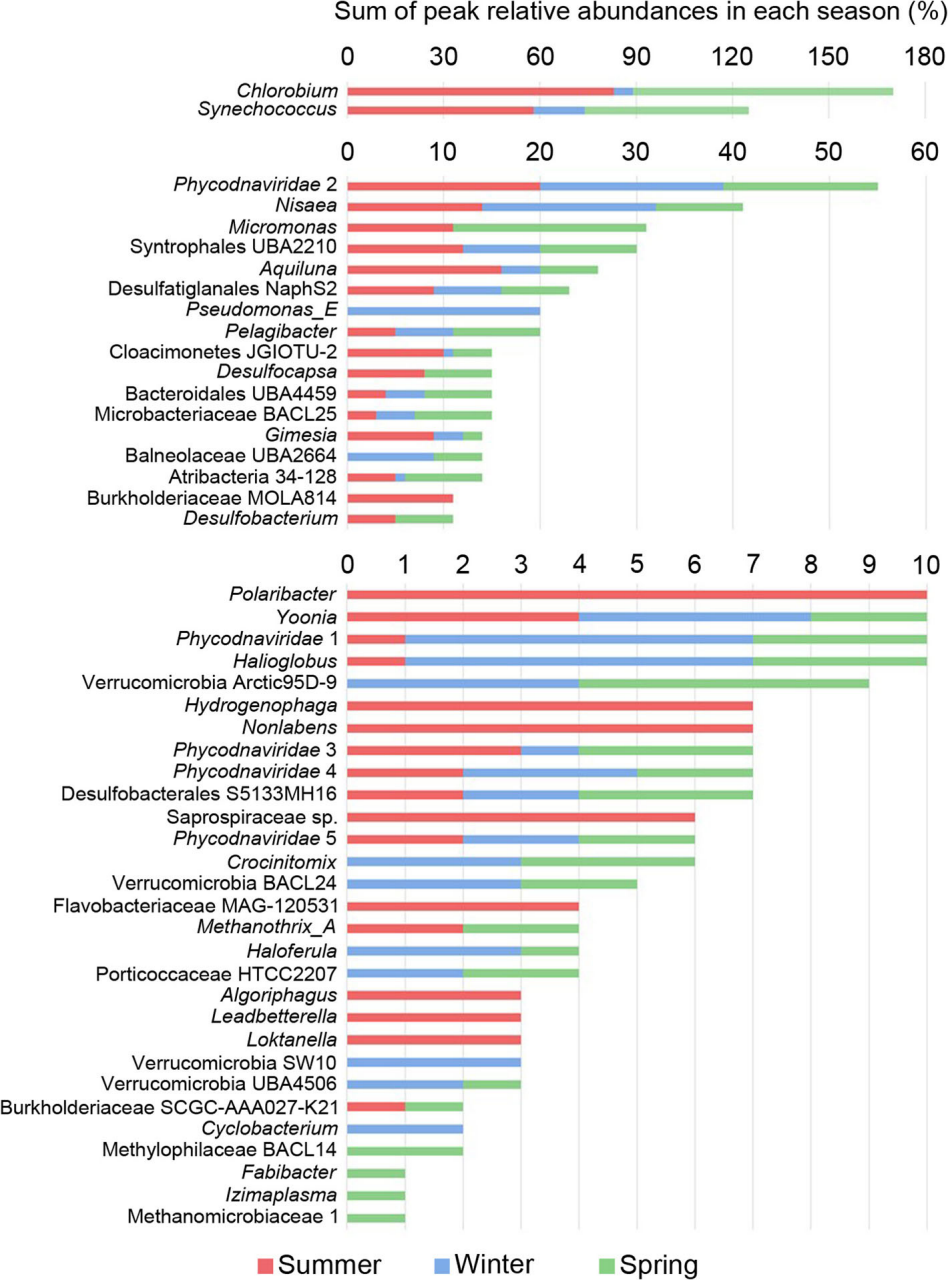
Seasonal influence on the peak relative abundance of major taxa in Ace Lake. Stacked bar graph depicting the sum of peak relative abundances from each season for abundant OTUs (those with ≥ 1% relative abundance). Note, as the peak relative abundance is shown for each season for each OTU, the bar (which represents the sum of those abundances) exceeds 100% for *Chlorobium* and *Synechococcus*. To aid the visualization of the wide range of OTU relative abundance values, the graph has been segregated into three parts with the abundance scale redrawn, and OTUs are arranged in descending order of total abundance; total abundance is the sum of the coverages (contig read depth × contig length) of the contigs of an OTU

**Fig. 5:**
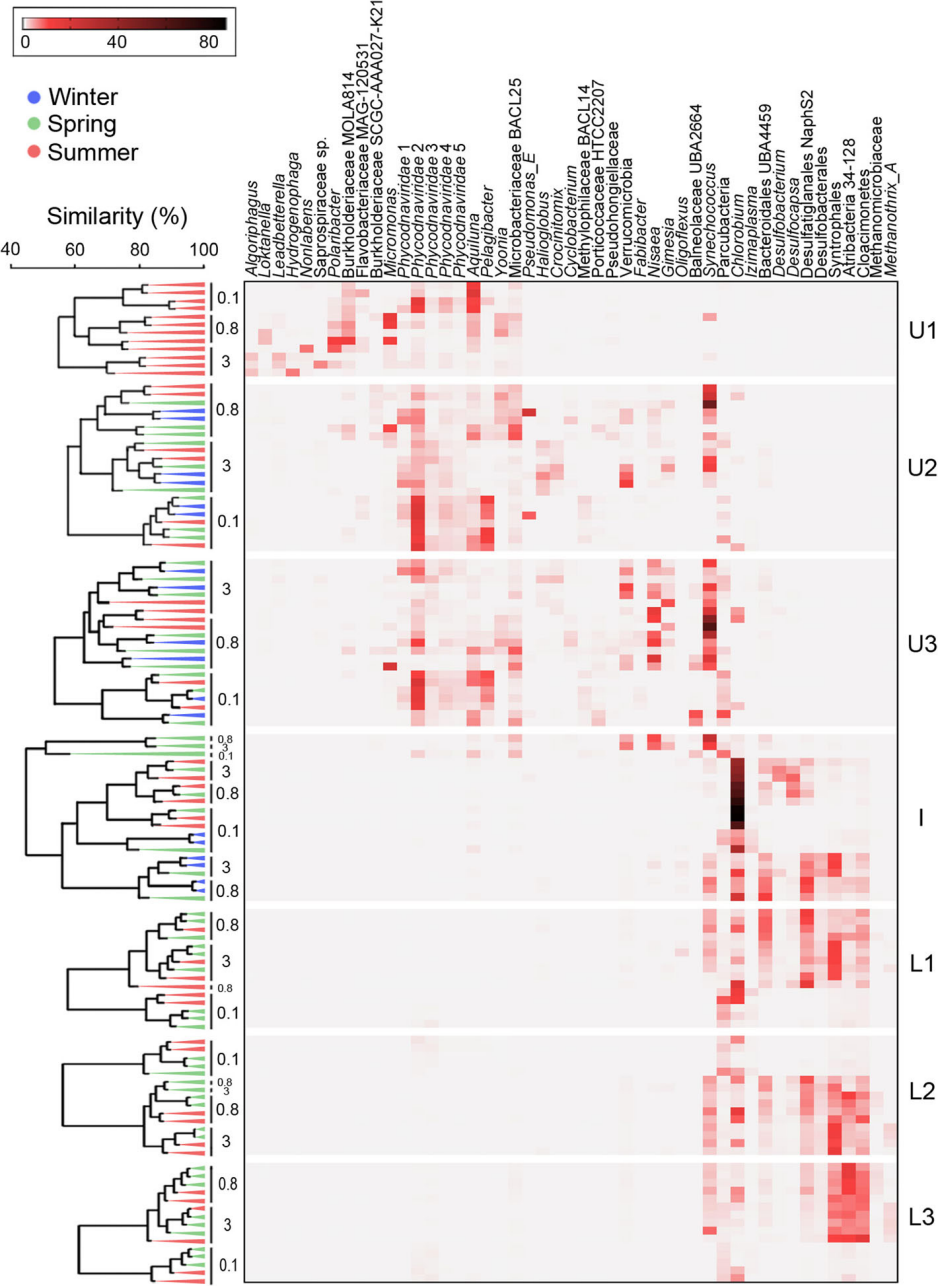
Depth distribution of the major taxa in Ace Lake. Heat map depicting the relative abundance of the most abundant OTUs by depth (right-hand *y*-axis, U1 to L3), by season (left-hand *y*-axis: summer, red; winter, blue; spring, green) and by filter size (left-hand *y*-axis: 3, 0.8, and 0.1 μm). The relative abundances of OTUs in each of the 120 metagenomes are in Additional file 1: Dataset S1. OTUs are arranged by depth from the upper to lower zones (left-to-right *x*-axis). A group-average cluster based on the Bray-Curtis percent similarities between the square root-transformed OTU relative abundances has been overlaid (left-hand *y*-axis). The cluster analysis shows the extent to which overall OTU relative abundance is influenced by size fraction, season, and depth. The gradient bar shows the colour scale in percentage used for the relative abundance of the OTUs. Filter sizes: 3, 20–3 μm; 0.8, 3–0.8 μm; 0.1, 0.8–0.1 μm; Depths: U1, upper 1; U2, upper 2; U3, upper 3; I, interface; L1, lower 1; L2, lower 2; L3, lower 3 (see Additional file 2: Table S1 for specific information about sampled depths). The upper zone had high, peak relative abundance of Alphaproteobacteria (30%), Actinobacteria (20%), Chlorophyta (20%), Verrucomicrobia (16%), and Betaproteobacteria (15%), plus a variety of dsDNA viruses (36%), whereas the abundant members of the lower zone were Deltaproteobacteria (39%), Cloacimonetes (16%), Atribacteria (15%), Firmicutes (6%), Omnitrophica (5%), Euryarchaeota (5%), Chloroflexi (4%), Tenericutes (4%), and Acetothermia (3%). At the interface, Chlorobi (84%) and Deltaproteobacteria (39%) were abundant. Some taxa, such as Bacteroidetes (U, 37%; I, 18%; L, 13%), Planctomycetes (U, 10%; I, 6%; L, 4%), Parcubacteria (U, 9%; I, 7%; L, 11%), and Gammaproteobacteria (U, 29%; I, 4%; L, 3%) were represented throughout the lake. The major taxonomic groups were represented by specific genera or species; for example, Alphaproteobacteria consisted of *Loktanella*, *Nisaea*, *Pelagibacter*, and *Yoonia* (Additional file 1: Table S8)

Alpha diversity was highest in the lower zone and lowest at the interface during summer (Fig. 3). The largest seasonal changes in alpha diversity occurred at the interface (Fig. 3) as a result of the *Chlorobium* population dominating in summer but reducing significantly in winter and spring (Figs. 4 and 5; Additional file 1: Fig. S2; Additional file 1: Dataset S1; also see below in the “The importance of sunlight”). Decreases in alpha diversity in the 3-0.8 μm fraction in U2 (spring) and U3 (summer) (Fig. 3) arose from increases in the *Synechococcus* population (Fig. 4). *Synechococcus* and *Chlorobium* were also the biggest contributors to similarity within summer samples, and dissimilarity between samples from different seasons (Additional file 1: Tables S3 and S4). The peak relative abundance (highest abundance in any metagenome) for Chlorobi (84%) and Cyanobacteria (63%), and their seasonally high contributions to peak relative abundance in summer and spring (Fig. 4; Additional file 1: Fig. S2 and Dataset S1), further illustrate the importance of these two phototrophic taxa to the ecosystem (see in the “The importance of sunlight” section).

In addition to these photoautotrophic bacteria, individual anaerobic Deltaproteobacteria showed variable abundance according to season, with certain OTUs showing decreased abundance in the winter correlated with the decline of *Chlorobium*, suggesting their growth is tightly coupled to *Chlorobium* (see the “Sulfur cycling: a tangled web” section). The Verrucomicrobia, represented by several abundant OTUs, were also more abundant in winter than summer, which may suggest that polymeric substrates preferred by this group were more available in winter (see the “Ace Lake nutrient cycles: carbon cycling” section).

The metabolic features inferred for each of the most abundant bacterial and archaeal OTUs were compiled (Additional file 1: Table S5; also see descriptions of nutrient cycles below). In general, OTUs from the upper zone were aerobic, whereas obligate anaerobes were restricted to the anoxic zone and the interface.

### The importance of sunlight

The penetration of photosynthetically active radiation (PAR) into the lake varies greatly due to seasonal effects on the level of incident radiation; the opaqueness, depth, and age of the surface ice; the depth of snow on surface ice; and the microbial density, which is particularly high at the interface [5, 13–15]. In summer, daily maximum incident radiation has been recorded as high a 1225 μE m^−2^ S^−1^ [14], with light intensity during ice-free conditions penetrating to the interface at levels as high as 100 μE m^−2^ S^−1^ [15]. However, such high light levels would not occur often at the interface [15]. Seasonal variation in light levels is very high, with daily maximum incident radiation of 1.3 μE m^−2^ S^−1^ recorded in winter [14]. This translates to PAR within the lake at a depth of 5 m being over four orders of magnitude lower in winter (0.03 μE m^−2^ S^−1^; 1.2 m of surface ice) than in summer (245 μE m^−2^ S^−1^; ice-free) [14].

The *Micromonas* (picoeukaryote) OTU was abundant in summer and spring but absent in winter, consistent with a reliance on sunlight for proliferation (Fig. 4; Additional file 1: Dataset S1). *Synechococcus* relative abundance was generally highest in spring, higher in the water column of the upper zone (U2, 51%) compared to summer where abundance was highest, deeper in the upper zone (U3, 58%) (Fig. 5; Additional file 1: Dataset S1). The depth distribution may relate to grazing pressure and the response of Ace Lake *Synechococcus* to light intensity [5, 16]. *Synechococcus* is a major primary producer in Ace Lake, performing oxygenic photosynthesis via the Calvin-Benson-Bassham (CBB) cycle. It also has the potential for photoheterotrophic and photomixotrophic growth, as reported for other *Synechococcus* strains that utilize glucose or glycerol [17–19]. Consistent with other *Synechococcus*, Ace Lake *Synechococcus* encodes transporters for the uptake and catabolism of sugars and glycerol [20], and exogenous sugars or glycerol may also be utilized for the synthesis of compatible solutes such as glucosylglycerol, sucrose, or trehalose [12–22]. Genes for the uptake and utilization of organic nitrogen sources, and for the reductive assimilation of nitrate to ammonia were present in Ace Lake *Synechococcus* (Additional file 1: Table S5), although nitrogenase was not identified, which is consistent with the inability of *Synechococcus* strains cultivated from the lake to fix nitrogen [16].

*Synechococcus* also exhibited high, peak relative abundance at the interface (25%), in the lower zone (8%), and during winter (16%) (Fig. 5; Additional file 1: Dataset S1). In mid-winter, relative abundance had dropped to 6% (July 2014) but the population increased by August of the same year (16%) (Additional file 1: Dataset S1). As the upper waters would be devoid of any light in winter (Additional file 1: Fig. S3 and Table S9), heterotrophic growth in the dark would account for such an increase. In spring (Oct. 2014), a *Synechococcus* bloom appears to have occurred (51%) (Additional file 1: Dataset S1), similar to previous observations from September 1992 [16]. The October 2014 spring bloom appears to have extended into the anoxic interface where the *Chlorobium* abundance was at its lowest (see below). It is possible that fermentative abilities allow *Synechococcus* to survive in dark and anoxic conditions, as inferred for *Synechococcus* in the Black Sea [23]. Ace Lake *Synechococcu*s encodes enzymes potentially involved in fermentation, including synthesis and mobilization of glycogen [23, 24], the latter coupled to hydrogen production using a Hox hydrogenase [25] or lactate production using D-lactate dehydrogenase [23].

*Chlorobium* at the interface appears to be markedly affected by the changes in light conditions. While peak relative abundance was as high as 83% in summer, the population decreased considerably in winter (to ~ 6%), falling further through spring (1%; Oct. 2014) before blooming to high abundance 2 months later in summer (59%; Dec. 2014) (Additional file 1: Dataset S1). The biomass associated with *Chlorobium* at the interface is considerable with cell counts exceeding 10^8^ cells mL^−1^ [10]. A video taken down through the water column into the interface illustrates the remarkable layer that forms and the intensity of the GSB within it (Additional file 3 including Movie S1). The *Chlorobium* biomass sinks as particulate matter in the lake [5, 10], explaining its abundance in the lower zone in summer (L1, 17%; L2, 14%; L3, 6%) (Fig. 5; Additional file 1: Dataset S1).

*Chlorobium* spp. have a genetic capacity for anoxygenic photoautotrophy via the reverse tricarboxylic acid (rTCA) cycle [26] and are able to efficiently harvest light in low-light surroundings using chlorosomes [27]. *Chlorobium* spp. may also contribute to organic cycling via photoassimilation of simple organic compounds (such as acetate and dicarboxylates) (Additional file 1: Table S5), which can boost photoautotrophic growth [28]. The closest match to the Ace Lake *Chlorobium* is *Chlorobium phaeovibrioides* which has 99% 16S rRNA gene identity, but only 85% average nucleotide identity (ANI). Unlike *C. phaeovibrioides*, Ace Lake *Chlorobium* is green rather than brown in colour (Additional file 3: Movie S1). As well as possessing the biosynthetic pathway for the carotenoid chlorobactene, found in green-coloured GSB, Ace Lake *Chlorobium* lacks the capacities to synthesize bacteriochlorophyll *e* or the carotenoid isorenieratene, both found in brown-coloured GSB [29, 30]. Because these enzymes are encoded in a bacteriochlorophyll *e* gene cluster that may be subject to horizontal gene transfer [31] and given the considerable depth of sequence coverage for the Ace Lake C*hlorobium* IMG MAGs, we infer these genes to be absent.

**Fig. 6:**
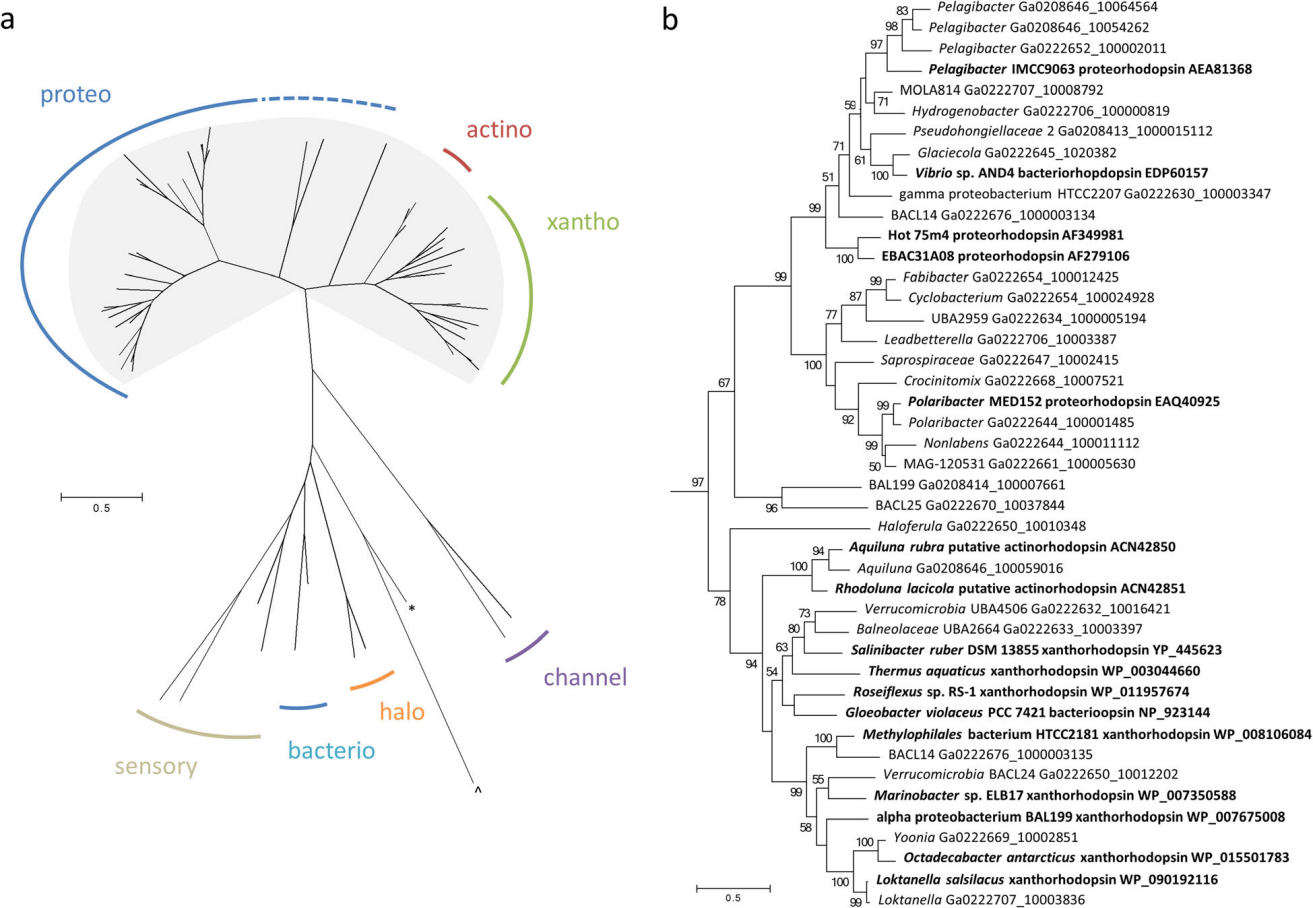
Ace Lake rhodopsins. **a** Unrooted maximum-likelihood tree of Ace Lake and reference rhodopsin sequences. All Ace lake sequences fell within the shaded area. *Nostoc sp. sensory rhodopsin Q8YSC4; ^*Nanosalina* sp. J07AB43 xenorhodopsin EGQ43296. Rhodopsin types are labelled with short-hand names (e.g. ‘proteo’ for proteorhodopsin). **b** Expanded view of the shaded region of the tree, rooted using the sensory rhodopsins. Ace lake sequences (taxon and IMG locus tag); reference sequences (bold font). Bootstrap values over 50% are reported

In addition to the dominant phototrophic bacteria in Ace Lake that utilize light to fuel carbon fixation (*Synechococcus* and *Chlorobium*), a diverse range of OTUs also harvest light to augment a heterotrophic metabolism. The potential for photoheterotrophic growth is attested by the prevalence of light-driven, proton-pumping rhodopsins (proteorhodopsins, actinorhodopsins, xanthorhodopsins) that derive from phylogenetically diverse bacteria in Ace Lake, including members of Bacteroidetes, Verrucomicrobia, Actinobacteria, Alphaproteobacteria, Gammaproteobacteria, and Betaproteobacteria (Fig. 6; Additional file 1: Table S5). Based on sequence analysis [32–34], the majority of rhodopsins are inferred to be green-absorbing, consistent with individual OTUs being found either predominantly or exclusively in the layers above the interface, with several OTUs detected only in surface waters. However, Ace Lake *Hydrogenophaga* had a blue-absorbing proteorhodopsin, and Methylophilaceae BACL14 had both green- and blue-absorbing rhodopsins, encoded by adjacent genes. Although blue-absorbing rhodopsins may indicate expression deeper in the water column [32], it can also indicate expression in ice since solar radiation is highly scattered in ice, and blue light predominates [35]. Certain Ace Lake OTUs (*Yoonia*, *Hydrogenophaga*) have genes for Type II reaction centres and bacteriochlorophyll synthesis, as well as for proton-pumping rhodopsins, but lack ribulose bisphosphate carboxylase/oxygenase (RubisCO), suggesting the capacity for bacteriochlorophyll-dependent photoheterotrophy. In these Ace Lake OTUs, it is possible that light is used for auxiliary energy production, such as under oligotrophic growth conditions [36].

### The influence of viruses

**Fig. 7:**
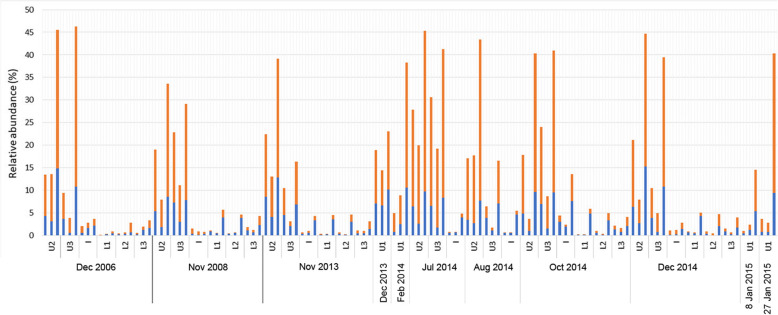
Temporal and seasonal abundance of viruses in Ace Lake. Stacked bar graph depicting the relative abundance of viruses. The data on the *x*-axis are sampling date and depth (U1, upper 1; U2, upper 2; U3, upper 3; I, interface; L1, lower 1; L2, lower 2; L3, lower 3). Each depth contains three bars which represent from left to right, 20–3, 3–0.8, and 0.8–0.1 μm filter fractions (see Additional file 1: Table S1 for specific information about sampled depths). The sampling periods represent summer (Dec. 2006, Dec. 2013, Feb. 2014, Dec. 2014, and Jan. 2015; only Upper 1 was sampled in Dec. 2013, Feb. 2014, and on 8th and 27th Jan. 2015), winter (Jul. 2014 and Aug. 2014), and spring (Nov. 2008, Nov. 2013, and Oct. 2014). The data on the *y*-axis represent the sum of the relative abundances (%) of the virus OTUs, classified as dsDNA (orange bar) or Other viruses (blue bar). The Other viruses are unclassified viruses identified in IMG plus Unassigned contigs that were confidently predicted by VirSorter as viruses (category 1 and 2) and prophages (category 4 and 5); note that the metagenome data do not represent ssDNA or RNA viruses

Viral sequences in metagenome data can derive from viruses integrated into cellular genomes (e.g. provirus), viruses associated with cells (e.g. infecting cells or remaining associated with cell surfaces during filtration), or from virions free in the water column. The size fractionation provided the capacity to infer the presence of virions if the viral signatures were in different size fractions to their hosts. This was also gauged from sequence coverage of contigs for viruses (e.g. lysogens having equivalent coverage with their host), correlation analyses, and by assembling closed viral genomes. Viral abundance varied mainly with depth, being most abundant in the upper, photic zone where peak relative abundance reached 46% (Fig. 7) (Additional file 1: Dataset S1). Consistent with this, analysis of complete, circular viral genomes identified 337 contigs representing 173 distinct viruses, most of which were phage (*Caudovirales*) that were most prevalent in the upper zone (U, 87; I, 21; L, 53) (Additional file 4: Table S1). In the upper zone, viral relative abundance in winter was similar to summer, and in fact was somewhat lower in spring indicating that a high viral presence was maintained effectively throughout the seasons (Fig. 7).

The 51 abundant Ace Lake OTUs included five algal viruses (*Phycodnaviridae* 1–5; Figs. 4 and 5), which represented 261 virus clusters and 109 singletons, many of which were taxonomically classified as nucleocytoplasmic large DNA viruses (NCLDVs). The viral clusters associated with *Phycodnaviridae* 3 differed to *Phycodnaviridae* 1, 2, 4, and 5 which shared clusters (Additional file 4: Table S2), and the relative abundance of *Phycodnaviridae* 1, 2, 4, and 5 positively correlated, but not with *Phycodnaviridae* 3 (Additional file 4: Table S3). While these associations were recognizable, co-occurrence was not observed between the most abundant green alga (*Micromonas*) and any of the *Phycodnaviridae* 1–5 (Additional file 4: Table S3), or any of the NCLDV clusters tested (7, 9, 20, 32, 35, and 66) which were chosen based on their abundance and representation in multiple metagenomes. It is important to define the seasonal contributions the algae and their viruses make to the ecosystem, and will require a dedicated effort, possibly involving single-cell and single-virus genome sequencing.

Using a cyanophage sequence assembled from the 2006 metagenome data, viral contigs from a number of cyanophage clusters and singletons were identified, all of which were exclusive to Ace Lake data as they did not cluster with any other IMG/VR sequences (Additional file 4: Table S4). Neither the Ace Lake *Synechococcus* OTU nor the *Synechococcus* IMG MAGs contain CRISPR-Cas genes, consistent with their reported absence in marine cyanobacteria [37], so viruses could not be mapped to the *Synechococcus* host through CRISPR spacer matches. In all the metagenome data, the cyanophage contigs were only assembled from the 0.1 μm fraction, and the *Synechococcus* contigs only from the 0.8 and 3 μm fractions (Fig. 5), consistent with size partitioning of the phage particles and the cells, and indicating the cyanophage sequences recovered were from virions released after active replication in the host. Despite this, no correlation was observed between the abundance of the cyanophage and *Synechococcus*, indicating the cyanophage were not primarily responsible for variations in *Synechococcus* abundance. PAR reaching *Synechococcus* is influenced not just by daylength (Additional file 1: Fig. S3), but also by cloud cover impacting sunlight reaching the lake surface (i.e. sunlight hours; Additional file 1: Fig. S4); snow cover on the lake, which in part is impacted by wind strength (Additional file 1: Fig. S5); and the presence and thickness of ice (particularly in summer when ice melts). We therefore speculate that the changeable nature of PAR availability influences *Synechococcus* growth to the extent that viral growth is sporadic and appears largely uncoupled from patterns of cellular growth.

*Chlorobium* spp. defend against viral predation using restriction enzymes and the CRISPR-Cas system [31, 38], and a subtype-2 Cas gene locus has been identified in the Ace Lake *Chlorobium* [10, 11]. *Chlorobium* spacers matched to two groups of Ace Lake contigs: one cluster (CL1024) and one singleton (SG14554). To confirm the specificity of these matches, these virus contigs were compared to the IMG/VR spacer database [39], which identified Chlorobi and Gammaproteobacteria as possible hosts (Additional file 4: Tables S5, S6 and S7). Contigs from the Ace Lake metagenomes that had high identity (≥ 98% identity to the two CL1024 contigs; ≥ 92% identity to SG14554) and a high alignment fraction (≥ 98%) were gathered to expand sequence representation. Additionally, two virus clusters (CL248 and CL400) were identified as being abundant at the interface (Additional file 4: Table S8). CRISPR spacers to CL248 were present in *Chlorobium* IMG MAGs assembled from lower zone metagenomes (as well as specific contigs for Gammaproteobacteria, including *Klebsiella pneumoniae* and *Marinobacter antarcticus*).

**Fig. 8:**
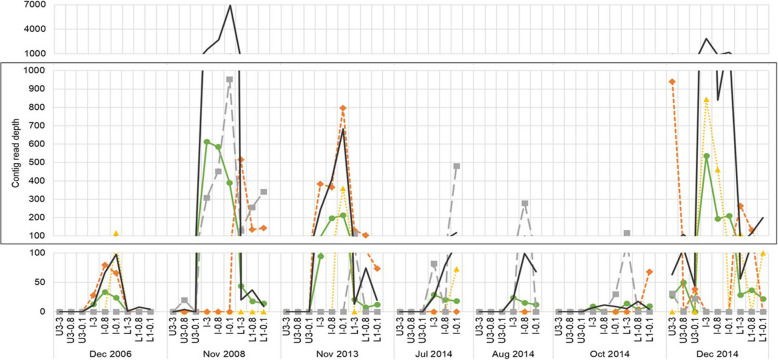
Association between the abundance of *Chlorobium* and the abundance of its potential viruses. Line graph depicting the contig read depth of *Chlorobium* (black line) and the expanded CL1024 cluster (green circle), expanded SG14554 singleton (yellow triangle), CL248 (orange diamond), and CL400 (grey square) in the metagenomes from the three filter fractions (3, 0.8, and 0.1 μm) from the interface (I) and neighbouring depths (upper 3, U3; lower 1, L1). The metagenomes represent summer (Dec. 2006 and Dec. 2014), winter (Jul. 2014 and Aug. 2014; L1 was not sampled in winter), and spring (Nov. 2008, Nov. 2013, and Oct. 2014). Filter sizes: 3, 20–3 μm; 0.8, 3–0.8 μm; 0.1, 0.8–0.1 μm

The abundance of the expanded CL1024 cluster (Pearson correlation coefficient *r* = 0.7, *P* = 2e− 11), expanded SG14554 singleton (*r* = 0.97, *P* = 0.02), and CL400 cluster (*r* = 0.9, *P* = 5e− 11) had significant positive correlations with *Chlorobium* abundance; the CL248 cluster also had a positive, but not significant, correlation (*r* = 0.5, *P* = 0.7) (Fig. 8). These positive correlations may be explained by the viruses growing cooperatively with the *Chlorobium*, but not being a cause of its immediate decline, which would be characterized by a negative correlation. Moreover, in October 2014 when *Chlorobium* abundance was at its lowest, CL1024, SG14554, and CL248 were not detected, indicating the *Chlorobium* decline is not associated with large-scale virion production by these viruses, which are thus likely not responsible for the *Chlorobium* decline. The marked reduction in the *Chlorobium* population through winter and spring is therefore likely caused by other factors, such as cellular decay due to insufficient light for maintenance energy production, and/or predation by eukaryotes.

CRISPR spacers are derived from invading DNA, and up to 60 spacers were identified in spacer arrays from *Chlorobium* IMG MAGs. Many spacers were present more than once across the 10-year sampling period. We speculate that the capacity to defend against diverse viruses and grow cooperatively with specific viruses is likely to contribute to the ability of the species to regenerate so well in summer, with the ability to reach such high cell density being the main strategy for *Chlorobium* to prevail through winter and spring (Fig. 4). The Ace Lake *Chlorobium* and the viruses it supports (e.g. CL1024, SG14554, CL400, and CL248; Additional file 4: Table S9) have only been identified in Ace Lake. Viral contigs from the three clusters, plus the singleton, are distinct from Chlorobi viral genomes recovered from Trout Bog Lake in Wisconsin (PRJNA611599, SAMN14323023 [40]) and seem to represent distinct genera based on vContact2 classification into distinct genus-level clusters (Additional file 4: Dataset S1). Adaptation to environmental conditions including polar light cycle, low temperature (Additional file 1: Fig. S3, S6 and S7) and specific nutrient conditions is likely to be a strong driver making the Ace Lake *Chlorobium* virus system endemic to the Vestfold Hills in Antarctica or possibly polar environments.

While viruses of phototrophs were the most abundant, it was notable that one complete, circular genome was for a ~ 528 kb ‘huge’ phage [41] belonging to cluster 24. Contigs constituting complete, circular genomes for the phage were present in the 0.1 μm fraction from November 2008 (L1, 0.9 % relative abundance) and October 2014 (L1, 2 %; L2, 1%) (Additional file 4: Table S1 and S10). Large CL24 contigs were also present at 1 or 2% relative abundance in 0.1 μm-fraction metagenomes from L1 or L2 depths (November 2008 and 2013, and December 2013), and from the 3 μm fraction in L3, November 2008 at 0.2% relative abundance (Additional file 4: Table S10). The size and depth partitioning of contigs for the virus likely reflect virion (0.1 μm) and intracellular (3 μm) forms, and the presence of the phage in multiple years attests to its persistence within the lower zone population. The phage has a putative type I-C CRISPR-Cas system, and CRISPR arrays with spacer sequences were also present in CL24 virus contigs, even though the spacer acquisition genes (*cas1*, *cas2*, and *cas4*) were absent. These are characteristics of some, recently reported huge phage [41]. The spacers did not match any virus contigs in the IMG/VR viral database, but were identical to spacers from some Ace Lake bacterial contigs (a Gammaproteobacteria and a Firmicutes). Since the CRISPR repeat sequences of the phage and these bacteria (presumed hosts) were identical, it is possible that this huge phage uses host *cas* genes to acquire spacers that target other viruses infecting its hosts [41].

### Ace Lake nutrient cycles: carbon cycling

**Fig. 9:**
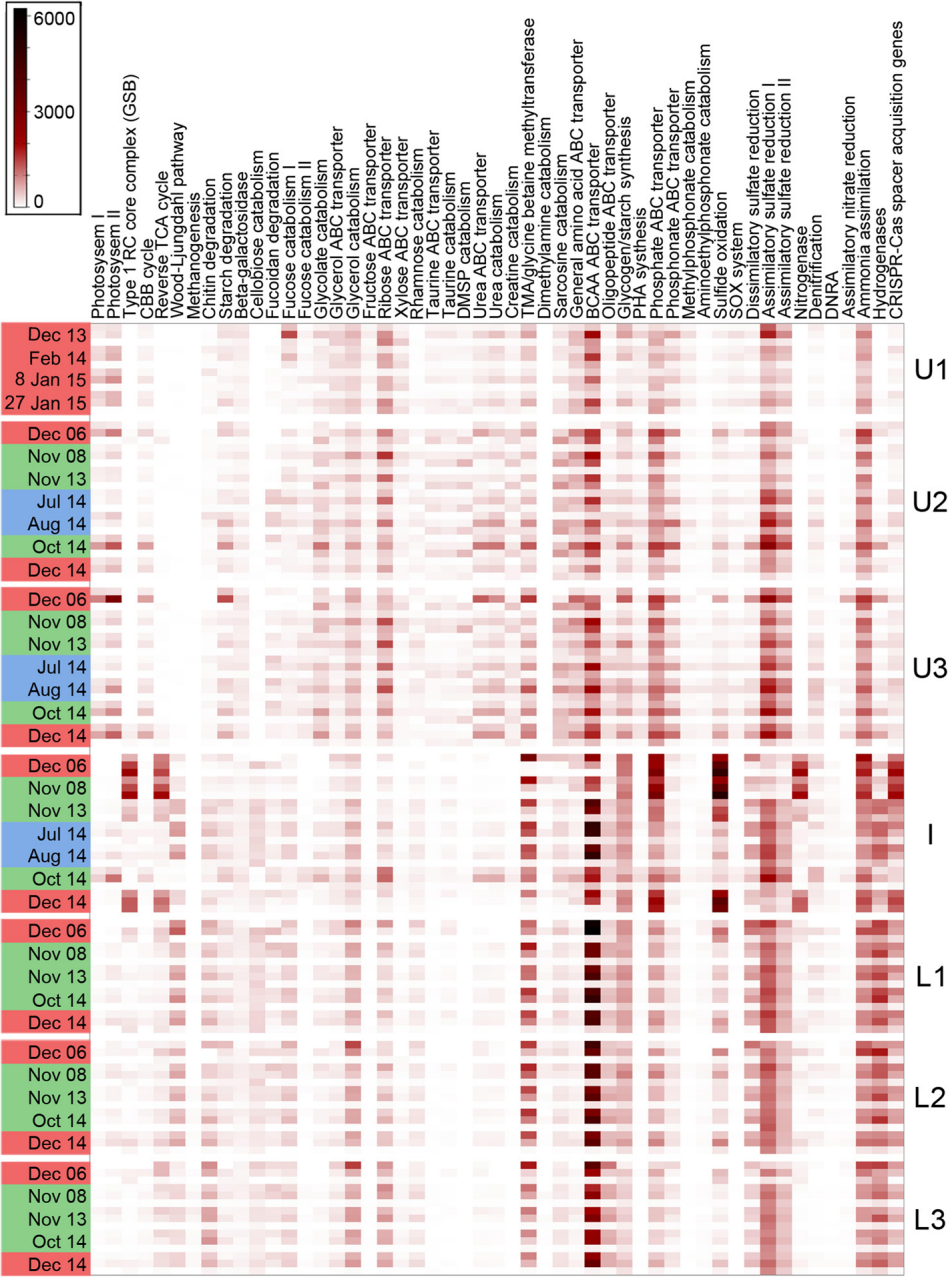
The abundance of specific enzymes or pathways involved in energy conservation and metabolism in Ace Lake. Heat map depicting the normalized abundance of specific enzymes or pathways (*x*-axis) by season (left-hand *y*-axis: summer, red; winter, blue; spring, green) and by depth (right-hand *y*-axis: U1 to L3). Each sampling date (left-hand *y*-axis) displays abundances for each of the three filter fractions: top, 20–3 μm; middle, 3–0.8 μm; bottom, 0.8–0.1 μm. The gradient bar shows the colour scale used for the normalized abundance of the enzyme or pathway. Depths: U1, upper 1; U2, upper 2; U3, upper 3; I, interface; L1, lower 1; L2, lower 2; L3, lower 3 (see Additional file 1: Table S1 for specific information about sampled depths). BCAA ABC transporter, branched-chain amino acid ATP-binding cassette transporter; Cas, CRISPR-associated; CBB cycle, Calvin-Benson-Bassham cycle; CRISPR, clustered regularly interspaced short palindromic repeats; DMSP, dimethylsulfoniopropionate; DNRA, dissimilatory nitrate reduction to ammonium; PHA, polyhydroxyalkanoate; reverse TCA cycle, reverse tricarboxylic acid cycle; SOX system, sulfur-oxidizing system; TMA, trimethylamine; Type 1 RC core complex (GSB), Type 1 reaction centre core complex (green sulfur bacteria)

**Fig. 10:**
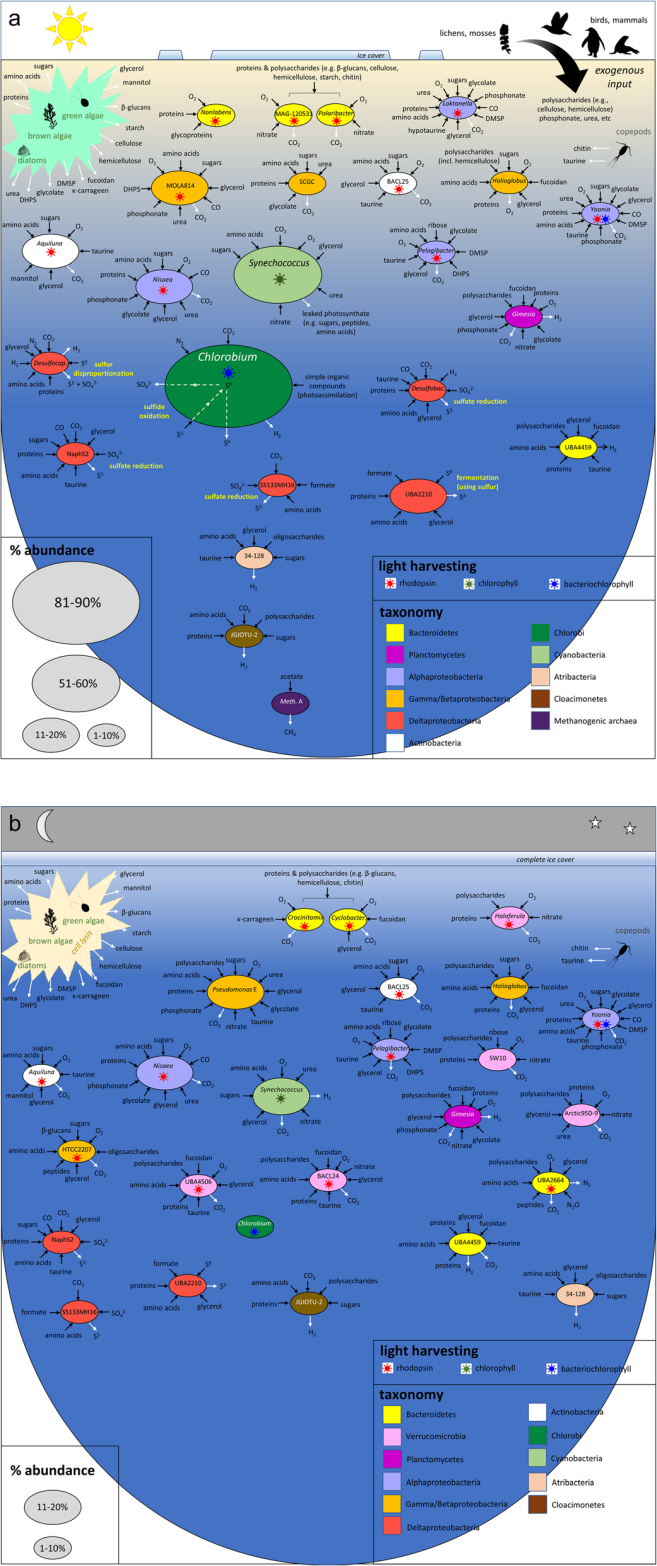
Seasonal nutrient cycles in Ace Lake. Nutrient cycling processes depicted for the most abundant Ace Lake bacteria and archaea occurring in summer (**a**) and winter (**b**). The nutrient cycles for each season integrate data for taxa peak relative abundance (Figs. 4 and 5; Additional file 1: Dataset S1) and functional potential (Fig. 9; Additional file 1: Table S5-S7), highlighting in particular changes in light-driven processes in the upper zone and interface. Also shown are seasonal differences in ice cover and sunlight penetration (yellow-blue gradient); inputs from algae and exogenous sources; and the peak relative abundances of the major OTUs that contribute to the nutrient cycles (ellipse size). *Chlorobium* marks the interface between the upper and lower zones. Winter (**b**) does not include the lower, anoxic zone as it was not sampled during winter. 34-128, Atribacteria 34-128; Arctic95D-9, Verrucomicrobia Arctic95D-9; BACL24, Verrucomicrobia BACL24; BACL25, Microbacteriaceae BACL25; Desulfobac., *Desulfobacterium*; Desulfocap., *Desulfocapsa*; JGIOTU-2, Cloacimonetes JGIOTU-2; HTCC2207, Porticoccaceae HTCC2207; MAG-120531, Flavobacteriaceae MAG-120531; Meth. A, *Methanothrix_A*; MOLA814, Burkholderiaceae MOLA814; NaphS2, Desulfatiglanales NaphS2; S5133MH16, Desulfobacterales S5133MH16; SCGC, Burkholderiaceae SCGC-AAA027-K21; SW10, Verrucomicrobia SW10; UBA2210, Syntrophales UBA2210; UBA2664, Balneolaceae UBA2664; UBA4459, Bacteroidales UBA4459; UBA4506, Verrucomicrobia UBA4506; DHPS, 2,3-dihydroxypropane-1-sulfonate; DMSP, dimethylsulfoniopropionate. Image credits: Animal and plant silhouettes are courtesy of PhyloPic [42]

The abundance of enzymes or pathways involved in energy conservation and metabolism (KEGG analysis; Fig. 9), the metabolic traits of abundant OTUs (Additional file 1: Table S5), the glycoconjugate degradation enzymes of abundant OTUs (Additional file 1: Table S6), the hydrogenases of abundant OTUs (Additional file 1: Table S7), and the relative abundance of the major OTUs (Figs. 4 and 5; Additional file 1: Dataset S1) were integrated to generate seasonal nutrient cycles (Fig. 10). Three carbon fixation processes predominate in Ace Lake: CBB cycle, the rTCA cycle, and the Wood-Ljungdahl (WL) pathway (reductive acetyl-CoA pathway). The CBB cycle is linked to *Synechococcus*, which uses it for oxygenic photosynthesis in the oxic upper layers (U2 and U3). *Chlorobium*, which dominates the interface, employs a light-driven rTCA cycle with sulfide as the electron donor; thus, this carbon fixation pathway was dominant at the interface, where it showed a marked seasonal variation based on *Chlorobium* abundance. *Chlorobium* sinking into the lower zone contributed to the persistence of genes associated with the rTCA cycle. However, we infer that other bacteria that encode the rTCA cycle (e.g. Cloacimonetes JGIOTU-2) may have an active cycle that is fuelled by hydrogen oxidation (see the “Hydrogen cycling: pivotal to multiple nutrient cycles” section). The WL pathway was found exclusively in the lower zone, with its abundance linked to sulfate-reducing Deltaproteobacteria and methanogenic archaea. The latter includes hydrogenotrophic and acetoclastic methanogens, although in acetoclastic methanogens, the WL pathway is not used for carbon fixation [43].

Organic nutrients can be supplied by the primary producing algae and bacteria via exudation or cell lysis. Other nutrients are generated by Ace Lake zooplankton within the water column (i.e. the copepod *P. antarctica* – also see Additional file 3: Movie S1) and a more diverse range of organisms (algae, zooplankton, diatoms, bacteria) colonizing benthic mats, or are of exogenous origin, generated by microbial mats, birds, and seals [5]. While exogenous input would be at its highest in summer when the surface ice has melted and snow-melt drains into the lake, any seasonal changes in the availability of organic material have little impact on catabolic pathways and enzymes (as judged by KEGG analysis).

Recalcitrant polymeric algal material and particulate matter can be remineralized by bacteria throughout the Ace Lake water column. The genomic potential to catalyse the depolymerization of polysaccharides (e.g. chitin, starch, laminarin, cellulose, pectin, fucoidan, carrageenan) was associated with Bacteroidetes, Verrucomicrobia, Planctomycetes, Gammaproteobacteria, and Cloacimonetes OTUs. Bacteroidetes, Verrucomicrobia, and Planctomycetes OTUs also encode capacities for the degradation of fucoidan (fucosidases, sulfatases, fucose catabolic enzymes), which has the potential to be used as both a carbon and sulfur source. Of these groups, it is notable that the most abundant Verrucomicrobia OTUs fell to below 1% relative abundance in summer, suggesting that their higher abundance in winter (Additional file 1: Dataset S1) may be linked to recalcitrant organic matter that is more abundant in winter, especially polymeric substrates that derive from decaying algae.

Algae (including brown algae, green algae, diatoms) and zooplankton (including copepods) release organic solutes such as glycolate, simple sugars, amino acids, peptides, methanol, urea, organosulfonates, and phosphonates [44–50] (Additional file 1: Supplementary text). Although present in *Synechococcus* to detoxify endogenous glycolate (a photorespiratory by-product), the abundance of glycolate oxidase genes in Ace Lake is largely attributable to the breakdown of glycolate by heterotrophic bacteria. Amino acid and peptide primary transporters were found across the examined Ace Lake OTUs, although some differential abundances were apparent, with general amino acid ABC transporters predominating in the upper, oxic zone, and ABC transporters for branched-chain amino acids (BCAA) and peptides more prevalent in the lower, anoxic zone. The latter accords with peptide and amino acid oxidation (especially BCAA) by phylogenetically diverse anaerobes, with these substrates potentially used as carbon, nitrogen, and energy sources [51–53]. Methanol has the potential to be utilized as a carbon and energy source by Ace Lake methylotrophs. Urea transporters and urea catabolism genes were detected throughout the water column, although they were more prevalent in the upper zone due to their presence in *Synechococcus* and many aerobic Proteobacteria and Verrucomicrobia. Taurine uptake and catabolism genes were also more prevalent in the oxic layers, and taurine was predicted to be used as a nitrogen source by many OTUs, with some also using it as a sulfur source. Another organosulfonate, dimethylsulfoniopropionate (DMSP), is a potential source of carbon and sulfur and is a preferred source of reduced sulfur for roseobacters (Additional file 1: Supplementary text) [54] and *Pelagibacter* [55]; accordingly, DMSP catabolism genes were limited to the upper, oxic zone of the lake. Based on catabolic pathways encoded in Ace Lake OTUs, glycine betaine can be utilized as a source of glycine by both aerobic and anaerobic bacteria, although for certain sulfate-reducing Deltaproteobacteria, the methyl group of glycine betaine provides a source of reductant for anaerobic respiration [56]. The capacity to degrade phosphonates is exhibited by several Ace Lake OTUs that encode enzymes to degrade methylphosphonate and 2-aminoethylphosphonate as sources of phosphorus and, in the case of the latter, nitrogen [47, 57]. See Additional file 1: Supplementary text for more description about carbon cycling.

### Sulfur cycling: a tangled web

*Chlorobium* and the anaerobic, sulfate-reducing bacteria (SRB) constitute a sulfur cycle, whereby *Chlorobium* oxidizes sulfide to sulfate and the SRB reduce sulfate to sulfide [10, 11]. The Deltaproteobacteria *Desulfobacterium*, Desulfobacterales S5133MH16, and Desulfatiglanales NaphS2 were the most abundant SRB in Ace Lake, and encode the ability to respire by dissimilatory sulfate reduction (DSR) to sulfide. Certain other anaerobic Deltaproteobacteria present in the lake lack the ability for DSR, including the abundant OTUs *Desulfocapsa* and Syntrophales UBA2210. *Desulfocapsa* can disproportionate sulfur (and thiosulfate) to sulfide and sulfate, thereby providing potential substrates for both *Chlorobium* (sulfide) and SRB (sulfate) [58–60]. Syntrophales UBA2210 is inferred to reduce sulfur to sulfide as part of a fermentative metabolism. *Chlorobium* is a potential major source of sulfur: extracellular sulfur globules (generated and mobilized via water-soluble polysulfides) are an intermediate in sulfide oxidation [61]. Additionally, *Desulfocapsa* is likely to be dependent on the ability of *Chlorobium* to scavenge sulfide, since sulfur disproportionation can only proceed in environments where sulfide levels are kept low [60, 62].

*Desulfobacterium* and *Desulfocapsa* exhibited seasonal variation between summer and winter, which may be linked to the marked decline of *Chlorobium* (see the “The importance of sunlight” section). Both these Deltaproteobacteria OTUs fell to below 1% relative abundance in winter (Additional file 1: Dataset S1). Further, *Chlorobium* relative abundance was positively correlated with the relative abundance of *Desulfobacterium* (*r* = 0.94, *P* = 0.002, 3 μm size fraction) and *Desulfocapsa* (*r* = 0.81, *P* = 0.026, 3 μm size fraction; *r* = 0.8, *P* = 0.031, 0.8 μm size fraction). By contrast, the relative abundances of Desulfatiglanales NaphS2, Desulfobacterales S5133MH16, and Syntrophales UBA2210 were each negatively correlated with *Chlorobium* relative abundance, although the relationship was not significant.

The Ace Lake *Chlorobium* lacks the Sox system for thiosulfate utilization to sulfate [11]. The considerable sequence coverage for the *Chlorobium* OTU, and knowledge that the *sox* gene cluster is encoded on a mobile genetic element in some Chlorobiaceae [63] indicates *sox* genes are absent in the Ace Lake *Chlorobium* population. However, Sox systems are present in diverse Alpha- and Betaproteobacteria consistent with the presence of KEGG hits in the upper, oxic zone. These proteobacteria also potentially provide a source of sulfate to the lake that augments the major contribution made by *Chlorobium*.

DSR is dominant in the anoxic, lower zone due to SRB belonging to Deltaproteobacteria. The detection of DSR-related genes in the upper, oxic zone is attributed mainly to the presence of adenylylsulfate reductase genes (*aprAB*) in *Pelagibacter*; however, rather than being employed for DSR, it has been proposed that this enzyme is employed solely for the detoxification of sulfite (such as generated by taurine catabolism) to adenosine 5′-phosphosulfate [8].

Typical of GSB, *Chlorobium* in Ace Lake lacks any genomic evidence of assimilatory sulfate reduction (ASR) and therefore requires exogenous sulfide for biosynthetic purposes [64]. Accordingly, based on the abundance of ASR-related genes within the water column, this process was lowest at the interface at the times when *Chlorobium* was most abundant. ASR-related genes were prevalent throughout the water column, with numerous bacteria capable of using inorganic sulfate as a source of biosynthetic sulfur, including *Synechococcus*, Verrucomicrobia, various Proteobacteria (but not Deltaproteobacteria), and certain Bacteroidetes.

### Nitrogen cycle: all about ammonia

Nitrogen appears to be the limiting nutrient in the upper zone due to its rapid depletion at the onset of primary productivity in spring [5]. Bacteria in the lower zone contribute to replenishment of ammonium and other reduced nitrogen in the upper zone through degradation of amino acids [5, 10]. The Ace Lake metagenome data is dominated by genes for nitrogen fixation and ammonia assimilation, with no evidence for nitrification and a low capacity for denitrification. *Chlorobium* and *Desulfocapsa* encode nitrogenases; however, *Chlorobium* abundance is highly dependent on season, and diazotrophy would only be necessary when ammonia levels are low [10, 11]. Several OTUs exhibit the genomic potential for incomplete denitrification: respiratory nitrate reductase (NarG) (*Nisaea*), periplasmic nitrate reductase (NapA) (Verrucomicrobia BACL24; *Pseudomonas* E; *Desulfocapsa*) for nitrate dissimilation, nitrite reductase (nitric oxide forming) (NirK) (Balneolaceae UBA2664, *Nisaea*, *Pelagibacter*, Bacteroidales UBA4459), and nitrous oxide reductase (NosZ) (Balneolaceae UBA2664, Bacteroidales UBA4459). There is also the potential for dissimilatory nitrate reduction to ammonia via cytochrome *c* nitrite reductase (NrfA) (*Desulfocapsa*). The Ace Lake metagenome data is dominated by genes for nitrogen fixation and ammonia assimilation, with no evidence for nitrification and a low capacity for denitrification.

### Hydrogen cycling: pivotal to multiple nutrient cycles

Multiple types of hydrogenases are represented among Ace Lake bacteria and archaea, including respiratory uptake hydrogenases, and hydrogenases linked to fermentation and/or redox balance (Additional file 1: Table S7). Hydrogenases were more abundant in the lower zone than the upper zone (Fig. 9), where they are associated with the use of hydrogen as an energy source and/or production of hydrogen as part of anaerobic respiration or fermentation. Hydrogen-evolving hydrogenases can be used by aerobes that adopt a fermentative metabolism in response to oxygen limitation, and are employed for survival rather than growth (e.g. *Gimesia*) [65]. *Synechococcus* has the genomic potential for fermentative growth under prolonged anoxic conditions, including hydrogen evolution via a Hox hydrogenase (see the “The importance of sunlight” section). Thus, *Synechococcus* is a potential source of hydrogen in Ace Lake. Ace Lake *Chlorobium*, which lacks any evidence of hydrogenases, is potentially also a major source of hydrogen [10], derived from a membrane-bound nitrogenase that generates hydrogen as a by-product of nitrogen fixation [66]. Thus, hydrogen production by *Chlorobium* would be seasonal, and occur only when ammonia is limiting [10].

Anaerobes such as sulfate-reducing Deltaproteobacteria (*Desulfobacterium* and Desulfatiglanales NaphS2) and hydrogenotrophic methanogens (Methanomicrobiaceae 1) have hydrogen-uptake hydrogenases that exploit hydrogen as an energy source for carbon fixation via the WL pathway, coupled to the reduction of sulfate to sulfide or the reduction of CO_2_ to methane, respectively. Ace Lake *Desulfocapsa* encodes both a nitrogenase and a membrane-bound hydrogenase (Additional file 1: Table S5); this suggests that hydrogen released as a by-product of nitrogen fixation can be re-oxidized to protons, thus recovering the electrons for energy conservation. Hydrogen oxidation is also inferred for other OTUs using heterodisulfide-linked hydrogenases that could be employed for biosynthetic purposes (Bacteroidales UBA4459, Cloacimonetes JGIOTU-2) [67]. Certain anaerobic bacteria (Atribacteria 34-128, Cloacimonetes JGIOTU-2) were inferred to have respiratory systems that include a membrane-bound hydrogenase, which utilizes protons both as a substrate for hydrogen production and for ion translocation, with reductant provided by oxidation of organic substrates [68]. Cytosolic hydrogenases serve as hydrogen-evolving hydrogenases to re-oxidize redox carriers during fermentative growth by obligate anaerobes (Bacteroidales UBA4459, *Desulfocapsa*, Desulfatiglanales NaphS2, Atribacteria 34-128, *Izimaplasma*) [69]. These data for hydrogen cycling in Ace Lake are consistent with the proposal that microbial hydrogen metabolism is highly important to ecosystem function in both oxic and anoxic environments [70].

## Conclusions

The microbial community in Ace Lake is defined by a marine-seed population that was isolated from the Southern Ocean ~ 5000 year ago [10]. While chemolithoautotrophic Thaumarchaeota thrive in the Southern Ocean in winter [7, 8] and occupy an equivalent niche in some Antarctic lakes (e.g. Lake Bonney) [9], the community in Ace Lake has not retained this capacity. Instead, phototrophic processes drive primary production with major contributions from *Synechococcus* and *Chlorobium*, with less abundant SRB and other bacteria capable of chemolithoautotrophy via the WL pathway supplementing photoautotrophic carbon fixation. The Southern Ocean supports a very low abundance of *Synechococcus*, other cyanobacteria, and cyanophage, and only certain marine-derived Antarctic lakes and ponds support planktonic and/or microbial mat populations of cyanobacteria (Ref. [71] and papers within). Rather than Chlorobi, phototrophic purple bacteria are important primary producers in Antarctic Lake Fryxell [72]. GSB are present in a number of stratified aquatic systems in the Vestfold Hills [15] and are abundant in some Arctic lakes (e.g. *Pelodictyon* in Lake A [73]), but have not been reported to be generally abundant in Antarctic aquatic systems. Given the observed distinctiveness of the limited number of Antarctic aquatic systems that have been examined using metagenomics [2], there is a strong case to expand the metagenomics base and characterize the dynamic of those systems. Our study of microbial community changes throughout a complete seasonal cycle in Antarctica includes 120 metagenomes that also represent a summer time-series. An important finding from this seasonal assessment is, while viruses are abundant, particularly in the more seasonally dynamic photic zone, their total abundance varies little with season and they do not appear to strongly impact the life cycle of their hosts. In Organic Lake (Vestfold Hills) a virophage was predicted to promote carbon flux mediated by algae [74], and in Deep Lake (Vestfold Hills) viruses of haloarchaea were hypothesized to protect hosts against more destructive viruses by distributing mutated cell surface genes within the lake population [75]. Akin to the viruses of haloarchaea, we speculate here that huge phage provide a similar service to their anaerobic bacterial hosts. While viruses undoubtedly contribute to turnover and nutrient cycling in Antarctic aquatic systems, Antarctic viruses may tend to be less predatory and more enterprising in the ways they interact with their hosts than their cousins from warmer environments. Teasing apart the effects of low temperature from the polar light regime on characteristics of virus-host interactions may be achievable through comparative metagenome analyses of lake systems from the Tibetan Plateau (the ‘third pole’) where light cycles are defined by the 33° N latitude location of the lakes, compared to those in the Vestfold Hills at 68° S.

## Methods

### Biomass collection and metadata

Descriptions are provided (with photographs) for the 2013–2015 Ace Lake (− 68.47253999, 78.18801996) sampling expedition (Additional file 1: Fig. S1) and the 120 metagenomes analysed (Additional file 1: Table S1). Biomass was collected by sequential size fractionation through a 20-μm prefilter onto 3.0-, 0.8-, and 0.1-μm pore-sized, large format (293-mm polyethersulfone membrane) filters and DNA was extracted from the biomass as described previously [11, 76, 77]. Samples taken in 2008/2009 and 2013/2014 and 2014/2015 included approximately the same six depths (U2, U3, I, L1, L2, and L3) that were sampled in 2006/2007 [10]. In summer when lake ice melted (2013–2015), surface samples were taken from the shore (U1) (Additional file 1: Fig. S1). In winter, samples were taken to the depth of the interface (U2, U3, I) but were not taken from the lower anoxic zone because the focus was on seasonal changes in the photic zone, and working within the enclosed mobile work shelters in winter resulted in hydrogen sulfide levels accumulating to unsafe levels thereby precluding sampling. Depth, salinity, and lake temperature were typically measured during sample collection (Additional file 1: Fig. S8 and Table S9). Data for air temperature, wind velocity, and sunlight hours were obtained from the Australian Antarctic Data Centre, Australia, using measurements from Davis Station which is ~ 15 km from Ace Lake. Daylength data was obtained for Davis Station from a web service [78]. Air temperature was an average of maximum and minimum daily temperature, and daylength was the daily number of hours the sun was above the horizon. The sunlight hours were the number of hours of bright sunshine (determined using a Campbell-Stokes sunshine recorder), excluding hours of cloud cover (Additional file 1: Table S9).

### Metagenome sequencing, assembly, annotation, and overview of analyses

Metagenome sequencing of the 2006/2007 samples was performed using Sanger and 454 technologies [10, 11]. The 2008/2009 and 2013–2015 samples were sequenced using Illumina technology, as described previously [77]. Assembly was performed with metaSpades in-house (2006/2007 data) or at the Joint Genome Institute (2008/2009 and 2013–2015 data), and all contigs > 200 bp were uploaded and annotated by the IMG pipeline [79]. The IMG pipeline also generated medium- and high-quality IMG MAGs from each metagenome sequenced and annotated at JGI; the MAGs are available with their respective metagenomes in IMG (see metagenome IDs in Additional file 1: Table S1). In addition to the individual assembled metagenomes, a single co-assembly of all qc-filtered raw reads was produced using Megahit v1.1.1 with the setting --meta-large [80]. Note that two metagenomes did not have their qc-filtered raw reads included (Nov2013_L3_0.1μm and Dec2014_I_3.0μm) because they were not available at the time of co-assembly. Individual metagenome reads were mapped to the co-assembly, a read depth file was created, and bins (MetaBAT MAGs) were generated using MetaBAT v.2.12.1 with minContig length 2500 bp [12]. CheckM v. 1.0.7 [81] was used to calculate MetaBAT MAG completeness and contamination, and RefineM v. 0.0.23 [82] was used to assess taxonomic identity of the MetaBAT MAGs. In order to facilitate detection of viruses in subsequent analyses, all unbinned long contigs (≥ 10 kb) from the co-assembly were retained along with the dataset of MetaBAT MAGs.

The Ace Lake time series sequencing and assembly data were used for the following analyses: contig taxonomy classification, identification of OTUs, relative abundance calculations, and assessment of the functional potential of abundant OTUs. The taxonomies of the protein-coding genes in IMG (phylodist file) were used for assignment of contig taxonomy, using the following criteria: (a) at least 30% of the genes identified on a contig must have a taxonomic assignment, if not, then the contig was unassigned; (b) for a contig, the taxon to which most of its genes belonged was used as the taxonomic assignment of that contig; and (c) if all genes on a contig had different taxonomies, the contig was unassigned. Additionally, contigs with no identified genes or genes that could not be assigned any taxonomy were denoted ‘unassigned’. The small subunit (SSU) rRNA genes on the unassigned contigs were aligned against the NCBI-nr nucleotide database using blastn (Blast+ v2.9.0), and the IMG gene annotations of contigs ≥ 1 kb were manually analysed to assess the composition of the unassigned contigs. SSU rRNA genes within unassigned contigs were used to assign domain level taxonomy. VirSorter v.1.0.3 was used to screen unassigned contigs against the virome database through the CyVerse Discovery Environment [83] to identify potential viruses and prophages [84, 85]; all unassigned contigs from the 2006 metagenomes were screened, along with all unassigned contigs > 10 kb from the 2008/2009 and 2013–2015 metagenomes, as well as 1–10 kb unassigned contigs from 2008/2009 and 2013–2015 metagenomes that had relative abundance ≥ 0.1% in at least one metagenome or had a read depth ≥ 200. Only the most confident and likely predictions of viruses (category 1 and 2) and prophages (category 4 and 5) were used. Based on final taxonomic assignments, contigs were binned as OTUs at the lower taxonomic rank available.

### Calculations of taxonomic abundance

The relative abundance of an OTU in a metagenome was calculated using the formula:

$$ \frac{\sum_{\mathrm{OTU}}\left({\mathrm{ContigLength}}_{\mathrm{i}}\times \mathrm{Read}\ \mathrm{depth}\ {\mathrm{of}\ \mathrm{contig}}_{\mathrm{i}}\right)}{\sum_{\mathrm{metag}}\left({\mathrm{ContigLength}}_{\mathrm{j}}\times \mathrm{Read}\ \mathrm{depth}\ {\mathrm{of}\ \mathrm{contig}}_{\mathrm{j}}\right)}\times 100 $$

where metag is a metagenome, OTU is an OTU in a metagenome, i refers to contigs belonging to the OTU in a metagenome, and j refers to all contigs in a metagenome.

These relative abundance measurements are based on contigs. The numerator is the abundance of an OTU in a metagenome and is the sum of the coverages (contig read depth × contig length) of contigs assigned to the OTU; this abundance was calculated using a python script. The denominator represents the total abundance of all contigs in a metagenome and is the sum of the coverages of all contigs in a metagenome that represent the 51 abundant OTUs, ‘Other’ Bacteria, Eukarya, Archaea or Virus OTUs, and ‘unassigned’ contigs. The relative abundance calculations were performed using Primer 7 (Primer-e, NZ). All relative abundance values reported are comparable because the denominator is consistent across calculations. Peak relative abundance of OTUs describes the highest relative abundance of an OTU in the 120 Ace Lake metagenomes, unless otherwise specified (e.g. within a season, time period, filter fraction, lake depth). To assess overall virus relative abundance in each of the Ace Lake metagenomes, all virus OTUs were grouped at the phylum level. The OTUs included five *Phycodnaviridae* OTUs that were part of the 51 abundant OTUs, plus ‘Other’ Virus OTUs, and included Unassigned contigs that were confidently predicted by VirSorter as viruses (category 1 and 2) and prophages (category 4 and 5). Some of the phylum-level categories were grouped as ‘Other’ viruses, either because their relative abundance was very low in most metagenomes (< 1% relative abundance) or their taxonomy was ambiguous (e.g. unclassified viruses or unassigned viruses). For abundance calculations of viral clusters, see the “Viral analyses” section.

### Statistical analyses

The environmental factors were normalized in Primer 7 before abundance data from the 120 metagenomes were analysed (Additional file 1: Table S9). Sample collection date, depth, filter fraction, and season were used as factors to group metagenome samples (Additional file 1: Table S1). For each OTU, taxonomic profiles from genus to domain were added as indicators to group species variables. For all analyses, relative species abundances were square root-transformed and Bray-Curtis similarity was used to generate resemblance matrices. Similarity percentage (SIMPER) analysis was performed on transformed relative abundances to determine the highest contributing OTUs at different depths across the three seasons (Additional file 1: Tables S3 and S4); similarity was calculated within a season and dissimilarity was calculated between seasons. Simpson’s index of diversity was calculated for all metagenomes as a measure of alpha diversity. A distance-based linear model (distLM) was used to explore the relationship between the variations in OTU relative abundances across samples and environmental factors (lake depth, lake salinity, air temperature, daylength, and sunlight hours). The lake temperature at different depths was not used, as it was not recorded for all sampling periods. A dbRDA plot was generated to fit the Bray-Curtis similarity matrix using a step-wise variable selection procedure, with adjusted *R*^2^ as the fitness measure. Statistical analyses (Pearson product moment correlation, ANOVA (analysis of variance) regression analysis for statistical significance) were performed to identify correlations between the abundances of certain OTUs.

### Abundant OTUs: bin refinement, reclassification, and analysis of functional potential

The contigs belonging to the abundant OTUs were binned in their respective OTU bins, which were refined using RefineM v0.0.24 software with default parameters, except --cov_perc 1000000000 option to prevent it from filtering based on coverage [82]. All metagenomes from specific depths in which the relative abundance of an OTU was high were considered for refinement of that OTU bin. For example, the *Chlorobium* bin was refined using filtered reads from only interface metagenomes, whereas the *Halioglobus* bin was refined using filtered reads from upper zone (U2 and U3) metagenomes (Additional file 1: Dataset S1). The filtered OTU bins were further aligned against the MetaBAT MAGs using blastn module of Blast+ v2.9.0. The OTU bins that were mixed bins according to RefineM output and/or had spurious hits to multiple MetaBAT MAGs were considered low confidence OTUs and were excluded from further analysis. Based on the best hits to MetaBAT MAGs, some of the species-level OTUs were merged and reclassified to genus level. For example, *Candidatus* Pelagibacter ubique and *Candidatus* Pelagibacter sp. IMCC9063 were merged together as *Pelagibacter*. In addition, some species-level OTUs were merged and reclassified to a higher taxa level and then split to genus-level OTUs. For example, 5 abundant verrucomicrobial OTUs (*Coraliomargarita akajimensis, Chthoniobacter flavus, Haloferula* sp. BvORR071*, Prosthecobacter debontii*, and *Rubritalea squalenifaciens*) had good hits to multiple Verrucomicrobia MetaBAT MAGs, so the OTUs were merged to a higher taxa level common to all 5 (Verrucomicrobia) and then split into 5 genus-level OTUs to which they had best Blast hits (Verrucomicrobia SW10, Verrucomicrobia UBA4506, Verrucomicrobia BACL24, Verrucomicrobia Arctic95D-9, and *Haloferula*). In addition to 51 high-quality OTU bins, a Parcubacteria bin was identified which was split into 6 OTUs based on best hits to MetaBAT MAGs. For the alphanumeric genus OTUs, a higher taxa level (phylum, order, or family) was added to the names for context. All low abundance OTUs (relative abundance < 1% in all metagenomes) and low confidence OTUs were grouped as other archaea, bacteria, eukarya, or viruses (Additional file 1: Dataset S1; Additional file 2: Fig. S1). To validate the taxonomic classification of the abundant OTUs, their protein match identity, ANI, and SSU rRNA gene identity were calculated against reference genomes. The pyani v0.2.9 package installed in python v3.6.5 was used to calculate the ANI of the filtered OTU bins against the reference genomes [86]. For aligning the DNA sequences, blastn module of Blast+ v2.9.0 was used (Additional file 1: Table S8). The relative abundances of the filtered OTUs were recalculated in metagenomes from specific depths (Additional file 1: Dataset S1), and the filtered OTUs were used to assess the functional contribution of the abundant microbes. Within each filtered bin, the contigs with ≤ 2 open reading frames were excluded from the analysis. Some OTUs (e.g. the 6 Parcubacteria OTUs) lacked sufficient number of genes in their bins to be able to reliably infer functional potential and were excluded from function-based analyses. All annotations of genes involved in pathways and processes were manually examined in a similar way to a previous assessment of the veracity of gene functional assignments [87]. Protein sequences that were identified as hydrogenases based on catalytic domains were classified further using the hydrogenase classifier HydDB [88].

### KEGG-based analysis of functional potential

The overall functional potential of Ace Lake was analysed using the KEGG orthology (KO) terms for specific pathways/enzymes (Additional file 1: Tables S10 and S11). To calculate the abundances of the pathways/enzymes in each metagenome, the IMG KEGG annotation files were used [79]. However, for enzymes that catalyse redox reactions (e.g. sulfur oxidation/reduction) or reactions catalysed by homologous enzymes (e.g. ammonia/methane monooxygenase), databases were created that included the protein sequences and previously observed functional roles. To assign a specific role, the protein sequences of the KO annotations from the metagenomes were aligned against the respective databases using the pairwise2 package of biopython (python v3.6.5; pairwise2.align.localms(query.seq, reference.seq, 2, -1, -.5, -.1, score_only=1)). The abundances of the selected KEGG numbers were calculated by summing the read depth of contigs corresponding to predicted genes assigned KO annotations (Additional file 1: Table S10). The KEGG abundances were used to calculate the normalized abundance of a pathway or an enzyme in a metagenome using the formula:

$$ \frac{\sum_{\mathrm{P}/\mathrm{E}}\left({\mathrm{abundance}}_{\mathrm{KEGG}}\right)}{\sum_{\mathrm{metag}}\left({\mathrm{Read}\ \mathrm{depth}}_{\mathrm{cont}}\right)}\times \left(\frac{\sum_{\mathrm{all}}\left({\sum}_{\mathrm{metag}}\left({\mathrm{Read}\ \mathrm{depth}}_{\mathrm{cont}}\right)\right)}{N}\right) $$

where metag is a metagenome, cont are contigs corresponding to predicted genes assigned KO annotations in a metagenome, P/E is a pathway/enzyme in a metagenome, KEGG refers to all KEGG numbers associated with the pathway/enzyme (Additional file 1: Table S9), all refers to all metagenomes in Ace Lake, and *N* is total number of metagenomes (120).

These abundance calculations are based on predicted genes in metagenomes. The numerator (left-hand) is the abundance of a pathway/enzyme in a metagenome and was calculated using a python script to sum and/or average the abundances of selected KEGG numbers associated with it (Additional file 1: Table S10). The denominator (left-hand) is the sum of read depths of contigs corresponding to predicted genes with KO annotations in a metagenome. The pathway/enzyme abundances were normalized relative to the mean of the sum of read depths of contigs corresponding to predicted genes with KO annotations from all 120 metagenomes (right-hand). The abundant organisms that probably contributed to a pathway/enzyme were also identified.

### Phylogeny

Multiple alignment of rhodopsin amino acid sequences (56 sequences) was performed using MUSCLE [89] and maximum-likelihood phylogenetic trees were constructed with the JTT matrix-based model [90] in MEGA6 [91]. Only positions with less than 20% alignment gaps or missing data were included in the analysis, resulting in 229 aa positions in the final dataset.

### Viral analyses

A catalogue of viruses was generated using an approach described previously [92], incorporating virus contig sequences exclusively from Antarctic metagenomes, their virus cluster or singleton assignments based on IMG/VR database clustering and their matches to spacers from IMG metagenomes and genomes [39]. For sequence clustering, the Ace Lake virus catalogue was combined with the IMG/VR 2.0 database, compared using blastn (BLAST+ v2.9.0), and clustered based on nucleotide sequence similarity with standard threshold (≥ 95% ANI and ≥ 85% alignment fraction) [39, 93]. Ace Lake viral contigs were either classified into clusters (groups of 2 or more sequences) or as singletons. For host assignment, Ace Lake viral contigs were compared to the IMG/VR spacer database using blastn (BLAST+ v2.9.0) with specific options for short sequences (word size 7, no dust filtering) [39]. Additional catalogues were generated for NCLDVs (2,296 contigs) and virophages (69 contigs) based on the detection of specific marker genes, namely NCLDV and Virophage major capsid proteins, using hmmsearch [94, 95]. The contigs of *Phycodnaviridae* 1–5 OTUs were compared against the Ace Lake virus and NCLDV catalogues to identify matching virus clusters and singletons. Spacer sequences in *Chlorobium* CRISPR arrays (in the IMG CRISPR annotation files) identified in 8 interface metagenomes (Dec. 2006 0.1 μm and 3 μm; Nov. 2013 0.1 μm; Jul. 2014 0.1, 0.8, and 3 μm; Aug. 2014 0.1 μm; Dec. 2014 0.1 μm) were used to assess potential Ace Lake *Chlorobium* viruses. The spacer sequences were aligned against all the assembled Ace Lake metagenome contigs using the -megablast option of Blast+ v2.6.0, with *e*-value 0.001 and 97% identity cut-off. By comparing the contigs with matches to *Chlorobium* spacers to the Ace Lake virus catalogue, potential *Chlorobium* virus contigs were identified. Host contigs were identified by analysing the spacer matches to the selected virus contigs using the IMG/VR spacer database, and host taxonomy assigned as described above (the “Metagenome sequencing, assembly, annotation, and overview of analyses” section). The representation of virus clusters and singletons was expanded by aligning virus contigs (from clusters and singletons) associated with *Chlorobium* against all Ace Lake metagenome contigs using the blastn mode of Blast+ v2.9.0, with *e*-value 0.0001 and 90% identity cut-off. Metagenome contigs with high identity and a high alignment fraction were incorporated into the cluster or singleton. A cyanophage (549 kb in length) was assembled from the Dec. 2006, 5-m depth, 0.1-μm filter fraction metagenome data (Contig Ga0078900_115654, 3300016486). The assembly was aligned against the contigs in the Ace Lake virus catalogue using the blastn mode of Blast+ v2.9.0, with high identity (~ 100%) matches used to identify virus clusters and singletons. To determine the abundance of sequences representing viral clusters or singletons, the read depth of contigs corresponding to a cluster and from a time period (e.g. Dec. 2006) were summed to obtain their total read depth in that time period. Those with total read depth > 4000 (which equates to 0.6-fold of the maximum *Chlorobium* read depth) in at least one time period were considered to be abundant. Potential hosts of all clusters were explored using IMG/VR (Additional file 4: Table S8) and hosts of clusters abundant in interface and lower zones were further identified using the IMG/VR spacer database. Correlation analyses were performed (see the “Statistical analyses” section) using the average read depth of specific marker genes for *Chlorobium* (16S rRNA, recombinase A and bacteriochlorophyll A) and S*ynechococcus* (16S rRNA and recombinase A) with their respective viruses, and between *Micromonas* and *Phycodnaviridae* 1–5 using their relative abundances. The putative *Chlorobium* viral contigs were annotated using Prokka v1.13 [96] and VirSorter v1.0.5 [93]. To assess similarity to known viral genomes, viral contigs were first dereplicated (95% ANI, 85% alignment fraction) using MUMMER v4.0.0b2 [97]. The resulting dataset was used to build a genome network based on shared gene content using vContact 2 [98], using diamond for all-vs-all protein comparison, MCL for protein clustering, and ClusterOne for genome clustering, yielding approximately genus-level groupings (Additional file 4: Dataset S1). The viral contigs represented partial genomes and could not be determined as temperate or lytic phage, and genome annotation did not reveal any putative auxiliary metabolic genes. Complete, circular virus genomes were identified in the 120 Ace Lake metagenomes and their classification determined based on the presence of VOG marker genes, identified using hmmsearch [99]. Viral contigs were classified into 173 distinct viruses based on their GC content, length, virus cluster assignment, and alignment using Mauve v2.4.0 [100]. The virus contigs from individual metagenomes were aligned against the sequence of the huge phage (November 2008, L1, 0.1 μm; Contig Ga0208769_1000001) using ‘align with progressive mauve’ mode of Mauve v2.4.0 [42] to determine if the contigs represented complete (or nearly complete) phage sequences.

## Supplementary information

**Additional file 1:** Ace Lake carbon cycle, expedition information, and taxonomic and functional analyses. Supplementary text, figures, tables and dataset.

**Additional file 2:** Analysis of ‘unassigned’ taxa. Supplementary text, figures and tables.

**Additional file 3:** Ace Lake video. Movie and text description.

**Additional file 4:** Viral analyses. Supplementary tables and dataset.

## Data Availability

All genomes, metagenomes, and medium- and high-quality MAGs (MetaBAT MAGs and IMG MAGs) used in this study are available in IMG: see details in Additional file 1: Table S1 and S8; Additional file 4: Table S4 and S10.

## References

[1] Margesin R, Miteva V (2011). Diversity and ecology of psychrophilic microorganisms. Res Microbiol..

[2] Cavicchioli R (2015). Microbial ecology of Antarctic aquatic systems. Nat Rev Microbiol..

[3] Cavicchioli R (2019). A vision for a 'microbcentric' future. Microb Biotechnol..

[4] Cary SC, McDonald IR, Barrett JE, Cowan DA (2010). On the rocks: the microbiology of Antarctic Dry Valley soils. Nat Rev Microbiol..

[5] Rankin LM, Gibson JAE, Franzrnann PD, Burton HR (1999). The chemical stratification and microbial communities of Ace Lake, Antarctica: a review of the characteristics of a marine-derived meromictic lake. Polarforschung..

[6] Brum JR, Hurwitz BL, Schofield O, Ducklow HW, Sullivan MB (2016). Seasonal time bombs: dominant temperate viruses affect Southern Ocean microbial dynamics. ISME J..

[7] Grzymski JJ, Riesenfeld CS, Williams TJ, Dussaq AM, Ducklow H, Erickson M (2012). A metagenomic assessment of winter and summer bacterioplankton from Antarctica Peninsula coastal surface waters. ISME J..

[8] Williams TJ, Long E, Evans F, Demaere MZ, Lauro FM, Raftery MJ (2012). A metaproteomic assessment of winter and summer bacterioplankton from Antarctic Peninsula coastal surface waters. ISME J..

[9] Vick-Majors TJ, Priscu JC, Amaral-Zettler LA (2014). Modular community structure suggests metabolic plasticity during the transition to polar night in ice-covered Antarctic lakes. ISME J..

[10] Lauro FM, DeMaere MZ, Yau S, Brown MV, Ng C, Wilkins D (2011). An integrative study of a meromictic lake ecosystem in Antarctica. ISME J..

[11] Ng C, DeMaere MZ, Williams TJ, Lauro FM, Raftery M, Gibson JA (2010). Metaproteogenomic analysis of a dominant green sulfur bacterium from Ace Lake. Antarctica. ISME J..

[12] Kang DD, Li F, Kirton E, Thomas A, Egan R, An H (2019). MetaBAT 2: an adaptive binning algorithm for robust and efficient genome reconstruction from metagenome assemblies. PeerJ..

[13] Hand RM, Burton HR (1981). Microbial ecology of an Antarctic saline meromictic lake. Hydrobiol..

[14] Burch MD (1988). Annual cycle of phytoplankton in Ace Lake, an ice covered, saline meromictic lake. Hydrobiol..

[15] Burke CM, Burton HR (1988). Photosynthetic bacteria in meromictic lakes and stratified fjords of the Vestfold Hills. Antarctica. Hydrobiol..

[16] Powell LM, Bowman JP, Skerratt JH, Franzmann PD, Burton HR (2005). Ecology of a novel *Synechococcus* clade occurring in dense populations in saline Antarctic lakes. Mar Ecol Prog Ser..

[17] Van Baalen C, Hoare DS, Brandt E (1971). Heterotrophic growth of blue-green algae in dim light. J Bacteriol..

[18] Lambert DH, Stevens SE (1986). Photoheterotrophic growth of *Agmenellum quadruplicatum* PR-6. J Bacteriol..

[19] Ludwig M, Bryant DA. *Synechococcus* sp. Strain PCC 7002 transcriptome: acclimation to temperature, salinity, oxidative stress, and mixotrophic growth conditions. Front Microbiol. 2012;3:354. 10.3389/fmicb.2012.00354 PMC3468840 23087677

[20] Chen X, Schreiber K, Appel J, Makowka A, Fähnrich B, Roettger M (2016). The Entner-Doudoroff pathway is an overlooked glycolytic route in cyanobacteria and plants. Proc Natl Acad Sci U S A..

[21] Reed RH, Borowitzka LJ, Mackay MA, Chudek JA, Foster R, Warr SRC (1986). Organic solute accumulation in osmotically stressed cyanobacteria. FEMS Microbiol Rev..

[22] Engelbrecht F, Marin K, Hagemann M (1999). Expression of the *ggpS* gene, involved in osmolyte synthesis in the marine cyanobacterium *Synechococcus* sp. strain PCC 7002, revealed regulatory differences between this strain and the freshwater strain *Synechocystis* sp. strain PCC 6803. Appl Environ Microbiol..

[23] Callieri C, Slabakova V, Dzhembekova N, Slabakova N, Peneva E, Cabello-Yeves PJ (2019). The mesopelagic anoxic Black Sea as an unexpected habitat for *Synechococcus* challenges our understanding of global “deep red fluorescence”. ISME J..

[24] Suzuki E, Ohkawa H, Moriya K, Matsubara T, Nagaike Y, Iwasaki I (2010). Carbohydrate metabolism in mutants of the cyanobacterium *Synechococcus elongatus* PCC 7942 defective in glycogen synthesis. Appl Environ Microbiol..

[25] McNeely K, Xu Y, Ananyev G, Bennette N, Bryant DA, Dismukes GC (2011). *Synechococcus* sp. strain PCC 7002 nifJ mutant lacking pyruvate:ferredoxin oxidoreductase. Appl Environ Microbiol..

[26] Buchanan BB, Arnon DI (1990). A reverse Krebs cycle in photosynthesis: consensus at last. Photosynth Res..

[27] Eisen JA, Nelson KE, Paulsen IT, Heidelberg JF, Wu M, Dodson RJ (2002). The complete genome sequence of *Chlorobium tepidum* TLS, a photosynthetic, anaerobic, green-sulfur bacterium. Proc Natl Acad Sci U S A..

[28] Imhoff JF (2003). Phylogenetic taxonomy of the family *Chlorobiaceae* on the basis of 16S rRNA and *fmo* (Fenna-Matthews-Olson protein) gene sequences. Int J Syst Evol Microbiol..

[29] Maresca JA, Romberger SP, Bryant DA (2008). Isorenieratene biosynthesis in green sulfur bacteria requires the cooperative actions of two carotenoid cyclases. J Bacteriol..

[30] Thweatt JL, Ferlez BH, Golbeck JH, Bryant DA (2017). BciD is a Radical-SAM enzyme that completes bacteriochlorophyllide I biosynthesis by oxidizing a methyl group into a formyl group at C-7. J Biol Chem..

[31] Llorens-Marès T, Liu Z, Allen LZ, Rusch DB, Craig MT, Dupont CL, Bryant DA (2017). Casamayor EO Speciation and ecological success in dimly lit waters: horizontal gene transfer in a green sulfur bacteria bloom unveiled by metagenomic assembly. ISME J..

[32] Béjà O, Spudich EN, Spudich JL, Leclerc M, DeLong EF (2001). Proteorhodopsin phototrophy in the ocean. Nature..

[33] Kim SY, Waschuk SA, Brown LS, Jung KH (1777). Screening and characterization of proteorhodopsin color-tuning mutations in *Escherichia coli* with endogenous retinal synthesis. Biochim Biophys Acta..

[34] Olson DK, Yoshizawa S, Boeuf D, Iwasaki W, DeLong EF (2018). Proteorhodopsin variability and distribution in the North Pacific Subtropical Gyre. ISME J..

[35] Koh EY, Atamna-Ismaeel N, Martin A, Cowie RO, Béjà O, Davy SK (2010). Proteorhodopsin-bearing bacteria in Antarctic sea ice. Appl Environ Microbiol..

[36] Thiel V, Tank M, Bryant DA (2018). Diversity of chlorophototrophic bacteria revealed in the omics era. Annu Rev Plant Biol..

[37] Cai F, Axen SD, Kerfeld CA (2013). Evidence for the widespread distribution of CRISPR-Cas system in the phylum Cyanobacteria. RNA Biol..

[38] Boldyreva D, Babenko VV, Kanygina AV, Lunina ON, Letarova MA, Kostryukova ES (2020). Genome sequences of a green-colored *Chlorobium phaeovibrioides* strain containing two plasmids and a closely related plasmid-free brown-colored strain. Microbiol Resour Announc..

[39] Paez-Espino D, Roux S, Chen IA, Palaniappan K, Ratner A, Chu K (2019). IMG/VR v.2.0: an integrated data management and analysis system for cultivated and environmental viral genomes. Nucleic Acids Res..

[40] Berg M, Goudeau D, Olmsted C, McMahon KD, Thweatt J, Bryant D, et al. Host population diversity as a driver of viral infection cycle in wild populations of green sulfur bacteria with long standing virus-host interactions. BioRxiv. 2020. 10.1101/2020.03.05.979559. 10.1038/s41396-020-00870-1 PMC8163819 33452481

[41] Al-Shayeb B, Sachdeva R, Chen LX, Ward F, Munk P (2020). Devoto1 A, et al. Clades of huge phages from across Earth's ecosystems. Nature..

[42] PhyloPic. http://phylopic.org/ (2019). Accessed December, 2019.

[43] Ferry JG, Kastead KA, Cavicchioli R (2007). Methanogenesis. Archaea: molecular and cellular biology.

[44] Søndergaard M, Schierup HH (1982). Release of extracellular organic carbon during a diatom bloom in Lake Mossø: molecular weight fractionation. Freshw Biol..

[45] Bjørnsen PK (1988). Phytoplankton exudation of organic matter: Why do healthy cells do it?. Limnol Oceanogr..

[46] Conover RJ, Gustavson KR (1999). Sources of urea in arctic seas: zooplankton metabolism. Mar Ecol Prog Ser..

[47] Martinez A, Tyson GW, Delong EF (2010). Widespread known and novel phosphonate utilization pathways in marine bacteria revealed by functional screening and metagenomic analyses. Environ Microbiol..

[48] Allen AE, Dupont CL, Oborník M, Horák A, Nunes-Nesi A, McCrow JP (2011). Evolution and metabolic significance of the urea cycle in photosynthetic diatoms. Nature..

[49] Metcalf WW, Griffin BM, Cicchillo RM, Gao J, Janga SC, Cooke HA (2012). Synthesis of methylphosphonic acid by marine microbes: a source for methane in the aerobic ocean. Science..

[50] Mincer TJ, Aicher AC (2016). Methanol production by a broad phylogenetic array of marine phytoplankton. PLoS One..

[51] Heider J, Mai X, Adams MW (1996). Characterization of 2-ketoisovalerate ferredoxin oxidoreductase, a new and reversible coenzyme A-dependent enzyme involved in peptide fermentation by hyperthermophilic archaea. J Bacteriol..

[52] Rees GN, Harfoot CG, Sheehy AJ (1998). Amino acid degradation by the mesophilic sulfate-reducing bacterium *Desulfobacterium vacuolatum*. Arch Microbiol..

[53] Pelletier E, Kreimeyer A, Bocs S, Rouy Z, Gyapay G, Chouari R (2008). “*Candidatu*s Cloacamonas acidaminovorans”: genome sequence reconstruction provides a first glimpse of a new bacterial division. J Bacteriol..

[54] Geng H, Belas R (2010). Molecular mechanisms underlying roseobacter-phytoplankton symbioses. Curr Opin Biotechnol..

[55] Sun J, Todd JD, Thrash JC, Qian Y, Qian MC, Temperton B (2016). The abundant marine bacterium *Pelagibacter* simultaneously catabolizes dimethylsulfoniopropionate to the gases dimethyl sulfide and methanethiol. Nat Microbiol..

[56] Ticak T, Kountz DJ, Girosky KE, Krzycki JA, Ferguson DJ (2014). A nonpyrrolysine member of the widely distributed trimethylamine methyltransferase family is a glycine betaine methyltransferase. Proc Natl Acad Sci U S A..

[57] Cook AM, Daughton CG, Alexander M (1978). Phosphonate utilization by bacteria. J Bacteriol..

[58] Frederiksen TM, Finster K (2003). Sulfite-oxido-reductase is involved in the oxidation of sulfite in *Desulfocapsa sulfoexigens* during disproportionation of thiosulfate and elemental sulfur. Biodegradation..

[59] Frederiksen TM, Finster K (2004). The transformation of inorganic sulfur compounds and the assimilation of organic and inorganic carbon by the sulfur disproportionating bacterium *Desulfocapsa sulfoexigens*. Antonie van Leeuwenhoek..

[60] Finster KW, Kjeldsen KU, Kube M, Reinhardt R, Mussmann M, Amann R (2013). Complete genome sequence of *Desulfocapsa sulfexigens*, a marine deltaproteobacterium specialized in disproportionating inorganic sulfur compounds. Stand Genomic Sci..

[61] Marnocha CL, Levy AT, Powell DH, Hanson TE, Chan CS (2016). Mechanisms of extracellular S^0^ globule production and degradation in *Chlorobaculum tepidum* via dynamic cell–globule interactions. Microbiol..

[62] Thamdrup B, Finster K, Hansen JW, Bak F (1993). Bacterial disproportionation of elemental sulfur coupled to chemical reduction of iron or manganese. Appl Environ Microbiol..

[63] Gregersen LH, Bryant DA, Frigaard N (2011). Mechanisms and evolution of oxidative sulfur metabolism in green sulfur bacteria. Front Microbiol..

[64] Frigaard NU, Chew AG, Li H, Maresca JA, Bryant DA (2003). *Chlorobium tepidum*: insights into the structure, physiology, and metabolism of a green sulfur bacterium derived from the complete genome sequence. Photosynth Res..

[65] Berney M, Greening C, Conrad R, Jacobs WR, Cook GM (2014). An obligately aerobic soil bacterium activates fermentative hydrogen production to survive reductive stress during hypoxia. Proc Natl Acad Sci U S A..

[66] Krahn E, Schneider K, Muller A (1996). Comparative characterization of H_2_ production by the conventional Mo nitrogenase and the alternative “iron-only” nitrogenase of *Rhodobacter capsulatus hup*^*–*^ mutants. Appl Microbiol Biotechnol..

[67] Hocking WP, Stokke R, Roalkvam I, Steen IH (2014). Identification of key components in the energy metabolism of the hyperthermophilic sulfate-reducing archaeon *Archaeoglobus fulgidus* by transcriptome analyses. Front Microbiol..

[68] Sapra R, Bagramyan K, Adams MW (2003). A simple energy-conserving system: proton reduction coupled to proton translocation. Proc Natl Acad Sci U S A..

[69] Skennerton CT, Haroon MF, Briegel A, Shi J, Jensen GJ, Tyson GW (2016). Phylogenomic analysis of *Candidatus* 'Izimaplasma' species: free-living representatives from a Tenericutes clade found in methane seeps. ISME J..

[70] Greening C, Biswas A, Carere CR, Jackson CJ, Taylor MC, Stott MB (2016). Genomic and metagenomic surveys of hydrogenase distribution indicate H_2_ is a widely utilised energy source for microbial growth and survival. ISME J..

[71] Wilkins D, Yau S, Williams TJ, Allen MA, Brown MV, DeMaere MZ (2013). Key microbial drivers in Antarctic aquatic environments. FEMS Microbiol Rev..

[72] Karr EA, Sattley WM, Jung DO, Madigan MT, Achenbach LA (2003). Remarkable diversity of phototrophic purple bacteria in a permanently frozen Antarctic lake. Appl Environ Microbiol..

[73] Comeau AM, Harding T, Galand PE, Vincent WF, Lovejoy C (2012). Vertical distribution of microbial communities in a perennially stratified Arctic lake with saline, anoxic bottom waters. Sci Rep..

[74] Yau S, Lauro FM, DeMaere MZ, Brown MV, Thomas T, Raftery MJ (2011). Virophage control of Antarctic algal host–virus dynamics. PNAS..

[75] Tschitschko B, Williams TJ, Allen MA, Páez-Espino D, Kyrpides N, Zhong L (2015). Antarctic archaea–virus interactions: metaproteome-led analysis of invasion, evasion and adaptation. ISME J..

[76] DeMaere MZ, Williams TJ, Allen MA, Brown MV, Gibson JA, Rich J (2013). High level of intergenera gene exchange shapes the evolution of haloarchaea in an isolated Antarctic lake. PNAS..

[77] Tschitschko B, Erdmann S, DeMaere MZ, Roux S, Panwar P, Allen MA (2018). Genomic variation and biogeography of Antarctic haloarchaea. Microbiome..

[78] Timeanddate.com. https://www.timeanddate.com (1998). Accessed 15 Aug 2019.

[79] Huntemann M, Ivanova NN, Mavromatis K, Tripp HJ, Paez-Espino D, Tennessen K, et al. The standard operating procedure of the DOE-JGI Metagenome Annotation Pipeline (MAP v.4). Stand Genomic Sci. 2015;11:17. 10.1186/s40793-016-0138-x PMC4766715 26918089

[80] Li D, Luo R, Liu CM, Leung CM, Ting HF, Sadakane K, et al. MEGAHIT v1.0: A fast and scalable metagenome assembler driven by advanced methodologies and community practices. Methods. 2016;102:3–11. 10.1016/j.ymeth.2016.02.020 27012178

[81] Parks DH, Imelfort M, Skennerton CT, Hugenholtz P, Tyson GW (2015). CheckM: assessing the quality of microbial genomes recovered from isolates, single cells, and metagenomes. Genome Res..

[82] Parks DH, Rinke C, Chuvochina M, Chaumeil P, Woodcroft BJ, Evans PN (2017). Recovery of nearly 8,000 metagenome-assembled genomes substantially expands the tree of life. Nat Microbiol..

[83] CyVerse. https://de.cyverse.org (2018). Accessed on 11–19 Mar 2020.

[84] Roux S, Enault F, Hurwitz BL, Sullivan MB (2015). VirSorter: mining viral signal from microbial genomic data. PeerJ..

[85] Merchant N, Lyons E, Goff S, Vaughn M, Ware D, Micklos D (2016). The iPlant Collaborative: cyberinfrastructure for enabling data to discovery for the life sciences. PLOS Biol..

[86] Pritchard L, Glover RH, Humphris S, Elphinstone JG, Toth IK (2016). Genomics and taxonomy in diagnostics for food security: soft-rotting enterobacterial plant pathogens. Anal Methods..

[87] Allen MA, Lauro FM, Williams TJ, Burg D, Siddiqui KS, DeFrancisci D (2009). The genome sequence of the psychrophilic archaeon, *Methanococcoides burtonii*: the role of genome evolution in cold adaptation. ISME J..

[88] Søndergaard D, Pedersen CN, Greening C (2016). HydDB: a web tool for hydrogenase classification and analysis. Sci Rep..

[89] Edgar RC (2004). MUSCLE: multiple sequence alignment with high accuracy and high throughput. Nucleic Acids Res..

[90] Jones DT, Taylor WR, Thornton JM (1992). The rapid generation of mutation data matrices from protein sequences. Comput Appl Biosci..

[91] Tamura K, Stecher G, Peterson D, Filipski A, Kumar S. MEGA6: Molecular Evolutionary Genetics Analysis version 6.0. Mol Biol Evol. 2013;30:2725–2729. 10.1093/molbev/mst197 PMC3840312 24132122

[92] Paez-Espino D, Eloe-Fadrosh EA, Pavlopoulos GA, Thomas AD, Huntemann M, Mikhailova N (2016). Uncovering Earth's virome. Nature.

[93] Roux S, Adriaenssens EM, Dutilh BE, Koonin EV, Kropinski AM, Krupovic M (2019). Minimum Information about an Uncultivated Virus Genome (MIUViG). Nat Biotechnol..

[94] Paez-Espino D, Zhou J, Roux S, Nayfach S, Pavlopoulos GA, Schulz F (2019). Diversity, evolution, and classification of virophages uncovered through global metagenomics. Microbiome..

[95] Schulz F, Roux S, Paez-Espino D, Jungbluth S, Walsh DA, Denef VJ (2020). Giant virus diversity and host interactions through global metagenomics. Nature..

[96] Seemann T (2014). Prokka: rapid prokaryotic genome annotation. Bioinformatics..

[97] Marçais G, Delcher AL, Phillippy AM, Coston R, Salzberg SL, Zimin A (2018). MUMmer4: A fast and versatile genome alignment system. PLoS Comput Biol..

[98] Bin Jang H, Bolduc B, Zablocki O, Kuhn JH, Roux S, Adriaenssens EM (2019). Taxonomic assignment of uncultivated prokaryotic virus genomes is enabled by gene-sharing networks. Nature Biotechnol..

[99] VOGDB. http://vogdb.org/ (2020). VOG97 accessed January, 2020.

[100] Darling AC, Mau B, Blattner FR, Perna NT (2004). Mauve: multiple alignment of conserved genomic sequence with rearrangements. Genome Res..

