# Supplementary material for: Influence of the polar light cycle on seasonal dynamics of an Antarctic lake microbial community
Source: Microbiome. 2020 Aug 9;8:116. doi: 10.1186/s40168-020-00889-8 (PMC7416419; doi:10.1186/s40168-020-00889-8)
Supplement: Supplementary file 2 — Additional file 1: Ace Lake carbon cycle, expedition information, and taxonomic and functional analyses. Supplementary text, figures, tables and dataset. [file 40168_2020_889_MOESM1_ESM.zip › 40168_2020_889_MOESM1_ESM.pdf]

Additional file 1

Influence of the polar light cycle on seasonal dynamics of an Antarctic lake microbial community.

Pratibha Panwar, Michelle A. Allen, Timothy J. Williams, Alyce M. Hancock, Sarah Brazendale, James Bevington, Simon Roux, David Paez-Espino, Stephen Nayfach, Maureen Berg, Frederik Schulz, Amy Chen, Marcel Huntemann, Nicole Shapiro, Nikos Kyrpides, Tanja Woyke, Emiley A. Eloe-Fadrosh and Ricardo Cavicchioli

Additional file 1: Ace Lake carbon cycle, expedition information, and taxonomic and functional analyses

Supplementary text

Carbon cycling

Supplementary Figures

Fig. S1 Ace Lake 2013-2015 expedition.
Fig. S2 Seasonal influence on the peak relative abundance of major taxa in Ace Lake.
Fig. S3 Annual daylength at Davis Station, Vestfold Hills, Antarctica.
Fig. S4 Recorded sunlight hours at Davis Station, Vestfold Hills, Antarctica.
Fig. S5 Recorded wind velocity at Davis Station, Vestfold Hills, Antarctica.
Fig. S6 Recorded maximum daily temperature at Davis Station, Vestfold Hills, Antarctica.
Fig. S7 Recorded minimum daily temperature at Davis Station, Vestfold Hills, Antarctica.
Fig. S8 Temperature, salinity and dissolved oxygen profiles recorded for Ace Lake.

Supplementary Tables

Table S1 Ace Lake metagenomes used in this study.
Table S2 MetBAT MAGs of 51 OTUs from Ace Lake.
Table S3 SIMPER analysis showing similarity within each season group and the OTUs contributing to the similarity.
Table S4 SIMPER analysis showing dissimilarity between season groups and the OTUs contributing to the dissimilarity.
Table S5 Metabolic traits of the abundant OTUs identified in Ace Lake.
Table S6 Glycoside hydrolases and other glycoconjugate degradation enzymes from the abundant OTUs in Ace Lake.
Table S7 Hydrogenases of the abundant OTUs in Ace Lake.
Table S8 Abundant OTUs in Ace Lake.
Table S9 Environmental data for Ace lake samples.
Table S10 Pathways and enzymes analysed.
Table S11 KEGG numbers used to calculate abundance of specific pathways and enzymes.

Dataset

Dataset S1 See accompanying dataset (excel file): OTU relative abundances.

References

Carbon cycling

Overall, Bacteroidetes appears to have preferences for complex organic compounds, especially polysaccharides and glycoconjugates, which is consistent with growth assessments of isolates [1-3]. Balneolaceae UBA2664*,* detected in both U3 and the interface, appears to be a facultative anaerobe. The Verrucomicrobia also appear to be orientated toward using biopolymers, including sulfated polysaccharides (e.g., fucoidan, heparan sulfate) [4-6]. The Planctomycetes OTU (*Gimesia*) also encodes enzymes for sulfated polysaccharide degradation (e.g. fucoidan, carrageenans) [6]. Aerobic heterotrophic Gammaproteobacteria (*Pseudomonas* E, *Halioglobus,* Porticoccaceae HTCC2207) were inferred to be capable of degrading a broad range of substrates, including some biopolymers. *Pseudomonas* E was only observed in the Aug 2014 winter sample (20%) at a time when *Halioglobus* exhibited its peak relative abundance (6%) (Additional file 1: Dataset S1). Alphaproteobacteria, Betaproteobacteria, and Actinobacteria OTUs from the upper zone appeared to be heterotrophs that prefer organic solutes, including those released by algae and biopolymer-degrading heterotrophs. The Alphaproteobacteria *Loktanella* and *Yoonia* belong to the *Roseobacter* group ('roseobacters') within Rhodobacteraceae, which are often associated with phytoplankton and macroalgae [7-8]; *Loktanella* species have also been isolated from Ace Lake microbial mats [9]. The *Pelagibacter* complex appears capable of assimilating low-molecular-weight organic substrates, especially organic acids and sulfonates [10], including taurine, dimethylsulfoniopropionate (DMSP), and 2,3-dihydroxypropane-1-sulfonate (DHPS). In the anoxic layers (including the interface), anaerobic heterotrophs were dominated by Deltaproteobacteria, including sulfate respirers (*Desulfobacterium*, Desulfobacterales S5133MH16, Desulfatiglanales NaphS2) and sulfur disproportionators (*Desulfocapsa*), both of which appear capable of autotrophic growth. Other OTUs in the anoxic layers appear to have fermentation-based metabolisms, including Syntrophales UBA2210, Bacteroidales UBA4459, Atribacteria 34-128, Cloacimonetes JGIOTU-2, and *Izimaplasma* (Tenericutes). Among these, Bacteroidales UBA4459 and Cloacimonetes JGIOTU-2 also exhibit genomic capacities for biopolymer degradation. The Euryarchaeota identified were exclusively methanogens (*Methanothrix* A and Methanomicrobiaceae 1), which were present at depths (L2 and L3) where methane levels are high [11].

Ace Lake OTUs encode a diverse array of glycoside hydrolases (GHs) and other enzymes implicated in the degradation of complex carbohydrates and glycoconjugates. Among the 45 Ace Lake OTUs, 147 proteins were identified representing 30 GH families (Carbohydrate-Active Enzyme [CAZy] database [12-13]) (Table S11). As algal storage products, starch and laminarin are released from lysed cells and hydrolyzed by α-amylases (GH13/GH57) and β-glucanases (GH16/GH17/GH64), respectively. Within Ace Lake, these enzymes are encoded predominantly by Bacteroidetes and Verrucomicrobia, although oligo-1,6-glucosidases (GH13), for the hydrolysis of starch-derived oligosaccharides, are found in diverse OTUs. Chitin, produced by Ace Lake copepods, is hydrolyzed by chitinase (GH18/GH19), which is present in OTUs from the entire water column, representing Verrucomicrobia, Bacteroidetes, Deltaproteobacteria, and Cloacimonetes.

Celluloses and hemicelluloses may derive from moss and lichen blown into the lake from the surrounding Vestfold Hills [14], and green and brown algae in the lake [11, 15]. Cellulases (endoglucanases; GH5) for endohydrolysis of exogenous cellulose are encoded in *Polaribacter* and Balneolaceae UBA2664; the former protein includes a cellulose-binding domain. However, for certain other OTUs, endoglucanases (GH8) appear to be part of a biosynthetic pathway synthesizing cellulose as a component of an extracellular matrix that produces an aggregative phenotype [16]. Thus, different Ace Lake bacteria have the potential to synthesize and degrade cellulose. Cellobiose, released from cellulose degradation, can be degraded further to simple sugars via cellobiose phosphorylase (GH36), encoded in

Bacteroidales UBA4459, or β-glucosidases, which are present in many Ace Lake OTUs. β-glucosidases (GH1/GH3) encoded by diverse Ace Lake bacteria release glucose by hydrolysis of β-D-glucosides, including cellulose degradation products. For hemicellulosic material, endo-1,4-β-xylanases (GH10/GH39/GH43) (encoded in multiple Verrucomicrobia and Bacteroidetes OTUs, as well as *Halioglobus*) and endo-1,4-β-glucanase (GH9) (Bacteroidales UBA4459) cleave xylan or xyloglucan backbones, whereas β-xylosidase (GH39) (Verrucomicrobia and Bacteroidetes OTUs) and α-xylosidase (GH31) (multiple Bacteroidetes, as well as *Halioglobus* and Burkholderiaceae SCGC-AAA027-K21) hydrolyze terminal xylose residues. These enzymes may act in concert with β-glucosidases to release glucose and xylose from hemicellulose. Mannans are another component of hemicellulose, and mannose-rich polymers are prevalent in diatom cell-walls [17]; and β-mannosidases (GH2) and mannan endo-1,4-β-mannosidases (GH5, GH26) are encoded in OTUs from across Verrucomicrobia, Bacteroidetes, Planctomycetes, and Gammaproteobacteria. Certain OTUs have the potential to hydrolyze structural constituents of pectin, including arabinogalactan using endo-1,4-β-galactanase (GH53) (*Polaribacter*, Cloacimonetes JGIOTU-2), and rhamnogalacturonan using α-L-rhamnosidase (multiple Verrucomicrobia and Bacteroidetes OTUs).

The sulfated polysaccharide fucoidan, a component of cell walls of Phaeophyceae (brown algae) [4], has the potential to be used as a carbon and sulfur source. Hydrolysis by α-L-fucosidase (fucoidanase) releases the sugar fucose. The secreted enzyme α-L-fucosidase (GH29) is encoded in several OTUs (especially from Verrucomicrobia and Bacteroidetes), and these and other OTUs encoded enzymes for fucose degradation, by either of two alternative pathways involving either phosphorylated or non-phosphorylated intermediates [18]; the former was more prevalent in Ace Lake. Fucoidan digestion is facilitated by secreted sulfatases, which remove the sulfate groups and render the glycosidic linkages more accessible to the action of α-L-fucosidase [4, 19]; these were encoded by Ace Lake Bacteroidetes, Verrucomicrobia, and Planctomycetes OTUs, and would contribute to sulfur mineralization in Ace Lake.

Phytoplankton release organic solutes that include glycolate, amino acids, peptides, simple sugars, and methanol [20-22]. Methanol may be utilized as a carbon and energy source by Ace Lake methylotrophs such as Methylophilaceae BACL14 which was abundant only in spring, possibly indicative of an association with the *Synechococcus* bloom.

In primary producing algae, the photorespiratory by-product glycolate is either excreted or metabolized further to glycolate by glycolate oxidase, and is regarded as a potentially important carbon source for heterotrophic bacteria in marine waters [23]. In heterotrophic bacteria, glyoxylate can be combined with acetyl-CoA to produce malate using malate synthase, which is found in most of the OTUs that possess glycolate oxidase. Alternatively, alanine-glyoxylate aminotransferase (AGT) can convert glyoxylate into glycine, with the concomitant deamination of alanine to pyruvate; a putative AGT is found in all OTUs that possess glycolate oxidase.

Amino acid and peptide primary transporters were found across the examined Ace Lake OTUs, although some differential abundances were apparent (Additional file 1: Dataset S1); general amino acid ABC transporters predominated in the upper, oxic zone, and ABC transporters for branched-chain amino acids (BCAA) and peptides were more prevalent in the lower, anoxic zone. The latter accords with peptide and amino acid oxidation (including fermentation) by phylogenetically diverse anaerobes (e.g., Bacteroidales UBA4459, sulfate-reducing Deltaproteobacteria, Cloacimonetes JGIOTU-2) [24-25]. BCAA may be favored over other amino acids by anaerobes because, in addition to being carbon and nitrogen sources, catabolism of the resulting branched-chain 2-oxoacids can be used for energy conservation, including substrate-level phosphorylation [26].

Urea is excreted by zooplankton, including copepods [27], as well as being a diatom metabolite [28]. For the examined Ace Lake OTUs, urea can be catabolized as a nitrogen source using urease (*Synechococcus, Loktanella, Yoonia*, *Nisaea*, *Pseudomonas* E, Burkholderiaceae MOLA814, Burkholderiaceae SCGC-AAA027-K21) or urea amidolyase (Verrucomicrobia BACL24, Verrucomicrobia Arctic95D-9, *Pseudomonas* E, Pseudohongiellaceae 2, *Halioglobus*). Urea transporters and urea catabolism genes were detected throughout the water column, although more prevalent in the upper zone due to their presence in *Synechococcus* and many aerobic Proteobacteria and Verrucomicrobia.

Taurine uptake and catabolism genes were more prevalent in the oxic layers. The organosulfonate taurine, a solute found in marine invertebrates, is a potential nitrogen source; the ability to acquire nitrogen via transamination of taurine [29] or the related solute hypotaurine [30] is encoded across many OTUs. Overall, the ability to use taurine as a nitrogen source are far more prevalent than the ability to use it as a sulfur source across Ace Lake OTUs (Additional file 1: Table S5).

Several OTUs exhibit the capacity to degrade the organosulfonate 2,3-dihydroxypropane-1-sulfonate, a derivative of algal sulfolipids, to pyruvate, and hence use as a carbon source (*Hydrogenobacter*, *Pelagibacter,* Burkholderiaceae MOLA814) [31].

The algal osmolyte and antioxidant DMSP can be cleaved to dimethylsulfide and acrylate (assimilated as a carbon source) (*Yoonia*); or assimilated as a carbon and sulfur source by the demethylation pathway (*Pelagibacter, Yoonia, Loktanella*) [32]. DMSP is a preferred source of reduced sulfur for roseobacters [7] and *Pelagibacter* [33].

Glycine betaine, another algal osmolyte [34], can be utilized as a source of glycine by Ace Lake OTUs (*Pelagibacter*, *Desulfobacterium*, *Nisaea*, *Loktanella*, *Yoonia*) with dimethylglycine and sarcosine as intermediates; this is initiated by demethylation catalyzed by either betaine-homocysteine methyltransferase (*Pelagibacter*) or glycine betaine methyltransferase (*Desulfobacterium*, Desulfobacterales S5133MH16, *Nisaea*, *Loktanella*, *Yoonia*, Porticoccaceae HTCC2207) [35-36]. For sulfate-reducing Deltaproteobacteria, the demethylation of glycine betaine also provides a source of reductant for anaerobic respiration (anaerobic methylotrophy) (*Desulfobacterium,* Desulfobacterales S5133MH16) [37]. Trimethylamine, a natural breakdown product of glycine betaine [38], can be degraded using trimethylamine methyltransferase. Glycine betaine methyltransferase and trimethylamine methyltransferase are homologous [37], and could not be distinguished by KEGG analysis; combined, the two methyltransferases were detected throughout the entire water column.

Phosphonates are ubiquitous in natural environments, with methylphosphonate (Mpn) and 2-aminoethylphosphonate (2-AEPn) being especially prevalent in marine systems [39-40]. The capacity to degrade phosphonates is exhibited by several Ace Lake OTUs, including Mpn (*Loktanella*, *Yoonia*, *Nisaea*, *Pseudomonas* E, Burkholderiaceae MOLA814, *Desulfocapsa*), with methane gas generated as a byproduct [41], and 2-AePn (*Gimesia, Nisaea*), which liberates ammonia as well as phosphate for assimilation [39]. Overall, phosphonate ABC transporters were more abundant than identifiable phosphonate catabolism genes in Ace Lake; this observation may be at least partly attributable to certain phosphonate transporters also being capable of phosphate uptake [42], and/or the diversity of available phosphonates (aside from Mpn and 2-AEPn) and potential phosphonate degradation pathways [43].

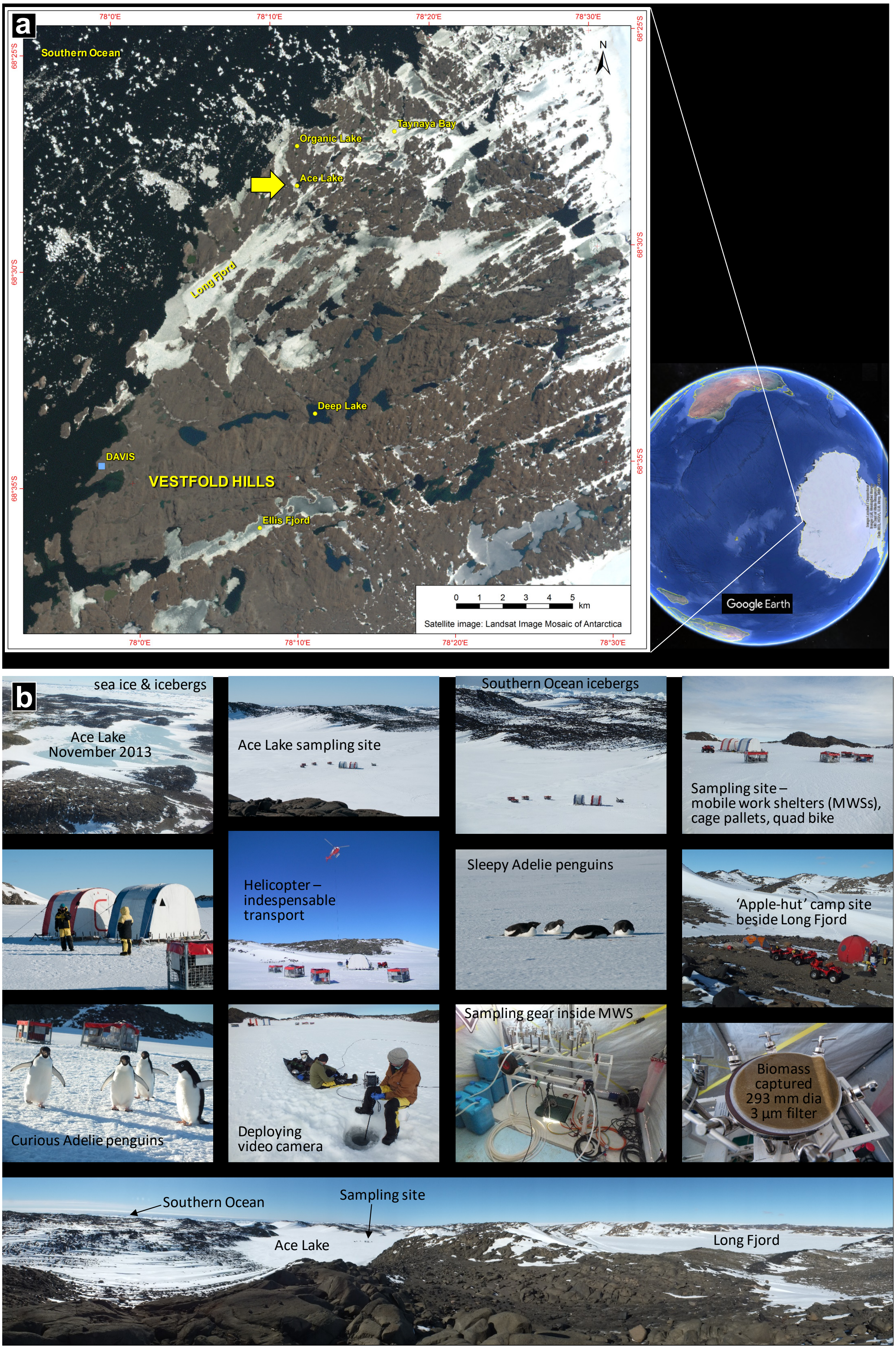
a
78°0'E
78°10'E
78°20'E
78°30'E
68°25'S
68°30'S
68°35'S
Southern Ocean
N
Taynaya Bay
Organic Lake
Ace Lake
Long Fjord
Deep Lake
DAVIS
VESTFOLD HILLS
Ellis Fjord
0 1 2 3 4 5 km
Satellite image: Landsat Image Mosaic of Antarctica
Google Earth
b
sea ice & icebergs
Ace Lake
November 2013
Ace Lake sampling site
Southern Ocean icebergs
Sampling site –
mobile work shelters (MWSs),
cage pallets, quad bike
Helicopter –
indespensable
transport
Sleepy Adelie penguins
'Apple-hut' camp site
beside Long Fjord
Curious Adelie penguins
Deploying
video camera
Sampling gear inside MWS
Biomass
captured
293 mm dia
3 μm filter
Southern Ocean
Sampling site
Ace Lake
Long Fjord

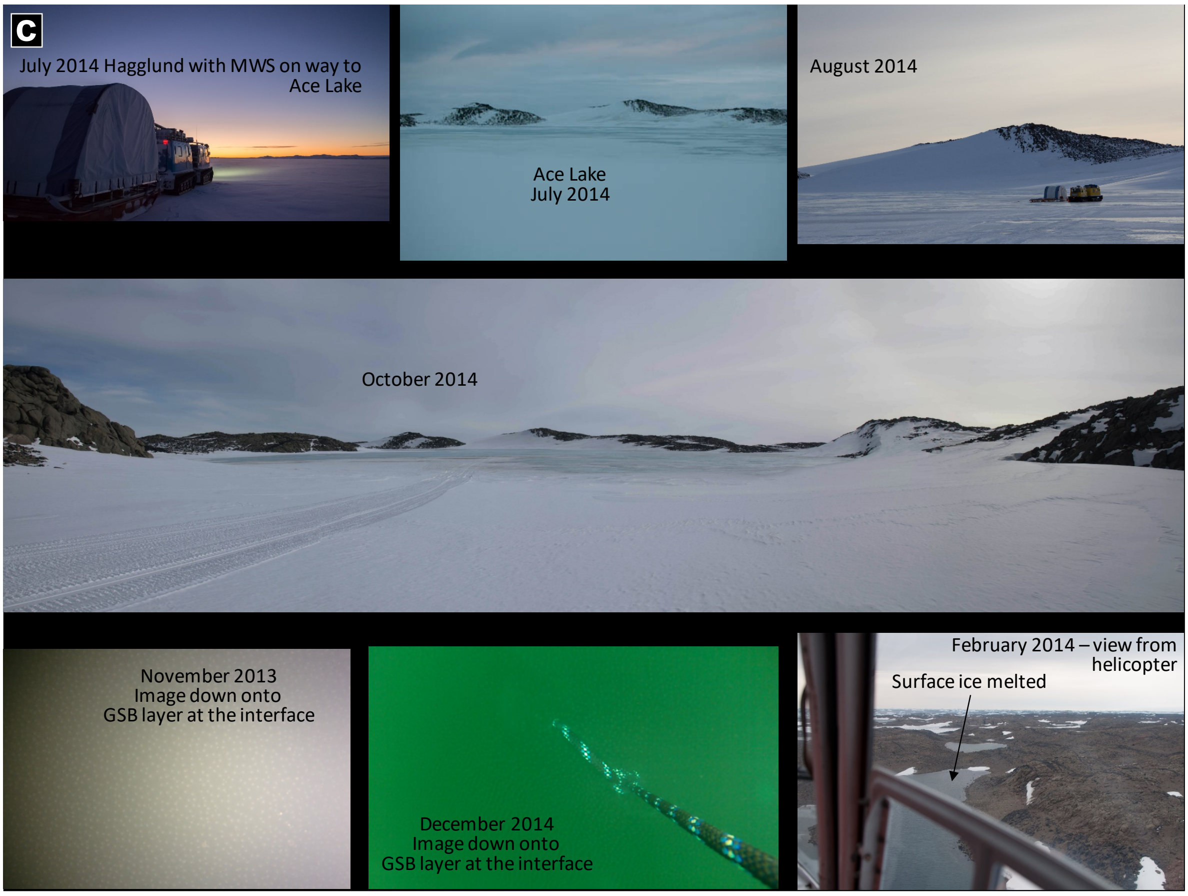

Fig. S1 Ace Lake 2013-2015 expedition. **(a)** View of the Earth showing Antarctica with an inset satellite image of the Vestfold Hills highlighting the location of Ace Lake (arrow). Credit to Google Earth (Image Landsat/Copernicus; Image U.S. Geological Survey; US Dept of State Geographer; Data SIO, NOAA, U.S. Navy, NGA, GEBCO); Credit to the Landsat Image Mosaic of Antarctica – the map was produced by the Australian Antarctic Data Centre. **(b, c)** Sampling expedition at Ace Lake 2013 – 2015. Photo credits: Sarah Payne, Alyce Hancock, Rob Isaac, John Foster and Rick Cavicchioli. See Additional file 1: Table S1 for a summary of the Ace Lake metagenomes used in this study, and Additional file 1: Table S9 for a summary of environmental data for Ace lake samples.

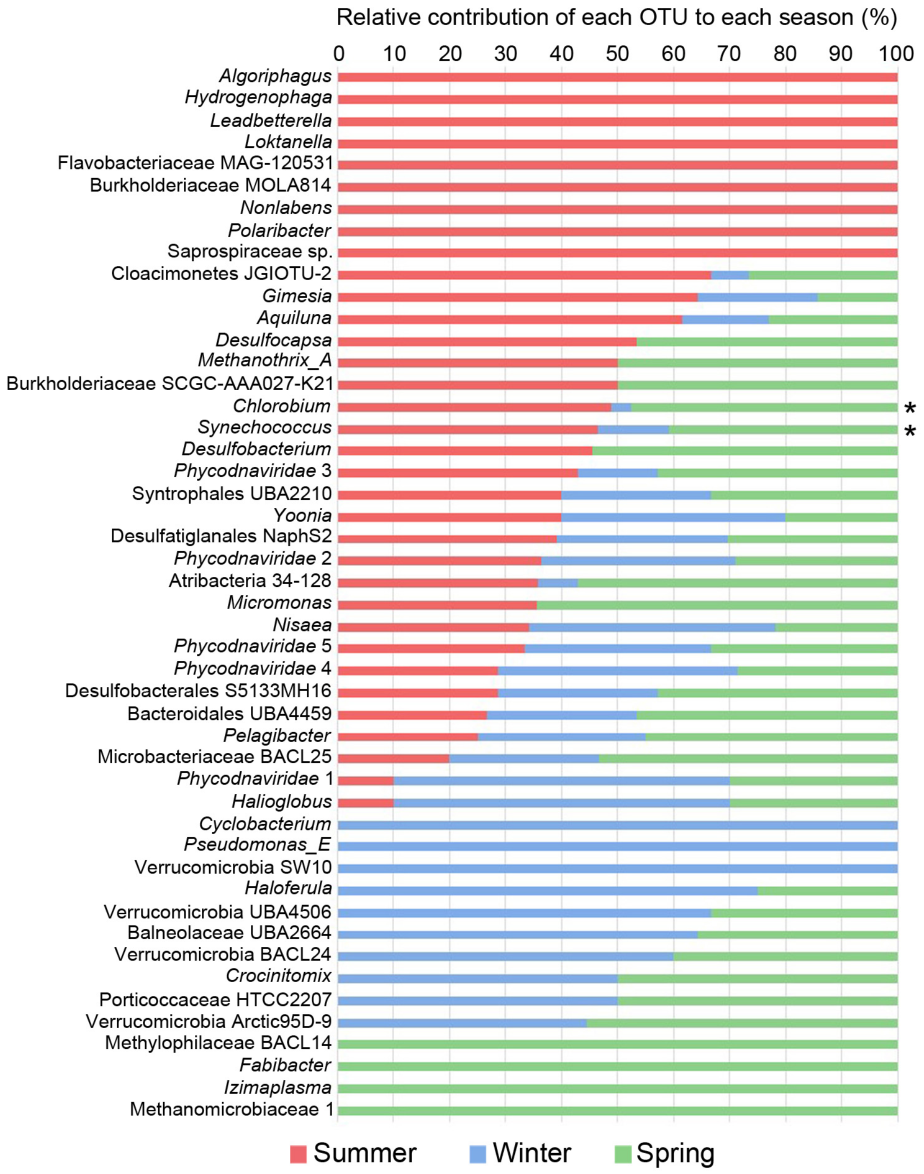

Fig. S2 Seasonal influence on the peak relative abundance of major taxa in Ace Lake. The 100% stacked bar graph depicts the relative contribution of abundant OTUs to summer (red), winter (blue), and spring (green). Fig. 4 depicts the seasonal influence on the peak relative abundance of major taxa, whereas this plot highlights the relative contribution by season. That is, the data used to plot this graph were peak relative abundances of the OTUs (as for Fig 4), but Fig. 4 is a stacked-bar chart that specifically shows the peak relative abundances of the OTUs in each season and gives a direct measure of the abundances in each season based on the length and color of the bars. However, as a 100% stacked-bar chart here, the input peak relative abundances are recalculated to 100% for all OTUs showing them as equivalently lengthed bars, with each showing the seasonal relative abundance of the OTU. For example, the top nine entries show 100% red bars indicating they were only detected as abundant OTUs (those with >1% relative abundance) in summer, whereas the relative contribution of *Chlorobium* and Burkholderiaceae SCGC-AAA027-K21 to summer is ~50%. However, note that the peak relative abundance of *Chlorobium* in summer is 83% whereas it is 1% for Burkholderiaceae SCGC-AAA027-K21 (Additional file 1: Dataset S1). * Due to their relevance in this study, *Chlorobium* and *Synechococcus* are highlighted.

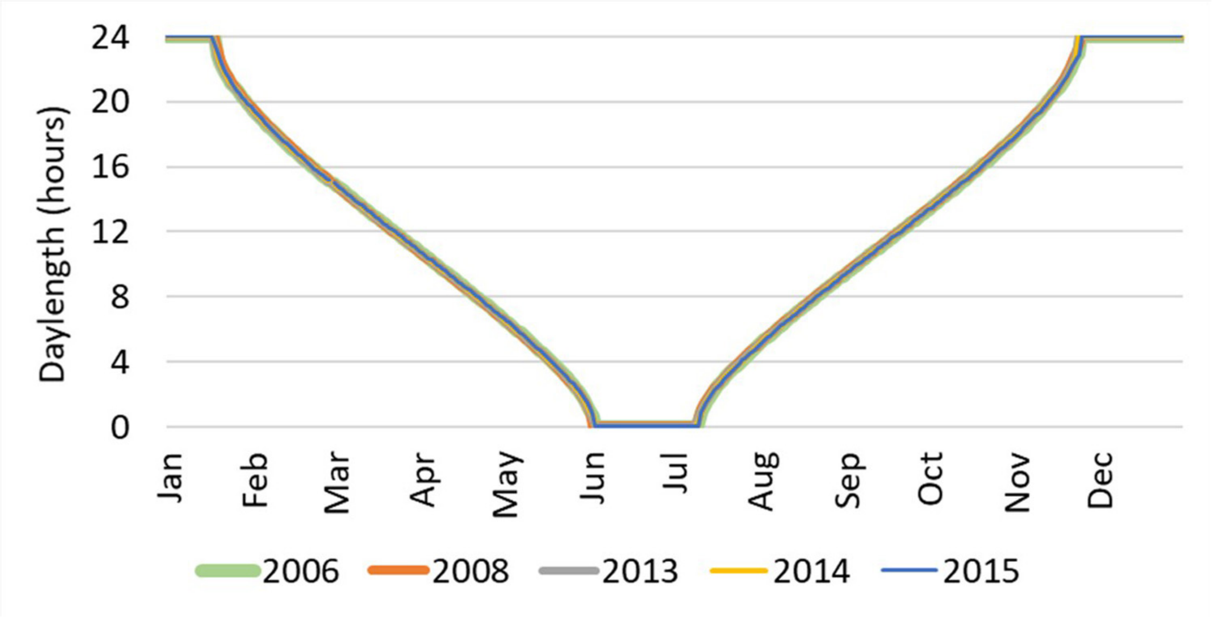

Fig. S3 Annual daylength at Davis Station, Vestfold Hills, Antarctica. Line graph depicting the daylength (number of hours the sun is above the horizon) at Davis Station in 2006 (green), 2008 (orange), 2013 (grey), 2014 (yellow) and 2015 (blue). Credit: timeanddate.com [44].

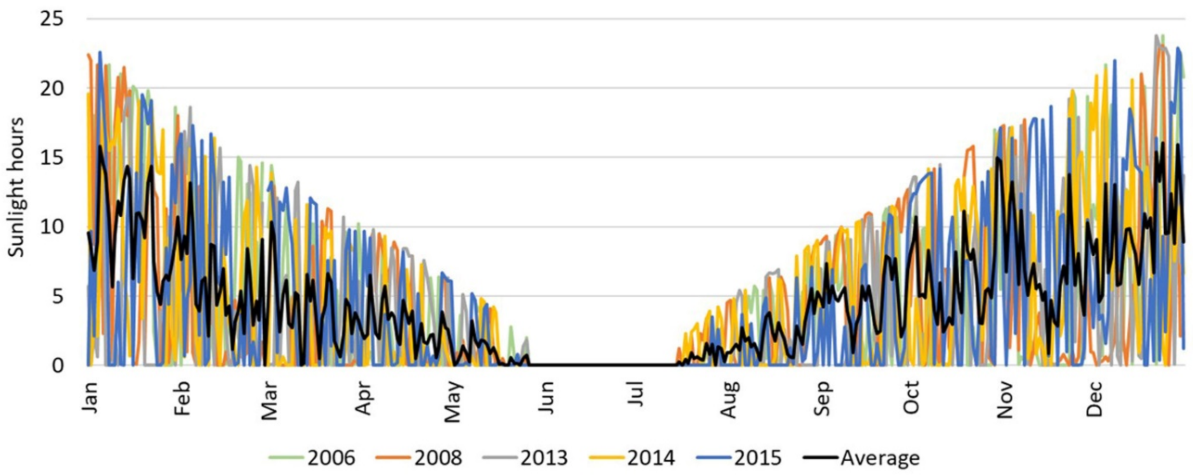

Fig. S4 Recorded sunlight hours at Davis Station, Vestfold Hills, Antarctica. Line graph depicting the daily sunlight hours (hours of bright sunshine without cloud cover) measured at Davis Station in 2006 (green), 2008 (orange), 2013 (grey), 2014 (yellow) and 2015 (blue), and the monthly average sunlight hours from all five years (black). Data from the Australian Antarctic Data Centre.

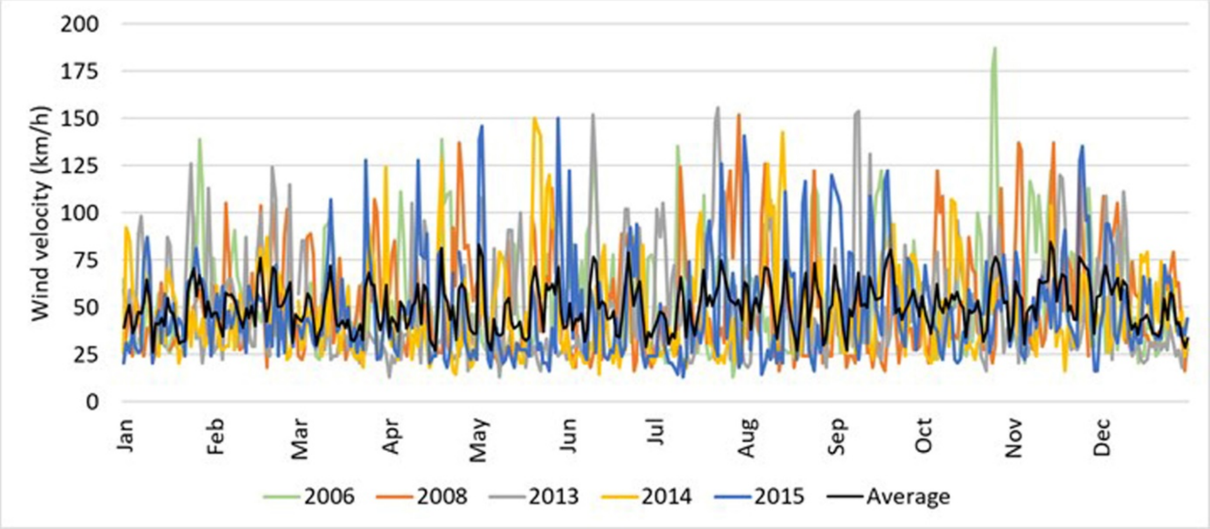

Fig. S5 Recorded wind velocity at Davis Station, Vestfold Hills, Antarctica. Line graph depicting the daily wind velocity measured at Davis Station in 2006 (green), 2008 (orange), 2013 (grey), 2014 (yellow) and 2015 (blue), and the monthly average wind velocity from all five years (black). Data from the Australian Antarctic Data Centre.

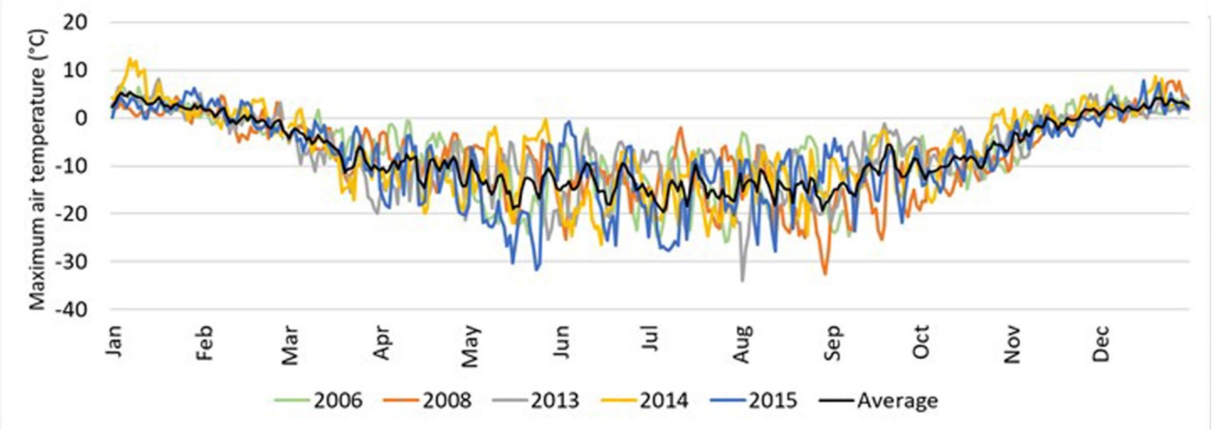

Fig. S6 Recorded maximum daily temperature at Davis Station, Vestfold Hills, Antarctica. Line graph depicting the daily maximum temperature measured at Davis Station in 2006 (green), 2008 (orange), 2013 (grey), 2014 (yellow) and 2015 (blue), and the monthly average maximum temperature calculated from all five years (black). Data from the Australian Antarctic Data Centre.

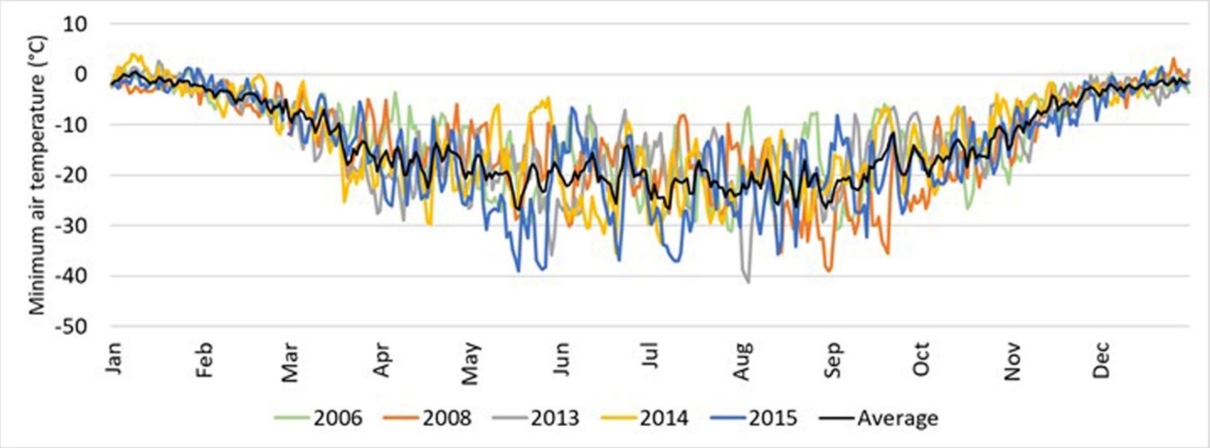

Fig. S7 Recorded minimum daily temperature at Davis Station, Vestfold Hills, Antarctica. Line graph depicting the daily minimum temperature measured at Davis Station in 2006 (green), 2008 (orange), 2013 (grey), 2014 (yellow) and 2015 (blue), and the monthly average minimum temperature calculated from all five years (black). Data from the Australian Antarctic Data Centre.

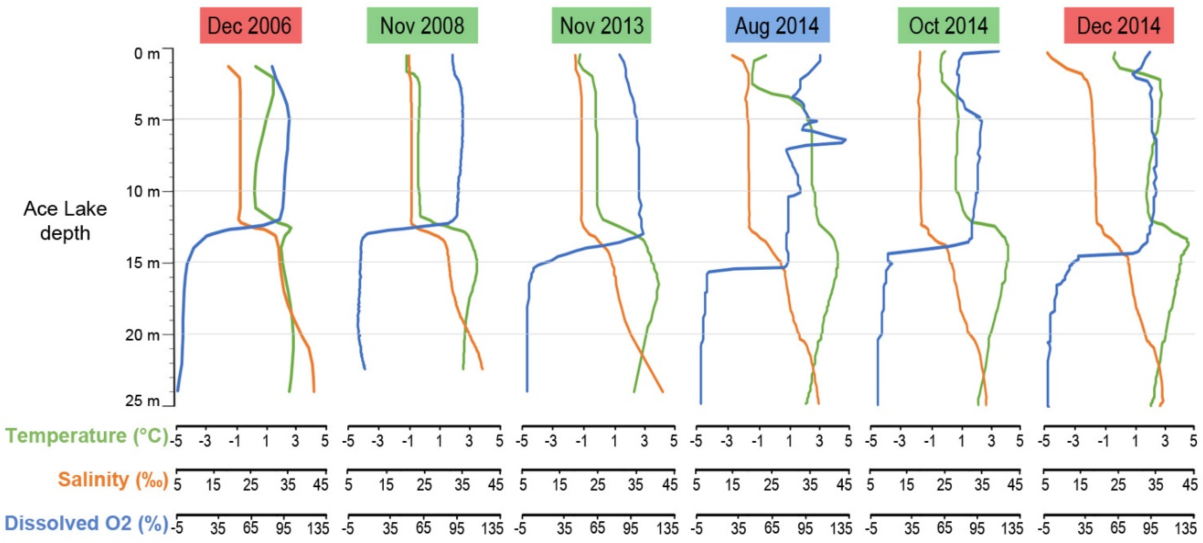

Fig. S8 Temperature, salinity and dissolved oxygen profiles recorded for Ace Lake. Scatter plots depicting depth profiles for lake temperature (green), salinity (orange) and dissolved oxygen content (blue) from Ace Lake between 2006 and 2014. Background color of the date labels denote the season: summer (red), winter (blue), and spring (green). The x-axis is separate for each parameter. The dissolved oxygen content was normalized to account for differences between data collection devices (YSI Sonde: 2006, 2008; TOA WQC: 2013-2014).

Table S1 Ace Lake metagenomes used in this study.

Season	Sample collection date	Ace Lake zone	Depth	‡ Filter fraction	IMG genome ID	Gold analysis project ID	Metagenome filtered reads (bp)	Assembled metagenome size (bp)	Total protein-coding genes
Summer	20 Dec 2006	Upper 2	5 m	3	3300028202	Ga0302065	65944407	9717163	18015
	20 Dec 2006	Upper 2	5 m	0.8	3300028221	Ga0302064	188760566	27952213	53952
	20 Dec 2006	Upper 2	5 m	0.1	3300028228	Ga0302071	514425517	33518956	64687
	20 Dec 2006	Upper 3	11.5 m	3	3300028205	Ga0302063	152109562	22138314	39285
	20 Dec 2006	Upper 3	11.5 m	0.8	3300028289	Ga0302062	194556802	16906227	32171
	20 Dec 2006	Upper 3	11.5 m	0.1	3300028222	Ga0302066	501692433	29126306	60086
	20 Dec 2006	Interface	12.7 m	3	3300028203	Ga0302061	83214739	10703483	20757
	20 Dec 2006	Interface	12.7 m	0.8	3300028201	Ga0302060	208538507	11925309	23740
	20 Dec 2006	Interface	12.7 m	0.1	3300028204	Ga0302067	240290391	6971450	13087
	20 Dec 2006	Lower 1	14 m	3	3300028200	Ga0302059	118655678	15468656	31907
	20 Dec 2006	Lower 1	14 m	0.8	3300028302	Ga0302058	165208287	27504336	56468
	20 Dec 2006	Lower 1	14 m	0.1	3300028219	Ga0302068	169703894	23317396	54216
	20 Dec 2006	Lower 2	18 m	3	3300028199	Ga0302057	114460928	12486049	26210
	20 Dec 2006	Lower 2	18 m	0.8	3300028227	Ga0302056	214177665	34270862	71009
	20 Dec 2006	Lower 2	18 m	0.1	3300028216	Ga0302069	145906502	15860072	40100
	20 Dec 2006	Lower 3	23 m	3	3300028292	Ga0302054	105388116	11819279	24794
	20 Dec 2006	Lower 3	23 m	0.8	3300028226	Ga0302055	231162768	33899871	71413
	20 Dec 2006	Lower 3	23 m	0.1	3300028296	Ga0302070	292220289	26024886	62208
Spring	19 Nov 2008	Upper 2	5 m	3	3300025601	Ga0208768	10168447444	374845559	637417
	19 Nov 2008	Upper 2	5 m	0.8	3300025513	Ga0208413	8608322293	358461005	555436
	19 Nov 2008	Upper 2	5 m	0.1	3300025425	Ga0208646	9326252194	190824688	354920
	21 Nov 2008	Upper 3	11.8 m	3	3300025502	Ga0208903	9958328840	309922874	529432
	21 Nov 2008	Upper 3	11.8 m	0.8	3300025603	Ga0208414	10372524015	387727814	649215
	21 Nov 2008	Upper 3	11.8 m	0.1	3300025438	Ga0208770	8652779583	208281887	381283
	21 Nov 2008	Interface	12.8 m	3	3300025433	Ga0208900	7377945147	191332554	330516
	21 Nov 2008	Interface	12.8 m	0.8	3300025380	Ga0208901	7969400898	118925863	224047
	21 Nov 2008	Interface	12.8 m	0.1	3300025362	Ga0208647	15030492867	90472821	190960
	21 Nov 2008	Lower 1	14.1 m	3	3300025649	Ga0208279	8878877148	403510882	775430
	21 Nov 2008	Lower 1	14.1 m	0.8	3300025628	Ga0208902	9024438900	379168081	728210
	21 Nov 2008	Lower 1	14.1 m	0.1	3300025697	Ga0208769	7433358222	401517242	923143
	21 Nov 2008	Lower 2	18 m	3	3300025642	Ga0208648	9701518914	444311389	775322
	21 Nov 2008	Lower 2	18 m	0.8	3300025586	Ga0207996	10550636481	338938472	589716
	21 Nov 2008	Lower 2	18 m	0.1	3300025669	Ga0208904	8489799212	415535816	832930

	23 Nov 2008	Lower 3	23 m	3	3300025698	Ga0208771	8926498848	428043704	894948
	23 Nov 2008	Lower 3	23 m	0.8	3300025661	Ga0208905	8835913368	414688901	822281
	23 Nov 2008	Lower 3	23 m	0.1	3300025736	Ga0207997	8391237271	477169979	1113701
Spring	24 Nov 2013	Upper 2	5 m	3	3300022867	Ga0222629	4225013370	144719058	289211
	24 Nov 2013	Upper 2	5 m	0.8	3300023243	Ga0222630	4462325958	205826389	369592
	24 Nov 2013	Upper 2	5 m	0.1	3300022843	Ga0222631	3805948564	100883143	212850
	25 Nov 2013	Upper 3	12.5 m	3	3300022842	Ga0222632	4534814707	163226887	302245
	25 Nov 2013	Upper 3	12.5 m	0.8	3300022847	Ga0222633	4208778962	155718155	244054
	25 Nov 2013	Upper 3	12.5 m	0.1	3300023235	Ga0222634	4703733094	143622133	282929
	26 Nov 2013	Interface	13.5 m	3	3300022882	Ga0222626	4632992773	197528912	370963
	26 Nov 2013	Interface	13.5 m	0.8	3300023244	Ga0222627	4017414066	152968368	281280
	26 Nov 2013	Interface	13.5 m	0.1	3300022871	Ga0222628	4289343500	153918125	304781
	26 Nov 2013	Lower 1	15 m	3	3300023234	Ga0222635	2830397582	132062988	251704
	26 Nov 2013	Lower 1	15 m	0.8	3300022854	Ga0222636	4179971653	189382169	349194
	26 Nov 2013	Lower 1	15 m	0.1	3300023435	Ga0222637	3982384098	204889614	458784
	26 Nov 2013	Lower 2	19 m	3	3300023298	Ga0222638	3861886442	173692067	351338
	26 Nov 2013	Lower 2	19 m	0.8	3300023262	Ga0222639	5356530473	256708329	493455
	26 Nov 2013	Lower 2	19 m	0.1	3300023297	Ga0222640	4526133618	236042504	568485
	27 Nov 2013	Lower 3	24 m	3	3300022828	Ga0222641	2032322733	65695823	149469
	27 Nov 2013	Lower 3	24 m	0.8	3300022887	Ga0222642	4489480975	197136157	423504
	27 Nov 2013	Lower 3	24 m	0.1	3300031227	Ga0307928	21163513792	1050144399	2413590
Summer	17 Dec 2014	Upper 1	Surface	3	3300022841	Ga0222644	3505709238	109878484	205134
	17 Dec 2014	Upper 1	Surface	0.8	3300022833	Ga0222645	3007301388	112095376	172874
	17 Dec 2014	Upper 1	Surface	0.1	3300022822	Ga0222646	3926440146	72848168	141301
Summer	15 Feb 2014	Upper 1	Surface	3	3300022827	Ga0222647	4445471441	150261289	262344
	15 Feb 2014	Upper 1	Surface	0.8	3300023054	Ga0222648	4101153533	186668359	262345
	15 Feb 2014	Upper 1	Surface	0.1	3300022839	Ga0222649	4105154760	94401441	195630
Winter	2 Jul 2014	Upper 2	5 m	3	3300023237	Ga0222650	4712346032	179194270	291313
	2 Jul 2014	Upper 2	5 m	0.8	3300022866	Ga0222651	4450973256	227490836	403969
	2 Jul 2014	Upper 2	5 m	0.1	3300022853	Ga0222652	4388723345	128153264	250568
	3 Jul 2014	Upper 3	12.5 m	3	3300022857	Ga0222653	3349508936	162523775	274815
	3 Jul 2014	Upper 3	12.5 m	0.8	3300022836	Ga0222654	3812123689	173061625	297508
	3 Jul 2014	Upper 3	12.5 m	0.1	3300023245	Ga0222655	4389831560	141659134	285227
	3 Jul 2014	Interface	13.5 m	3	3300022834	Ga0222656	3025335676	150334734	279053
	3 Jul 2014	Interface	13.5 m	0.8	3300023241	Ga0222657	3917460255	176108874	316827
	3 Jul 2014	Interface	13.5 m	0.1	3300023257	Ga0222658	4754144028	246566898	516984
Winter	20 Aug 2014	Upper 2	5 m	3	3300023236	Ga0222659	3535315349	145971573	260745

	20 Aug 2014	Upper 2	5 m	0.8	3300023239	Ga0222660	3675443392	161858999	300236
	20 Aug 2014	Upper 2	5 m	0.1	3300023229	Ga0222661	3581244138	112903843	219283
	21 Aug 2014	Upper 3	13 m	3	3300022885	Ga0222662	4805699185	232800896	422661
	21 Aug 2014	Upper 3	13 m	0.8	3300022845	Ga0222663	3046800658	127278965	240608
	21 Aug 2014	Upper 3	13 m	0.1	3300023296	Ga0222664	4126784684	163100043	305555
	21 Aug 2014	Interface	14.5 m	3	3300022864	Ga0222665	4208293249	203541480	379585
	21 Aug 2014	Interface	14.5 m	0.8	3300024048	Ga0222666	4438778032	185710747	327952
	21 Aug 2014	Interface	14.5 m	0.1	3300022890	Ga0222667	3761803592	196439047	427804
Spring	20 Oct 2014	Upper 2	5 m	3	3300022865	Ga0222668	3718130970	159691784	283171
	20 Oct 2014	Upper 2	5 m	0.8	3300022825	Ga0222669	3500964757	137992510	261144
	20 Oct 2014	Upper 2	5 m	0.1	3300023294	Ga0222670	4051255334	135330843	259473
	20 Oct 2014	Upper 3	12 m	3	3300022848	Ga0222674	3461486260	157234838	316382
	20 Oct 2014	Upper 3	12 m	0.8	3300023238	Ga0222675	3185298810	140908866	262229
	20 Oct 2014	Upper 3	12 m	0.1	3300023240	Ga0222676	3685976302	125847023	262910
	21 Oct 2014	Interface	13 m	3	3300022856	Ga0222671	3793702914	185885369	366842
	21 Oct 2014	Interface	13 m	0.8	3300022859	Ga0222672	3615901126	148572713	281988
	21 Oct 2014	Interface	13 m	0.1	3300022821	Ga0222673	3169765298	119795036	247086
	21 Oct 2014	Lower 1	16 m	3	3300022855	Ga0222677	2823639110	137224766	262841
	21 Oct 2014	Lower 1	16 m	0.8	3300023249	Ga0222678	3472734434	161447324	294441
	21 Oct 2014	Lower 1	16 m	0.1	3300022858	Ga0222679	3214387734	162887351	368840
	21 Oct 2014	Lower 2	19 m	3	3300023434	Ga0222680	3699374508	165008949	330503
	21 Oct 2014	Lower 2	19 m	0.8	3300022838	Ga0222681	3195707102	158062637	299108
	21 Oct 2014	Lower 2	19 m	0.1	3300023246	Ga0222682	3202188919	153939570	372354
	21 Oct 2014	Lower 3	24 m	3	3300023251	Ga0222683	3707575608	149036067	306831
	21 Oct 2014	Lower 3	24 m	0.8	3300023295	Ga0222684	4015996994	166137713	367296
	21 Oct 2014	Lower 3	24 m	0.1	3300022874	Ga0222685	3523521042	181923112	450383
Summer	4 Dec 2014	Upper 2	5 m	3	3300023501	Ga0222686	3558906481	126636802	250738
	4 Dec 2014	Upper 2	5 m	0.8	3300022844	Ga0222687	3528199602	163618968	306086
	4 Dec 2014	Upper 2	5 m	0.1	3300023293	Ga0222688	3287944538	81894154	178097
	4 Dec 2014	Upper 3	12 m	3	3300023231	Ga0222689	3372774996	116441688	240321
	4 Dec 2014	Upper 3	12 m	0.8	3300023227	Ga0222690	3766666990	103396553	207492
	4 Dec 2014	Upper 3	12 m	0.1	3300022851	Ga0222691	3582064538	119299278	248470
	4 Dec 2014	Interface	13.4 m	3	3300031697	Ga0307929	14149086706	400324806	718959
	4 Dec 2014	Interface	13.4 m	0.8	3300022826	Ga0222693	2989229242	78299135	145800
	4 Dec 2014	Interface	13.4 m	0.1	3300023292	Ga0222694	3878932484	85111111	181733
	4 Dec 2014	Lower 1	14 m	3	3300023253	Ga0222695	3420681173	167955693	307470
	4 Dec 2014	Lower 1	14 m	0.8	3300023233	Ga0222696	3250064514	144877168	252928

	4 Dec 2014	Lower 1	14 m	0.1	3300022868	Ga0222697	3895509417	195190896	414173
	3 Dec 2014	Lower 2	19 m	3	3300022860	Ga0222698	4079964767	181977179	369802
	3 Dec 2014	Lower 2	19 m	0.8	3300022846	Ga0222699	3983828178	165102958	309999
	3 Dec 2014	Lower 2	19 m	0.1	3300023061	Ga0222700	3209269596	152256002	384107
	3 Dec 2014	Lower 3	24 m	3	3300022884	Ga0222701	4021442672	179261304	381611
	3 Dec 2014	Lower 3	24 m	0.8	3300023299	Ga0222702	5006350890	217304898	440798
	3 Dec 2014	Lower 3	24 m	0.1	3300023256	Ga0222703	3621396862	179844837	445634
Summer	8 Jan 2015	Upper 1	Surface	3	3300022829	Ga0222706	3645848765	78301103	152629
	8 Jan 2015	Upper 1	Surface	0.8	3300022832	Ga0222707	3757499746	136667441	270106
	8 Jan 2015	Upper 1	Surface	0.1	3300023242	Ga0222708	3407544904	121628756	269881
Summer	27 Jan 2015	Upper 1	Surface	3	3300023230	Ga0222709	3829689694	116684467	219301
	27 Jan 2015	Upper 1	Surface	0.8	3300023429	Ga0222710	3298326784	165138532	262012
	27 Jan 2015	Upper 1	Surface	0.1	3300022837	Ga0222711	3616258196	93765159	194928

‡ Filter fractions: 3, 20–3 µm; 0.8, 3–0.8 µm; 0.1, 0.8–0.1 µm

Table S2 MetBAT MAGs of 51 OTUs from Ace Lake.

OTUs*	MAGs	GTDB taxonomy	Genome size (Mb)	Gene count	GC content (%)	Completeness (%)	Contamination (%)	Strain heterogeneity (%)
Aquiluna	Bin802	s__*Aquiluna* sp1	1	1114	51.6	71	2	100
	Bin842	s__*Aquiluna* sp1	0.5	601	51.4	39	0	0
	Bin1781	s__*Aquiluna* sp1	0.82	901	51.5	53	0	0
Microbacteriaceae BACL25	Bin1187	s__BACL25 sp1	1.08	1239	57.5	57	5	95
	Bin1172	s__BACL25 sp1	1.34	1419	57.4	87	3	79
	Bin1399	s__BACL25 sp1	1.02	1098	58.2	90	5	81
	Bin534	s__BACL25 sp1	0.69	785	57.9	60	4	67
Loktanella	Bin864	s__*Loktanella salsilacus*	3.35	3541	59.7	97	0.3	0
Nisaea	Bin1427	g__BAL199	6.2	5801	68.7	96	4	18
	Bin283	g__BAL199	4.39	4220	69	78	0	0
Pelagibacter	Bin1939	s__*Pelagibacter ubique*	1.34	1578	28.5	65	11	56
	Bin2016	s__*Pelagibacter ubique*	0.97	1196	29	72	12	59
	Bin1535	s__*Pelagibacter ubique*	1.14	1394	28.2	57	13	46
	Bin978	s__*Pelagibacter ubique*	0.82	966	28.6	65	5	85
	Bin1105	s__*Pelagibacter ubique*	0.7	852	28.5	57	3	71
	Bin1323	s__*Pelagibacter ubique*	1.08	1331	27.9	43	5	40
	Bin887	s__*Pelagibacter ubique*	0.49	605	30.9	14	5	67
	Bin2004	s__*Pelagibacter ubique*	0.35	457	30.8	14	1	100
	Bin1518	s__*Pelagibacter ubique*	0.37	444	27.4	33	0	0
	Bin363	s__*Pelagibacter*_A *ubique*	1.17	1255	30	98	0	0
	Bin1541	g__*Pelagibacter*	2.2	2597	28.1	31	8	20
	Bin1782	g__*Pelagibacter*	1.46	1947	27.6	30	8	30
	Bin1123	g__*Pelagibacter*	1.53	1647	25.8	13	4	100
	Bin1666	g__*Pelagibacter*	0.88	1128	26.3	15	2	0

	Bin1485	g__IMCC9063	1.11	1258	32.2	92	4	20
	Bin1036	g__IMCC9063	1.41	1608	32.9	95	5	0
Yoonia	Bin1729	s__*Loktanella vestfoldensis*	2.44	2588	61.3	88	3	57
Atribacteria 34-128	Bin894	g__34-128	0.73	739	33.6	52	0	0
	Bin1182	g__34-128	0.52	561	32.8	36	0.03	0
	Bin866	g__34-128	0.83	891	34.5	53	3	50
	Bin2083	g__34-128	0.62	679	32.9	22	0	0
Algoriphagus	Bin1943	g__*Algoriphagus*	4.36	4089	39.8	98	2	25
Leadbetterella	Bin277	g__*Leadbetterella*	4.46	3961	40.6	99	1	25
Saprospiraceae sp.	Bin420	f__Saprospiraceae	4.68	4049	35.4	83	2	0
Bacteroidales UBA4459	Bin1394	g__UBA4459	4.09	3374	33.7	95	4	36
Crocinitomix	Bin223	g__*Crocinitomix*	4.32	3912	37.7	99	1	50
Cyclobacterium	Bin1381	s__*Cyclobacterium qasimii*	6.29	5400	38.9	99.6	2	10
Fabibacter	Bin155	s__*Fabibacter* sp1	3.69	3395	40.2	98	2	22
Flavobacteriaceae MAG-120531	Bin1744	g__MAG-120531	2.14	1950	36.8	95	0.1	100
	Bin896	g__MAG-120531	1.96	1777	38.6	96	0.02	100
Nonlabens	Bin1375	g__*Nonlabens*	2.61	2555	40.7	91	2	10
	Bin690	s__*Nonlabens dokdonensis*	3.44	3052	36.1	97	0	0
Polaribacter	Bin1415	g__*Polaribacter*	4.13	4437	33.7	77	52	79
	Bin385	g__*Polaribacter*	4.08	3856	31.5	92	3	33
	Bin670	g__*Polaribacter*	1.9	1983	30.2	37	2	100
	Bin246	g__*Polaribacter*	2.64	2899	32.6	62	5	0
	Bin574	g__*Polaribacter*	3.03	2740	30.7	97	3	29
	Bin776	g__*Polaribacter*	1.01	1278	33.6	14	0	0
Balneolaceae UBA2664	Bin306	g__UBA2664	3.16	2873	40.3	93	0.8	50
Burkholderiaceae MOLA814	Bin1173	g__RS62	2.43	2466	54.2	97	1	50
Burkholderiaceae SCGC-	Bin1507	g__SCGC-AAA027-K21	2.41	2415	53.5	90	2	62

AAA027-K21								
Hydrogenophaga	Bin22	g__*Hydrogenophaga*	4.38	4214	63.3	99	1	0
Methylophilaceae BACL14	Bin470	s__BACL14 sp1	1.17	1250	38	95	0.2	100
Chlorobium	Bin1268	s__*Chlorobium phaeovibrioides*	1.84	1746	52.3	99.5	0.6	0
*Micromonas**	Bin919	unclassified	22	21579	59.4	75.4	47.58	0
Cloacimonetes JGIOTU-2	Bin1264	s__JGIOTU-2 sp1	0.9	972	39.1	39	0.1	100
	Bin1703	s__JGIOTU-2 sp1	1.87	1862	38.1	91	1	100
	Bin1683	s__JGIOTU-2 sp1	1.04	1084	38.6	65	1	67
Synechococcus	Bin1724	g__*Cyanobium*	2.47	2643	64.2	95	0.1	100
Desulfatiglanales NaphS2	Bin2047	g__NaphS2	5.56	5490	49.7	88	3	67
	Bin505	g__NaphS2	2.12	2267	48	51	5	80
	Bin1224	g__NaphS2	3.45	3663	50.2	65	1	0
Desulfobacterales S5133MH16	Bin1209	g__S5133MH16	3.54	3575	43.5	76	4	75
	Bin1110	g__S5133MH16	2.05	1929	39.5	86	0.5	0
	Bin1728	g__S5133MH16	3.65	3718	43	82	2	0
Desulfobacterium	Bin703	g__*Desulfobacterium*	6.23	5861	45.1	70	12	85
	Bin1072	g__*Desulfobacterium*	4.1	4015	46	54	5	50
Desulfocapsa	Bin20	s__*Desulfocapsa sulfexigens*	4.27	3835	44.2	96	3	17
	Bin2043	s__*Desulfocapsa sulfexigens*	4.04	3664	44.1	99	2	0
	Bin134	s__*Desulfocapsa sulfexigens*	4.52	4198	44.6	99	3	0
Syntrophales UBA2210	Bin962	s__UBA2210 sp1	1.37	1474	47	71	4	58
	Bin2060	g__UBA2210	1.99	2015	47.7	82	3	0
Methanomicrobiaceae 1	Bin1205	f__Methanomicrobiaceae	1.08	1261	48.1	52	2	75
Methanothrix_A	Bin23	g__*Methanothrix*_A	2.38	2450	49.3	97	0	0
Halioglobus	Bin1377	g__*Halioglobus*	3.98	3737	54.8	95	1	50
Porticoccaceae HTCC2207	Bin525	g__HTCC2207	2.27	2203	46	95	1	75
	Bin686	g__HTCC2207	2.68	2598	48.9	97	0	0

	Bin271	g__HTCC2207	2.73	2558	50.4	100	9	21
Pseudohongiellaceae 1	Bin706	g__OM182	3.44	3528	46.2	70	6	70
Pseudohongiellaceae 2	Bin2107	s__OM182 sp1	2.82	2461	52.6	95	0.1	0
Pseudomonas_E	Bin911	s__*Pseudomonas*_E *alcaliphila*	4.98	4621	62.6	99.5	2	9
Phycodnaviridae 1, 2, 4, 5*	Bin62	p__Proteobacteria	7.98	10891	46.3	47	24	2
Phycodnaviridae 3*	Bin1350	d__Bacteria	0.2	230	33.5	3	0.4	0
	Bin1042	d__Bacteria	0.22	278	35.4	3	0.4	0
	Bin1755	d__Bacteria	0.45	472	40.7	5	0.5	0
	Bin97	d__Bacteria	0.44	432	30.2	6	0.5	0
	Bin1551	d__Bacteria	0.52	513	36.1	5	0.5	0
	Bin1998	d__Bacteria	0.37	364	27.1	4	0.5	0
	Bin784	d__Bacteria	0.26	319	29.4	3	0.5	0
	Bin2102	d__Bacteria	0.45	470	32.4	6	0.5	0
	Bin494	unclassified	0.32	373	31.9	4	0	0
	Bin651	unclassified	0.34	387	31.5	5	0.6	0
	Bin932	unclassified	0.3	370	29	4	0.6	11.11
	Bin1852	unclassified	0.51	602	31.8	6	0.4	0
Oligoflexus	Bin927	s__*Oligoflexus tunisiensis*	4.51	4186	41.8	93	2	0
	Bin255	s__*Oligoflexus tunisiensis*	3.65	3465	41.7	88	0	0
Gimesia	Bin1542	s__*Gimesia maris*	7.14	5613	48.8	99	1	0
	Bin1604	s__*Gimesia maris*	7.49	5969	46.3	96	1	0
Izimaplasma	Bin1380	g__*Izimaplasma*	1.56	1592	30.7	97	3	50
Haloferula	Bin1608	g__*Haloferula*	4	3763	55	99	3	7
Verrucomicrobia Arctic95D-9	Bin1509	g__Arctic95D-9	5.28	4609	55.9	93	3	14
	Bin831	g__Arctic95D-9	7.06	6136	55.3	98	3	14
	Bin560	g__Arctic95D-9	5.2	4312	53.1	100	2	0
Verrucomicrobia BACL24	Bin1278	g__BACL24	3.69	3350	53.3	98	3	25
	Bin82	g__BACL24	4.08	3602	52.9	100	1	0

	Bin341	g__BACL24	3.34	3060	54.8	97	2	0
Verrucomicrobia SW10	Bin1259	g__SW10	6.88	5856	55.1	99	5	0
Verrucomicrobia UBA4506	Bin1231	g__UBA4506	3.56	3316	52.5	96	1	0

MetBAT MAGs were generated using MetabAT v2.12.1 and their GTDB (Genome Taxonomy Database) taxonomic classification was assessed using RefineM v0.0.23. Genome completeness, contamination, and strain heterogeneity were calculated using CheckM v1.0.7. See **Methods: Metagenome sequencing, assembly, annotation and overview of analyses** for details on OTU taxonomic classification. CheckM calculates genome contamination from the number of multicopy marker genes in each marker set [45]. Strain heterogeneity indicates the percentage of the contamination that is due to inclusion of gene fragments from multiple strains. For example, Bin802 (*Aquiluna* sp1) has 2% contamination but 100% strain heterogeneity, which indicates that all contamination in *Aquiluna* sp1 genome is due to gene fragments from multiple strains of this species. CheckM calculates strain heterogeneity based on the difference in amino acid identity (AAI) of the multicopy marker genes, to assess whether contaminating gene fragments belong to other strains or other taxa [45]. *GTDB does not provide taxonomic classification for Eukarya and Viruses, therefore, the GTDB taxonomic classifications of MAGs with matches to *Micromonas* and Phycodnaviridae 1-5 OTUs were mostly ‘unclassified’. d__, domain; p__, phylum; f__, family; g__, genus; s__, species.

Table S3 SIMPER analysis showing similarity within each season group and the OTUs contributing to the similarity.

Depth*	Similarity within sample groups and top 5 contributing OTUs		
	**Winter**	**Spring**	**Summer**
**Upper 2**	62%<br>Phycodnaviridae 2	59%<br>Phycodnaviridae 2	40%<br>Phycodnaviridae 2<br>Other viruses<br>*Synechococcus*<br>Phycodnaviridae 3<br>Phycodnaviridae 4<br>Phycodnaviridae 5
**Upper 3**	61%<br>Phycodnaviridae 2<br>*Synechococcus*<br>Other viruses<br>*Nisaea*<br>Microbacteriaceae BACL25	62%<br>Phycodnaviridae 2	42%<br>*Synechococcus*<br>Phycodnaviridae 2
**Interface**	70%<br>*Chlorobium*	55%<br>*Chlorobium*	43%<br>*Chlorobium*
**Lower 1**	NA	70%<br>Syntrophales<br>Cloacimonetes<br>Desulfatiglanales NaphS2<br>Omnitrophica<br>Bacteroidales UBA4459<br>Atribacteria 34-128	52%<br>*Chlorobium*
**Lower 2**	NA	69%<br>Syntrophales<br>Cloacimonetes<br>Desulfatiglanales NaphS2<br>Atribacteria 34-128	51%<br>*Chlorobium*<br>Syntrophales<br>Cloacimonetes
**Lower 3**	NA	71%<br>Atribacteria 34-128	53%<br>Atribacteria 34-128<br>Cloacimonetes<br>Syntrophales

* SIMPER analysis was not conducted on the samples from Upper 1, as they were collected only during summer. The lower zone was not sampled in winter. NA, not available.

Table S4 SIMPER analysis showing dissimilarity between season groups and the OTUs contributing to the dissimilarity.

Depth*	Season	Dissimilarity between sample groups and top 3 contributing OTUs	
		**Spring**	**Summer**
**Upper 2**	**Winter**	40%<br>*Synechococcus*	53%<br>*Synechococcus*<br>Verrucomicrobia
	**Spring**	NA	52%<br>*Synechococcus*
**Upper 3**	**Winter**	37%<br>*Synechococcus*	51%<br>*Synechococcus*
	**Spring**	NA	50%<br>*Synechococcus*
**Interface**	**Winter**	43%<br>*Chlorobium*	56%<br>*Chlorobium*
	**Spring**	NA	55%<br>*Chlorobium*
**Lower 1**	**Spring**	NA	41%<br>*Chlorobium*<br>Desulfatiglanales NaphS2
**Lower 2**	**Spring**	NA	41%<br>Syntrophales<br>*Chlorobium*
**Lower 3**	**Spring**	NA	39%<br>Cloacimonetes<br>Atribacteria 34-128<br>*Chlorobium*

* SIMPER analysis was not conducted on the samples from Upper 1, as they were collected only during summer. The lower zone was not sampled in winter. NA, not available.

Table S5 Metabolic traits of the abundant OTUs identified in Ace Lake.

OTU bin	Taxonomy	Predicted properties based on gene content
Synechococcus	Cyanobacteria, Synechococcales, Synechococcaceae, *Synechococcus*, *Synechococcus* sp. SynAce01	Aerobic oxygenic photoautotroph (Calvin cycle) in the light, chlorophyll-based.<br>Possible aerobic heterotroph under dark conditions, using exogenous sugars and glycerol; however, these may be used as precursors for compatible solute biosynthesis.<br>Possible facultative anaerobe under dark and anoxic conditions: fermentation using stored glycogen coupled to evolution of H_2.<br>Glycolysis via Entner-Doudoroff pathway.<br>Pentose phosphate pway.<br>Tricarboxylic acid cycle (oxidative).<br>Aerobic respiration.<br>C sources: CO_2, urea, cyanate, sugars.<br>Glycogen storage.<br>N sources: nitrate, ammonia, urea, cyanate, amino acids, peptides.<br>S sources: sulfate (by assimilatory sulfate reduction), arylsulfates (by arylsulfatase and assimilatory sulfate reduction).<br>Sulfide oxidation to sulfur/polysulfide (possibly for detoxification).<br>ABC transporters (urea, amino acids, sugars).<br>Other transporters (peptides).
Algoriphagus	Bacteroidetes, Sphingobacteria, Sphingobacteriales, Cyclobacteriaceae, *Algoriphagus*	Aerobic heterotroph.<br>Glycolytic pathway unclear.<br>Tricarboxylic acid cycle (oxidative).<br>Aerobic respiration.<br>C sources include starch, fucoidan, xylan, β-glucans, levan, chitin, sulfate esters, peptides, amino acids.<br>N sources include peptides, amino acids, ammonia, chitin.<br>S sources include sulfate (by assimilatory sulfate reduction), sulfate esters.<br>TRAP transporter.<br>TonB-dependent receptors/transporters.
Leadbetterella	Bacteroidetes, Cytophagia, Cytophagales, Cytophagaceae,	Aerobic heterotroph, including photoheterotrophy (rhodopsin-based).<br>Glycolysis: Embden-Meyerhof-Parnas pathway.<br>Pentose phosphate pathway.<br>Tricarboxylic acid cycle (oxidative).

	Leadbetterella	Aerobic respiration.<br>C sources include starch, β-glucans, fucoidan (and possibly other sulfate esters), mannan, chitin, glucosylceramide, glycerol, peptides, amino acids.<br>N sources include ammonia, amino acids, peptides, chitin.<br>S sources include sulfate (by assimilatory sulfate reduction), sulfate esters.<br>TonB-dependent receptors/transporters.<br>TRAP transporter.<br>POT transporter (peptides).
Nonlabens	Bacteroidetes, Bacteroidia, Flavobacteriales, Flavobacteriaceae, *Nonlabens*	Aerobic heterotroph, including photoheterotrophy (rhodopsin-based).<br>Glycolysis: Embden-Meyerhof-Parnas pathway.<br>Tricarboxylic acid cycle (oxidative).<br>Aerobic respiration.<br>C sources include glycoproteins, peptides.<br>Glycogen/starch storage.<br>N sources include ammonia, glycoproteins, peptides.<br>S sources: possibly organic.<br>TonB-dependent receptors/transporters.
Saprospiraceae sp.	Bacteroidetes, Sphingobacteria, Sphingobacteriales, Saprospiraceae	Aerobic heterotroph, including photoheterotrophy (rhodopsin-based).<br>Glycolysis: Embden-Meyerhof-Parnas pathway.<br>Tricarboxylic acid cycle (oxidative) (including glyoxylate bypass).<br>Aerobic respiration.<br>C sources include β-glucans, glucosylceramide, peptides, glycerol, acetate.<br>N sources include ammonia, peptides.<br>S sources: possibly organic.<br>TonB-dependent receptors/transporters.
Polaribacter	Bacteroidetes, Bacteroidia, Flavobacteriales, Flavobacteriaceae, *Polaribacter*	Aerobic heterotroph, including photoheterotrophy (rhodopsin-based).<br>Glycolysis: Embden-Meyerhof-Parnas pathway.<br>Pentose phosphate pathway.<br>Tricarboxylic acid cycle (oxidative) (including glyoxylate bypass).<br>Aerobic respiration.<br>C sources include cellulose, starch, xylan, mannan, arabinogalactan, β-glucans, glucosylceramide, oligosaccharides, sulfate esters (possibly including sulfoglycans), peptides, amino acids, glycerol, acetate.

		Glycogen/starch storage.<br>N sources: ammonia, nitrate, peptides.<br>S sources: possibly organic.<br>TonB-dependent receptors/transporters.<br>POT transporter (peptides).
Crocinitomix	Bacteroidetes,<br>Bacteroidia,<br>Flavobacteriales,<br>Crocinitomicaceae,<br>*Crocinitomix*	Aerobic heterotroph, including photoheterotrophy (rhodopsin-based).<br>Glycolysis: Embden-Meyerhof-Parnas pathway, Entner-Doudoroff pathway.<br>Tricarboxylic acid cycle (oxidative).<br>Aerobic respiration.<br>C sources include β-glucans, κ-carrageen, mannosides, oligosaccharides, simple sugars, peptides, amino acids.<br>Sulfide oxidation to sulfur/polysulfide (possibly for detoxification).<br>N sources include peptides, amino acids, ammonia.<br>S sources include sulfate (by assimilatory sulfate reduction).<br>TonB-dependent receptors/transporters.<br>POT transporter (peptides).
Cyclobacterium	Bacteroidetes,<br>Bacteroidia,<br>Cytophagales,<br>Cyclobacteriaceae,<br>*Cyclobacterium*	Aerobic heterotroph, including photoheterotrophy (rhodopsin-based).<br>Glycolysis: Embden-Meyerhof-Parnas pathway.<br>Tricarboxylic acid cycle (oxidative).<br>Aerobic respiration.<br>C sources include β-glucans, xyloglucan, xylan, levan, chitin, fucoidan, rhamnogalacturonan, rhamnosides, levan, oligosaccharides, glycerol, sulfate esters, heparan sulfate, peptides, amino acids.<br>Sulfide oxidation to sulfur/polysulfide (possibly detoxification).<br>N sources include peptides, amino acids, ammonia.<br>S sources include sulfate (by assimilatory sulfate reduction), sulfate esters, heparan sulfate.<br>TonB-dependent receptors/transporters.<br>TRAP transporters.<br>POT transporter (peptides).<br>Polyhydroxyalkanoate storage.
Fabibacter	Bacteroidetes,<br>Bacteroidia,<br>Cytophagales,<br>Cyclobacteriaceae,	Aerobic heterotroph, including photoheterotrophy (rhodopsin-based).<br>Glycolysis: Embden-Meyerhof-Parnas pathway.<br>Tricarboxylic acid cycle (oxidative).<br>Aerobic respiration.

	Fabibacter, *Fabibacter* sp1	C sources include β-glucans, starch, levan, fucoidan, oligosaccharides, glycerol, sulfate esters, peptides, amino acids.<br>N sources include peptides, amino acids, ammonia.<br>S sources: possibly organic.<br>TonB-dependent receptors/transporters.<br>POT transporter (peptides).
Flavobacteriaceae MAG-120531	Bacteroidetes, Bacteroidia, Flavobacteriales, Flavobacteriaceae, genus MAG-120531	Aerobic heterotroph, including photoheterotrophy (rhodopsin-based).<br>Glycolysis: Embden-Meyerhof-Parnas pathway.<br>Tricarboxylic acid cycle (oxidative).<br>Aerobic respiration.<br>C sources include β-glucans, starch, levan, oligosaccharides, glycerol, sulfate esters, peptides, amino acids.<br>N sources include nitrite, peptides, amino acids, ammonia.<br>S sources: possibly organic.<br>TonB-dependent receptors/transporters.<br>POT transporter (peptides).
Bacteroidales UBA4459	Bacteroidetes, Bacteroidales, family F082, genus UBA4459	Anaerobic heterotroph (possibly for fermentation only).<br>Glycolysis: Embden-Meyerhof-Parnas pathway.<br>Pentose phosphate pathway.<br>Tricarboxylic acid cycle (oxidative).<br>Fermentation coupled to cytosolic H_2-evolving hydrogenases: reduction of protons (to H_2) to re-oxidize reduced cofactors.<br>C sources include starch, xylan/xyloglucan, mannan, β-glucans, simple sugars, cellobiose, fucoidan (and possibly other sulfate esters), glucosylceramide, peptides, amino acids, glycerol.<br>N sources include ammonia, amino acids, peptides, taurine.<br>Nitrous oxide dissimilation.<br>S sources: possibly organic.<br>ABC transporters (BCAA, sugars).<br>TonB-dependent receptors/transporters.<br>Sulfide oxidation to sulfur/polysulfide.
Balneolaceae UBA2664	Bacteroidetes, Rhodothermia, Balneolales,	Aerobic heterotroph (facultative anaerobic), including photoheterotrophy (rhodopsin-based).<br>Glycolysis: Embden-Meyerhof-Parnas pathway.<br>Pentose phosphate pathway.

	Balneolaceae,<br>genus UBA2664	Tricarboxylic acid cycle (oxidative).<br>Aerobic respiration.<br>C sources include cellulose, β-glucans, rhamnosides, sulfate esters, peptides, amino acids, glycerol.<br>Glycogen/starch storage.<br>N sources include ammonia, amino acids, peptides.<br>Nitrite and nitrous oxide dissimilation (possibly respiratory).<br>S sources: possibly organic.<br>TRAP transporter.<br>TonB-dependent receptors/transporters.
Verrucomicrobia BACL24	Verrucomicrobia,<br>Opitutae, Opitutales,<br>Opitutaceae,<br>genus BACL24	Aerobic heterotroph, including photoheterotrophy (rhodopsin-based).<br>Glycolysis: Embden-Meyerhof-Parnas pathway.<br>Pentose phosphate pathway.<br>Tricarboxylic acid cycle (oxidative).<br>Aerobic respiration (possibly facultative).<br>C sources include starch, fucoidan (and possibly other sulfate esters), xylan, β-glucans, chitin, mannan, rhamnosides, heparan sulfate, oligosaccharides, glycerol, peptides, amino acids.<br>Glycogen/starch storage.<br>N sources include ammonia, urea, nitrate, peptides, amino acids, chitin.<br>Nitrate dissimilation.<br>S sources include sulfate (by assimilatory sulfate reduction), sulfate esters, heparan sulfate<br>ABC transporter (peptides).
Verrucomicrobia UBA4506	Verrucomicrobia,<br>Opitutae,<br>genus UBA4506	Aerobic heterotroph, including photoheterotrophy (rhodopsin-based).<br>Glycolysis: Embden-Meyerhof-Parnas pathway.<br>Pentose phosphate pathway.<br>Tricarboxylic acid cycle (oxidative).<br>Aerobic respiration.<br>C sources include starch, fucoidan (and possibly other sulfate esters), xylan, β-glucan, cellulose, chitin, mannan, heparan sulfate, oligosaccharides, amino acids.<br>Microcompartments (for fucose degradation by-products).<br>Glycogen/starch storage.<br>N sources include ammonia, amino acids, chitin.<br>S sources include sulfate (by assimilatory sulfate reduction), sulfate esters, heparan sulfate<br>ABC transporter (peptides).<br>TRAP transporter.

		Assimilatory sulfate reduction.
Verrucomicrobia Arctic95D-9	Verrucomicrobia, Verrucomicrobiae, Verrucomicrobiales, family DEV007, genus Arctic95D-9	Aerobic heterotroph.<br>Glycolysis: Embden-Meyerhof-Parnas pathway, Entner-Doudoroff pathway.<br>Pentose phosphate pathway.<br>Tricarboxylic acid cycle (oxidative).<br>Aerobic respiration.<br>C sources include β-glucans, cellulose, chitosan/chitooligosaccharides, glycerol, sulfate esters, heparan sulfate, peptides, amino acids.<br>N sources include ammonia, urea, nitrate, peptides, amino acids.<br>S sources include sulfate (by assimilatory sulfate reduction), sulfate esters, heparan sulfate.<br>TonB-dependent receptors/transporters.<br>POT transporter (peptides).
Verrucomicrobia SW10	Verrucomicrobia, Verrucomicrobiae, Verrucomicrobiales, Rubritaleaceae, genus SW10	Aerobic heterotroph.<br>Glycolysis: Embden-Meyerhof-Parnas pathway.<br>Pentose phosphate pathway.<br>Tricarboxylic acid cycle (oxidative).<br>Aerobic respiration.<br>C sources include β-glucans, chitosan/chitooligosaccharides, rhamnosides, oligosaccharides, sulfate esters, heparan-sulfate, peptides, amino acids.<br>N sources include ammonia, nitrate, peptides, amino acids.<br>S sources include sulfate (by assimilatory sulfate reduction), sulfate esters, heparan sulfate.<br>ABC transporter (ribose).<br>POT transporter (peptides).
Haloferula	Verrucomicrobia, Verrucomicrobiae, Verrucomicrobiales, Rubritaleaceae, *Haloferula*	Aerobic heterotroph, including photoheterotrophy (rhodopsin-based).<br>Glycolysis: Embden-Meyerhof-Parnas pathway.<br>Pentose phosphate pathway.<br>Tricarboxylic acid cycle (oxidative).<br>Aerobic respiration.<br>C sources include β-glucans, rhamnosides, chitosan/chitooligosaccharides, oligosaccharides, sulfate esters, heparan-sulfate, peptides, amino acids.<br>N sources include ammonia, nitrate, peptides, amino acids.<br>S sources include sulfate (by assimilatory sulfate reduction), sulfate esters, heparan sulfate.<br>TonB-dependent receptors/transporters.

		POT transporter (peptides).
Gimesia	Planctomycetes,<br>Planctomycetales,<br>Planctomycetaceae,<br>*Gimesia*	Aerobic heterotroph (facultative anaerobic).<br>Glycolysis: Embden-Meyerhof-Parnas pathway.<br>Pentose phosphate pathway.<br>Tricarboxylic acid cycle (oxidative).<br>Aerobic respiration.<br>Fermentation, H_2 generated by cytosolic hydrogenase (also possible sulfhydrogenase, in which case sulfide released).<br>C sources include fucoidan (and other sulfate esters), mannan, κ-carrageen, rhamnose, ribose, glycerol, glycolate, amino acids.<br>Glycogen/starch storage.<br>Microcompartments (for fucose and rhamnose degradation by-products).<br>N sources include ammonia, amino acids, 2-aminoethylphosphonate, nitrate.<br>P sources include 2-aminoethylphosphonate.<br>S sources include sulfate (by assimilatory sulfate reduction), sulfate esters.<br>Sulfide oxidation to sulfur/polysulfide.<br>ABC transporter (ribose).<br>TRAP transporter (ectoine).<br>POT transporter (peptides).<br>Flagella.
Aquiluna	Actinobacteria,<br>Actinomycetales,<br>Microbacteriaceae,<br>*Aquiluna*	Aerobic heterotroph, including photoheterotrophy (rhodopsin-based).<br>Glycolysis: Embden-Meyerhof-Parnas pathway.<br>Pentose phosphate pathway.<br>Tricarboxylic acid cycle (oxidative).<br>Aerobic respiration.<br>C sources include simple sugars, glycerol, mannitol, 2-oxoacids, dicarboxylates, amino acids.<br>N sources include ammonia, amino acids, taurine.<br>S sources: possibly organic.<br>ABC transporters (fructose, ribose, xylose, amino acids, glycerol, putrescine/spermidine).<br>TRAP transporter.<br>Phosphotransferase system for uptake (mannitol).
Microbacteriaceae BACL25	Actinobacteria,<br>Actinomycetales,	Aerobic heterotroph, including photoheterotrophy (rhodopsin-based).<br>Glycolysis: Embden-Meyerhof-Parnas pathway.

	Microbacteriaceae,<br>genus BACL25,<br>species BACL25 sp1	Pentose phosphate pathway.<br>Tricarboxylic acid cycle (oxidative) (including glyoxylate bypass).<br>Aerobic respiration.<br>C sources include oligosaccharides, simple sugars, glycerol, peptides, amino acids, acetate.<br>N sources include ammonia, amino acids, taurine.<br>S sources: possibly organic.<br>ABC transporters (fructose, ribose, amino acids, peptides, glycerol).<br>TRAP transporter.
Pelagibacter	Proteobacteria,<br>Alphaproteobacteria,<br>Pelagibacterales,<br>Pelagibacteraceae,<br>*Pelagibacter*	Aerobic heterotroph, including photoheterotrophy (rhodopsin-based).<br>Glycolysis: Embden-Meyerhof-Parnas pathway.<br>Pentose phosphate pathway.<br>Tricarboxylic acid cycle (oxidative) (including glyoxylate bypass).<br>Aerobic respiration.<br>C sources include ribose, amino acids, glycerol, glycerol-3-phosphate, glycolate, taurine, acetate, aliphatic amides, organic acids, DHPS, DMSP, 2-oxoacids, dicarboxylates, aldonates.<br>N sources include ammonia, amino acids, taurine, creatine, sarcosine.<br>Nitrite dissimilation (nitric oxide forming).<br>S sources include DMSP, S-containing amino acids.<br>Anaerobic methylthioadenosine pathway (methionine salvage; ethylene released as by-product).<br>ABC transporters (ribose, amino acids, glycerol, glycerol-3-phosphate, taurine, aliphatic amides).<br>TRAP transporters (2-oxoacids, dicarboxylates, aldonates).
Loktanella	Proteobacteria,<br>Alphaproteobacteria,<br>Rhodobacterales,<br>Rhodobacteraceae,<br>*Roseobacter* clade,<br>*Loktanella*	Aerobic heterotroph, including photoheterotrophy (rhodopsin-based).<br>Glycolysis: Entner-Doudoroff pathway.<br>Tricarboxylic acid cycle (oxidative).<br>Pentose phosphate pathway.<br>Aerobic respiration.<br>C sources include oligosaccharides, simple sugars, amino acids, peptides, glycerol, glycolate, 2-oxoacids, dicarboxylates, aldonates, DMSP.<br>Glycogen/starch storage.<br>Polyhydroxyalkanoate storage.<br>CO oxidation to CO_2.<br>N sources include ammonia, amino acids, peptides, urea, hypotaurine, creatine, sarcosine.<br>P sources include methylphosphonate (methane released as by-product).<br>S sources include sulfate (by assimilatory sulfate reduction), DMSP, hypotaurine.

		Sulfide oxidation to sulfur/polysulfide.<br>Thiosulfate oxidation to sulfate (Sox system).<br>ABC transporters (fructose, ribose, xylose, amino acids, peptides, glycerol, phosphonates, urea, putrescine/spermidine).<br>TRAP transporters (2-oxoacids, dicarboxylates, aldonates, indole acids).
Yoonia	Proteobacteria, Alphaproteobacteria, Alphaproteobacteria, Rhodobacterales, Rhodobacteraceae, *Roseobacter* clade, *Yoonia vestfoldensis*	Aerobic heterotroph, including photoheterotrophy (both bacteriochlorophyll- and rhodopsin-based).<br>Glycolysis: Entner-Doudoroff pathway.<br>Pentose phosphate pathway.<br>Tricarboxylic acid cycle (oxidative).<br>Aerobic respiration.<br>C sources include simple sugars, amino acids, peptides, glycerol, glycolate, 2-oxoacids, dicarboxylates, aldonates, DMSP.<br>Polyhydroxyalkanoate storage.<br>CO oxidation to CO_2.<br>N sources include ammonia, amino acids, peptides, urea, taurine, creatine, sarcosine.<br>P sources include methylphosphonate (methane released as by-product).<br>S sources include sulfate (by assimilatory sulfate reduction), DMSP (dimethylsulfide released as by-product), taurine.<br>Sulfide oxidation to sulfur/polysulfide.<br>Thiosulfate oxidation to sulfate (Sox system).<br>ABC transporters (fructose, ribose, xylose, amino acids, peptides, glycerol, taurine, phosphonates, urea, putrescine/spermidine).<br>TRAP transporters (2-oxoacids, dicarboxylates, aldonates, indole acids).
Nisaea	Proteobacteria, Alphaproteobacteria, Thalassobaculales, Thalassobaculaceae, *Nisaea* (formerly alphaproteobacterium BAL199)	Aerobic heterotroph, including rhodopsin-based photoheterotrophy.<br>Glycolysis: Embden-Meyerhof-Parnas pathway.<br>Pentose phosphate pathway.<br>Tricarboxylic acid cycle (oxidative) (including glyoxylate bypass).<br>Aerobic respiration.<br>C sources include simple sugars, amino acids, peptides, glycerol, glycerol-3-phosphate, glycolate, 2-oxoacids.<br>Polyhydroxyalkanoate storage.<br>CO oxidation to CO_2.<br>N sources include ammonia, amino acids, peptides, creatine, sarcosine, urea, 2-aminoethylphosphonate.

		Nitrate and nitrite dissimilation (nitric oxide forming).<br>P sources include 2-aminoethylphosphonate.<br>S sources include sulfate (by assimilatory sulfate reduction).<br>Anaerobic methylthioadenosine pathway (methionine salvage; ethylene released as by-product).<br>Thiosulfate oxidation to sulfate (Sox system).<br>ABC transporters (ribose, BCAA, peptides, glycerol-3-phosphate, phosphonates, urea, putrescine/spermidine).<br>TRAP transporters (2-oxoacids).<br>Flagella.
Pseudomonas E	Proteobacteria, Gammaproteobacteria, Pseudomonadales, Pseudomonadaceae, *Pseudomonas* E	Aerobic heterotroph.<br>Glycolysis: Embden-Meyerhof-Parnas pathway, Entner-Doudoroff pathway.<br>Pentose phosphate pathway.<br>Tricarboxylic acid cycle (oxidative) (including glyoxylate bypass).<br>Aerobic respiration.<br>C sources include starch, β-glucans, simple sugars, carboxylates, glycerol, peptides, amino acids, acetate, glycolate, aromatic compounds.<br>Polyhydroxyalkanoate storage.<br>N sources include nitrate, ammonia, amino acids, peptides, sarcosine, urea, cyanate, ethanolamine, taurine.<br>Nitrate dissimilation (to nitrite).<br>Nitric oxide conversion to nitrate (detoxification).<br>S sources include sulfate (by assimilatory sulfate reduction), taurine.<br>P sources include methylphosphonate (methane released as by-product).<br>ABC transporters (simple sugars, glycerol, peptides, amino acids, urea, phosphonate, sulfonate).<br>TRAP transporters (dicarboxylates, possibly aromatic compounds)<br>TonB-dependent receptors/transporters.<br>Flagella.<br>Type IV pili.
Pseudohongiellaceae 1	Proteobacteria, Gammaproteobacteria, Pseudomonadales, Pseudohongiellaceae, genus OM182	Aerobic heterotroph.<br>Glycolysis: Embden-Meyerhof-Parnas pathway.<br>Pentose phosphate pathway.<br>Tricarboxylic acid cycle (oxidative).<br>Aerobic respiration.<br>C sources include amino acids, peptides.

		N sources include ammonia, amino acids, peptides.<br>S sources include sulfate (by assimilatory sulfate reduction).<br>TRAP transporter (glutamate/glutamine).<br>POT transporter (peptides).
Pseudohongiellaceae 2	Proteobacteria, Gammaproteobacteria, Pseudomonadales, Pseudohongiellaceae, genus OM182, OM182 sp 1	Aerobic heterotroph, including photoheterotrophy (rhodopsin-based).<br>Glycolysis: Embden-Meyerhof-Parnas pathway.<br>Pentose phosphate pathway.<br>Tricarboxylic acid cycle (oxidative).<br>Aerobic respiration.<br>C sources include glycerol, amino acids, peptides, malonate.<br>N sources include ammonia, amino acids, peptides, urea.<br>S sources include sulfate (by assimilatory sulfate reduction).<br>Sulfide oxidation to sulfur/polysulfide.<br>Sulfite oxidation.<br>ABC transporters (ribose, peptides, amino acids).<br>TRAP transporter.<br>POT transporter (peptides).<br>Flagella.<br>Type IV pili.
Halioglobus	Proteobacteria, Gammaproteobacteria, Pseudomonadales, Halieaceae, *Halioglobus*	Aerobic heterotroph.<br>Glycolysis: Embden-Meyerhof-Parnas pathway.<br>Pentose phosphate pathway.<br>Tricarboxylic acid cycle (oxidative) (including glyoxylate bypass).<br>Aerobic respiration.<br>C sources include xylan, mannan, sugars, levan, fucoidan, glycerol, amino acids, peptides, acetate, glycolate.<br>N sources include ammonia, amino acids, peptides, urea.<br>S sources include sulfate (by assimilatory sulfate reduction) and possibly sulfate esters (by sulfatases and assimilatory sulfate reduction).<br>Sulfide oxidation to sulfur/polysulfide.<br>ABC transporters (peptides, amino acids).<br>TRAP transporter.<br>POT transporter (peptides).

Porticoccaceae HTCC2207	Proteobacteria, Gammaproteobacteria, Pseudomonadales, Porticoccaceae, genus HTCC2207	Aerobic heterotroph, including photoheterotrophy (rhodopsin-based).<br>Glycolysis: Embden-Meyerhof-Parnas pathway, Entner-Doudoroff pathway.<br>Pentose phosphate pathway.<br>Tricarboxylic acid cycle (oxidative) (including glyoxylate bypass).<br>Aerobic respiration.<br>C sources include β-glucans, oligosaccharides, sugars, glycerol, peptides, amino acids, acetate.<br>N sources include ammonia, peptides, amino acids, sarcosine.<br>S sources include sulfate (by assimilatory sulfate reduction).<br>ABC transporters (peptides, possibly sulfonates).<br>Type IV pili.<br>POT transporter (peptides).
Hydrogenophaga	Proteobacteria, Betaproteobacteria, Burkholderiales, Comamonadaceae, *Hydrogenophaga*	Aerobic heterotroph, including photoheterotrophy (both bacteriochlorophyll- and rhodopsin-based).<br>Glycolysis: Embden-Meyerhof-Parnas pathway, Entner-Doudoroff pathway.<br>Pentose phosphate pathway.<br>Tricarboxylic acid cycle (oxidative) (including glyoxylate bypass).<br>Aerobic respiration.<br>C sources include simple sugars, amino acids, peptides, glycerol, glycerol-3-phosphate, 2-oxoacids, aldonates, DHPS.<br>Polyhydroxyalkanoate storage.<br>CO oxidation to CO_2.<br>N sources include ammonia, amino acids, peptides, cyanate, nitrate.<br>S sources include sulfate (by assimilatory sulfate reduction), DHPS.<br>Sulfide oxidation to sulfur/polysulfide.<br>Thiosulfate oxidation to sulfate (Sox system).<br>ABC transporters (amino acids, peptides, glycerol-3-phosphate, putrescine/spermidine).<br>TRAP transporters (2-oxoacids, aldonates).<br>Flagella.
Burkholderiaceae MOLA814	Proteobacteria, Betaproteobacteria, Burkholderiales, Burkholderiaceae, genus RS62, Betaproteobacteria bacterium sp.	Aerobic heterotroph, including photoheterotrophy (rhodopsin-based).<br>Glycolysis: Embden-Meyerhof-Parnas pathway, Entner-Doudoroff pathway.<br>Pentose phosphate pathway.<br>Tricarboxylic acid cycle (oxidative) (including glyoxylate bypass).<br>Aerobic respiration.<br>C sources include simple sugars, amino acids, 2-oxoacids, glycerol, organic acids, DHPS, sulfoacetaldehyde.

	MOLA814	Polyhydroxyalkanoate storage.<br>CO oxidation to CO_2.<br>N sources include ammonia, amino acids, urea, cyanate.<br>P sources include methylphosphonate (methane released as by-product).<br>S sources include sulfate (by assimilatory sulfate reduction), DHPS, sulfoacetaldehyde.<br>Thiosulfate oxidation to sulfate (Sox system).<br>ABC transporters (ribose, amino acids, phosphonates, urea).<br>TRAP transporters (2-oxoacids).
Burkholderiaceae SCGC-AAA027-K21	Proteobacteria, Betaproteobacteria, Burkholderiaceae, genus SCGC-AAA027-K21	Aerobic heterotroph.<br>Glycolysis: Embden-Meyerhof-Parnas pathway.<br>Pentose phosphate pathway.<br>Tricarboxylic acid cycle (oxidative) (including glyoxylate bypass).<br>C sources include levan, sugars, amino acids, peptides, glycerol, 2-oxoacids, dicarboxylates, acetate, glycolate, cysteate.<br>Polyhydroxyalkanoate storage.<br>CO oxidation to CO_2.<br>N sources include ammonia, amino acids, peptides, urea, cysteate.<br>S sources: possibly organic.<br>Sulfide oxidation to sulfur/polysulfide.<br>Sulfite oxidation.<br>Thiosulfate oxidation to sulfate (Sox system).<br>ABC transporters (simple sugars, amino acids, urea).<br>TRAP transporters (2-oxoacids, dicarboxylates).<br>POT family (peptides).
Methylophilaceae BACL14	Proteobacteria, Betaproteobacteria, Methylophilaceae, genus BACL14	Aerobic heterotroph, including photoheterotrophy (rhodopsin-based).<br>Glycolysis: Embden-Meyerhof-Parnas pathway, Entner-Doudoroff pathway.<br>Pentose phosphate pathway.<br>Tricarboxylic acid cycle (oxidative).<br>Aerobic respiration.<br>Methanol and formate oxidation (energy sources only).<br>C sources include simple sugars, amino acids, peptides.<br>N sources include ammonia, amino acids, peptides.<br>S sources include sulfate (by assimilatory sulfate reduction).

Oligoflexus	Proteobacteria,<br>Oligoflexia,<br>Oligoflexales,<br>Oligoflexaceae,<br>*Oligoflexus,*<br>*Oligoflexus tunisiensis*	Aerobic heterotroph.<br>Glycolysis: Embden-Meyerhof-Parnas pathway.<br>Pentose phosphate pathway.<br>Tricarboxylic acid cycle (oxidative).<br>Aerobic respiration.<br>C sources include amino acids, peptides, glycerol, starch.<br>N sources include amino acids, peptides, ammonia.<br>ABC transporters (peptides).<br>TonB-dependent receptors/transporters.<br>POT transporter (peptides).<br>S sources: possibly organic.<br>Flagella.
Chlorobium	Chlorobi, Chlorobia,<br>Chlorobiales,<br>Chlorobiaceae,<br>*Chlorobium*	Anoxygenic photoautotroph (reverse tricarboxylic acid cycle), bacteriochlorophyll-based.<br>Glycolysis: Embden-Meyerhof-Parnas pathway.<br>Pentose phosphate pathway.<br>Tricarboxylic acid cycle (oxidative).<br>Photoassimilation of simple organic compounds (acetate, propionate, pyruvate).<br>Glycogen storage.<br>N sources: ammonia, dinitrogen (releases H_2 as by-product).<br>Sulfide oxidation to sulfate via sulfur/polysulfide.<br>Obligate anaerobe.
Desulfobacterium	Proteobacteria,<br>Deltaproteobacteria,<br>Desulfobacterales,<br>Desulfobacteraceae,<br>*Desulfobacterium*	Anaerobic autotroph (Wood-Ljungdahl pathway) and heterotroph.<br>Glycolysis: Embden-Meyerhof-Parnas pathway.<br>Pentose phosphate pathway.<br>Tricarboxylic acid cycle (oxidative).<br>Sulfate and fumarate respiration.<br>C sources include CO_2, CO, amino acids, glycerol, glycerol-3-phosphate, peptides, 2-oxoacids, dicarboxylates, cellulose.<br>Glycogen/starch storage.<br>CO oxidation to CO_2.<br>N sources include ammonia, amino acids, taurine.<br>ABC transporters (amino acids, glycerol, glycerol-3-phosphate, peptides/glutathione, putrescine/spermidine, taurine).<br>TRAP transporters (2-oxoacids, dicarboxylates, indole acids).

		H_2 oxidation via membrane-bound hydrogenase.<br>Flagella.<br>Gas vesicles.
Desulfatiglanales NaphS2	Proteobacteria,<br>Deltaproteobacteria,<br>Desulfatiglanales,<br>family NaphS2,<br>genus NaphS2	Anaerobic heterotroph and autotroph (Wood-Ljungdahl pathway).<br>Glycolysis: Embden-Meyerhof-Parnas pathway.<br>Pentose phosphate pathway.<br>Tricarboxylic acid cycle (oxidative).<br>Sulfate and fumarate respiration.<br>C sources include CO_2, CO, simple sugars, amino acids, peptides, glycerol, chitin, organic acids.<br>Polyhydroxyalkanoate storage.<br>CO oxidation to CO_2.<br>N sources include ammonia, amino acids, peptides, taurine, chitin.<br>H_2 generated by cytosolic hydrogenase (also possible sulfhydrogenase, in which case sulfide released).<br>H_2 oxidation via membrane-bound hydrogenase.<br>ABC transporters (ribose, BCAA, peptides, glycerol).<br>TRAP transporters (2-oxoacids, dicarboxylates, indole acids).<br>Gas vesicles.
Desulfobacterales S5133MH16	Proteobacteria,<br>Deltaproteobacteria,<br>Desulfobacterales,<br>family BuS5,<br>genus S5133MH16	Anaerobic autotroph (Wood-Ljungdahl pathway) and heterotroph.<br>Glycolysis: Embden-Meyerhof-Parnas pathway.<br>Pentose phosphate pathway.<br>Tricarboxylic acid cycle (oxidative).<br>Sulfate respiration.<br>C sources include CO_2, formate, amino acids.<br>Glycogen/starch storage.<br>N sources include ammonia, amino acids.<br>ABC transporters (amino acids).<br>TRAP transporters (indole acids).<br>Type IV pili.
Desulfocapsa	Proteobacteria,<br>Deltaproteobacteria,<br>Desulfobacterales,<br>Desulfobulbaceae,	Anaerobic autotroph (Wood-Ljungdahl pathway) and heterotroph.<br>Glycolysis: Embden-Meyerhof-Parnas pathway, Entner-Doudoroff pathway.<br>Pentose phosphate pathway.<br>Tricarboxylic acid cycle (oxidative).

	Desulfocapsa	Sulfur and thiosulfate disproportionation (autotrophic).<br>C sources include CO_2, amino acids, glycerol, glycerol-3-phosphate, 2-oxoacids, dicarboxylates.<br>Glycogen/starch storage.<br>N sources include ammonia, dinitrogen (releases H_2 as by-product).<br>Nitrate and nitrite dissimilation (to ammonia).<br>P sources include methylphosphonate (methane released as by-product).<br>Sulfide oxidation to sulfur/polysulfide.<br>H_2 generated by cytosolic hydrogenase (also possible sulfhydrogenase, in which case sulfide released).<br>H_2 oxidation via membrane-bound hydrogenase.<br>ABC transporters (amino acids, glycerol, glycerol-3-phosphate, phosphonates, putrescine/spermidine).<br>TRAP transporters (2-oxoacids, dicarboxylates, indole acids).<br>Phosphotransferase system for uptake (possibly glucose).<br>Flagella.<br>Gas vesicles.
Syntrophales UBA2210	Proteobacteria, Deltaproteobacteria, Syntrophales, family UBA2210, genus UBA2210	Anaerobic heterotroph (fermentation only).<br>Glycolysis: Embden-Meyerhof-Parnas pathway.<br>Pentose phosphate pathway.<br>Tricarboxylic acid cycle (oxidative).<br>Reduction of sulfur/polysulfide (to sulfide) to re-oxidize reduced cofactors during fermentation.<br>C sources include glycerol, glycerol-3-phosphate (possibly from phospholipids), amino acids, peptides, 2-oxoacids.<br>Formate oxidation.<br>N sources include ammonia, amino acids, peptides.<br>ABC transporters (amino acids, glycerol-3-phosphate).<br>TRAP transporters (2-oxoacids, indole acids).<br>Phosphotransferase system for uptake (possibly acetylgalactosamine).
Izimaplasma	Tenericutes, Izimaplasmatales, Izimaplasmataceae, *Izimaplasma*	Anaerobic heterotroph (fermentation only).<br>Glycolysis: Embden-Meyerhof-Parnas pathway.<br>Pentose phosphate pathway.<br>Incomplete tricarboxylic acid cycle.<br>Fermentation coupled to cytosolic H_2-evolving hydrogenase: reduction of protons (to H_2) to re-oxidize reduced cofactors.

		Formate oxidation.<br>C sources include sugars/oligosaccharides, glucosylceramide, amino acids, peptides, glycerol.<br>Glycogen/starch storage.<br>N sources include ammonia, amino acids, peptides.<br>S sources: possibly organic.<br>ABC transporters (peptides).<br>Microcompartments (aldehyde metabolism).
Atribacteria 34-128	Atribacteria,<br>class JS1,<br>order SB-45,<br>family 34-128<br>genus 34-128	Anaerobic heterotroph.<br>Glycolysis: Embden-Meyerhof-Parnas pathway.<br>Pentose phosphate pathway.<br>Tricarboxylic acid cycle (oxidative).<br>Anaerobic respiration via membrane-bound hydrogenase: sodium-motive force generated by Mrp antiporter complex linked to H_2 evolution.<br>Fermentation coupled to cytosolic H_2-evolving hydrogenase: reduction of protons (to H_2) to re-oxidize reduced cofactors.<br>C sources include oligosaccharides, sugars (including fucose), glycerol, amino acids.<br>Microcompartments (for fucose degradation by-products).<br>N sources include ammonia, amino acids, taurine.<br>S sources: possibly organic.<br>ABC transporters (sugars, amino acids)<br>TRAP transporter.
Cloacimonetes JGIOTU-2	Cloacimonetes, Cloacimonadia, order JGIOTU-2, family JGIOTU-2,<br>genus JGIOTU-2	Anaerobic heterotroph (fermentative).<br>Glycolysis: Embden-Meyerhof-Parnas pathway.<br>Anaerobic respiration via membrane-bound hydrogenase: sodium-motive force generated by Mrp antiporter complex linked to H_2 evolution.<br>C sources include β-glucans, chitin, arabinogalactan, sugars, peptides, amino acids.<br>N sources include ammonia, peptides, amino acids.<br>ABC transporter (BCAA).<br>H_2 oxidation via cytosolic hydrogenase (heterodisulfide-linked).
Methanothrix A	Euryarchaeota, Methanomicrobia, Methanosarcinales, Methanotrichaceae,	Anaerobic acetoclastic methanogen using Wood-Ljungdahl pathway.<br>Glycolysis: Embden-Meyerhof-Parnas pathway.<br>C sources include acetate (methane is generated).<br>N sources include ammonia.

	Methanothrix A	S sources: possibly organic.
Methanomicrobiaceae 1	Euryarchaeota, Methanomicrobia, Methanomicrobiales, Methanomicrobiaceae	Anaerobic hydrogenotrophic methanogen using Wood-Ljungdahl pathway.<br>Glycolysis: Embden-Meyerhof-Parnas pathway.<br>Hydrogen oxidation.<br>C sources include CO_2 (methane is generated).<br>N sources include ammonia.<br>S sources: possibly organic.

ABC, ATP-binding cassette; BCAA, branched-chain amino acids; DHPS, 2,3-dihydroxypropane-1-sulfonate; DMSP, dimethylsulfoniopropionate; POT, proton-dependent oligopeptide transporter; TRAP, tripartite ATP-independent periplasmic.

Table S6 Glycoside hydrolases and other glycoconjugate degradation enzymes from the abundant OTUs in Ace Lake.

OTU	Clade	Enzyme	EC	CAZy	Signal peptide	Function and % sequence identity[a]
**Chitin degradation**						
**chitinase** (3.2.1.14)						Endo-hydrolysis of N-acetyl-β-D-glucosaminide (1→4)-beta-linkages in chitin and chitodextrins.
BACL24	Verrucomicrobia	chitinase	3.2.1.14	GH18	Y	39% chitinase A1 *Bacillus circulans* ChiA1
UBA4506	Verrucomicrobia	chitinase	3.2.1.14	GH18	Y	39% chitinase A1 *Bacillus circulans* ChiA1
Algoriphagus	Bacteroidetes	chitinase	3.2.1.14	GH18	N	36% chitinase A1 *Bacillus circulans* ChiA1
Leadbetterella	Bacteroidetes	chitinase	3.2.1.14	GH18	Y	24% endochitinase B *Emericella nidulans* ChiB
Cyclobacterium	Bacteroidetes	chitinase	3.2.1.14	GH18	Y	24% endochitinase B *Emericella nidulans* ChiB
JGIOTU-2	Cloacimonetes	chitinase	3.2.1.14	GH18	N	51% chitinase D *Bacillus circulans* ChiD; has C-term secretion system C-terminal sorting domain
NaphS2	Deltaproteobacteria	chitinase	3.2.1.14	GH19	Y	34% endochitinase *Solanum lycopersicum* CHI9
**lysozyme/muramidase** (3.2.1.17)						Hydrolysis of (1→4)-β-linkages between N-acetyl-D-glucosamine residues in chitodextrins.
Arctic95D-9	Verrucomicrobia	lysozyme	3.2.1.17	GH25	Y	25% lysozyme M1 *Streptomyces globisporus* Acm
SW10	Verrucomicrobia	lysozyme	3.2.1.17	GH25	n.d.[b]	-

Haloferula	Verrucomicrobia	lysozyme	3.2.1.17	GH25	Y	27% autolytic lysozyme *Clostridium acetobutylicum* Lyc
**chitosanase** (3.2.1.132)						Endohydrolysis of β-(1→4)-linkages between D-glucosamine residues in a partly acetylated chitosan.
Arctic95D-9	Verrucomicrobia	chitosanase	3.2.1.132	GH75	Y	27% endo-chitosanase C *Aspergillus oryzae* CsnC
SW10	Verrucomicrobia	chitosanase	3.2.1.132	GH75	N	27% endo-chitosanase C *Aspergillus oryzae* CsnC
Haloferula	Verrucomicrobia	chitosanase	3.2.1.132	GH75	N	-
**Cellulose degradation**						
**endoglucanase** (3.2.1.4)						Endohydrolysis of (1→4)-β-D-glucosidic linkages in β-D-glucans (e.g., cellulose, lichenin).
Polaribacter	Bacteroidetes	endoglucanase + cellulose-binding domain (CBM family 6)	3.2.1.4	GH5	Y	25% endoglucanase C *Hungateiclostridium thermocellum* CelC
UBA2664	Bacteroidetes	endoglucanase	3.2.1.4	GH5	Y	40% endoglucanase D *Clostridium cellulolyticum* CelCCD
Desulfobacterium	Deltaproteobacteria	endoglucanase	3.2.1.4	GH5	Y	47% endoglucanase *Ralstonia solanacearum* Egl
**cellobiose phosphorylase** (2.4.1.20)						Catalyzes the phosphorolysis of cellobiose, yielding glucose 1-phosphate and glucose.
UBA4459	Bacteroidetes	cellobiose phosphorylase	2.4.1.20	GH36	N	62% cellobiose phosphorylase *Thermotoga neapolitana* CbpA

**Polysaccharide/ oligosaccharide degradation**						
**α-amylase** (3.2.1.1)						Endohydrolysis of (1→4)-α-D-glucosidic linkages in polysaccharides with at least three (1→4)-α-linked D-glucose units.
BACL24	Verrucomicrobia	α-amylase	3.2.1.1	GH13	Y	25% α-amylase 3 *Dictyoglomus thermophilum* AmyC
UBA4506	Verrucomicrobia	α-amylase	3.2.1.1	GH13	Y	25% α-amylase 3 *Dictyoglomus thermophilum* AmyC
Leadbetterella	Bacteroidetes	α-amylase	3.2.1.1	GH13	Y	23% α-amylase 3 *Dictyoglomus thermophilum* AmyC
Polaribacter	Bacteroidetes	α-amylase	3.2.1.1	GH13	Y	35% periplasmic α-amylase *Escherichia coli* MalS
UBA4459	Bacteroidetes	α-amylase	3.2.1.1	GH13	Y	25% α-amylase 3 *Dictyoglomus thermophilum* AmyC
Pseudomonas E	Gammaproteobacteria	α-amylase	3.2.1.1	GH13	Y	57% periplasmic α-amylase *Escherichia coli* MalS
Oligoflexus	Oligoflexia	α-amylase	3.2.1.1	GH13	N	23% α-amylase 3 *Dictyoglomus thermophilum* AmyC
Haloferula	Verrucomicrobia	α-amylase	3.2.1.1	GH57	N	-
Cyclobacterium	Bacteroidetes	α-amylase	3.2.1.1	GH57	N	23% α-amylase *Pyrococcus abyssi* AmyA
**oligo-1,6-glucosidase** (3.2.1.10)						Hydrolysis of (1→6)-α-D-glucosidic linkages in some oligosaccharides produced from starch/glycogen by α-amylase.
Fabibacter	Bacteroidetes	oligo-1,6-glucosidase	3.2.1.10	GH13	Y	57% oligo-1,6-glucosidase *Geobacillus thermoglucosidasius* MalL

Yoonia	Alphaproteobacteria	oligo-1,6-glucosidase	3.2.1.10	GH13	N	37% oligo-1,6-glucosidase *Bacillus coagulans* MalL
Pseudomonas E	Gammaproteobacteria	oligo-1,6-glucosidase	3.2.1.10	GH13	N	35% oligo-1,6-glucosidase *Bacillus subtilis* MalL
Izimaplasma	Tenericutes	oligo-1,6-glucosidase	3.2.1.10	GH13	N	44% oligo-1,6-glucosidase *Bacillus cereus* MalL
**neopullalanase** (3.2.1.135)						Hydrolysis of pullulan to panose (6-α-D-glucosylmaltose).
UBA4459	Bacteroidetes	neopullulanase	3.2.1.135	GH13	Y	47% neopullulanase *Bacteroides thetaiotaomicron* SusA
JGIOTU-2	Cloacimonetes	neopullulanase	3.2.1.135	GH13	N	36% neopullulanase *Thermoactinomyces vulgaris* TvaII
**pullalanase** (3.2.1.41)						Hydrolysis of (1→6)-α-D-glucosidic linkages in pullulan, amylopectin, glycogen; generates linear polymers of 1→6-linked maltotriose units.
JGIOTU-2	Cloacimonetes	pullulanase	3.2.1.41	GH13	N	39% pullulanase *Thermotoga maritima* PulA
**β-glucanase (endo-β-1,3-1,4 glucanase)** (3.2.1.73)						Hydrolysis of (1→4)-β-D-glucosidic linkages in β-D-glucans containing (1→3)- and (1→4)-bonds (e.g., laminarin).
BACL24	Verrucomicrobia	β-glucanase	3.2.1.73	GH16	N	47% β-glucanase *Rhodothermus marinus* BglA
BACL24	Verrucomicrobia	β-glucanase	3.2.1.73	GH16	Y	40% β-glucanase *Rhodothermus marinus* BglA
Arctic95D-9	Verrucomicrobia	β-glucanase	3.2.1.73	GH16	Y	34% β-glucanase *Rhodothermus marinus* BglA
SW10	Verrucomicrobia	β-glucanase	3.2.1.73	GH16	Y	39% β-glucanase *Rhodothermus marinus* BglA

Algoriphagus	Bacteroidetes	β-glucanase	3.2.1.73	GH16	Y	38% β-glucanase *Rhodothermus marinus* BglA
Leadbetterella	Bacteroidetes	β-glucanase	3.2.1.73	GH16	Y	43% β-glucanase *Rhodothermus marinus* BglA
Saprospiraceae sp.	Bacteroidetes	β-glucanase	3.2.1.73	GH16	Y	45% β-glucanase *Rhodothermus marinus* BglA
Saprospiraceae sp.	Bacteroidetes	β-glucanase	3.2.1.73	GH16	Y	55% β-glucanase *Rhodothermus marinus* BglA
Polaribacter	Bacteroidetes	β-glucanase	3.2.1.73	GH16	Y	33% β-glucanase *Rhodothermus marinus* BglA
Crocinitomix	Bacteroidetes	β-glucanase	3.2.1.73	GH16	Y	44% β-glucanase *Rhodothermus marinus* BglA
Fabibacter	Bacteroidetes	β-glucanase	3.2.1.73	GH16	Y	41% β-glucanase *Rhodothermus marinus* BglA
MAG-120531	Bacteroidetes	β-glucanase	3.2.1.73	GH16	N	40% β-glucanase *Rhodothermus marinus* BglA
UBA4459	Bacteroidetes	β-glucanase	3.2.1.73	GH16	Y	42% β-glucanase *Rhodothermus marinus* BglA
UBA2664	Bacteroidetes	β-glucanase	3.2.1.73	GH16	Y	46% β-glucanase *Rhodothermus marinus* BglA
HTCC2207	Gammaproteobacteria	β-glucanase	3.2.1.73	GH16	Y	39% β-glucanase *Rhodothermus marinus* BglA
**exo-1,3-glucanase** (3.2.1.58)						Successive hydrolysis of β-D-glucose units from the non-reducing ends of (1→3)-β-D-glucans, releasing α-glucose (e.g., laminarin).
Polaribacter	Bacteroidetes	exo-1,3-glucanase	3.2.1.58	GH17	Y	28% glucan 1,3-β-glucosidase *Arthroderma benhamiae*

Polaribacter	Bacteroidetes	exo-1,3-glucanase	3.2.1.58	GH17	N	27% glucan 1,3-β-glucosidase *Saccharomyces cerevisiae*
UBA4459	Bacteroidetes	exo-1,3-glucanase	3.2.1.58	GH17	Y	26% glucan 1,3-β-glucosidase *Arthroderma benhamiae*
UBA4459	Bacteroidetes	exo-1,3-glucanase	3.2.1.58	GH17	N	31% glucan 1,3-β-glucosidase *Saccharomyces cerevisiae*
**β-1,3(4)-glucanase** (3.2.1.6)						Endohydrolysis of (1→3)- or (1→4)-linkages in β-D-glucans when the glucose residue whose reducing group is involved in the linkage to be hydrolyzed is itself substituted at C-3 (e.g., laminarin, lichenin, cereal D-glucans).
JGIOTU-2	Cloacimonetes	β-1,3(4)-glucanase	3.2.1.6	GH64	Y	-
**xyloglucan endo-1,4-β-glucanase** (3.2.1.151)						Endohydrolysis of 1,4-β-D-glucosidic linkages in xyloglucan, generating xyloglucan oligosaccharides.
UBA4459	Bacteroidetes	xyloglucan endo-1,4-β-glucanase	3.2.1.151	GH9	Y	40% xyloglucan-specific endo-β-1,4-glucanase *Bacteroides ovatus* BoGH9A
**β-1,6-glucanase** (3.2.1.75)						
Leadbetterella	Bacteroidetes	endo-1,6-β-D-glucanase	3.2.1.75	GH30	Y	34% endo-1,6-β-D-glucanase *Neurospora crassa* Neg-1
**β-glucosidase** (3.2.1.21)						Hydrolysis of terminal, non-reducing beta-D-glucosyl residues with release of β-D-glucose (e.g., cellobiose).
Haloferula	Verrucomicrobia	β-glucosidase	3.2.1.21	GH1	N	33% β-glucosidase 18 *Oryza sativa* subsp. *japonica* BGLU18
Loktanella	Alphaproteobacteria	β-glucosidase	3.2.1.21	GH1	Y	43% β-glucosidase *Agrobacterium* sp. Abg

BACL24	Verrucomicrobia	β-glucosidase	3.2.2.21	GH3	Y	34% periplasmic β-glucosidase *Escherichia coli* BglX
UBA4506	Verrucomicrobia	β-glucosidase	3.2.2.21	GH3	Y	34% periplasmic β-glucosidase *Escherichia coli* BglX
Crocinitomix	Bacteroidetes	β-glucosidase	3.2.2.21	GH3	Y	40% β-glucosidase *Bacteroides ovatus* BoGH3B
Saprospiraceae sp.	Bacteroidetes	β-glucosidase	3.2.2.21	GH3	Y	42% periplasmic β-glucosidase *Salmonella typhimurium* BglX
Polaribacter	Bacteroidetes	β-glucosidase	3.2.2.21	GH3	Y	32% β-glucosidase *Bacteroides ovatus* BoGH3B
Nisaea	Alphaproteobacteria	β-glucosidase	3.2.2.21	GH3	N	40% periplasmic β-glucosidase *Escherichia coli* BglX
**α-glucosidase** (3.2.1.20)						Hydrolysis of terminal, non-reducing (1→4)-linked α-D-glucose residues with release of α-D-glucose.
Haloferula	Verrucomicrobia	α-glucosidase	3.2.1.20	GH13	N	26% maltase 1 *Drosophila virilis* Mal-B1
MAG-120531	Bacteroidetes	α-glucosidase	3.2.1.20	GH13	Y	31% maltase 1 *Drosophila virilis* Mal-B1
BACL25	Gammaproteobacteria	α-glucosidase	3.2.1.20	GH13	N	36% maltase 1 *Drosophila virilis* Mal-B1
SW10	Verrucomicrobia	α-glucosidase	3.2.1.20	GH31	N	39% α-glucosidase 2 *Bacillus thermoamyloliquefaciens*
Fabibacter	Bacteroidetes	α-glucosidase	3.2.1.20	GH31	N	41% α-glucosidase 2 *Bacillus thermoamyloliquefaciens*
Cyclobacterium	Bacteroidetes	α-glucosidase	3.2.1.20	GH31	N	40% α-glucosidase 2 *Bacillus thermoamyloliquefaciens*
**levanase (**3.2.1.65)						Random hydrolysis of (2→6)-β-D-fructofuranosidic linkages in (2→6)-β-D-

						fructans (levans) containing more than 3 fructose units.
Cyclobacterium	Bacteroidetes	levanase	3.2.1.65	GH32	Y	41% levanase *Bacillus subtilis* SacC
Fabibacter	Bacteroidetes	levanase	3.2.1.65	GH32	Y	50% levanase *Bacillus subtilis* SacC
MAG-120531	Bacteroidetes	levanase	3.2.1.65	GH32	Y	46% levanase *Bacillus subtilis* SacC
**β-xylanase (endo-1,4-β-xylanase)** (3.2.1.8)						Endohydrolysis of (1→4)-β-D-xylosidic linkages in xylans.
BACL24	Verrucomicrobia	endo-1,4-β-xylanase	3.2.1.8	GH10	Y	25% endo-1,4-β-xylanase A *Streptomyces lividans* XlnA
UBA4506	Verrucomicrobia	endo-1,4-β-xylanase	3.2.1.8	GH10	Y	27% endo-1,4-β-xylanase B *Thermotoga neapolitana* XynB
Algoriphagus	Bacteroidetes	endo-1,4-β-xylanase	3.2.1.8	GH10	Y	27% endo-1,4-β-xylanase B *Thermotoga neapolitana* XynB
Polaribacter	Bacteroidetes	endo-1,4-β-xylanase	3.2.1.8	GH10	Y	28% endo-1,4-β-xylanase B *Thermotoga neapolitana* XynB
Cyclobacterium	Bacteroidetes	endo-1,4-β-xylanase	3.2.1.8	GH10	Y	27% endo-1,4-β-xylanase C *Neosartorya fumigata* XlnC
Halioglobus	Gammaproteobacteria	endo-1,4-β-xylanase	3.2.1.8	GH10	Y	38% endo-1,4-β-xylanase *Agaricus bisporus* XlnA
Algoriphagus	Bacteroidetes	endo-1,4-β-xylanase	3.2.1.8	GH39	Y	22% endo-1,4-β-xylanase 2 *Magnaporthe oryzae* XYL2
Cyclobacterium	Bacteroidetes	endo-1,4-β-xylanase	3.2.1.8	GH43	Y	-
**β-xylosidase** (3.2.1.37)						Hydrolysis of (1→4)-β-D-xylans, to remove successive D-xylose residues from the non-reducing termini.

UBA4459	Bacteroidetes	β-xylosidase	3.2.1.37	GH3	Y	31% xylan 1,4-β-xylosidase *Prevotella ruminicola* Xyl3A
Algoriphagus	Bacteroidetes	β-xylosidase	3.2.1.37	GH39	Y	21% β-xylosidase *Thermoanaerobacterium saccharolyticum* XynB
Polaribacter	Bacteroidetes	β-xylosidase	3.2.1.37	GH39	Y	24% β-xylosidase *Geobacillus stearothermophilus* XynB
UBA4459	Bacteroidetes	β-xylosidase	3.2.1.37	GH39	Y	28% β-xylosidase *Thermoanaerobacterium saccharolyticum* XynB
BACL24	Verrucomicrobia	β-xylosidase	3.2.1.37	GH39	N	33% β-xylosidase *Geobacillus stearothermophilus* XynB
**α-xylosidase** (3.2.1.177)						Hydrolysis of terminal, non-reducing α-D-xylose residues with release of α-D-xylose.
Algoriphagus	Bacteroidetes	α-xylosidase	3.2.1.177	GH31	Y	27% α-xylosidase *Saccharolobus solfataricus* XylS
Cyclobacterium	Bacteroidetes	α-xylosidase	3.2.1.177	GH31	Y	25% α-xylosidase *Saccharolobus solfataricus* XylS
Halioglobus	Gammaproteobacteria	α-xylosidase	3.2.1.177	GH31	N	32% α-xylosidase *Escherichia coli* YicI
SCGC-AAA027-K21	Betaproteobacteria	α-xylosidase	3.2.1.177	GH31	N	25% α-xylosidase *Escherichia coli* YicI
**arabinogalactan endo-1,4-β-galactanase** (3.2.1.89)						Hydrolysis of (1→4)-β-D-galactosidic linkages in type I arabinogalactans (e.g., pectin side-chains), generating oligosaccharides.
Polaribacter	Bacteroidetes	arabinogalactan endo-1,4-β-galactanase	3.2.1.89	GH53	N	27% arabinogalactan endo-β-1,4-galactanase *Bacillus licheniformis* GanB
JGIOTU-2	Cloacimonetes	arabinogalactan endo-1,4-β-galactanase	3.2.1.89	GH53	Y	32% arabinogalactan endo-β-1,4-galactanase *Bacillus licheniformis* GanB

**β-galactosidase** (3.2.1.23)						Hydrolysis of terminal non-reducing β-D-galactose residues in β-D-galactosides.
SW10	Verrucomicrobia	β-galactosidase	3.2.1.23	GH2	Y	26% β-galactosidase *Thermotoga maritima* LacZ
Izimaplasma	Tenericutes	β-galactosidase	3.2.1.23	GH2	N	44% β-galactosidase *Thermoanaerobacterium thermosulfurigenes* LacZ
34-128	Atribacteria	β-galactosidase	3.2.1.23	GH2	N	57% β-galactosidase *Thermoanaerobacter pseudethanolicus* LacZ
Cyclobacterium	Bacteroidetes	β-galactosidase	3.2.1.23	GH42	Y	-
HTCC2207	Gammaproteobacteria	β-galactosidase	3.2.1.23	GH42	N	47% β-galactosidase Thermus thermophilus BgaA
**α-galactosidase** (3.2.1.22)						Hydrolysis of terminal, non-reducing α-D-galactose residues in α-D-galactosides, including galactose oligosaccharides, galactomannans and galactolipids
BACL24	Verrucomicrobia	α-galactosidase	3.2.1.22	GH36	N	.-
Haloferula	Verrucomicrobia	α-galactosidase	3.2.1.22	GH36	N	-
HTCC2207	Gammaproteobacteria	α-galactosidase	3.2.1.22	GH36	N	39% α-galactosidase *Escherichia coli* RafA
Izimaplasma	Tenericutes	α-galactosidase	3.2.1.22	GH36	N	43% α-galactosidase AgaA *Geobacillus stearothermophilus* AgaA
34-128	Atribacteria	α-galactosidase	3.2.1.22	GH36	N	37% α-galactosidase AgaA *Geobacillus stearothermophilus* AgaA
**κ-carrageenase** (3.2.1.83)						Endohydrolysis of (1→4)-β-D-linkages between D-galactose 4-sulfate and 3,6-anhydro-D-galactose in κ-carrageenans.

Crocinitomix	Bacteroidetes	κ-carrageenase	3.2.1.83	GH16	Y	25% κ-carrageenase *Pseudoalteromonas carrageenovora* CgkA
Gimesia	Planctomycetes	κ-carrageenase	3.2.1.83	GH16	N	42% κ-carrageenase *Pseudoalteromonas carrageenovora* CgkA
**β-mannanase (mannan endo-1,4-β-mannosidase)** (3.2.1.78)						Hydrolysis of (1→4)-β-D-mannosidic linkages in mannans, galactomannans, and glucomannans.
BACL24	Verrucomicrobia	β-mannanase	3.2.1.78	GH5	Y	-
Leadbetterella	Bacteroidetes	β-mannanase	3.2.1.78	GH5	Y	24% mannan endo-1,4-β-mannosidase A *Podospora anserina*
Gimesia	Planctomycetes	β-mannanase	3.2.1.78	GH5	Y	-
Halioglobus	Gammaproteobacteria	β-mannanase	3.2.1.78	GH5	Y	22% mannan endo-1,4-β-mannosidase 1 *Oryza sativa* subsp. *japonica*
BACL24	Verrucomicrobia	β-mannanase	3.2.1.78	GH26	N	-
UBA4506	Verrucomicrobia	β-mannanase	3.2.1.78	GH26	N	36% mannan endo-1,4-β-mannosidase A and B *Bacillus mannanilyticus*
Polaribacter	Bacteroidetes	β-mannanase	3.2.1.78	GH26	Y	33% mannan endo-1,4-β-mannosidase *Cellvibrio japonicus* ManA
**β-mannosidase** (3.2.1.25)						Hydrolysis of terminal, non-reducing β-D-mannosyl residues in β-D-mannosides with release of β-D-mannose.
BACL24	Verrucomicrobia	β-mannosidase	3.2.1.25	GH2	Y	24% β-mannosidase B *Emericella nidulans* MndB
Polaribacter	Bacteroidetes	β-mannosidase	3.2.1.25	GH2	Y	30% β-mannosidase B *Emericella nidulans* MndB

UBA4459	Bacteroidetes	β-mannosidase	3.2.1.25	GH2	Y	33% β-mannosidase *Capra hircus* MANBA
**α-mannosidase** (3.2.1.24)						Hydrolysis of terminal, non-reducing α-D-mannose residues in α-D-mannosides
UBA4459	Bacteroidetes	α-mannosidase + carbohydrate-binding domain	3.2.1.24	GH38	Y	-
Synechococcus	Cyanobacteria	α-mannosidase	3.2.1.24	GH38	N	34% α-mannosidase G *Dictyostelium discoideum* ManG
**L-arabinofuranosidase** (3.2.1.55)						Hydrolysis of terminal non-reducing α-L-arabinofuranoside residues in α-L-arabinosides.
SW10	Verrucomicrobia	sulfatase + α-L-arabinofuranosidase	3.2.1.55	GH43	Y	29% extracellular exo-α-(1→5)-L-arabinofuranosidase *Streptomyces avermitilis* Araf43A
**α-L-fucosidase** (3.2.1.51)						Releases L-fucose from α-L-fucosides
BACL24	Verrucomicrobia	α-L-fucosidase	3.2.1.51	GH29	Y	29% α-L-fucosidase *Dictyostelium discoideum* AlfA
UBA4506	Verrucomicrobia	α-L-fucosidase	3.2.1.51	GH29	Y	33% α-L-fucosidase *Branchiostoma floridae*
Leadbetterella	Bacteroidetes	α-L-fucosidase	3.2.1.51	GH29	Y	28% α-L-fucosidase 1 *Arabidopsis thaliana* FUC1
Cyclobacterium	Bacteroidetes	α-L-fucosidase	3.2.1.51	GH29	Y	40% α-L-fucosidase 1 *Arabidopsis thaliana* FUC1
Fabibacter	Bacteroidetes	α-L-fucosidase	3.2.1.51	GH29	Y	43% α-L-fucosidase *Dictyostelium discoideum* AlfA
UBA4459	Bacteroidetes	α-L-fucosidase	3.2.1.51	GH29	Y	29% α-L-fucosidase *Dictyostelium discoideum* AlfA

Halioglobus	Gammaproteobacteria	α-L-fucosidase	3.2.1.51	GH29	Y	33% α-L-fucosidase *Bos taurus* FUCA1
**α-L-rhamnosidase** (3.2.1.40)						Hydrolysis of terminal non-reducing α-L-rhamnose residues in α-L-rhamnosides (e.g., pectin side-chains).
BACL24	Verrucomicrobia	α-L-rhamnosidase	3.2.1.40	-	Y	-
SW10	Verrucomicrobia	α-L-rhamnosidase	3.2.1.40	-	Y	24% α-L-rhamnosidase *Streptomyces avermitilis*
Haloferula	Verrucomicrobia	α-L-rhamnosidase	3.2.1.40	-	Y	32% α-L-rhamnosidase *Streptomyces avermitilis*
Cyclobacterium	Bacteroidetes	α-L-rhamnosidase	3.2.1.40	-	Y	40% α-L-rhamnosidase *Streptomyces avermitilis*
UBA2664	Bacteroidetes	α-L-rhamnosidase	3.2.1.40	-	Y	20% % α-L-rhamnosidase *Formosa agariphila*
**rhamnogalacturonan acetylesterase** (3.1.1.86)						Hydrolytic cleavage of 2-O-acetyl- or 3-O-acetyl groups of alpha-D-galacturonic acid in rhamnogalacturonan I.
Cyclobacterium	Bacteroidetes	rhamnogalacturonan acetylesterase	3.1.1.86	-	Y	41% rhamnogalacturonan acetylesterase *Bacillus subtilis* RhgT
**Unknown**						
Cyclobacterium	Bacteroidetes	GH2 family glycoside hydrolase	-	GH2	Y	-
Cyclobacterium	Bacteroidetes	GH9 family glycoside hydrolase	-	GH9	N	-
Desulfobacterium	Deltaproteobacteria	GH13 family glycoside hydrolase	-	GH13	N	-

Gimesia	Planctomycetes	GH13 family glycoside hydrolase	-	GH13	Y	-
Arctic95D-9	Verrucomicrobia	GH92 family glycoside hydrolase	-	GH92	Y	-
Polaribacter	Bacteroidetes	GH92 family glycoside hydrolase	-	GH92	Y	-
Crocinitomix	Bacteroidetes	GH92 family glycoside hydrolase	-	GH92	Y	-
Cyclobacterium	Bacteroidetes	GH92 family glycoside hydrolase	-	GH92	Y	-
MAG-120531	Bacteroidetes	α-mannosidase-like protein	-	GH92	Y	-
UBA4459	Bacteroidetes	GH92 family glycoside hydrolase	-	GH92	Y	-
Cyclobacterium	Bacteroidetes	NodB polysaccharide deacetylase domain + Galactose-binding-like domain	3.5.1.-	-	Y	signal peptide + NodB polysaccharide deacetylase domain + Galactose-binding-like domain
**Glycosphingolipid degradation**						
**glucosylceramidase** (3.2.1.45)						Releases glucose from glucosylceramide (D-glucosyl-N-acylsphingosine), a glycosphingolipid.
Saprospiraceae sp.	Bacteroidetes	glucosylceramidase	3.2.1.45	GH30	Y	33% lysosomal acid glucosylceramidase *Homo sapiens* Gba
Polaribacter	Bacteroidetes	glucosylceramidase	3.2.1.45	GH30	N	32% lysosomal acid glucosylceramidase *Sus scrofa* Gba
UBA4459	Bacteroidetes	glucosylceramidase	3.2.1.45	GH30	Y	35% lysosomal acid glucosylceramidase *Mus musculus* Gba

Izimaplasma	Tenericutes	glucosylceramidase	3.2.1.45	GH30	N	36% lysosomal acid glucosylceramidase *Homo sapiens* Gba
Synechococcus	Cyanobacteria	glucosylceramidase	3.2.1.45	GH116	N	34% non-lysosomal glucosylceramidase *Rattus norvegicus* Gba2
**Glycoprotein degradation**						Cleaves an entire glycan from a glycoprotein.
**peptide-N-glycolase** (3.5.1.52)						
Nonlabens	Bacteroidetes	peptide-N-glycolase	3.5.1.52	-	N	51% Peptide-N^4-(N-acetyl-β-D-glucosaminyl)asparagine amidase F *Elizabethkingia miricol*a Ngl
**mannosylglycoprotein endo-β-mannosidase** (3.2.1.152)						
BACL24	Verrucomicrobia	β-mannosidase	3.2.1.25	GH2	Y	28% mannosylglycoprotein endo-β-mannosidase *Lilium longiflorum*
**Glycosaminoglycan degradation**						
**chondroitin disaccharide hydrolase** (3.2.1.180)						Releases 4-deoxy-4,5-didehydro D-glucuronic acid or 4-deoxy-4,5-didehydro L-iduronic acid from chondroitin disaccharides, hyaluronan disaccharides and heparin disaccharides and cleaves both glycosidic (1→3) and (1→4) bonds.
BACL24	Verrucomicrobia	chondroitin disaccharide hydrolase	3.2.1.180	GH88	N	27% unsaturated glucuronyl hydrolase *Bacillus* sp. Ugl
UBA4506	Verrucomicrobia	chondroitin disaccharide hydrolase	3.2.1.180	GH88	N	27% unsaturated glucuronyl hydrolase *Bacillus sp.* Ugl

Leadbetterella	Bacteroidetes	chondroitin disaccharide hydrolase	3.2.1.180	GH88	Y	31% unsaturated chondroitin disaccharide hydrolase *Streptococcus pneumoniae* Ugl
Cyclobacterium	Bacteroidetes	chondroitin disaccharide hydrolase	3.2.1.180	GH88	N	22% unsaturated chondroitin disaccharide hydrolase *Streptococcus pneumoniae* Ugl
UBA2664	Bacteroidetes	chondroitin disaccharide hydrolase	3.2.1.180	GH88	Y	29% unsaturated chondroitin disaccharide hydrolase *Streptococcus pneumoniae* Ugl
**Cellulose biosynthesis – aggregation**						
UBA4506	Verrucomicrobia	endoglucanase	3.2.1.4	GH8	Y	33% minor endoglucanase Y *Dickeya dadantii* CelY
Hydrogenophaga	Betaproteobacteria	endoglucanase	3.2.1.4	GH8	Y	46% endoglucanase *Salmonella typhi* BcsZ
Desulfocapsa	Deltaproteobacteria	endoglucanase	3.2.1.4	GH8	Y	34% minor endoglucanase Y *Dickeya dadantii* CelY

[a] Sequence identity based on closest match to characterized protein, as determined by ExPASy BLAST database. [b] n.d., not determined – presence of signal peptide could not be determined, due to incompleteness of available sequence. GH, glycoside hydrolase.

Table S7 Hydrogenases of the abundant OTUs in Ace Lake.

OTU	Hydrogenase	Proposed function	Location
Desulfobacterium	[NiFe] Group 1b	Periplasmic H_2-uptake hydrogenase; liberates and transfers electrons via cytochromes to terminal reductases for sulfate and fumarate respiration.	Periplasm
Desulfocapsa	[NiFe] Group 1c	Membrane-bound H_2-uptake hydrogenase; H_2 oxidation liberates electrons, possibly for sulfur oxidation/respiration.	Membrane-bound
Desulfocapsa	[NiFe] Group 3b	Directly couples oxidation of NADPH to H_2 evolution (also possible sulfhydrogenase, in which case sulfide released).	Cytosol
Gimesia	[NiFe] Group 3b	Directly couples oxidation of NADPH to H_2 evolution (also possible sulfhydrogenase, in which case sulfide released).	Cytosol
Bacteroidales UBA4459	[FeFe] Group A3	Bifurcating/confurcating hydrogenase linked to fermentation: electron transfer from NADH and reduced ferredoxin to generate H_2.	Cytosol
Bacteroidales UBA4459	[NiFe] Group 3c	Heterodisulfide-linked, bifurcates electrons from H_2 to heterodisulfide and ferredoxin (oxidized).	Cytosol
Bacteroidales UBA4459	[NiFe] Group 3d	Directly interconverts electrons between H_2 and NAD depending on redox state.	Cytosol
Synechococcus	[NiFe] Group 3d	Directly interconverts electrons between H_2 and NAD depending on redox state; may favor H_2 production, and be used during dark, anaerobic fermentation.	Cytosol
Desulfatiglanales NaphS2	[NiFe] Group 1b	Periplasmic H_2-uptake hydrogenase; liberates and transfers electrons via cytochromes to terminal reductases for sulfate and fumarate respiration.	Periplasm
Desulfatiglanales NaphS2	[NiFe] Group 3b	Directly couples oxidation of NADPH to H_2 evolution (also possible sulfhydrogenase, in which case sulfide released).	Cytosol
Atribacteria 34-128	[NiFe] Group 4d	Membrane-bound, respiratory H_2-evolving, generates sodium-motive force via Mrp antiporter complex.	Membrane-bound
Atribacteria 34-128	[FeFe] Group A3	Bifurcating/confurcating hydrogenase linked to fermentation: electron transfer from NADH and reduced ferredoxin to generate H_2.	Cytosol
Cloacimonetes JGIOTU-2	[NiFe] Group 3c	Heterodisulfide-linked, bifurcates electrons from H_2 to heterodisulfide and	Cytosol

		ferredoxin (oxidized).	
Cloacimonetes JGIOTU-2	[NiFe] Group 4g	Membrane-bound, respiratory H_2-evolving, generates sodium-motive force via Mrp antiporter module.	Membrane-bound
Izimaplasma	[FeFe] Group A3	Bifurcating/confurcating hydrogenase linked to fermentation: electron transfer from NADH and reduced ferredoxin to generate H_2.	Cytosol
Methanomicrobiaceae 1	[NiFe] Group 3a	F_{420}-dependent; directly couples oxidation of H_2 to reduction of F_{420} during methanogenesis.	Cytosol

F_{420}, 8-hydroxy-5-deazaflavin (coenzyme).

Table S8 Abundant OTUs in Ace Lake.

<table>
<tr><th>Abundant phyla/classes in Ace Lake</th><th>OTUs</th><th>Original taxonomic classification[b]</th><th>Reference genome/assembly (Assembly accession ID)</th><th>% ANI (% alignment fraction)</th><th>16S/18S SSU % identity</th><th>MetaBAT MAG matches</th></tr>
<tr><td rowspan="4">Actinobacteria</td><td>Aquiluna</td><td>Candidatus Aquiluna sp. IMCC13023</td><td>Candidatus Aquiluna sp. IMCC13023 (GCF_000257665.1)</td><td>87 (83)</td><td>100
99
99</td><td>Bin802 s_Aquiluna sp1
Bin842 s_Aquiluna sp1
Bin1781 s_Aquiluna sp1</td></tr>
<tr><td rowspan="3">Microbacteriaceae BACL25</td><td>Mesorhizobium sp. F7</td><td>Mesorhizobium sp. F7 (GCF_000798645.1)</td><td>72 (29)</td><td>NM</td><td rowspan="3">Bin1187 s_BACL25 sp1
Bin1172 s_BACL25 sp1
Bin1399 s_BACL25 sp1
Bin534 s_BACL25 sp1</td></tr>
<tr><td>Microcella sp. HL-107</td><td>Microcella sp. HL-107 (GCF_002813345.1)</td><td>72 (27)</td><td>96
96</td></tr>
<tr><td>Yonghaparkia sp. Root332</td><td>Yonghaparkia sp. Root332 (GCF_001425665.1)</td><td>72 (31)</td><td>NM</td></tr>
<tr><td rowspan="3">Alphaproteobacteria (Proteobacteria)</td><td>Loktanella</td><td>Loktanella salsilacus</td><td>Loktanella salsilacus DSM 16199 (GCF_900114485.1)</td><td>84 (77)</td><td>NM</td><td>Bin864 s_Loktanella salsilacus</td></tr>
<tr><td>Nisaea</td><td>alpha proteobacterium BAL199</td><td>alpha proteobacterium BAL199 (GCF_000171835.1)</td><td>80 (57)</td><td>98
97</td><td>Bin1427 g_BAL199
Bin283 g_BAL199</td></tr>
<tr><td>Pelagibacter</td><td>Candidatus Pelagibacter ubique</td><td>Candidatus Pelagibacter ubique HIMB083 (GCF_000504225.1)</td><td>77 (68)</td><td>99
99
92</td><td>Bin1939 s_Pelagibacter ubique
Bin2016 s_Pelagibacter ubique
Bin1535 s_Pelagibacter ubique
Bin978 s_Pelagibacter ubique
Bin1105 s_Pelagibacter ubique
Bin1323 s_Pelagibacter ubique</td></tr>
</table>

		Candidatus *Pelagibacter* sp. IMCC9063	Candidatus *Pelagibacter* sp. IMCC9063 (GCF_000195085.1)	90 (90)	100<br>100	Bin887 s_*Pelagibacter ubique*<br>Bin2004 s_*Pelagibacter ubique*<br>Bin1518 s_*Pelagibacter ubique*<br>Bin363 s_*Pelagibacter ubique*<br>Bin1541 g_*Pelagibacter*<br>Bin1782 g_*Pelagibacter*<br>Bin1123 g_*Pelagibacter*<br>Bin1666 g_*Pelagibacter*<br>Bin1485 g_IMCC9063<br>Bin1036 g_IMCC9063
	Yoonia	*Yoonia vestfoldensis*	*Yoonia vestfoldensis* SKA53 (GCF_000152785.1)	93 (89)	100	Bin1729 s_*Yoonia vestfoldensis*
			Yoonia vestfoldensis DSM 16212 (GCF_000382265.1)	86 (77)	99	
Atribacteria	Atribacteria 34-128	unclassified Atribacteria, Atribacteria bacterium JGI 0000014-F07	Atribacteria bacterium 34_128 (GCA_001509285.1)	81 (18)	NA	Bin894 g_34-128<br>Bin1182 g_34-128<br>Bin866 g_34-128<br>Bin2083 g_34-128<br>Bin1876 p__Firmicutes
Bacteroidetes	*Algoriphagus*	*Algoriphagus antarcticus*	*Algoriphagus antarcticus* DSM 15986 (GCF_002150685.1)	78 (46)	NM	Bin1943 g_*Algoriphagus*
	Leadbetterella	Cytophagales bacterium TFI 002	Cytophagales bacterium TFI 002 (NZ_LT907983.1)	71 (19)	91	Bin277 g_*Leadbetterella*
	Saprospiraceae sp.	*Phaeodactylibacter xiamenensis*	*Phaeodactylibacter xiamenensis* KD52 (GCF_000759025.1)	69 (6)	NM	Bin420 f_Saprospiraceae
	Bacteroidales UBA4459	*Lentimicrobium saccharophilum*	*Lentimicrobium saccharophilum* TBC1 (GCF_001192835.1)	70 (11)	90<br>89<br>89	Bin1394 g_ UBA4459

					89<br>88<br>88	
	Crocinitomix	*Crocinitomix catalasitica*	*Crocinitomix catalasitica* ATCC 23190 (GCF_000621625.1)	73 (34)	96	Bin223 g_*Crocinitomix*
	Cyclobacterium	*Cyclobacterium qasimii*	*Cyclobacterium qasimii* M12-11B (GCF_000427295.1)	86 (82)	99	Bin1381 g_*Cyclobacterium*
	Fabibacter	*Roseivirga spongicola*	*Roseivirga spongicola* UST030701-084 (GCF_001592965.1)	73 (47)	94	Bin155 s_*Fabibacter sp1*
	Flavobacteriaceae MAG-120531	*Sediminicola* sp. YIK13	*Sediminicola* sp. YIK13 (GCF_001430825.1)	72 (37)	NM	Bin1744 g_MAG-120531<br>Bin896 g_MAG-120531
	Nonlabens	*Nonlabens xylanidelens*,	*Nonlabens xylanidelens* DSM 16809 (GCF_002934445.1)	75 (47)	NM	Bin1375 g_*Nonlabens*<br>Bin690 s_*Nonlabens dokdonensis*
		Nonlabens dokdonensis	*Nonlabens dokdonensis* DSW-6 (GCF_000332115.1)	75 (43)	NM	
	Polaribacter	unclassified *Polaribacter*, *Polaribacter* sp. KT25b	*Polaribacter* sp. KT25b (NZ_LT629752.1)	84 (63)	NM	Bin1415 g_*Polaribacter*<br>Bin385 g_*Polaribacter*<br>Bin670 g_*Polaribacter*<br>Bin246 g_*Polaribacter*<br>Bin574 g_*Polaribacter*<br>Bin776 g_*Polaribacter*
	Balneolaceae UBA2664	*Rhodohalobacter halophilus*	*Rhodohalobacter halophilus* JZ3C29 (GCF_001715195.1)	72 (29)	94<br>94	Bin306 g_ UBA2664
Betaproteobacteria (Proteobacteria)	Burkholderiaceae MOLA814	Betaproteobacteria bacterium MOLA814	Betaproteobacteria bacterium MOLA814 (GCF_000496475.1)	98 (94)	100	Bin1173 g_RS62
	Burkholderiaceae SCGC-AAA027-K21	Beta proteobacterium MWH-P2sevCIIIb	Beta proteobacterium MWH-P2sevCIIIb (GCF_003003055.1)	72 (25)	98	Bin1507 g_SCGC-AAA027-K21
	Hydrogenophaga	*Hydrogenophaga crassostreae*,	*Hydrogenophaga crassostreae* LPB0072 (GCF_001640105.1)	79 (49)	NM	Bin22 g_*Hydrogenophaga*

		Hydrogenophaga taeniospiralis	*Hydrogenophaga taeniospiralis* NBRC 102512 (GCF_001592305.1)	79 (49)	NM	
	Methylophilaceae BACL14	Methylophilales bacterium HTCC2181	Methylophilales bacterium HTCC2181 (GCF_000168995.1)	82 (92)	99	Bin470 s_BACL14 sp1
Chlorobi	*Chlorobium*	*Chlorobium phaeovibrioides*	*Chlorobium phaeovibrioides* DSM 265 (NC_009337.1)	85 (85)	99	Bin1268 s_*Chlorobium phaeovibrioides*
Chlorophyta (Eukarya)	*Micromonas*†	*Micromonas commoda*,	*Micromonas commoda* (NC_013038.1 - NC_013054.1)	75 (6)	NM	Bin919 Unclassified<br>Bin1249 Unclassified<br>Bin1079 Unclassified<br>Bin282 Unclassified
		Micromonas pusilla	*Micromonas pusilla* CCMP1545 (GCF_000151265.2)	75 (7)	NM	
Cloacimonetes	Cloacimonetes JGIOTU-2	unclassified Cloacimonetes	Cloacimonetes bacterium JGI OTU-2 (GCF_000493905.1)	81 (22)	NA	Bin1703 s_JGIOTU-2 sp1<br>Bin1683 s_JGIOTU-2 sp1<br>Bin1264 s_JGIOTU-2 sp1<br>Bin2003 f_TCS61<br>Bin1346 g_TCS61
			Cloacimonetes bacterium TCS61 (GCA_001577125.1)	71 (5)	NA	
Cyanobacteria	*Synechococcus*	*Synechococcus* sp. SynAce01	*Synechococcus* sp. SynAce01 (NZ_CP018091.1)	99 (97)	100	Bin1724 g_*Cyanobium*
Deltaproteobacteria (Proteobacteria)	Desulfatiglanales NaphS2	delta proteobacterium NaphS2	delta proteobacterium NaphS2 (GCF_000179315.1)	75 (34)	97<br>93<br>93<br>93	Bin2047 g_NaphS2<br>Bin505 g_NaphS2<br>Bin1224 g_NaphS2
	Desulfobacterales S5133MH16	*Desulfosarcina* sp. BuS5	*Desulfosarcina* sp. BuS5 (GCF_000472805.1)	74 (31)	93	Bin1209 g_S5133MH16<br>Bin1110 g_S5133MH16<br>Bin1728 g_S5133MH16<br>Bin2047 g_NaphS2<br>Bin505 g_NaphS2
	Desulfobacterium	*Desulfobacterium vacuolatum*	*Desulfobacterium vacuolatum* DSM 3385 (GCF_900176365.1)	82 (41)	98<br>98	Bin703 g_*Desulfobacterium*<br>Bin1072 g_*Desulfobacterium*
	Desulfocapsa	*Desulfocapsa sulfexigens*	*Desulfocapsa sulfexigens* DSM	78 (63)	97	Bin20 s_*Desulfocapsa*

			10523 (NC_020304.1)		97<br>97	*sulfexigens*<br>Bin2043 s_*Desulfocapsa sulfexigens*<br>Bin134 s_*Desulfocapsa sulfexigens*
	Syntrophales UBA2210	*Syntrophus aciditrophicus*	*Syntrophus aciditrophicus* SB (NC_007759.1)	70 (17)	92<br>92	Bin2060 g_UBA2210<br>Bin962 s_UBA2210 sp1<br>Bin899 g_UBA6078
		Syntrophus gentianae	*Syntrophus gentianae* DSM 8423	71 (26)	92	
		Smithella sp. F21	*Smithella* sp. F21	71 (35)	NM	
		Smithella sp. SCADC	*Smithella* sp. SCADC	70 (20)	91<br>91<br>91	
Euryarchaeota (Archaea)	Methanomicrobiaceae 1	*Methanoplanus limicola*	*Methanoplanus limicola* DSM 2279 (GCF_000243255.1)	73 (27)	94	Bin1205 f_Methanomicrobiaceae<br>Bin2059 s_*Methanomicrobium mobile*<br>Bin1141 f_Methanomicrobiaceae
	Methanothrix A	*Methanosaeta harundinacea*	*Methanosaeta harundinacea* 6Ac (GCF_000235565.1)	73 (22)	NM	Bin23 g_*Methanothrix_A*
Gammaproteobacteria (Proteobacteria)	*Halioglobus*	*Halioglobus pacificus*	*Halioglobus pacificus* strain RR3-57 (GCF_001953075.1)	72 (37)	NM	Bin1377 g_*Halioglobus*
		Marine gamma proteobacterium HTCC2148	Marine gamma proteobacterium HTCC2148 (GCF_000156295.1)	73 (32)	NM	
	Porticoccaceae HTCC2207	gamma proteobacterium HTCC2207	gamma proteobacterium HTCC2207 (GCF_000153445.1)	75 (45)	97	Bin525 g_HTCC2207<br>Bin686 g_HTCC2207<br>Bin271 g_HTCC2207
	Pseudohongiellaceae *<br><br>Pseudohongiellaceae 1	*Pseudohongiella spirulinae*	*Pseudohongiella spirulinae* KCTC 32221 (GCF_001444425.1)	71 (18)	NM	Bin706 - g_OM182<br>Bin2107 - s_OM182 sp1

	Pseudohongiellaceae 2					
	Pseudomonas E	unclassified *Pseudomonas*, *Pseudomonas alcaliphila*,	*Pseudomonas alcaliphila* JCM 10630 (GCF_900101755.1)	92 (82)	NM	Bin911 s_*Pseudomonas_E alcaliphila*
		unclassified *Pseudomonas*, *Pseudomonas pseudoalcaligenes*	*Pseudomonas pseudoalcaligenes* CECT 5344 (GCF_000297075.2)	96 (78)	NM	
Oligoflexia (Proteobacteria)	Oligoflexus	Pseudobacteriovorax antillogorgiicola RKEM611	Pseudobacteriovorax antillogorgiicola (GCF_900177345.1)	70(8)	NM	Bin927 s_*Oligoflexus tunisiensis*<br>Bin255 s_*Oligoflexus tunisiensis*
Parcubacteria	Parcubacteria	unclassified Parcubacteria		NA	NA	Bin1642 g_UBA6065<br>Bin1194 o_UBA9983<br>Bin1725 g_2-02-FULL-39-13<br>Bin1572 g_UBA2196<br>Bin2081 c_ABY1<br>Bin1304 s_2-12-FULL-45-10 sp1
Phycodnaviridae (dsDNA virus)	Phycodnaviridae 1 †	Bathycoccus sp. RCC1105 virus BpV	Bathycoccus sp. RCC1105 virus BpV (NC_014765.1)	70 (18)	NA	Bin62 p_Proteobacteria
	Phycodnaviridae 2 †	Micromonas sp. RCC1109 virus MpV1	Micromonas sp. RCC1109 virus MpV1 (NC_014767.1)	76 (54)	NA	Bin62 p_Proteobacteria
	Phycodnaviridae 3 †	Chrysochromulina ericina virus	Chrysochromulina ericina virus isolate CeV-01B (GCF_001399245.1)	71 (5)	NA	Bin1350 d_Bacteria<br>Bin1042 d_Bacteria<br>Bin1755 d_Bacteria<br>Bin97 d_Bacteria<br>Bin1551 d_Bacteria<br>Bin1998 d_Bacteria<br>Bin784 d_Bacteria<br>Bin2102 d_Bacteria<br>Bin494 Unclassified<br>Bin651 Unclassified<br>Bin932 Unclassified<br>Bin1852 Unclassified

	Phycodnaviridae 4 †	Micromonas pusilla virus 12T	Micromonas pusilla virus 12T (GCF_000906035.1)	75 (21)	NA	Bin62 p_Proteobacteria
	Phycodnaviridae 5 †	Micromonas pusilla virus SP1 sensu lato			NA	Bin62 p_Proteobacteria
Planctomycetes	*Gimesia*	*Gimesia maris*	*Gimesia maris* DSM 8797 (GCF_000181475.1)	76 (59)	98<br>98<br>98	Bin1542 s_*Gimesia maris*<br>Bin1604 s_*Gimesia maris*
Tenericutes	*Izimaplasma*	Candidatus *Izimaplasma* sp. HR2	Candidatus *Izimaplasma* sp. HR2 (GCF_000753575.1)	75 (54)	NM	Bin1380 g_*Izimaplasma*
Verrucomicrobia	Verrucomicrobia *<br><br>Verrucomicrobia Arctic95D-9<br>Verrucomicrobia BACL24<br>Verrucomicrobia SW10<br>Verrucomicrobia UBA4506<br>*Haloferula*	*Coraliomargarita akajimensis*, *Chthoniobacter flavus*, *Haloferula* sp. BvORR071, *Prosthecobacter debontii*, *Rubritalea squalenifaciens*	*Coraliomargarita akajimensis* DSM 45221 (NC_014008.1)	73 (19)	95<br>94<br>84	Bin1608 g_*Haloferula*<br>Bin1509 g_Arctic95D-9<br>Bin831 g_Arctic95D-9<br>Bin560 g_Arctic95D-9<br>Bin1278 g_BACL24<br>Bin82 g_BACL24<br>Bin341 g_BACL24<br>Bin1259 g_SW10<br>Bin1231 g_UBA4506<br>Bin1414 f_Opitutaceae<br>Bin192 f_Opitutaceae<br>Bin869 g_UBA6053
			Chthoniobacter flavus Ellin428 (GCF_000173075.1)	71 (6)	83	
			Haloferula sp. BvORR071 (GCF_000739615.1)	72 (18)	85<br>91	
			Prosthecobacter debontii ATCC 700200 (GCF_900167535.1)	71 (7)	85<br>88	
			Rubritalea squalenifaciens DSM 18772 (GCF_900141815.1)	72 (15)	88	

[a] OTUs were classified based on BLAST best hits to MetaBAT MAGs and 16S/18S SSU gene identity and %ANI to reference genomes. [b] Species names as classified in the IMG protein taxonomy file (phylodist data). [†] The functional potential of algal (*Micromonas*) and virus (Phycodnaviridae 1-5) OTUs was not analysed. * Contigs with BLAST hits to bin706 were studied under Pseudohongiellaceae 1 and those with matches to bin2107 were studied as Pseudohongiellaceae 2. Similarly, contigs with best BLAST hits to bin1509, bin831, and bin560 were clustered under Verrucomicrobia Arctic95D-9 and contigs with matches to bin1278, bin82, and bin341 were grouped under Verrucomicrobia BACL24. In each OTU bin, the contigs with BLAST hits to red-highlighted MetaBAT MAGs were excluded from functional potential analysis of that OTU, due to insufficient genes being present in the respective bins. For example, Syntrophales contigs had best BLAST hits to UBA2210 or UBA6078. While the contigs with matches to UBA2210 were studied under Syntrophales UBA2210, the Syntrophales UBA6078 OTU could not be analysed due to low gene counts. The *Micromonas* and Phycodnaviridae1-5 are also highlighted in red because their functional potential was not assessed. ANI, Average nucleotide identity; MAG, metagenome-assembled genome; NA, not applicable; NM, no match. d_, domain; p_, phylum; c_, class; o_, order; f_, family; g_, genus; s_, species.

Table S9 Environmental data for Ace lake samples.

Date	Depth (m)	Salinity (‰)	Lake temperature (°C)	On sample collection date			Monthly average values			Maximum wind velocity (km/h)	Ice cover height
				Air temperature (°C)	Sunlight (h)	Daylength (h)	Air temperature (°C/day)	Sunlight (h/day)	Daylength (h/day)		
20 Dec 2006	5	22	1	-1	9	24	-0.1	10	24	24	NM
20 Dec 2006	11.5	22	0.3	-1	9	24	-0.1	10	24	24	
20 Dec 2006	12.7	28	3	-1	9	24	-0.1	10	24	24	
20 Dec 2006	14	32	2	-1	9	24	-0.1	10	24	24	
20 Dec 2006	18	35	3	-1	9	24	-0.1	10	24	24	
20 Dec 2006	23	42	3	-1	9	24	-0.1	10	24	24	
19 Nov 2008	5	22	-0.4	-5	0.2	22	-4	4	21	80	Ice thickness 1.8 m
21 Nov 2008	11.8	22	-0.3	-3	1	22	-4	4	21	70	
21 Nov 2008	12.8	26	3	-3	1	22	-4	4	21	70	
21 Nov 2008	14.1	31	3	-3	1	22	-4	4	21	70	
21 Nov 2008	18	34	3	-3	1	22	-4	4	21	70	
23 Nov 2008	23	40	3	-2	0	23	-4	4	21	81	
24 Nov 2013	5	21	-0.2	-5	4	24	-4	8	21	31	Completely covered by thick ice
25 Nov 2013	12.5	23	1	-3	2	24	-4	8	21	39	

26 Nov 2013	13.5	30	3	-2	18	24	-4	8	21	55	
26 Nov 2013	15	33	4	-2	18	24	-4	8	21	55	
26 Nov 2013	19	36	3	-2	18	24	-4	8	21	55	
27 Nov 2013	24	42	2	1	3	24	-4	8	21	72	
17 Dec 2013	0	16	NM	-1	1	24	-0.4	10	24	22	Completely covered by thick ice
15 Feb 2014	0	7	3	0.5	8	17	-2	7	17	43	Half covered by ice
2 Jul 2014	5	15	NM	-22	0	0	-20	1	2	26	Completely covered by thick ice
3 Jul 2014	12.5	21	NM	-22	0	0	-20	1	2	24	
3 Jul 2014	13.5	29	NM	-22	0	0	-20	1	2	24	
20 Aug 2014	5	19	2	-25	1	8	-17	3	7	26	Completely covered by 1 m+ ice
21 Aug 2014	13	24	4	-23	0	8	-17	3	7	57	
21 Aug 2014	14.5	27	4	-23	0	8	-17	3	7	57	
20 Oct 2014	5	19	1	-10	8	16	-10	7	15	31	Completely covered by ~2 m ice
20 Oct 2014	12	21	2	-10	8	16	-10	7	15	31	
21 Oct 2014	13	24	4	-10	11	16	-10	7	15	35	
21 Oct 2014	16	27	4	-10	11	16	-10	7	15	35	

21 Oct 2014	19	25	3	-10	11	16	-10	7	15	35	
21 Oct 2014	24	34	2	-10	11	16	-10	7	15	35	
4 Dec 2014	5	21	3	-3	11	24	0.3	10	24	26	~1.8 m ice, very north edge starting to melt behind island
4 Dec 2014	12	22	2	-3	11	24	0.3	10	24	26	
4 Dec 2014	13.4	29	5	-3	11	24	0.3	10	24	26	
4 Dec 2014	14	31	5	-3	11	24	0.3	10	24	26	
3 Dec 2014	19	35	3	-1	6	24	0.3	10	24	55	
3 Dec 2014	24	40	2	-1	6	24	0.3	10	24	55	
8 Jan 2015	0	5	NM	2	0	24	0.8	9	23	74	Mostly covered in poor quality ice
27 Jan 2015	0	10	2	3	9	20	0.8	9	23	65	No Ice

Air temperature, sunlight hours, and maximum wind velocity data were obtained from Australian Antarctic Data Centre, Australia for Davis Station in Vestfold Hills, East Antarctica. Daylength data was obtained from timeanddate.com [44] for Davis Station. The monthly average values are the mean of the values observed in a month and were used for statistical analysis using Primer7. NM, not measured.

Table S10 Pathways and enzymes analysed.

**Pathway/enzyme**	**KEGG numbers**
**Carbon cycle**	
Fermentation	K00016 + K03778 + (K00169 + K00170)/2
Respiration	(K02256 + K02262 + K02274 + K02276)/4
Methanogenesis	(K00400 + K00401)/2
Methane oxidation	(K16157 + K16158 + K16159 + K16161 + K10944m + K10945m + K10946m)/7
Mo/Cu carbon monoxide dehydrogenase	(K03518 + K03519 + K03520)/3
rTCA cycle	(K15230 + K15231)/2
rTCA cycle II	(K15234 + K15233 + K15232 + K00174 + K00175 + K00244)/6
Wood-Ljungdahl pathway	(K00194 + K00197)/2
Calvin cycle	(K01602 + K00855)/2
**Nitrogen cycle**	
Nitrogen fixation	(K02586 + K02591)/2
Ammonia assimilation	(K01915 + K00264 + K00265 + K00266 + K00284)/5
Dissimilatory nitrate reduction	K00370
Dissimilatory nitrite reduction (ammonia-forming)	K03385
Dissimilatory nitrite reduction (nitric oxide-forming)	K00368
Assimilatory nitrate reduction	(K17877 + K00366 + K00360 + K00367)/4
Nitrification	(K10535 + K10944a + K10945a + K10946a)/4
Anammox	(K20932 + K20933 + K20934 + K20935)/4
Nitric oxide reduction	(K02305 + K04561 + K00376)/3
Periplasmic nitrate reduction	K02567
Denitrification	Dissimilatory nitrate reduction + Periplasmic nitrate reduction + Dissimilatory nitrite reduction (NO-forming) + Nitric oxide reduction
**Sulfur cycle**	
SOX system	(K17222 + K17223 + K17224 + K17225 + K17226 + K17227)/6
Cysteine dioxygenase	K00456
Thiosulfate/3-mercaptopyruvate sulfurtransferase	K01011
Sulfate reduction I	(K00958r + K00955 + K00956 + K00957)/4
Sulfate reduction II	K00958r
APS reduction I	(K00860 + K00955)/2
APS reduction II	(K05907 + K00390)/2
APS reduction III	(K00394r + K00395r)/2
PAPS reduction	K00390
Sulfite reduction I	(K00380 + K00381 + K00392)/3
Sulfite reduction II	(K11180r + K11181r)/2
Sulfide oxidation	(K17218 + K17229)/2
Sulfur/polysulfide oxidation	(K11180o + K11181o)/2
Sulfite oxidation	(K00394o + K00395o)/2
APS oxidation	K00958o
Assimilatory sulfate reduction I	Sulfate reduction I + APS reduction I + PAPS reduction + Sulfite reduction I
Assimilatory sulfate reduction II	Sulfate reduction I + APS reduction II + Sulfite reduction I
Dissimilatory sulfate reduction	Sulfate reduction II + APS reduction III + Sulfite reduction II
Sulfide oxidation to sulfate	Sulfide oxidation + Sulfur/polysulfide oxidation + Sulfite oxidation + APS oxidation
**Photosystems**	
Photosystem I	(K02689 + K02690 + K02691 + K02692 + K02693 + K02694)/6

Photosystem II	(K02703 + K02704 + K02705 + K02706 + K02707 + K02708)/6
Type 1 RC core complex (GSB)	(K08940 + K08941 + K08942 + K08943)/4
Anoxygenic photosystem II	(K08928 + K08929)/2
Astaxanthin	(K09836 + K15746)/2
**CRISPR-Cas system**	
CRISPR-Cas spacer acquisition	(K15342 + K09951)/2
CRISPR 1I	(K07012 + K07475)/2
CRISPR 1IA	(K19088 + K19087)/2
CRISPR 1IC	K19117
CRISPR 1IE	(K19123 + K19046)/2
CRISPR 1IF	(K19127 + K19128 + K19129)/3
CRISPR 2II	K09952
CRISPR 2IIA	K19137
CRISPR 2IIB	K07464
CRISPR 1III	K07016
CRISPR 1IIIA	K19138
CRISPR 1IIIB	K19141
**Hydrogenases**	
[NiFe] hydrogenase	(K00437 + K05922)/2
NAD-reducing hydrogenase/diaphorase	K00436
NADP-reducing hydrogenase	K18332
Iron-hydrogenase	K17997
Ferredoxin hydrogenase (monomeric)	K00532
Ferredoxin hydrogenase (trimeric)	K00533
Membrane-bound hydrogenase	K18016
Methanophenazine hydrogenase	K14068
Coenzyme F420 hydrogenase	K00440
5,10-Methenyltetrahydromethanopterin hydrogenase	K13942
F420-non-reducing hydrogenase	K14126
Sulfhydrogenase	K17993
**Substrate transporters**	
Urea transporter	(K11959 + K11960 + K11961 + K11962 + K11963)/5
Sulfate transporter	(K02048 + K02046 + K02047 + K02045)/4
Nitrate/nitrite transporter	(K15576 + K15577 + K15578 + K15579)/4
Bicarbonate transporter	(K11950 + K11951 + K11952 + K11953)/4
Taurine transporter	(K15551 + K15552 + K10831)/3
Sulfonate transporter	(K15553 + K15554 + K15555)/3
Spermidine/putrescine transporter	(K11069 + K11070 + K11071 + K11072)/4
Putrescine transporter	(K11073 + K11074 + K11075 + K11076)/4
Phosphate transporter	(K02040 + K02037 + K02038 + K02036)/4
Phosphonate transporter	(K02044 + K02042 + K02041)/3
2-Aminoethylphosphonate transporter	(K11081 + K11082 + K11083 + K11084)/4
Glycine betaine/proline transporter	(K02002 + K02001 + K02000)/3
Osmoprotectant transporter	(K05845 + K05846 + K05847)/3
Maltose/maltodextrin transporter	(K10108 + K10109 + K10110)/3
Arabinogalactan oligomer/maltooligosaccharide transporter	(K15770 + K15771 + K15772)/3
Raffinose/stachyose/melibiose transporter	(K10117 + K10118 + K10119)/3
Alpha-Glucoside transporter	(K10232 + K10233 + K10234 + K10235)/4
Glucose/arabinose transporter	(K10196 + K10197 + K10198 + K10199)/4
Glucose/mannose transporter	(K17315 + K17316 + K17317)/3
Trehalose/maltose transporter	(K10236 + K10237 + K10238)/3

Trehalose transporter	(K17311 + K17312 + K17313 + K17314)/4
N-Acetylglucosamine transporter	(K10200 + K10201 + K10202)/3
Cellobiose transporter	(K10240 + K10241 + K10242)/3
N,N'-Diacetylchitobiose transporter	(K17329 + K17330 + K17331)/3
Putative chitobiose transporter	(K17244 + K17245 + K17246)/3
L-Arabinose transporter	(K10537 + K10538 + K10539)/3
Lactose/L-arabinose transporter	(K10188 + K10189 + K10190 + K10191)/4
D-Xylose transporter	(K10543 + K10544 + K10545)/3
Xylobiose transporter	(K17326 + K17327 + K17328)/3
Multiple sugar transporter	(K10546 + K10547 + K10548)/3
Fructose transporter	(K10552 + K10553 + K10554)/3
Rhamnose transporter	(K10559 + K10560 + K10561 + K10562)/4
Ribose transporter	(K10439 + K10440 + K10441)/3
Erythritol transporter	(K17202 + K17203 + K17204)/3
Putative fructooligosaccharide transporter	(K10120 + K10121 + K10122)/3
Glycerol transporter	(K17321 + K17322 + K17323 + K17324 + K17325)/5
Putative multiple sugar transporter	(K02027 + K02025 + K02026)/3
Putative simple sugar transporter	(K02058 + K02057 + K02056)/3
Lysine/arginine/ornithine transporter	(K10013 + K10015 + K10016 + K10017)/4
Histidine transporter	(K10014 + K10015 + K10016 + K10017)/4
Glutamine transporter	(K10036 + K10037 + K10038)/3
Arginine transporter	(K09996 + K09997 + K09998 + K09999 + K10000)/5
Glutamate/aspartate transporter	(K10001 + K10002 + K10003 + K10004)/4
Aspartate/glutamate/glutamine transporter	(K10039 + K10040 + K10041)/3
Octopine/nopaline transporter	(K10018 + K10019 + K10020 + K10021)/4
General L-amino acid transporter	(K09969 + K09970 + K09971 + K09972)/4
Glutamate transporter	(K10005 + K10006 + K10007 + K10008)/4
Cystine transporter	(K02424 + K10009 + K10010)/3
L-Cystine transporter	(K16956 + K16957 + K16958 + K16959 + K16960)/5
Arginine/ornithine transporter	(K10022 + K10023 + K10024 + K10025)/4
Arginine/lysine/histidine transporter	(K23059 + K17077 + K23060)/3
Branched-chain amino acid transporter	(K01999 + K01997 + K01998 + K01995 + K01996)/5
Neutral amino acid transporter	(K11954 + K11955 + K11956 + K11957 + K11958)/5
D-Methionine transporter	(K02073 + K02072 + K02071)/3
Oligopeptide transporter	(K15580 + K15581 + K15582 + K15583 + K10823)/5
Dipeptide transporter	(K12368 + K12369 + K12370 + K12371 + K12372 + K16199 + K16200 + K16201 + K16202)/9
**Substrate degradation pathways/enzymes**	
Licheninase	K01216
Glucan endo-1,3-beta-glucosidase	(K01199 + K19891 + K19892 + K19893)/4
Beta-galactosidase	(K01190 + K12111 + K12308 + K12309 + K01188 + K05349 + K05350)/7
Xylan 1,4-beta-xylosidase	(K01198 + K15920 + K22268)/3
Cellulase/endoglucanase	(K01179 + K19357 + K20542)/3
Laminarinase	K01180
Carrageenase	(K20846 + K20850)/2
Agarase	(K01219 + K20851)/2
Pullulanase	(K01200 + K21575)/2
Beta-amylase	K01177
Maltogenic alpha-amylase	(K01208 + K05992)/2
Exo-amylase	K22253
Glucoamylase/glucan 1,4-alpha-glucosidase	(K01178 + K12047 + K21574)/3
Sucrose-6-phosphatase	K07024
Beta-fructofuranosidase	K01193

Cellobiosidase	(K01225 + K19668)/2
Urea catabolism	(K01428 + K01429 + K01430)/3 + K01941
Glycerol catabolism	(K00111 + K00112 + K00113 + K00864 + K00005)/5
Methylphosphonate catabolism	(K06163 + K06164 + K06165 + K06166 + K05780)/5
Aminoethylphosphonate catabolism	(K03430 + K05306)/2
DMSP catabolism	K16953 + K17486
Glycolate utilization	K11472
Creatine utilization	K08688
Sarcosine utilization I	K00301
Sarcosine utilization II	(K00302 + K00303 + K00304 + K00305)/4
Taurine utilization	(K03851 + K03852)/2
Fucoidan degradation	K15923
Fucose utilization	(K00879 + K01628)/2
Fucose utilization II	K00064
Rhamnose utilization	(K00848 + K01629)/2
Chitin degradation I	K01183
Chitin degradation II	K13381
Dimethylamine utilization	K16178
Monomethylamine utilization	K16176
Cellobiose utilization	K00702
Starch degradation	K01176
**Substrate biosynthesis and storage**	
PHA storage	K03821 + K05973
Archaeal glycerol synthesis	K00096
Glycogen synthesis (overall)	K16149
Glycogen synthesis I	(K00975 + K00703)/2
Glycogen synthesis II	(K16146 + K16147)/2
**Others**	
Superoxidedismutase	(K00518 + K04564 + K04565 + K16627)/4
DMSO reduction	K07306
Sulfate ester hydrolysis	K01130
Trimethylamine/glycine betaine methyltransferase	K14083

APS, adenosine 5'-phosphosulfate; CRISPR, clustered regularly interspaced short palindromic repeats; DMSO, dimethyl sulfoxide; DMSP, dimethylsulfoniopropionate; GSB, green sulfur bacteria; PAPS, 3'-phosphoadenosine-5'-phosphosulfate; PHA, polyhydroxyalkanoates; RC core complex, reaction center core complex; rTCA cycle, reverse tricarboxylic acid cycle; SOX system, sulfur-oxidation system.

Table S11 KEGG numbers used to calculate abundance of specific pathways and enzymes.

KEGG number	Pathway/process/enzyme	Protein name	EC number
K00016	Fermentation	L-lactate dehydrogenase	EC:1.1.1.27
K03778	Fermentation	D-lactate dehydrogenase; LdhA	EC:1.1.1.28
K00169	Fermentation	Pyruvate ferredoxin oxidoreductase alpha subunit	EC:1.2.7.1
K00170	Fermentation	Pyruvate ferredoxin oxidoreductase beta subunit	EC:1.2.7.1
K02256	Respiration	Cytochrome c oxidase subunit 1	EC:1.9.3.1
K02262	Respiration	Cytochrome c oxidase subunit 3	
K02274	Respiration	Cytochrome c oxidase subunit I	EC:1.9.3.1
K02276	Respiration	Cytochrome c oxidase subunit III	EC:1.9.3.1
K00400	Methanogenesis	Methyl coenzyme M reductase system, component A2	
K00401	Methanogenesis	Methyl coenzyme M reductase beta subunit; McrB	EC:2.8.4.1
K16157	Methane oxidation	Methane monooxygenase component A alpha chain	EC:1.14.13.25
K16158	Methane oxidation	Methane monooxygenase component A beta chain	EC:1.14.13.25
K16159	Methane oxidation	Methane monooxygenase component A gamma chain	EC:1.14.13.25
K16161	Methane oxidation	Methane monooxygenase component C	EC:1.14.13.25
K10944a/m	Methane oxidation/Nitrification	Methane/ammonia monooxygenase subunit A	EC:1.14.18.3, EC:1.14.99.39
K10945a/m	Methane oxidation/Nitrification	Methane/ammonia monooxygenase subunit B	
K10946a/m	Methane oxidation/Nitrification	Methane/ammonia monooxygenase subunit C	
K03518	Mo/Cu carbon monoxide dehydrogenase	aerobic carbon-monoxide dehydrogenase small subunit	EC:1.2.5.3
K03519	Mo/Cu carbon monoxide dehydrogenase	aerobic carbon-monoxide dehydrogenase medium subunit	EC:1.2.5.3
K03520	Mo/Cu carbon monoxide dehydrogenase	aerobic carbon-monoxide dehydrogenase large subunit	EC:1.2.5.3
K15230	rTCA cycle	ATP-citrate lyase alpha-subunit; AclA	EC:2.3.3.8
K15231	rTCA cycle	ATP-citrate lyase beta-subunit; AclB	EC:2.3.3.8
K15232	rTCA cycle II	citryl-CoA synthetase large subunit	EC:6.2.1.18
K15233	rTCA cycle II	citryl-CoA synthetase small subunit	
K15234	rTCA cycle II	citryl-CoA lyase	EC:4.1.3.34
K00174	rTCA cycle II	2-oxoglutarate/2-oxoacid ferredoxin oxidoreductase subunit alpha	EC:1.2.7.3, EC1.2.7.11
K00175	rTCA cycle II	2-oxoglutarate/2-oxoacid ferredoxin oxidoreductase subunit beta	EC:1.2.7.3 1.2.7.11
K00244	rTCA cycle II	fumarate reductase flavoprotein subunit	EC:1.3.5.4
K00194	Wood-Ljungdahl pathway	Acetyl-CoA decarbonylase/synthase; AcsD	EC:2.1.1.245
K00197	Wood-Ljungdahl pathway	Acetyl-CoA decarbonylase/synthase; AcsC	EC:2.1.1.245
K01602	Calvin cycle	Ribulose-bisphosphate carboxylase small chain; RbcS	EC:4.1.1.39
K00855	Calvin cycle	Phosphoribulokinase; PrkB	EC:2.7.1.19
K02586	Nitrogen fixation	Nitrogenase molybdenum-iron protein alpha chain; NifD	EC:1.18.6.1

K02591	Nitrogen fixation	Nitrogenase molybdenum-iron protein beta chain; NifK	EC:1.18.6.1
K01915	Ammonia assimilation	Glutamine synthetase; GlnA	EC 6.3.1.2
K00264	Ammonia assimilation	Glutamate synthase (NADH); Glt1	EC 1.4.1.14
K00265	Ammonia assimilation	Glutamate synthase (NADPH) large chain; GltB	EC 1.4.1.13
K00266	Ammonia assimilation	Glutamate synthase (NADPH) small chain; GltD	EC 1.4.1.13
K00284	Ammonia assimilation	Glutamate synthase (ferredoxin); GltS	EC 1.4.7.1
K00370	Dissimilatory nitrate reduction	Nitrate reductase/nitrite oxidoreductase, alpha subunit; NarG, NarZ, NxrA	EC:1.7.5.1, EC:1.7.99.-
K03385	Dissimilatory nitrite reduction (ammonia-forming)	Nitrite reductase (cytochrome c-552); NrfA	EC:1.7.2.2
K00368	Dissimilatory nitrite reduction (NO-forming)	Nitrite reductase (NO-forming); NirK	EC:1.7.2.1
K17877	Assimilatory nitrate reduction	Nitrite reductase (NAD(P)H); Nit-6	EC:1.7.1.4
K00360	Assimilatory nitrate reduction	Assimilatory nitrate reductase electron transfer subunit; NasB	EC:1.7.99.-
K00366	Assimilatory nitrate reduction	Ferredoxin-nitrite reductase; NirA	EC:1.7.7.1
K00367	Assimilatory nitrate reduction	Ferredoxin-nitrate reductase; NarB	EC:1.7.7.2
K10535	Nitrification	Hydroxylamine dehydrogenase	EC:1.7.2.6
K02305	Nitric oxide reduction	Nitric oxide reductase subunit C; NorC	
K04561	Nitric oxide reduction	Nitric oxide reductase subunit B; NorB	EC:1.7.2.5
K00376	Nitric oxide reduction	Nitrous oxide reductase; NosZ	EC:1.7.2.4
K20932	Anammox	hydrazine synthase subunit	EC:1.7.2.7
K20933	Anammox	hydrazine synthase subunit	EC:1.7.2.7
K20934	Anammox	hydrazine synthase subunit	EC:1.7.2.7
K20935	Anammox	hydrazine dehydrogenase [EC:1.7.2.8]	
K02567	Periplasmic nitrate reduction	Periplasmic nitrate reductase; NapA	EC:1.7.99.-
K17222	SOX system	L-cysteine S-thiosulfotransferase; SoxA	EC:2.8.5.2
K17223	SOX system	L-cysteine S-thiosulfotransferase; SoxX	EC:2.8.5.2
K17224	SOX system	S-Sulfosulfanyl-L-cysteine sulfohydrolase; SoxB	EC:3.1.6.20
K17225	SOX system	Sulfane dehydrogenase subunit; SoxC	
K17226	SOX system	Sulfur-oxidizing protein; SoxY	
K17227	SOX system	Sulfur-oxidizing protein; SoxZ	
K00456	Cysteine dioxygenase	Cysteine dioxygenase; Cdo1	EC:1.13.11.20
K01011	Thiosulfate/3-mercaptopyruvate sulfurtransferase	Thiosulfate/3-mercaptopyruvate sulfurtransferase; Tst, Mpst	EC:2.8.1.1, EC:2.8.1.2
K00955	Sulfate reduction I/APS reduction I	Bifunctional enzyme CysN/CysC; CycNC	EC:2.7.7.4, EC:2.7.1.25
K00956	Sulfate reduction I	Sulfate adenylyltransferase subunit 1; CysN	EC:2.7.7.4
K00957	Sulfate reduction I	Sulfate adenylyltransferase subunit 2; CycD	EC:2.7.7.4
K00958o/r	Sulfate reduction I/Sulfate reduction II/APS	Sulfate adenylyltransferase; Sat	EC:2.7.7.4

	oxidation		
K00860	APS reduction I	Adenylylsulfate kinase; CycC	EC:2.7.1.25
K05907	APS reduction II	Adenylylsulfate reductase (glutathione); Apr	EC:1.8.4.9
K00390	APS reduction II/PAPS reduction	Phosphoadenosine phosphosulfate reductase; CysH	EC:1.8.4.8, EC:1.8.4.10
K00394o/r	APS reduction III/Sulfite oxidation	Adenylylsulfate reductase, subunit A; AprA	EC:1.8.99.2
K00395o/r	APS reduction III/Sulfite oxidation	Adenylylsulfate reductase, subunit B; AprB	EC:1.8.99.2
K00380	Sulfite reduction I	Sulfite reductase (NADPH) flavoprotein alpha-component; CysJ	EC:1.8.1.2
K00381	Sulfite reduction I	Sulfite reductase (NADPH) hemoprotein beta-component; CysI	EC:1.8.1.2
K00392	Sulfite reduction I	Sulfite reductase (ferredoxin); Sir	EC:1.8.7.1
K11180o/r	Sulfite reduction II/Sulfur/polysulfide oxidation	Dissimilatory sulfite reductase alpha subunit; DsrA	EC:1.8.99.5
K11181o/r	Sulfite reduction II/Sulfur/polysulfide oxidation	Dissimilatory sulfite reductase beta subunit; DsrB	EC:1.8.99.5
K17218	Sulfide oxidation	Sulfide:quinone oxidoreductase; Sqr	EC:1.8.5.4
K17229	Sulfide oxidation	Sulfide dehydrogenase [flavocytochrome c] flavoprotein chain; FccB	EC:1.8.2.3
K02689	Photosystem I	Photosystem I P700 chlorophyll a apoprotein A1; PsaA	
K02690	Photosystem I	Photosystem I P700 chlorophyll a apoprotein A2; PsaB	
K02691	Photosystem I	Photosystem I subunit VII; PsaC	
K02692	Photosystem I	Photosystem I subunit II; PsaD	
K02693	Photosystem I	Photosystem I subunit IV; PsaE	
K02694	Photosystem I	Photosystem I subunit III; PsaF	
K02703	Photosystem II	Photosystem II P680 reaction center D1 protein; PsbA	EC:1.10.3.9
K02704	Photosystem II	Photosystem II CP47 chlorophyll apoprotein; PsbB	
K02705	Photosystem II	Photosystem II CP43 chlorophyll apoprotein; PsbC	
K02706	Photosystem II	Photosystem II P680 reaction center D2 protein; PsbD	EC:1.10.3.9
K02707	Photosystem II	Photosystem II cytochrome b559 subunit alpha; PsbE	
K02708	Photosystem II	Photosystem II cytochrome b559 subunit beta; PsbF	
K08940	Type 1 RC core complex (GSB)	Photosystem P840 reaction center large subunit; PscA	
K08941	Type 1 RC core complex (GSB)	Photosystem P840 reaction center iron-sulfur protein; PscB	
K08942	Type 1 RC core complex (GSB)	Photosystem P840 reaction center cytochrome c551; PscC	
K08943	Type 1 RC core complex (GSB)	Photosystem P840 reaction center protein PscD	
K08928	Anoxygenic photosystem II	Photosynthetic reaction center L subunit; PufL	
K08929	Anoxygenic photosystem II	Photosynthetic reaction center M subunit; PufM	
K09836	Astaxanthin	beta-carotene ketolase (CrtW type)	
K15746	Astaxanthin	beta-carotene 3-hydroxylase	EC:1.14.15.24
K15342	CRISPR-Cas spacer acquisition	CRISP-associated protein Cas1	
K09951	CRISPR-Cas spacer acquisition	CRISPR-associated protein Cas2	
K07012	CRISPR 1I	CRISPR-associated endonuclease/helicase Cas3	EC:3.1.-.-,

			EC:3.6.4.-
K07475	CRISPR 1I	CRISPR-associated endonuclease Cas3-HD	EC:3.1.-.-
K19088	CRISPR 1IA	CRISPR-associated protein Cst1; Cas8a	
K19087	CRISPR 1IA	CRISPR-associated protein Csa5	
K19117	CRISPR 1IC	CRISPR-associated protein Csd1; Cas8c	
K19123	CRISPR 1IE	CRISPR system Cascade subunit CasA; Cse1	
K19046	CRISPR 1IE	CRISPR system Cascade subunit CasB; Cse2	
K19127	CRISPR 1IF	CRISPR-associated protein Csy1	
K19128	CRISPR 1IF	CRISPR-associated protein Csy2	
K19129	CRISPR 1IF	CRISPR-associated protein Csy3	
K09952	CRISPR 2II	CRISPR-associated endonuclease Csn1; Cas9	EC:3.1.-.-
K19137	CRISPR 2IIA	CRISPR-associated protein Csn2	
K07464	CRISPR 2IIB	CRISPR-associated exonuclease Cas4	EC:3.1.12.1
K07016	CRISPR 1III	CRISPR-associated protein Csm1; Cas10	
K19138	CRISPR 1IIIA	CRISPR-associated protein Csm2	
K19141	CRISPR 1IIIB	CRISPR-associated protein Cmr5	
K00437	[NiFe] hydrogenase	[NiFe] Hydrogenase large subunit; HydB	EC:1.12.2.1
K05922	[NiFe] hydrogenase	Quinone-reactive Ni/Fe-hydrogenase large subunit; HydB	EC:1.12.5.1
K00436	NAD-reducing hydrogenase/diaphorase	NAD-reducing hydrogenase large subunit; HoxH	EC:1.12.1.2
K18332	NADP-reducing hydrogenase	NADP-reducing hydrogenase subunit; HndD	EC:1.12.1.3
K17997	Iron-hydrogenase	Iron-hydrogenase subunit alpha; HydA	EC:1.12.1.4
K00532	Ferredoxin hydrogenase (monomeric)	Ferredoxin hydrogenase	EC:1.12.7.2
K00533	Ferredoxin hydrogenase (trimeric)	Ferredoxin hydrogenase large subunit	EC:1.12.7.2
K18016	Membrane-bound hydrogenase	Membrane-bound hydrogenase subunit alpha; MbhL	EC:1.12.7.2
K14068	Methanophenazine hydrogenase	Methanophenazine hydrogenase, large subunit; VhoA, VhtA	EC:1.12.98.3
K00440	Coenzyme F420 hydrogenase	Coenzyme F420 hydrogenase subunit alpha; FrhA	EC:1.12.98.1
K13942	5,10-Methenyltetrahydromethanopterin hydrogenase	5,10-Methenyltetrahydromethanopterin hydrogenase; Hmd	EC:1.12.98.2
K14126	F420-non-reducing hydrogenase	F420-Non-reducing hydrogenase large subunit; MvhA, VhuA, VhcA	EC:1.12.99.-, EC:1.8.98.5
K17993	Sulfhydrogenase	Sulfhydrogenase alpha subunit; HydA	EC:1.12.1.3, EC:1.12.1.5
K11472	Glycolate utilization	Glycolate oxidase FAD binding subunit; GlcE	
K08688	Creatine utilization	Creatinase	EC:3.5.3.3
K00301	Sarcosine utilization I	Sarcosine oxidase	EC:1.5.3.1
K00302	Sarcosine utilization II	Sarcosine oxidase, subunit alpha	EC:1.5.3.1
K00303	Sarcosine utilization II	Sarcosine oxidase, subunit beta	EC:1.5.3.1

K00304	Sarcosine utilization II	Sarcosine oxidase, subunit delta	EC:1.5.3.1
K00305	Sarcosine utilization II	Sarcosine oxidase, subunit gamma	EC:1.5.3.1
K03851	Taurine utilization	Taurine-pyruvate aminotransferase; Tpa	EC:2.6.1.77
K03852	Taurine utilization	Sulfoacetaldehyde acetyltransferase	EC:2.3.3.15
K01130	Sulfate ester hydrolysis	Arylsulfatase; AslA	EC:3.1.6.1
K15923	Fucoidan degradation	Alpha-L-fucosidase 2; AXY8, FUC95A, AfcA	EC:3.2.1.51
K00879	Fucose utilization	L-fuculokinase; FucK	EC:2.7.1.51
K01628	Fucose utilization	L-fuculose-phosphate aldolase; FucA	EC:4.1.2.17
K00064	Fucose utilization II	D-threo-aldose 1-dehydrogenase	EC:1.1.1.122
K00848	Rhamnose utilization	Rhamnulokinase; RhaB	EC:2.7.1.5
K01629	Rhamnose utilization	Rhamnulose-1-phosphate aldolase; RhaD	EC:4.1.2.19
K01183	Chitin degradation I	Chitinase	EC:3.2.1.14
K13381	Chitin degradation II	Bifunctional chitinase/lysozyme	EC:3.2.1.14, EC:3.2.1.17
K14083	Trimethylamine/glycine betaine methyltransferase	Trimethylamine---corrinoid protein Co-methyltransferase; MttB	EC:2.1.1.250
K16178	Dimethylamine utilization	Dimethylamine---corrinoid protein Co-methyltransferase; MtbB	EC:2.1.1.249
K16176	Monomethylamine utilization	Methylamine---corrinoid protein Co-methyltransferase; MtmB	EC:2.1.1.248
K00702	Cellobiose utilization	Cellobiose phosphorylase	EC:2.4.1.20
K16149	Glycogen synthesis (overall)	1,4-alpha-glucan branching enzyme	EC:2.4.1.18
K00975	Glycogen synthesis I	Glucose-1-phosphate adenylyltransferase; GlgC	EC:2.7.7.27
K00703	Glycogen synthesis I	Starch synthase; GlgA	EC:2.4.1.21
K16146	Glycogen synthesis II	Maltokinase; Pep2	EC:2.7.1.175
K16147	Glycogen synthesis II	Starch synthase (maltosyl-transferring); GlcE	EC:2.4.99.16
K01176	Starch degradation	Alpha-amylase; AMY, AmyA, MalS	EC:3.2.1.1
K11959	Urea transporter	Urea transport system substrate-binding protein; UrtA	
K11960	Urea transporter	Urea transport system permease protein; UrtB	
K11961	Urea transporter	Urea transport system permease protein; UrtC	
K11962	Urea transporter	urea transport system ATP-binding protein; UrtD	
K11963	Urea transporter	urea transport system ATP-binding protein; UrtE	
K02045	Sulfate transporter	Sulfate/thiosulfate transport system ATP-binding protein; CysA	EC:7.3.2.3
K02046	Sulfate transporter	Sulfate/thiosulfate transport system permease protein; CysU	
K02047	Sulfate transporter	Sulfate/thiosulfate transport system permease protein; CysW	
K02048	Sulfate transporter	Sulfate/thiosulfate transport system substrate-binding protein; CysP	
K15576	Nitrate/nitrite transporter	Nitrate/nitrite transport system substrate-binding protein; NrtA, NasF, CynA	
K15577	Nitrate/nitrite transporter	Nitrate/nitrite transport system permease protein; NrtB, NasE, CynB	
K15578	Nitrate/nitrite transporter	Nitrate/nitrite transport system ATP-binding protein; NrtC, NasD	EC:3.6.3.-
K15579	Nitrate/nitrite transporter	Nitrate/nitrite transport system ATP-binding protein; NrtD, CynD	

K11950	Bicarbonate transporter	Bicarbonate transport system substrate-binding protein; CmpA	
K11951	Bicarbonate transporter	Bicarbonate transport system permease protein; CmpB	
K11952	Bicarbonate transporter	Bicarbonate transport system ATP-binding protein; CmpC	EC:3.6.3.-
K11953	Bicarbonate transporter	Bicarbonate transport system ATP-binding protein; CmpD	EC:3.6.3.-
K10831	Taurine transporter	Taurine transport system ATP-binding protein; TauB	EC:7.6.2.7
K15551	Taurine transporter	Taurine transport system substrate-binding protein; TauA	
K15552	Taurine transporter	Taurine transport system permease protein; TauC	
K15553	Sulfonate transporter	Sulfonate transport system substrate-binding protein; SsuA	
K15554	Sulfonate transporter	Sulfonate transport system permease protein; SsuC	
K15555	Sulfonate transporter	Sulfonate transport system ATP-binding protein; SsuB	EC:3.6.3.-
K11069	Spermidine/putrescine transporter	Spermidine/putrescine transport system substrate-binding protein; PotD	
K11070	Spermidine/putrescine transporter	Spermidine/putrescine transport system permease protein; PotC	
K11071	Spermidine/putrescine transporter	Spermidine/putrescine transport system permease protein; PotB	
K11072	Spermidine/putrescine transporter	Spermidine/putrescine transport system ATP-binding protein; PotA	EC:7.6.2.11
K11073	Putrescine transporter	Putrescine transport system substrate-binding protein; PotF	
K11074	Putrescine transporter	Putrescine transport system permease protein; PotI	
K11075	Putrescine transporter	Putrescine transport system permease protein; PotH	
K11076	Putrescine transporter	Putrescine transport system ATP-binding protein; PotG	
K02036	Phosphate transporter	Phosphate transport system ATP-binding protein; PstB	EC:7.3.2.1
K02037	Phosphate transporter	Phosphate transport system permease protein; PstC	
K02038	Phosphate transporter	Phosphate transport system permease protein; PstA	
K02040	Phosphate transporter	Phosphate transport system substrate-binding protein; PstS	
K02041	Phosphonate transporter	Phosphonate transport system ATP-binding protein	EC:7.3.2.2
K02042	Phosphonate transporter	Phosphonate transport system permease protein; PhnE	
K02044	Phosphonate transporter	Phosphonate transport system substrate-binding protein; PhnD	
K11081	2-Aminoethylphosphonate transporter	2-Aminoethylphosphonate transport system substrate-binding protein; PhnS	
K11082	2-Aminoethylphosphonate transporter	2-Aminoethylphosphonate transport system permease protein; PhnV	
K11083	2-Aminoethylphosphonate transporter	2-Aminoethylphosphonate transport system permease protein; PhnU	
K11084	2-Aminoethylphosphonate transporter	2-Aminoethylphosphonate transport system ATP-binding protein; PhnT	
K02000	Glycine betaine/proline transporter	Glycine betaine/proline transport system ATP-binding protein; ProV	EC:7.6.2.9
K02001	Glycine betaine/proline transporter	Glycine betaine/proline transport system permease protein; ProW	
K02002	Glycine betaine/proline transporter	Glycine betaine/proline transport system substrate-binding protein; ProX	
K05845	Osmoprotectant transporter	Osmoprotectant transport system substrate-binding protein; OpuC	
K05846	Osmoprotectant transporter	Osmoprotectant transport system permease protein; OpuBD	
K05847	Osmoprotectant transporter	Osmoprotectant transport system ATP-binding protein; OpuA	EC:7.6.2.9
K10108	Maltose/maltodextrin transporter	Maltose/maltodextrin transport system substrate-binding protein; MalE	
K10109	Maltose/maltodextrin transporter	Maltose/maltodextrin transport system permease protein; MalF	

K10110	Maltose/maltodextrin transporter	Maltose/maltodextrin transport system permease protein; MalG	
K15770	Arabinogalactan oligomer/maltooligosaccharide transporter	Arabinogalactan oligomer/maltooligosaccharide transport system substrate-binding protein; CycB, GanO	
K15771	Arabinogalactan oligomer/maltooligosaccharide transporter	Arabinogalactan oligomer/maltooligosaccharide transport system permease protein; GanP	
K15772	Arabinogalactan oligomer/maltooligosaccharide transporter	Arabinogalactan oligomer/maltooligosaccharide transport system permease protein; GanQ	
K10117	Raffinose/stachyose/melibiose transporter	Raffinose/stachyose/melibiose transport system substrate-binding protein; MsmE	
K10118	Raffinose/stachyose/melibiose transporter	Raffinose/stachyose/melibiose transport system permease protein; MsmF	
K10119	Raffinose/stachyose/melibiose transporter	Raffinose/stachyose/melibiose transport system permease protein; MsmG	
K10232	Alpha-Glucoside transporter	Alpha-glucoside transport system substrate-binding protein; AglE, GgtB	
K10233	Alpha-Glucoside transporter	Alpha-glucoside transport system permease protein; AglF, GgtC	
K10234	Alpha-Glucoside transporter	Alpha-glucoside transport system permease protein; AglG, GgtD	
K10235	Alpha-Glucoside transporter	Alpha-glucoside transport system ATP-binding protein; AglK	
K10196	Glucose/arabinose transporter	Glucose/arabinose transport system substrate-binding protein	
K10197	Glucose/arabinose transporter	Glucose/arabinose transport system permease protein	
K10198	Glucose/arabinose transporter	Glucose/arabinose transport system permease protein	
K10199	Glucose/arabinose transporter	Glucose/arabinose transport system ATP-binding protein	
K17315	Glucose/mannose transporter	Glucose/mannose transport system substrate-binding protein; GtsA, GlcE	
K17316	Glucose/mannose transporter	Glucose/mannose transport system permease protein; GtsB, GlcF	
K17317	Glucose/mannose transporter	Glucose/mannose transport system permease protein; GtsC, GlcG	
K10236	Trehalose/maltose transporter	Trehalose/maltose transport system substrate-binding protein; ThuE	
K10237	Trehalose/maltose transporter	Trehalose/maltose transport system permease protein; ThuF, SugA	
K10238	Trehalose/maltose transporter	Trehalose/maltose transport system permease protein; ThuG, SugB	
K17311	Trehalose transporter	Trehalose transport system substrate-binding protein; TreS	
K17312	Trehalose transporter	Trehalose transport system permease protein; TreT	
K17313	Trehalose transporter	Trehalose transport system permease protein; TreU	
K17314	Trehalose transporter	Trehalose transport system ATP-binding protein; TreV	
K10200	N-Acetylglucosamine transporter	N-acetylglucosamine transport system substrate-binding protein	
K10201	N-Acetylglucosamine transporter	N-acetylglucosamine transport system permease protein	
K10202	N-Acetylglucosamine transporter	N-acetylglucosamine transport system permease protein	
K10240	Cellobiose transporter	Cellobiose transport system substrate-binding protein; CebE	
K10241	Cellobiose transporter	Cellobiose transport system permease protein; CebF	
K10242	Cellobiose transporter	Cellobiose transport system permease protein; CebG	
K17329	N,N'-Diacetylchitobiose transporter	N,N'-diacetylchitobiose transport system substrate-binding protein; DasA	
K17330	N,N'-Diacetylchitobiose transporter	N,N'-diacetylchitobiose transport system permease protein; DasB	

K17331	N,N'-Diacetylchitobiose transporter	N,N'-diacetylchitobiose transport system permease protein; DasC	
K17244	Putative chitobiose transporter	Putative chitobiose transport system substrate-binding protein; ChiE	
K17245	Putative chitobiose transporter	Putative chitobiose transport system permease protein; ChiF	
K17246	Putative chitobiose transporter	Putative chitobiose transport system permease protein; ChiG	
K10537	L-Arabinose transporter	L-arabinose transport system substrate-binding protein; AraF	
K10538	L-Arabinose transporter	L-arabinose transport system permease protein; AraH	
K10539	L-Arabinose transporter	L-arabinose transport system ATP-binding protein; AraG	EC:7.5.2.12
K10188	Lactose/L-arabinose transporter	Lactose/L-arabinose transport system substrate-binding protein; LacE, AraN	
K10189	Lactose/L-arabinose transporter	Lactose/L-arabinose transport system permease protein; LacF, AraP	
K10190	Lactose/L-arabinose transporter	Lactose/L-arabinose transport system permease protein; LacG, AraQ	
K10191	Lactose/L-arabinose transporter	Lactose/L-arabinose transport system ATP-binding protein; LacK	
K10543	D-Xylose transporter	D-xylose transport system substrate-binding protein; XylF	
K10544	D-Xylose transporter	D-xylose transport system permease protein; XylH	
K10545	D-Xylose transporter	D-xylose transport system ATP-binding protein; XylG	EC:3.6.3.17
K17326	Xylobiose transporter	Xylobiose transport system substrate-binding protein; BxlE	
K17327	Xylobiose transporter	Xylobiose transport system permease protein; BxlF	
K17328	Xylobiose transporter	Xylobiose transport system permease protein; BxlG	
K10546	Multiple sugar transporter	Putative multiple sugar transport system substrate-binding protein; ChvE	
K10547	Multiple sugar transporter	Putative multiple sugar transport system permease protein; GguB	
K10548	Multiple sugar transporter	Putative multiple sugar transport system ATP-binding protein; GguA	EC:3.6.3.17
K10552	Fructose transporter	Fructose transport system substrate-binding protein; FrcB	
K10553	Fructose transporter	Fructose transport system permease protein; FrcC	
K10554	Fructose transporter	Fructose transport system ATP-binding protein; FrcA	
K10559	Rhamnose transporter	Rhamnose transport system substrate-binding protein; RhaS	
K10560	Rhamnose transporter	Rhamnose transport system permease protein; RhaP	
K10561	Rhamnose transporter	Rhamnose transport system permease protein; RhaQ	
K10562	Rhamnose transporter	Rhamnose transport system ATP-binding protein; RhaT	EC:3.6.3.17
K10439	Ribose transporter	Ribose transport system substrate-binding protein; RbsB	
K10440	Ribose transporter	Ribose transport system permease protein; RbsC	
K10441	Ribose transporter	Ribose transport system ATP-binding protein	EC:3.6.3.17
K17202	Erythritol transporter	Erythritol transport system substrate-binding protein; EryG	
K17203	Erythritol transporter	Erythritol transport system permease protein; EryF	
K17204	Erythritol transporter	Erythritol transport system ATP-binding protein; EryE	
K10120	Putative fructooligosaccharide transporter	Fructooligosaccharide transport system substrate-binding protein; MsmE	
K10121	Putative fructooligosaccharide transporter	Fructooligosaccharide transport system permease protein; MsmF	
K10122	Putative fructooligosaccharide transporter	Fructooligosaccharide transport system permease protein; MsmG	
K17321	Glycerol transporter	Glycerol transport system substrate-binding protein; GlpV	

K17322	Glycerol transporter	Glycerol transport system permease protein; GlpP	
K17323	Glycerol transporter	Glycerol transport system permease protein; GlpQ	
K17324	Glycerol transporter	Glycerol transport system ATP-binding protein; GlpS	
K17325	Glycerol transporter	Glycerol transport system ATP-binding protein; GlpT	
K02025	Putative multiple sugar transporter	Multiple sugar transport system permease protein	
K02026	Putative multiple sugar transporter	Multiple sugar transport system permease protein	
K02027	Putative multiple sugar transporter	Multiple sugar transport system substrate-binding protein	
K02056	Putative simple sugar transporter	Simple sugar transport system ATP-binding protein	EC:3.6.3.17
K02057	Putative simple sugar transporter	Simple sugar transport system permease protein	
K02058	Putative simple sugar transporter	Simple sugar transport system substrate-binding protein	
K10013	Lysine/arginine/ornithine transporter	Lysine/arginine/ornithine transport system substrate-binding protein; ArgT	
K10014	Histidine transporter	Histidine transport system substrate-binding protein; HisJ	
K10015	Lysine/arginine/ornithine transporter/Histidine transporter	Histidine transport system permease protein; HisM	
K10016	Lysine/arginine/ornithine transporter/Histidine transporter	Histidine transport system permease protein; HisQ	
K10017	Lysine/arginine/ornithine transporter/Histidine transporter	Histidine transport system ATP-binding protein; HisP	EC:7.4.2.1
K10036	Glutamine transporter	Glutamine transport system substrate-binding protein; GlnH	
K10037	Glutamine transporter	Glutamine transport system permease protein; GlnP	
K10038	Glutamine transporter	Glutamine transport system ATP-binding protein; GlnQ	EC:7.4.2.1
K09996	Arginine transporter	Arginine transport system substrate-binding protein; ArtJ	
K09997	Arginine transporter	Arginine transport system substrate-binding protein; ArtI	
K09998	Arginine transporter	Arginine transport system permease protein; ArtM	
K09999	Arginine transporter	Arginine transport system permease protein; ArtQ	
K10000	Arginine transporter	Arginine transport system ATP-binding protein; ArtP	EC:7.4.2.1
K10001	Glutamate/aspartate transporter	Glutamate/aspartate transport system substrate-binding protein; GltI	
K10002	Glutamate/aspartate transporter	Glutamate/aspartate transport system permease protein; GltK, AatM	
K10003	Glutamate/aspartate transporter	Glutamate/aspartate transport system permease protein; GltJ, AatQ	
K10004	Glutamate/aspartate transporter	Glutamate/aspartate transport system ATP-binding protein; GltL, AatP	EC:7.4.2.1
K10039	Aspartate/glutamate/glutamine transporter	Aspartate/glutamate/glutamine transport system substrate-binding protein; Peb1A, GlnH	
K10040	Aspartate/glutamate/glutamine transporter	Aspartate/glutamate/glutamine transport system permease protein; Peb1B, GlnP, GlnM	
K10041	Aspartate/glutamate/glutamine transporter	Aspartate/glutamate/glutamine transport system ATP-binding protein; Peb1C, GlnQ	EC:7.4.2.1
K10018	Octopine/nopaline transporter	Octopine/nopaline transport system substrate-binding protein; OccT, NocT	

K10019	Octopine/nopaline transporter	Octopine/nopaline transport system permease protein; OccM, NocM	
K10020	Octopine/nopaline transporter	Octopine/nopaline transport system permease protein; OccQ, NocQ	
K10021	Octopine/nopaline transporter	Octopine/nopaline transport system ATP-binding protein; OccP, NocP	EC:7.4.2.1
K09969	General L-amino acid transporter	General L-amino acid transport system substrate-binding protein; AapJ, BztA	
K09970	General L-amino acid transporter	General L-amino acid transport system permease protein; AapQ, BztB	
K09971	General L-amino acid transporter	General L-amino acid transport system permease protein; AapM, BztC	
K09972	General L-amino acid transporter	General L-amino acid transport system ATP-binding protein; AapP, BztD	EC:7.4.2.1
K10005	Glutamate transporter	Glutamate transport system substrate-binding protein; GluB	
K10006	Glutamate transporter	Glutamate transport system permease protein; GluC	
K10007	Glutamate transporter	Glutamate transport system permease protein; GluD	
K10008	Glutamate transporter	glutamate transport system ATP-binding protein; GluA	EC:7.4.2.1
K02424	Cystine transporter	L-cystine transport system substrate-binding protein; FliY, TcyA	
K10009	Cystine transporter	L-cystine transport system permease protein; TcyB, YecS	
K10010	Cystine transporter	L-cystine transport system ATP-binding protein; TcyC, YecC	EC:7.4.2.1
K16956	L-Cystine transporter	L-cystine transport system substrate-binding protein; TcyJ	
K16957	L-Cystine transporter	L-cystine transport system substrate-binding protein; TcyK	
K16958	L-Cystine transporter	L-cystine transport system permease protein; TcyL	
K16959	L-Cystine transporter	L-cystine transport system permease protein; TcyM	
K16960	L-Cystine transporter	L-cystine transport system ATP-binding protein; TcyN	EC:7.4.2.1
K10022	Arginine/ornithine transporter	Arginine/ornithine transport system substrate-binding protein; AotJ	
K10023	Arginine/ornithine transporter	Arginine/ornithine transport system permease protein; AotM	
K10024	Arginine/ornithine transporter	Arginine/ornithine transport system permease protein; AotQ	
K10025	Arginine/ornithine transporter	Arginine/ornithine transport system ATP-binding protein; AotP	EC:7.4.2.1
K23059	Arginine/lysine/histidine transporter	Arginine/lysine/histidine transporter system substrate-binding protein; ArtP, ArtI	
K17077	Arginine/lysine/histidine transporter	Arginine/lysine/histidine transport system permease protein; ArtQ	
K23060	Arginine/lysine/histidine transporter	arginine/lysine/histidine transport system ATP-binding protein; ArtR, ArtM	EC:7.4.2.1
K01995	Branched-chain amino acid transporter	Branched-chain amino acid transport system ATP-binding protein; LivG	
K01996	Branched-chain amino acid transporter	Branched-chain amino acid transport system ATP-binding protein; LivF	
K01997	Branched-chain amino acid transporter	Branched-chain amino acid transport system permease protein; LivH	
K01998	Branched-chain amino acid transporter	Branched-chain amino acid transport system permease protein; LivM	
K01999	Branched-chain amino acid transporter	Branched-chain amino acid transport system substrate-binding protein; LivK	
K11954	Neutral amino acid transporter	Neutral amino acid transport system substrate-binding protein; NatB	
K11955	Neutral amino acid transporter	Neutral amino acid transport system permease protein; NatC	
K11956	Neutral amino acid transporter	Neutral amino acid transport system permease protein; NatD	
K11957	Neutral amino acid transporter	Neutral amino acid transport system ATP-binding protein; NatA	
K11958	Neutral amino acid transporter	Neutral amino acid transport system ATP-binding protein; NatE	

K02073	D-Methionine transporter	D-methionine transport system substrate-binding protein; MetQ	
K02072	D-Methionine transporter	D-methionine transport system permease protein; MetI	
K02071	D-Methionine transporter	D-methionine transport system ATP-binding protein; MetN	
K15580	Oligopeptide transporter	Oligopeptide transport system substrate-binding protein; OppA, MppA	
K15581	Oligopeptide transporter	Oligopeptide transport system permease protein; OppB	
K15582	Oligopeptide transporter	Oligopeptide transport system permease protein; OppC	
K15583	Oligopeptide transporter	Oligopeptide transport system ATP-binding protein; OppD	
K10823	Oligopeptide transporter	Oligopeptide transport system ATP-binding protein; OppF	
K12368	Dipeptide transporter	Dipeptide transport system substrate-binding protein; DppA	
K12369	Dipeptide transporter	Dipeptide transport system permease protein; DppB	
K12370	Dipeptide transporter	Dipeptide transport system permease protein; DppC	
K12371	Dipeptide transporter	Dipeptide transport system ATP-binding protein; DppD	
K12372	Dipeptide transporter	Dipeptide transport system ATP-binding protein; DppF	
K16199	Dipeptide transporter	Dipeptide transport system substrate-binding protein; DppE	
K16200	Dipeptide transporter	Dipeptide transport system permease protein; DppB1	
K16201	Dipeptide transporter	Dipeptide transport system permease protein; DppC	
K16202	Dipeptide transporter	Dipeptide transport system ATP-binding protein; DppD	
K01216	Licheninase	Licheninase	EC:3.2.1.73
K01199	Glucan endo-1,3-beta-glucosidase	Glucan endo-1,3-beta-D-glucosidase	EC:3.2.1.39
K19891	Glucan endo-1,3-beta-glucosidase	Glucan endo-1,3-beta-glucosidase 1/2/3	EC:3.2.1.39
K19892	Glucan endo-1,3-beta-glucosidase	Glucan endo-1,3-beta-glucosidase 4	EC:3.2.1.39
K19893	Glucan endo-1,3-beta-glucosidase	Glucan endo-1,3-beta-glucosidase 5/6	EC:3.2.1.39
K01190	Beta-galactosidase	Beta-galactosidase; LacZ	EC:3.2.1.23
K12111	Beta-galactosidase	Evolved beta-galactosidase subunit alpha; EbgA	EC:3.2.1.23
K12308	Beta-galactosidase	Beta-galactosidase; LacA, BgaB	EC:3.2.1.23
K12309	Beta-galactosidase	Beta-galactosidase; GLB1, ELNR1	EC:3.2.1.23
K01188	Beta-galactosidase	Beta-glucosidase	EC:3.2.1.21
K05349	Beta-galactosidase	Beta-glucosidase; BglX	EC:3.2.1.21
K05350	Beta-galactosidase	Beta-glucosidase; BglB	EC:3.2.1.21
K01198	Xylan 1,4-beta-xylosidase	Xylan 1,4-beta-xylosidase; XynB	EC:3.2.1.37
K15920	Xylan 1,4-beta-xylosidase	Xylan 1,4-beta-xylosidase; XYL4	EC:3.2.1.37
K22268	Xylan 1,4-beta-xylosidase	Xylan 1,4-beta-xylosidase; XylA	EC:3.2.1.37
K01179	Cellulase/endoglucanase	Endoglucanase	EC:3.2.1.4
K19357	Cellulase/endoglucanase	Cellulase; CELB	EC:3.2.1.4
K20542	Cellulase/endoglucanase	Endoglucanase; BcsZ	EC:3.2.1.4
K01180	Laminarinase	Endo-1,3(4)-beta-glucanase	EC:3.2.1.6
K20846	Carrageenase	Kappa-carrageenase; CgkA	EC:3.2.1.83

K20850	Carrageenase	Iota-carrageenase; CgiA	EC:3.2.1.157
K01219	Agarase	Beta-agarase	EC:3.2.1.81
K20851	Agarase	Alpha-agarase; AgaA	EC:3.2.1.158
K01200	Pullulanase	Pullulanase; PulA	EC:3.2.1.41
K21575	Pullulanase	Neopullulanase; SusA	EC:3.2.1.135
K01177	Beta-amylase	Beta-amylase	EC:3.2.1.2
K01208	Maltogenic alpha-amylase	Cyclomaltodextrinase/maltogenic alpha-amylase/neopullulanase; Cd, Ma, NplT	EC:3.2.1.54, EC:3.2.1.133, EC:3.2.1.135
K05992	Maltogenic alpha-amylase	Maltogenic alpha-amylase; AmyM	EC:3.2.1.133
K22253	Exo-amylase	Glucan 1,4-alpha-maltotetraohydrolase; Mta	EC:3.2.1.60
K01178	Glucoamylase/glucan 1,4-alpha-glucosidase	Glucoamylase; SGA1	EC:3.2.1.3
K12047	Glucoamylase/glucan 1,4-alpha-glucosidase	Maltase-glucoamylase; MGAM	EC:3.2.1.20, EC:3.2.1.3
K21574	Glucoamylase/glucan 1,4-alpha-glucosidase	Glucan 1,4-alpha-glucosidase; SusB	EC:3.2.1.3
K07024	Sucrose-6-phosphatase	Sucrose-6-phosphatase	EC:3.1.3.24
K01193	Beta-fructofuranosidase	Beta-fructofuranosidase; INV, SacA	EC:3.2.1.26
K01225	Cellobiosidase	Cellulose 1,4-beta-cellobiosidase	EC:3.2.1.91
K19668	Cellobiosidase	Cellulose 1,4-beta-cellobiosidase; CbhA	EC:3.2.1.91
K08977	Bacterioruberin	Bisanhydrobacterioruberin hydratase	EC:4.2.1.161
K03821	PHA storage	Polyhydroxyalkanoate synthase subunit PhaC	EC:2.3.1.-
K05973	PHA storage	Poly(3-hydroxybutyrate) depolymerase; PhaZ	EC:3.1.1.75
K01428	Urea catabolism	Urease subunit alpha; UreC	EC:3.5.1.5
K01429	Urea catabolism	Urease subunit beta; UreB	EC:3.5.1.5
K01430	Urea catabolism	Urease subunit gamma; UreA	EC:3.5.1.5
K00111	Glycerol catabolism	Glycerol-3-phosphate dehydrogenase; GlpA, GlpD	EC:1.1.5.3
K00112	Glycerol catabolism	Glycerol-3-phosphate dehydrogenase subunit B; GlpB	EC:1.1.5.3
K00113	Glycerol catabolism	Glycerol-3-phosphate dehydrogenase subunit C; GlpC	EC:1.1.5.3
K00864	Glycerol catabolism	Glycerol kinase; GlpK, GK	EC:2.7.1.30
K00005	Glycerol catabolism	Glycerol dehydrogenase; GldA	EC:1.1.1.6
K00096	Archaeal glycerol synthesis	Glycerol-1-phosphate dehydrogenase [NAD(P)+]	EC:1.1.1.261
K00518	Superoxide dismutase	Nickel superoxide dismutase; SodN	EC:1.15.1.1
K04564	Superoxide dismutase	Superoxide dismutase, Fe-Mn family; SOD2	EC:1.15.1.1
K04565	Superoxide dismutase	Superoxide dismutase, Cu-Zn family; SOD1	EC:1.15.1.1
K16627	Superoxide dismutase	Superoxide dismutase, Cu-Zn family; SOD3	EC:1.15.1.1
K06163	Methylphosphonate catabolism	Alpha-D-ribose 1-methylphosphonate 5-phosphate C-P lyase; PhnJ	EC:4.7.1.1
K06164	Methylphosphonate catabolism	Alpha-D-ribose 1-methylphosphonate 5-triphosphate synthase subunit PhnI	EC:2.7.8.37

K06165	Methylphosphonate catabolism	Alpha-D-ribose 1-methylphosphonate 5-triphosphate synthase subunit PhnH	EC:2.7.8.37
K06166	Methylphosphonate catabolism	Alpha-D-ribose 1-methylphosphonate 5-triphosphate synthase subunit PhnG	EC:2.7.8.37
K05780	Methylphosphonate catabolism	Alpha-D-ribose 1-methylphosphonate 5-triphosphate synthase subunit PhnL	EC:2.7.8.37
K03430	Aminoethylphosphonate catabolism	2-Aminoethylphosphonate-pyruvate transaminase; PhnW	EC:2.6.1.37
K05306	Aminoethylphosphonate catabolism	Phosphonoacetaldehyde hydrolase; PhnX	EC:3.11.1.1
K07306	DMSO reduction	Anaerobic dimethyl sulfoxide reductase subunit A	EC:1.8.5.3
K16953	DMSP catabolism	Dimethylpropiothetin dethiomethylase; DddL	EC:4.4.1.3
K17486	DMSP catabolism	Dimethylsulfoniopropionate demethylase; DmdA	EC:2.1.1.269

APS, adenosine 5'-phosphosulfate; CRISPR, clustered regularly interspaced short palindromic repeats; DMSO, dimethyl sulfoxide; DMSP, dimethylsulfoniopropionate; GSB, green sulfur bacteria; PAPS, 3'-phosphoadenosine-5'-phosphosulfate; PHA, polyhydroxyalkanoates; RC core complex, reaction center core complex; rTCA cycle, reverse tricarboxylic acid cycle; SOX system, sulfur-oxidation system.

References

1. Bowman JP, Nichols CM, Gibson JA. *Algoriphagus ratkowskyi* gen. nov., sp. nov., *Brumimicrobium glaciale* gen. nov., sp. nov., *Cryomorpha ignava* gen. nov., sp. nov. and *Crocinitomix catalasitica* gen. nov., sp. nov., novel flavobacteria isolated from various polar habitats. Int J Syst Evol Microbiol. 2003;53:1343–55.
2. Van Trappen S, Vandecandelaere I, Mergaert J, Swings J. *Algoriphagus antarcticus* sp. nov., a novel psychrophile from microbial mats in Antarctic lakes. Int J Syst Evol Microbiol. 2004;54:1969–73.
3. Chen Z, Lei X, Lai Q, Li Y, Zhang B, Zhang J, et al. *Phaeodactylibacter xiamenensis* gen. nov., sp. nov., a member of the family Saprospiraceae isolated from the marine alga *Phaeodactylum tricornutum*. Int J Syst Evol Microbiol. 2014;64:3496–502.
4. Mavromatis K, Abt B, Brambilla E, Lapidus A, Copeland A, Deshpande S, et al. Complete genome sequence of *Coraliomargarita akajimensis* type strain (04OKA010-24^{T}). Stand Genomic Sci. 2010;2:290–9.
5. Wegner CE, Richter-Heitmann T, Klindworth A, Klockow C, Richter M, Achstetter T, et al. Expression of sulfatases in *Rhodopirellula baltica* and the diversity of sulfatases in the genus *Rhodopirellula*. Mar Genomics. 2013;9:51–61.
6. Lage OM, Bondoso J. Planctomycetes and macroalgae, a striking association. Front Microbiol. 2014;5:267.
7. Geng H, Belas R. Molecular mechanisms underlying roseobacter-phytoplankton symbioses. Curr Opin Biotechnol. 2010;21:332–8.
8. Wirth JS, Whitman WB. Phylogenomic analyses of a clade within the roseobacter group suggest taxonomic reassignments of species of the genera *Aestuariivita*, *Citreicella*, *Loktanella*, *Nautella*, *Pelagibaca*, *Ruegeria*, *Thalassobius*, *Thiobacimonas* and *Tropicibacter*, and the proposal of six novel genera. Int J Syst Evol Microbiol. 2018;68:2393–411.
9. Van Trappen S, Mergaert J, Swings J. *Loktanella salsilacus* gen. nov., sp. nov., *Loktanella fryxellensis* sp. nov. and *Loktanella vestfoldensis* sp. nov., new members of the *Rhodobacter* group, isolated from microbial mats in Antarctic lakes. Int J Syst Evol Microbiol. 2004;54:1263–9.
10. Schwalbach MS, Tripp HJ, Steindler L, Smith DP, Giovannoni SJ. The presence of the glycolysis operon in SAR11 genomes is positively correlated with ocean productivity. Environ Microbiol. 2010;12:490–500.
11. Rankin LM, Gibson JAE, Franzrnann PD, Burton HR. The chemical stratification and microbial communities of Ace Lake, Antarctica: a review of the characteristics of a marine-derived meromictic lake. Polarforschung. 1999;66:33–52.
12. Carbohydrate-Active enZYmes Database. http://www.cazy.org/ (1998). Accessed April 2019 – February 2020.
13. Cantarel BL, Coutinho PM, Rancurel C, Bernard T, Lombard V, Henrissat B. The Carbohydrate-Active EnZymes database (CAZy): an expert resource for glycogenomics. Nucleic Acids Res. 2009;37:D233–8.
14. Leishman MR, Wild C. Vegetation abundance and diversity in relation to soil nutrients and soil water content in Vestfold Hills, East Antarctica. Antarct Sci. 2001;13:126–34.
15. Percival E. The polysaccharides of green, red and brown seaweeds: their basic structure, biosynthesis and function. British Phycol J. 1979;14:103–17.
16. Zogaj X, Nimtz M, Rohde M, Bokranz W, Römling U. The multicellular morphotypes of *Salmonella typhimurium* and *Escherichia coli* produce cellulose as the second component of the extracellular matrix. Mol Microbiol. 2001;39:1452–63.
17. Brown MR. The amino-acid and sugar composition of 16 species of microalgae used in mariculture. J Exp Mar Biol Ecol. 1991;145:79–99.

18. Bunesova V, Lacroix C, Schwab C. Fucosyllactose and L-fucose utilization of infant *Bifidobacterium longum* and *Bifidobacterium kashiwanohense*. BMC Microbiol. 2016;16:248.
19. Silchenko AS, Kusaykin MI, Zakharenko AM, Menshova RV, Khanh HHN, Dmitrenok PS, et al. Endo-1,4-fucoidanase from Vietnamese marine mollusk *Lambis* sp. which producing sulphated fucooligosaccharides. J Mol Catal B Enzym. 2014;102:154–60.
20. Søndergaard M, Schierup HH. Release of extracellular organic carbon during a diatom bloom in Lake Mossø: molecular weight fractionation. Freshw Biol. 1982;12:313–20.
21. Bjørnsen PK. Phytoplankton exudation of organic matter: Why do healthy cells do it? Limnol Oceanogr. 1988;33:151–4.
22. Mincer TJ, Aicher AC. Methanol production by a broad phylogenetic array of marine phytoplankton. PLoS One. 2016;11:e0150820.
23. Wright RT, Shah NM. The trophic role of glycolic acid in coastal seawater. I. Heterotrophic metabolism in seawater and bacterial cultures. Mar Biol. 1975;33:175–83.
24. Rees GN, Harfoot CG, Sheehy AJ. Amino acid degradation by the mesophilic sulfate-reducing bacterium *Desulfobacterium vacuolatum*. Arch Microbiol. 1998;169:76–80.
25. Pelletier E, Kreimeyer A, Bocs S, Rouy Z, Gyapay G, Chouari R, et al. "*Candidatu*s Cloacamonas acidaminovorans": genome sequence reconstruction provides a first glimpse of a new bacterial division. J Bacteriol. 2008;190:2572–79.
26. Heider J, Mai X, Adams MW. Characterization of 2-ketoisovalerate ferredoxin oxidoreductase, a new and reversible coenzyme A-dependent enzyme involved in peptide fermentation by hyperthermophilic archaea. J Bacteriol. 1996;178:780–7.
27. Conover RJ, Gustavson KR. Sources of urea in arctic seas: zooplankton metabolism. Mar Ecol Prog Ser. 1999;179:41–54.
28. Allen AE, Dupont CL, Oborník M, Horák A, Nunes-Nesi A, McCrow JP, et al. Evolution and metabolic significance of the urea cycle in photosynthetic diatoms. Nature. 2011;473:203–7.
29. Gorzynska AK, Denger K, Cook AM, Smits TH. Inducible transcription of genes involved in taurine uptake and dissimilation by *Silicibacter pomeroyi* DSS-3^{T}. Arch Microbiol. 2006;185:402–6.
30. Felux A, Denger K, Weiss M, Cook AM, Schleheck D. *Paracoccus denitrificans* PD1222 utilizes hypotaurine via transamination followed by spontaneous desulfination to yield acetaldehyde and, finally, acetate for growth. J Bacteriol. 2013;195:2921–30.
31. Mayer J, Huhn T, Habeck M, Denger K, Hollemeyer K, Cook AM. 2,3-Dihydroxypropane-1-sulfonate degraded by *Cupriavidus pinatubonensis* JMP134: purification of dihydroxypropanesulfonate 3-dehydrogenase. Microbiol. 2010;156:1556–64.
32. Reisch CR, Moran MA, Whitman WB. Bacterial catabolism of dimethylsulfoniopropionate (DMSP). Front Microbiol. 2011;2:172.
33. Sun J, Todd JD, Thrash JC, Qian Y, Qian MC, Temperton B, et al. The abundant marine bacterium *Pelagibacter* simultaneously catabolizes dimethylsulfoniopropionate to the gases dimethyl sulfide and methanethiol. Nat Microbiol. 2016;1:16065.
34. Keller MD, Kiene RP, Matrai PA, Bellows WK. Production of glycine betaine and dimethylsulfoniopropionate in marine phytoplankton. I. Batch cultures. Mar Biol. 1999;135:237–48.
35. Tripp HJ, Schwalbach MS, Meyer MM, Kitner JB, Breaker RR, Giovannoni SJ. Unique glycine-activated riboswitch linked to glycine-serine auxotrophy in SAR11. Environ Microbiol. 2009;11:230–8.
36. Sun J, Steindler L, Thrash JC, Halsey KH, Smith DP, Carter AE, et al. One carbon metabolism in SAR11 pelagic marine bacteria. PLoS One. 2011;6:e23973.

37. Ticak T, Kountz DJ, Girosky KE, Krzycki JA, Ferguson DJ Jr. A nonpyrrolysine member of the widely distributed trimethylamine methyltransferase family is a glycine betaine methyltransferase. Proc Natl Acad Sci U S A. 2014;111:E4668–76.
38. Oren A. Formation and breakdown of glycine betaine and trimethylamine in hypersaline environments. Antonie van Leeuwenhoek. 1990;58:291–8.
39. Martinez A, Tyson GW, Delong EF. Widespread known and novel phosphonate utilization pathways in marine bacteria revealed by functional screening and metagenomic analyses. Environ Microbiol. 2010;12:222–38.
40. Metcalf WW, Griffin BM, Cicchillo RM, Gao J, Janga SC, Cooke HA, et al. Synthesis of methylphosphonic acid by marine microbes: a source for methane in the aerobic ocean. Science. 2012;337:1104–7.
41. Kamat SS, Williams HJ, Raushel FM. Intermediates in the transformation of phosphonates to phosphate by bacteria. Nature. 2011;480:570–3.
42. Stasi R, Neves HI, Spira B. Phosphate uptake by the phosphonate transport system PhnCDE. BMC Microbiol. 2019;19:79.
43. Villarreal-Chiu JF, Quinn JP, McGrath JW. The genes and enzymes of phosphonate metabolism by bacteria, and their distribution in the marine environment. Front Microbiol. 2012;3:19.
44. Timeanddate.com. https://www.timeanddate.com (1998). Accessed 15 Aug 2019.
45. Parks DH, Imelfort M, Skennerton CT, Hugenholtz P, Tyson GW. CheckM: assessing the quality of microbial genomes recovered from isolates, single cells, and metagenomes. Genome Res. 2015;25:1043–55.
