# Supplementary material for: Influence of the polar light cycle on seasonal dynamics of an Antarctic lake microbial community
Source: Microbiome. 2020 Aug 9;8:116. doi: 10.1186/s40168-020-00889-8 (PMC7416419; doi:10.1186/s40168-020-00889-8)
Supplement: Supplementary file 3 — Additional file 2: Analysis of ‘unassigned’ taxa. Supplementary text, figures and tables. [file 40168_2020_889_MOESM2_ESM.pdf]

Additional file 2

Influence of the polar light cycle on seasonal dynamics of an Antarctic lake microbial community.

Pratibha Panwar, Michelle A. Allen, Timothy J. Williams, Alyce M. Hancock, Sarah Brazendale, James Bevington, Simon Roux, David Paez-Espino, Stephen Nayfach, Maureen Berg, Frederik Schulz, Amy Chen, Marcel Huntemann, Nicole Shapiro, Nikos Kyrpides, Tanja Woyke, Emiley A. Eloe-Fadrosh and Ricardo Cavicchioli

Additional file 2: Analysis of 'unassigned' taxa

Supplementary Text

Contigs with poor taxonomic assignments

Supplementary Figures

Fig. S1 Depth distribution of contigs and OTUs with poor taxonomic assignment.

Supplementary Tables

Table S1 Relative abundance, and genetic and taxonomic composition of unassigned contigs.
Table S2 Domain-level taxonomic composition of 'unassigned contigs' and their peak relative abundances at each depth.

Contigs with poor taxonomic assignments

A large number of contigs could not be confidently taxonomically assigned beyond the level of domain or virus and were grouped together as ‘other’ bacteria, archaea, eukarya or viruses. In addition, a large number of contigs that could not be assigned to the level of domain or virus were grouped together as ‘unassigned’. The total relative abundance of the ‘unassigned’ contigs in the metagenomes from different depths in the lake ranged from 7 – 71% (Additional file 2: Tables S1 and S2). With the exception of 28 contigs, the individual unassigned contigs (total ~1.3 million) had low relative abundance (<1% relative to the sum of coverages of all contigs, including unassigned contigs, in a metagenome).

Among the SSU gene-containing unassigned contigs, most SSU genes had best matches to uncultured bacteria (27 – 67%), with approximately twice as many matches occurring in the lower zone (e.g. L2, 67%) compared to the upper zone (e.g. U2, 35%) (Additional file 2: Table S1). A proportion of SSU genes had best matches to uncultured eukarya (0 – 10%), with the largest number of matches occurring at the surface (Additional file 2: Table S1). This distribution is consistent with representation by size fraction where the upper zone had more unassigned contigs in the 3.0 μm fraction (i.e. likely reflecting the uncultured eukarya), and the lower zone had more unassigned contigs in the 0.1 μm fraction (i.e. likely reflecting the uncultured bacteria) (Additional file 2: Fig. S1). Most ORFs in the unassigned contigs were annotated as hypothetical (57–71%; Additional file 2: Table S1), consistent with the high proportion of matches to uncultured species, while some of the others were annotated as viruses, mobile elements and tRNA (each 1-2%), with their abundances similar across lake depths (Additional file 2: Table S1).

To further interrogate the unassigned contigs, the peak relative abundance was evaluated for contigs that contained SSU genes and unassigned contigs that were identified as viral using VirSorter (Additional file 2: Table S2). This assessment showed that uncharacterized viruses appeared to make a larger contribution (up to 5% peak relative abundance) to specific metagenomes compared to uncharacterized cellular lineages (up to 0.5% peak relative abundance). This dynamic of individual metagenomes (i.e. snapshots in time, depth and size fraction) might reflect an influence of specific viruses on specific cellular lineages in the lake. This inference is consistent with analyses of abundant OTUs identifying *Phycodnaviridae* to contribute to individual metagenomes by as much as 20% peak relative abundance (see main text).

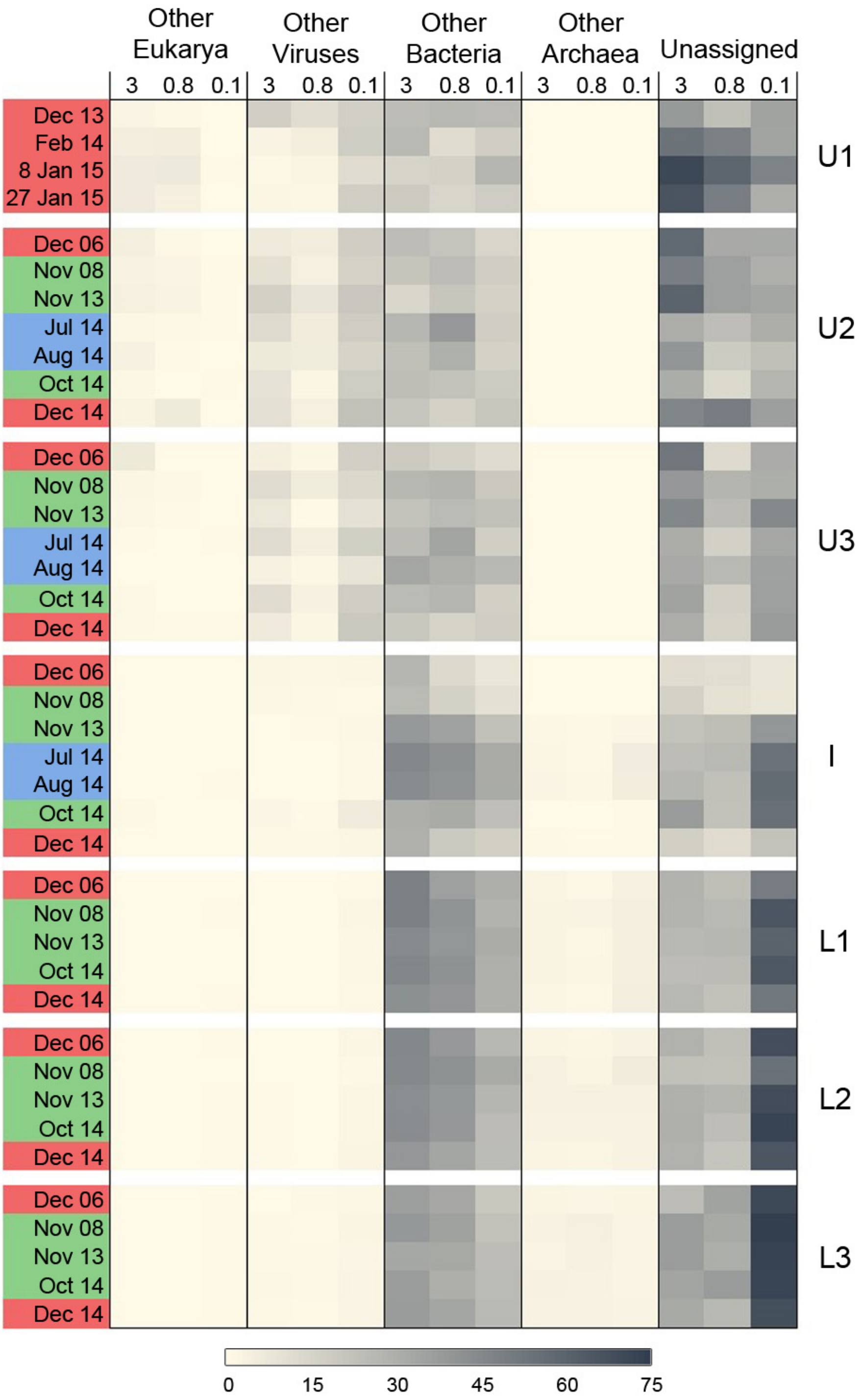

Fig. S1 Depth distribution of contigs and OTUs with poor taxonomic assignment. Heat map depicting the relative abundance of 'Other' Eukarya, Viruses, Bacteria and Archaea OTUs and contigs that could not be assigned any taxonomy (unassigned) (Additional file 1: Dataset S1). The percentage relative abundance (gradient bar) for taxa segregated by filter size (top x-axis: 3, 0.8 and 0.1 μm) displayed by depth (right-hand y-axis, U1 to L3) and by year and season (left-hand y-axis: summer, red; winter, blue; spring, green). Filter sizes: 3, 20–3 μm; 0.8, 3–0.8 μm; 0.1, 0.8–0.1 μm; Depths: U1, upper 1; U2, upper 2; U3, upper 3; I, interface; L1, lower 1; L2, lower 2; L3, lower 3 (see Additional file 1: Table S1 for specific information about sampled depths).

Table S1 Relative abundance, and genetic and taxonomic composition of unassigned contigs.

Depth	% Unassigned relative abundance‡	% Viral genes*	% Hypothetical genes*	% tRNA genes*	% Transposase genes*	16S/18S SSU DNA matches (%)†	
						Uncultured bacteria	Uncultured eukarya
Upper 1	22-67	2	71	2	1	27	10
Upper 2	12-57	1	65	1	1	35	5
Upper 3	12-49	2	62	2	1	34	3
Interface	7-54	1	59	1	1	57	2
Lower 1	21-62	1	57	1	1	65	0
Lower 2	20-69	1	59	1	1	67	0
Lower 3	23-71	2	71	1	1	66	0

‡ The unassigned contig relative abundances were calculated relative to the sum of coverages of all contigs (including unassigned contigs) in a metagenome. * The percentages were calculated relative to total gene annotations in the unassigned contigs in a metagenome. Values were averaged for samples from a depth and are based on data from contigs of length ≥1 kb. † Values for each depth indicate the percentage of 16S SSU genes on unassigned contigs that had matches to uncultured bacteria and the percentage of 18S SSU genes on unassigned contigs that had hits to uncultured eukarya.

Table S2 Domain-level taxonomic composition of 'unassigned contigs' and their peak relative abundances at each depth.

Depth	% Unassigned relative abundance ‡	Peak relative abundance (%) of unassigned contigs with an affiliation to†			
		Archaea	Bacteria	Eukarya	Viruses & Prophages
Upper 1	22-67	0	0.1	0.3	5 & 0.2
Upper 2	12-57	0	0.2	0.1	4 & 0.5
Upper 3	12-49	0	0.5	0.2	3 & 2
Interface	7-54	0.1	0.2	0.04	5 & 1
Lower 1	21-62	0.04	0.2	0	3 & 2
Lower 2	20-69	0.1	0.1	0.001	2 & 1
Lower 3	23-71	0.02	0.1	0	1 & 0.4

‡ The unassigned contig relative abundances were calculated relative to the sum of coverages of all contigs (including unassigned contigs) in a metagenome. † Relative abundances of Archaea-, Bacteria-, and Eukarya-affiliated unassigned contigs were calculated from the SSU gene-containing unassigned contigs with matches to respective taxonomic domains. For example, for % Eukarya, all unassigned contigs containing SSU genes with matches to Eukarya (including uncultured eukarya and any other eukarya) were pooled and their abundance calculated relative to the sum of coverages of all contigs (including unassigned contigs) in a metagenome. Viruses & Prophages relative abundances were calculated from unassigned contigs of length ≥1 kb that VirSorter confidently predicted as viruses (category 1 and 2) and prophages (category 4 and 5), respectively. The percentages were calculated relative to the sum of coverages of all contigs (including unassigned contigs) in a metagenome. The highest percent relative abundance of Archaea, Bacteria, Eukarya, and Viruses & Prophages in a depth were represented as peak relative abundances at respective depths. The peak relative abundances are directly comparable to the peak relative abundances of the OTUs described in the main text.
