# Supplementary material for: Influence of the polar light cycle on seasonal dynamics of an Antarctic lake microbial community
Source: Microbiome. 2020 Aug 9;8:116. doi: 10.1186/s40168-020-00889-8 (PMC7416419; doi:10.1186/s40168-020-00889-8)
Supplement: Supplementary file 4 — Additional file 3: Ace Lake video. Movie and text description. [file 40168_2020_889_MOESM3_ESM.zip › 40168_2020_889_MOESM3_ESM.pdf]

Additional file 3

Influence of the polar light cycle on seasonal dynamics of an Antarctic lake microbial community.

Pratibha Panwar, Michelle A. Allen, Timothy J. Williams, Alyce M. Hancock, Sarah Brazendale, James Bevington, Simon Roux, David Paez-Espino, Stephen Nayfach, Maureen Berg, Frederik Schulz, Amy Chen, Marcel Huntemann, Nicole Shapiro, Nikos Kyrpides, Tanja Woyke, Emiley A. Eloe-Fadrosh and Ricardo Cavicchioli

Additional file 3: Ace Lake video

Supplementary Video

The video was taken from the main sampling hole on Ace Lake Nov 23, 2013, using a Contour +2 video camera (1920 x 1080, 30 frames sec^{-1}) illuminated using a Fantasea Radiant Video light mounted in a custom housing (Fig. S1). The duration and quality of the original video was edited in order to obtain a file size < 20 Mb.

- 0-9 sec: Inside the mobile work shelter with expeditioners Sarah Payne, Alyce Hancock and Rick Cavicchioli (camera operator).
- 9-18 sec: Descending into and down the Ace Lake ice hole.
- 19+ sec: Numerous *Paralabidocera antarctica* copepods are visible from within the ice hole down the water column (~12 m) to the interface.
- 26+ sec: The colour of the image changes from clear blue (upper zone waters) to green-brown as the camera approaches the interface.
- 30-42 sec: Relatively regularly spaced white spots become visible forming a dot pattern on the surface of the interface, which increase in size as the camera descends.
- 43-56 sec: After the camera enters the interface the image loses clarity and the colour becomes green-brown or vivid green caused by the dense (> 10^8 cells ml^{-1}), thick (~1 m) layer of green sulfur bacteria (GSB; *Chlorobium*).
- 57-1:06 sec: As the camera re-emerges from the interface the dotted surface pattern is somewhat visible towards the left had side of the image.
- 67-1:10 sec: A white plume results from the camera being brought towards the lake surface. The copepods appear more active (than during the descent), possibly as a result of the copepods feeding on the GSB which have been brought up as source of food, the presence of the light re-emerging, and/or the physical disturbance created by the camera.
- 1:11-1:14: Camera withdrawn from the ice hole.
- Note: Approximately eight hours after taking the video, the surface layer of the interface appeared to have reformed.

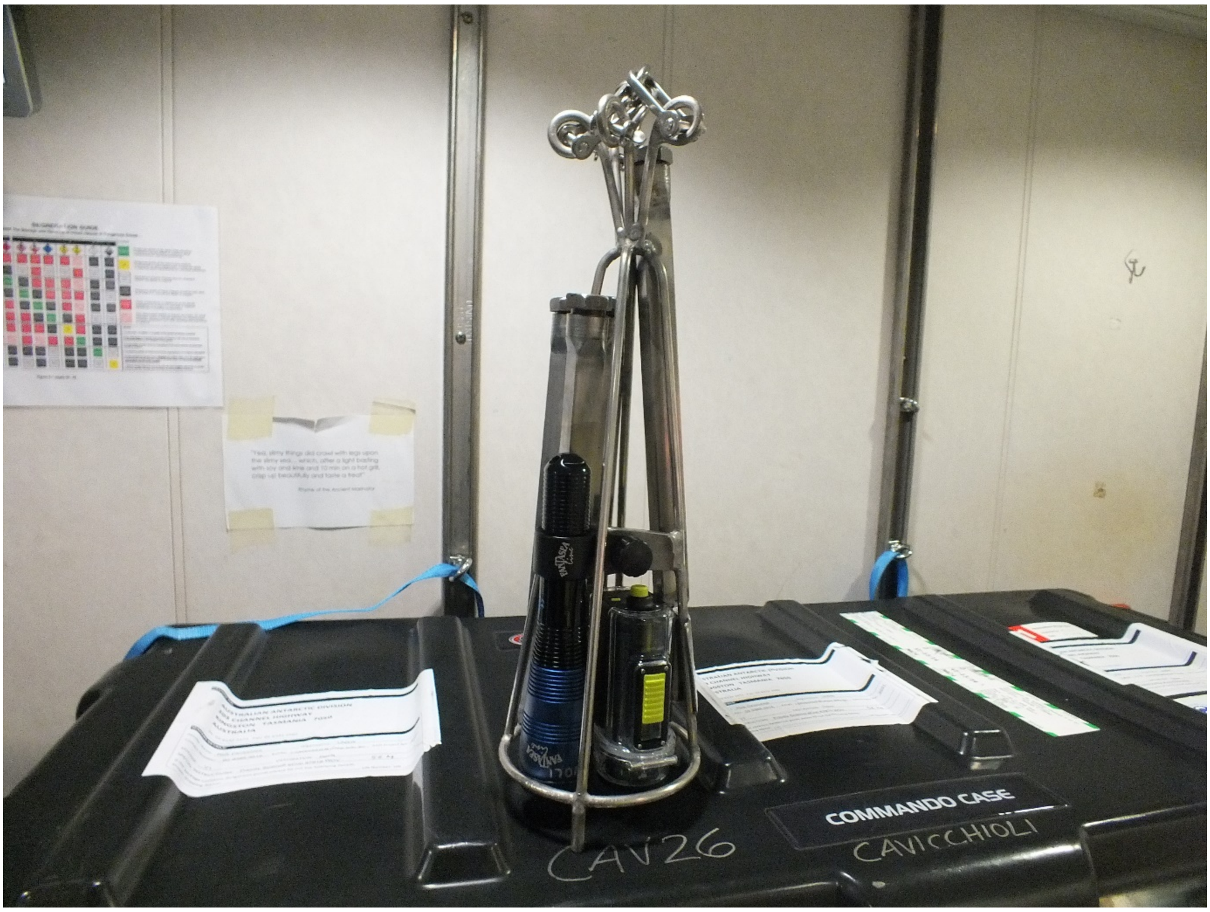

Fig. S1 Video camera and light mounted in housing.
