# Supplementary material for: Influence of the polar light cycle on seasonal dynamics of an Antarctic lake microbial community
Source: Microbiome. 2020 Aug 9;8:116. doi: 10.1186/s40168-020-00889-8 (PMC7416419; doi:10.1186/s40168-020-00889-8)
Supplement: Supplementary file 5 — Additional file 4: Viral analyses. Supplementary tables and dataset. [file 40168_2020_889_MOESM4_ESM.zip › 40168_2020_889_MOESM4_ESM.pdf]

Additional file 4

Influence of the polar light cycle on seasonal dynamics of an Antarctic lake microbial community.

Pratibha Panwar, Michelle A. Allen, Timothy J. Williams, Alyce M. Hancock, Sarah Brazendale, James Bevington, Simon Roux, David Paez-Espino, Stephen Nayfach, Maureen Berg, Frederik Schulz, Amy Chen, Marcel Huntemann, Nicole Shapiro, Nikos Kyrpides, Tanja Woyke, Emiley A. Eloe-Fadrosh and Ricardo Cavicchioli

Additional file 4: Viral analyses

Supplementary Tables

Table S1 Complete, circular virus genomes in Ace Lake.
Table S2 Summary of the virus and NCLDV clusters, singletons and contigs associated with *Phycodnaviridae* 1-5 OTUs.
Table S3 Relative abundance-based correlation between *Micromonas* and *Phycodnaviridae* 1-5 OTUs.
Table S4 Ace Lake cyanophage metadata.
Table S5 *Chlorobium* viruses and their potential hosts.
Table S6 Spacer sequences with matches to two CL1024 and one SG14554 contigs.
Table S7 Host analysis of virus cluster 1024.
Table S8 Most abundant virus clusters in Ace Lake.
Table S9 Metadata associated with *Chlorobium* viruses.
Table S10 Metadata for the cluster 24 'huge' phage.

Dataset

Dataset S1 See accompanying dataset (excel file): Results of vContact clustering of putative Chlorobium phage sequences from Ace Lake with putative phage sequences from Trout Bog Lake.

Table S1 Complete, circular virus genomes in Ace Lake.

Clade[A]	Ace Lake zone	Virus cluster or singleton[B]	GC content	Contig ID	Taxon ID	Metagenome[C] (sample collection date, depth, filter fraction)	Contig length (bp)
Caudovirales	Upper	39	0.4	Ga0208413_1000137	3300025513	Nov 2008_U2_0.8 µm	61,327
				Ga0208414_1000255	3300025603	Nov 2008_U3_0.8 µm	61,327
Caudovirales	Upper	61	0.34	Ga0222634_1000074	3300023235	Nov 2013_U3_0.1 µm	60,667
Caudovirales	Upper	86	0.34	Ga0222644_1000071	3300022841	Dec 2013_U1_3 µm	57,734
				Ga0222646_100034	3300022822	Dec 2013_U1_0.1 µm	57,734
				Ga0222652_1000057	3300022853	Jul 2014_U2_0.1 µm	57,728
				Ga0222711_1000019	3300022837	27Jan 2015_U1_0.1 µm	57,728
				Ga0222668_1000121	3300022865	Oct 2014_U2_3 µm	57,720
				Ga0222674_1000083	3300022848	Oct 2014_U3_3 µm	57,720
Caudovirales	Upper	88	0.36	Ga0222631_1000001	3300022843	Nov 2013_U2_0.1 µm	198,785
Caudovirales	Upper	94	0.59	Ga0222649_1000036	3300022839	Feb 2014_U1_0.1 µm	56,488
Caudovirales	Upper	182	0.48	Ga0222686_1000050	3300023501	Dec 2014_U2_3 µm	61,266
Caudovirales	Upper	190	0.43	Ga0208414_1000068	3300025603	Nov 2008_U3_0.8 µm	116,917
				Ga0222630_1000008	3300023243	Nov 2013_U2_0.8 µm	110,308
				Ga0222645_100024	3300022833	Dec 2013_U1_0.8 µm	110,283
Caudovirales	Upper	294	0.37	Ga0222651_1000297	3300022866	Jul 2014_U2_0.8 µm	35,773
Caudovirales	Upper	355	0.49	Ga0222631_1000054	3300022843	Nov 2013_U2_0.1 µm	34,439
				Ga0222652_1000162	3300022853	Jul 2014_U2_0.1 µm	34,439

Caudovirales	Upper	415	0.47	Ga0222676_1000131	3300023240	Oct 2014_U3_0.1 µm	32,297
				Ga0222654_1000364	3300022836	Jul 2014_U3_0.8 µm	32,288
Caudovirales	Upper	440	0.52	Ga0222648_1000052	3300023054	Feb 2014_U1_0.8 µm	60,542
Caudovirales	Upper	468	0.43	Ga0208768_1000361	3300025601	Nov 2008_U2_3 µm	31,641
Caudovirales	Upper	548	0.33	Ga0222652_1000145	3300022853	Jul 2014_U2_0.1 µm	36,399
				Ga0222659_1000274	3300023236	Aug 2014_U2_3 µm	36,399
				Ga0222660_1000153	3300023239	Aug 2014_U2_0.8 µm	36,399
				Ga0222670_1000118	3300023294	Oct 2014_U2_0.1 µm	36,399
				Ga0222676_1000104	3300023240	Oct 2014_U3_0.1 µm	36,399
				Ga0222689_1000137	3300023231	Dec 2014_U3_3 µm	36,399
				Ga0222711_1000047	3300022837	27Jan 2015_U1_0.1 µm	36,399
Caudovirales	Upper	563	0.34	Ga0222663_1000084	3300022845	Aug 2014_U3_0.8 µm	71,162
				Ga0222676_1000026	3300023240	Oct 2014_U3_0.1 µm	71,162
				Ga0222690_1000051	3300023227	Dec 2014_U3_0.8 µm	71,162
				Ga0222691_1000031	3300022851	Dec 2014_U3_0.1 µm	71,162
Caudovirales	Upper	578	0.39	Ga0208768_1000193	3300025601	Nov 2008_U2_3 µm	46,295
				Ga0222711_1000027	3300022837	27Jan 2015_U1_0.1 µm	43,333
Caudovirales	Upper	614	0.38	Ga0222629_1000113	3300022867	Nov 2013_U2_3 µm	32,140
				Ga0222631_1000063	3300022843	Nov 2013_U2_0.1 µm	32,140
				Ga0222652_1000183	3300022853	Jul 2014_U2_0.1 µm	32,140

				Ga0222661_1000169	3300023229	Aug 2014_U2_0.1 µm	32,140
				Ga0222711_1000069	3300022837	27Jan 2015_U1_0.1 µm	32,138
				Ga0208646_1000327	3300025425	Nov 2008_U2_0.1 µm	31,918
				Ga0208770_1000334	3300025438	Nov 2008_U3_0.1 µm	31,918
Caudovirales	Upper	619	0.34	Ga0222629_1000065	3300022867	Nov 2013_U2_3 µm	40,762
Caudovirales	Upper	685	0.34	Ga0222630_1000110	3300023243	Nov 2013_U2_0.8 µm	41,001
				Ga0222644_1000115	3300022841	Dec 2013_U1_3 µm	41,001
				Ga0222676_1000088	3300023240	Oct 2014_U3_0.1 µm	41,001
				Ga0222688_1000030	3300023293	Dec 2014_U2_0.1 µm	41,001
				Ga0222691_1000082	3300022851	Dec 2014_U3_0.1 µm	41,001
				Ga0222711_1000034	3300022837	27Jan 2015_U1_0.1 µm	41,001
				Ga0222631_1000036	3300022843	Nov 2013_U2_0.1 µm	40,999
				Ga0222652_1000113	3300022853	Jul 2014_U2_0.1 µm	40,988
				Ga0222661_1000105	3300023229	Aug 2014_U2_0.1 µm	40,921
				Ga0222675_1000133	3300023238	Oct 2014_U3_0.8 µm	40,899
Caudovirales	Upper	723	0.6	Ga0222669_1000080	3300022825	Oct 2014_U2_0.8 µm	34,304
Caudovirales	Upper	727	0.3	Ga0222690_1000105	3300023227	Dec 2014_U3_0.8 µm	31,608
Caudovirales	Upper	811	0.38	Ga0222651_1000265	3300022866	Jul 2014_U2_0.8 µm	37,577
				Ga0222652_1000135	3300022853	Jul 2014_U2_0.1 µm	37,577
				Ga0222653_1000212	3300022857	Jul 2014_U3_3 µm	37,577

				Ga0222659_1000254	3300023236	Aug 2014_U2_3 µm	37,577
				Ga0222660_1000148	3300023239	Aug 2014_U2_0.8 µm	37,577
				Ga0222661_1000134	3300023229	Aug 2014_U2_0.1 µm	37,577
				Ga0222668_1000262	3300022865	Oct 2014_U2_3 µm	37,577
				Ga0222669_1000067	3300022825	Oct 2014_U2_0.8 µm	37,577
				Ga0222670_1000107	3300023294	Oct 2014_U2_0.1 µm	37,577
				Ga0222674_1000132	3300022848	Oct 2014_U3_3 µm	37,577
				Ga0222675_1000143	3300023238	Oct 2014_U3_0.8 µm	37,577
				Ga0222676_1000100	3300023240	Oct 2014_U3_0.1 µm	37,577
				Ga0222686_1000106	3300023501	Dec 2014_U2_3 µm	37,577
				Ga0222687_1000052	3300022844	Dec 2014_U2_0.8 µm	37,577
				Ga0222688_1000043	3300023293	Dec 2014_U2_0.1 µm	37,577
Caudovirales	Upper	814	0.5	Ga0222674_1000138	3300022848	Oct 2014_U3_3 µm	36,364
Caudovirales	Upper	834	0.57	Ga0222664_1000268	3300023296	Aug 2014_U3_0.1 µm	36,977
				Ga0222676_1000102	3300023240	Oct 2014_U3_0.1 µm	36,977
Caudovirales	Upper	843	0.43	Ga0208414_1000192	3300025603	Nov 2008_U3_0.8 µm	69,370
				Ga0208646_1000071	3300025425	Nov 2008_U2_0.1 µm	69,370
Caudovirales	Upper	922	0.35	Ga0222634_1000130	3300023235	Nov 2013_U3_0.1 µm	44,118
Caudovirales	Upper	925	0.43	Ga0208646_1000125	3300025425	Nov 2008_U2_0.1 µm	55,014
Caudovirales	Upper	936	0.3	Ga0208768_1000068	3300025601	Nov 2008_U2_3 µm	82,754

				Ga0208646_1000042	3300025425	Nov 2008_U2_0.1 µm	82,750
				Ga0208770_1000054	3300025438	Nov 2008_U3_0.1 µm	82,750
				Ga0208903_1000111	3300025502	Nov 2008_U3_3 µm	82,721
Caudovirales	Upper	960	0.45	Ga0222688_1000057	3300023293	Dec 2014_U2_0.1 µm	33,844
Caudovirales	Upper	961	0.35	Ga0222644_1000054	3300022841	Dec 2013_U1_3 µm	65,450
Caudovirales	Upper	1134	0.56	Ga0222652_1000107	3300022853	Jul 2014_U2_0.1 µm	42,580
Caudovirales	Upper	1234	0.45	Ga0222649_1000109	3300022839	Feb 2014_U1_0.1 µm	32,238
Caudovirales	Upper	1255	0.45	Ga0208414_1000468	3300025603	Nov 2008_U3_0.8 µm	40,133
				Ga0208646_1000215	3300025425	Nov 2008_U2_0.1 µm	40,133
				Ga0208768_1000236	3300025601	Nov 2008_U2_3 µm	40,133
				Ga0208770_1000240	3300025438	Nov 2008_U3_0.1 µm	40,133
				Ga0208903_1000351	3300025502	Nov 2008_U3_3 µm	40,133
Caudovirales	Upper	1303	0.46	Ga0222652_1000051	3300022853	Jul 2014_U2_0.1 µm	59,036
				Ga0222688_1000009	3300023293	Dec 2014_U2_0.1 µm	59,036
				Ga0222691_1000045	3300022851	Dec 2014_U3_0.1 µm	59,036
				Ga0222661_1000055	3300023229	Aug 2014_U2_0.1 µm	58,976
Caudovirales	Upper	1389	0.56	Ga0222633_1000195	3300022847	Nov 2013_U3_0.8 µm	67,914
				Ga0222632_1000099	3300022842	Nov 2013_U3_3 µm	67,839
				Ga0222634_1000056	3300023235	Nov 2013_U3_0.1 µm	67,839
Caudovirales	Upper	1609	0.41	Ga0208770_1000293	3300025438	Nov 2008_U3_0.1 µm	35,550

				Ga0208646_1000275	3300025425	Nov 2008_U2_0.1 µm	35,530
Caudovirales	Upper	1614	0.56	Ga0208414_1000465	3300025603	Nov 2008_U3_0.8 µm	40,262
Caudovirales	Upper	1687	0.36	Ga0222649_1000033	3300022839	Feb 2014_U1_0.1 µm	60,587
Caudovirales	Upper	1925	0.37	Ga0222670_1000134	3300023294	Oct 2014_U2_0.1 µm	34,611
Caudovirales	Upper	1931	0.4	Ga0222629_1000098	3300022867	Nov 2013_U2_3 µm	35,224
				Ga0222631_1000049	3300022843	Nov 2013_U2_0.1 µm	35,224
				Ga0222652_1000154	3300022853	Jul 2014_U2_0.1 µm	35,224
Caudovirales	Upper	2074	0.55	Ga0222632_1000124	3300022842	Nov 2013_U3_3 µm	57,505
Caudovirales	Upper	2122	0.36	Ga0222651_1000121	3300022866	Jul 2014_U2_0.8 µm	58,728
Caudovirales	Upper	2251	0.44	Ga0222633_1000388	3300022847	Nov 2013_U3_0.8 µm	42,580
Caudovirales	Upper	2260	0.31	Ga0222660_1000154	3300023239	Aug 2014_U2_0.8 µm	36,315
Caudovirales	Upper	2466	0.42	Ga0208770_1000066	3300025438	Nov 2008_U3_0.1 µm	75,636
Caudovirales	Upper	2664	0.42	Ga0222646_100080	3300022822	Dec 2013_U1_0.1 µm	35,853
				Ga0222652_1000149	3300022853	Jul 2014_U2_0.1 µm	35,853
				Ga0222661_1000141	3300023229	Aug 2014_U2_0.1 µm	35,853
				Ga0222670_1000120	3300023294	Oct 2014_U2_0.1 µm	35,853
				Ga0222688_1000048	3300023293	Dec 2014_U2_0.1 µm	35,853
				Ga0222711_1000048	3300022837	27Jan 2015_U1_0.1 µm	35,853
Caudovirales	Upper	3129	0.33	Ga0222650_1000104	3300023237	Jul 2014_U2_3 µm	76,097
Caudovirales	Upper	3153	0.33	Ga0222631_1000051	3300022843	Nov 2013_U2_0.1 µm	34,975

				Ga0222652_1000157	3300022853	Jul 2014_U2_0.1 µm	34,975
Caudovirales	Upper	3169	0.39	Ga0222688_1000054	3300023293	Dec 2014_U2_0.1 µm	35,117
Caudovirales	Upper	3187	0.46	Ga0222650_1000438	3300023237	Jul 2014_U2_3 µm	32,633
				Ga0222652_1000180	3300022853	Jul 2014_U2_0.1 µm	32,633
Caudovirales	Upper	3592	0.33	Ga0208414_1000754	3300025603	Nov 2008_U3_0.8 µm	28,792
Caudovirales	Upper	3890	0.38	Ga0222691_1000046	3300022851	Dec 2014_U3_0.1 µm	58,779
Caudovirales	Upper	3895	0.32	Ga0222691_1000080	3300022851	Dec 2014_U3_0.1 µm	41,353
Caudovirales	Upper	3927	0.33	Ga0222649_1000115	3300022839	Feb 2014_U1_0.1 µm	30,893
				Ga0222644_1000166	3300022841	Dec 2013_U1_3 µm	30,887
Caudovirales	Upper	3933	0.38	Ga0222650_1000363	3300023237	Jul 2014_U2_3 µm	37,577
				Ga0222654_1000296	3300022836	Jul 2014_U3_0.8 µm	37,577
				Ga0222655_1000141	3300023245	Jul 2014_U3_0.1 µm	37,577
				Ga0222630_1000128	3300023243	Nov 2013_U2_0.8 µm	37,509
Caudovirales	Upper	3945	0.53	Ga0222689_1000107	3300023231	Dec 2014_U3_3 µm	46,233
Caudovirales	Upper	3980	0.62	Ga0222670_1000135	3300023294	Oct 2014_U2_0.1 µm	34,523
				Ga0222688_1000055	3300023293	Dec 2014_U2_0.1 µm	34,523
				Ga0222661_1000151	3300023229	Aug 2014_U2_0.1 µm	34,520
Caudovirales	Upper	4928	0.38	Ga0222629_1000080	3300022867	Nov 2013_U2_3 µm	37,577
Caudovirales	Upper	4964	0.42	Ga0222646_100030	3300022822	Dec 2013_U1_0.1 µm	61,566
Caudovirales	Upper	4974	0.31	Ga0222707_1000108	3300022832	8Jan 2015_U1_0.8 µm	35,794

				Ga0222708_1000050	3300023242	8Jan 2015_U1_0.1 µm	35,794
				Ga0222711_1000050	3300022837	27Jan 2015_U1_0.1 µm	35,794
Caudovirales	Upper	4978	0.32	Ga0222646_100095	3300022822	Dec 2013_U1_0.1 µm	31,728
Unknown	Upper	5051	0.56	Ga0222691_1000044	3300022851	Dec 2014_U3_0.1 µm	59,477
Caudovirales	Upper	6587	0.63	Ga0222708_1000007	3300023242	8Jan 2015_U1_0.1 µm	76,295
Caudovirales	Upper	6662	0.4	Ga0222646_100076	3300022822	Dec 2013_U1_0.1 µm	36,531
Caudovirales	Upper	6676	0.33	Ga0222629_1000100	3300022867	Nov 2013_U2_3 µm	34,975
Caudovirales	Upper	6750	0.37	Ga0222664_1000144	3300023296	Aug 2014_U3_0.1 µm	55,212
Caudovirales	Upper	8545	0.39	Ga0208768_1000228	3300025601	Nov 2008_U2_3 µm	40,839
Caudovirales	Upper	9600	0.41	Ga0222689_1000136	3300023231	Dec 2014_U3_3 µm	36,717
Caudovirales	Upper	9840	0.59	Ga0222644_1000101	3300022841	Dec 2013_U1_3 µm	44,811
Caudovirales	Upper	10110	0.56	Ga0222707_1000112	3300022832	8Jan 2015_U1_0.8 µm	35,037
Caudovirales	Upper	10239	0.56	Ga0222670_1000558	3300023294	Oct 2014_U2_0.1 µm	15,420
Caudovirales	Upper	sg_8813	0.51	Ga0222646_100052	3300022822	Dec 2013_U1_0.1 µm	44,962
Caudovirales	Upper	sg_8814	0.59	Ga0222646_100054	3300022822	Dec 2013_U1_0.1 µm	44,811
Caudovirales	Upper	sg_8907	0.59	Ga0222645_100134	3300022833	Dec 2013_U1_0.8 µm	44,811
Unknown	Upper	sg_9264	0.53	Ga0222691_1000034	3300022851	Dec 2014_U3_0.1 µm	68,930
Caudovirales	Upper	sg_9323	0.62	Ga0222652_1000161	3300022853	Jul 2014_U2_0.1 µm	34,523
Caudovirales	Upper	sg_9693	0.48	Ga0222662_1000451	3300022885	Aug 2014_U3_3 µm	33,379
Unknown	Upper		0.55	Ga0222634_1000134	3300023235	Nov 2013_U3_0.1 µm	43,608

Caudovirales	Upper		0.29	Ga0222629_1000190	3300022867	Nov 2013_U2_3 µm	23,953
				Ga0222630_1000353	3300023243	Nov 2013_U2_0.8 µm	23,953
				Ga0222649_1000173	3300022839	Feb 2014_U1_0.1 µm	23,953
				Ga0222650_1000687	3300023237	Jul 2014_U2_3 µm	23,953
				Ga0222652_1000292	3300022853	Jul 2014_U2_0.1 µm	23,953
				Ga0222655_1000302	3300023245	Jul 2014_U3_0.1 µm	23,953
				Ga0222661_1000259	3300023229	Aug 2014_U2_0.1 µm	23,953
				Ga0222670_1000261	3300023294	Oct 2014_U2_0.1 µm	23,953
				Ga0222676_1000205	3300023240	Oct 2014_U3_0.1 µm	23,953
Retrovirales	Upper		0.52	Ga0222647_1001013	3300022827	Feb 2014_U1_3 µm	9,276
Microviridae	Upper		0.49	Ga0222662_1004683	3300022885	Aug 2014_U3_3 µm	4,292
				Ga0222663_1002047	3300022845	Aug 2014_U3_0.8 µm	4,292
				Ga0222675_1003300	3300023238	Oct 2014_U3_0.8 µm	4,292
				Ga0222690_1001523	3300023227	Dec 2014_U3_0.8 µm	4,292
CressDNAParvo	Upper		0.5	Ga0222633_1005399	3300022847	Nov 2013_U3_0.8 µm	3,562
CressDNAParvo	Upper		0.49	Ga0222648_1005368	3300023054	Feb 2014_U1_0.8 µm	3,536
CressDNAParvo	Upper		0.51	Ga0222645_105175	3300022833	Dec 2013_U1_0.8 µm	3,122
CressDNAParvo	Upper		0.5	Ga0222630_1006513	3300023243	Nov 2013_U2_0.8 µm	3,115
CressDNAParvo	Upper		0.49-0.5	Ga0222633_1006260	3300022847	Nov 2013_U3_0.8 µm	3,081
				Ga0222644_1002881	3300022841	Dec 2013_U1_3 µm	3,081

				Ga0222645_105245	3300022833	Dec 2013_U1_0.8 µm	3,081
CressDNAParvo	Upper		0.39	Ga0208413_1024040	3300025513	Nov 2008_U2_0.8 µm	2,137
				Ga0208768_1018460	3300025601	Nov 2008_U2_3 µm	2,137
				Ga0222648_1012621	3300023054	Feb 2014_U1_0.8 µm	2,137
				Ga0222668_1009307	3300022865	Oct 2014_U2_3 µm	2,137
				Ga0222707_1004304	3300022832	8Jan 2015_U1_0.8 µm	2,137
				Ga0222710_1007623	3300023429	27Jan 2015_U1_0.8 µm	2,137
				Ga0222644_1005372	3300022841	Dec 2013_U1_3 µm	2,074
Unknown	Upper		0.47-0.48	Ga0222686_1006371	3300023501	Dec 2014_U2_3 µm	2,033
				Ga0222687_1009818	3300022844	Dec 2014_U2_0.8 µm	2,033
				Ga0222690_1005126	3300023227	Dec 2014_U3_0.8 µm	2,033
Caudovirales	Upper, Interface	711	0.61	Ga0208414_1000366	3300025603	Nov 2008_U3_0.8 µm	48,437
				Ga0208770_1000171	3300025438	Nov 2008_U3_0.1 µm	48,437
				Ga0208903_1000269	3300025502	Nov 2008_U3_3 µm	48,437
				Ga0222673_1000112	3300022821	Oct 2014_I_0.1 µm	48,437
Caudovirales	Upper, Interface	1926	0.42	Ga0222631_1000047	3300022843	Nov 2013_U2_0.1 µm	35,853
				Ga0222634_1000201	3300023235	Nov 2013_U3_0.1 µm	35,853
				Ga0222664_1000276	3300023296	Aug 2014_U3_0.1 µm	35,853
				Ga0222676_1000105	3300023240	Oct 2014_U3_0.1 µm	35,853
				Ga0222691_1000108	3300022851	Dec 2014_U3_0.1 µm	35,853

Caudovirales	Upper, Interface	5848	0.41	Ga0222671_1000145	3300022856	Oct 2014_I_3 µm	36,717
				Ga0222674_1000135	3300022848	Oct 2014_U3_3 µm	36,717
Caudovirales	Interface	169	0.52	Ga0222673_1000089	3300022821	Oct 2014_I_0.1 µm	56,215
Caudovirales	Interface	388	0.39	Ga0222667_1000128	3300022890	Aug 2014_I_0.1 µm	43,076
Caudovirales	Interface	735	0.34	Ga0222628_1000134	3300022871	Nov 2013_I_0.1 µm	37,031
Caudovirales	Interface	868	0.39	Ga0222658_1000119	3300023257	Jul 2014_I_0.1 µm	49,083
				Ga0222667_1000096	3300022890	Aug 2014_I_0.1 µm	49,083
Caudovirales	Interface	1230	0.39	Ga0222664_1000178	3300023296	Aug 2014_U3_0.1 µm	47,643
				Ga0222673_1000118	3300022821	Oct 2014_I_0.1 µm	47,643
Caudovirales	Interface	1480	0.53	Ga0222665_1000014	3300022864	Aug 2014_I_3 µm	116,028
Caudovirales	Interface	2193	0.46	Ga0222666_1000279	3300024048	Aug 2014_I_0.8 µm	34,155
Caudovirales	Interface	2653	0.34	Ga0222673_1000073	3300022821	Oct 2014_I_0.1 µm	62,977
Caudovirales	Interface	2987	0.42	Ga0222667_1000147	3300022890	Aug 2014_I_0.1 µm	40,847
Caudovirales	Interface	3162	0.58	Ga0222676_1000050	3300023240	Oct 2014_U3_0.1 µm	57,415
Caudovirales	Interface	3886	0.33	Ga0222628_1000116	3300022871	Nov 2013_I_0.1 µm	39,121
Caudovirales	Interface	3903	0.3	Ga0222628_1000072	3300022871	Nov 2013_I_0.1 µm	49,428
Caudovirales	Interface	4460	0.3	Ga0208647_1000090	3300025362	Nov 2008_I_0.1 µm	39,252
				Ga0208901_1000202	3300025380	Nov 2008_I_0.8 µm	39,252
Caudovirales	Interface	4998	0.36	Ga0222694_1000023	3300023292	Dec 2014_I_0.1 µm	46,925
Caudovirales	Interface	6611	0.41	Ga0222673_1000151	3300022821	Oct 2014_I_0.1 µm	41,192

Caudovirales	Interface	6749	0.59	Ga0222673_1000068	3300022821	Oct 2014_I_0.1 µm	64,142
Caudovirales	Interface	8415	0.31	Ga0222671_1000142	3300022856	Oct 2014_I_3 µm	37,783
Caudovirales	Interface	8461	0.4	Ga0208647_1000095	3300025362	Nov 2008_I_0.1 µm	38,594
Caudovirales	Interface	8535	0.52	Ga0208900_1000079	3300025433	Nov 2008_I_3 µm	57,510
Unknown	Interface	sg_10466	0.31	Ga0222694_1000048	3300023292	Dec 2014_I_0.1 µm	35,253
Caudovirales	Interface	sg_8715	0.34	Ga0222673_1000129	3300022821	Oct 2014_I_0.1 µm	45,604
Caudovirales	Interface, Lower	116	0.31	Ga0222626_1000295	3300022882	Nov 2013_I_3 µm	32,335
				Ga0222657_1000246	3300023241	Jul 2014_I_0.8 µm	32,281
				Ga0222696_1000266	3300023233	Dec 2014_L1_0.8 µm	32,200
Caudovirales	Interface, Lower	540	0.34	Ga0222637_1000124	3300023435	Nov 2013_L1_0.1 µm	42,243
				Ga0222667_1000132	3300022890	Aug 2014_I_0.1 µm	42,243
				Ga0222697_1000106	3300022868	Dec 2014_L1_0.1 µm	42,243
Caudovirales	Interface, Lower	714	0.32	Ga0208904_1000360	3300025669	Nov 2008_L2_0.1 µm	39,410
				Ga0222628_1000114	3300022871	Nov 2013_I_0.1 µm	39,366
				Ga0222664_1000244	3300023296	Aug 2014_U3_0.1 µm	39,366
				Ga0222667_1000156	3300022890	Aug 2014_I_0.1 µm	39,366
				Ga0222697_1000123	3300022868	Dec 2014_L1_0.1 µm	39,366
				Ga0222637_1000143	3300023435	Nov 2013_L1_0.1 µm	39,308
				Ga0222694_1000034	3300023292	Dec 2014_I_0.1 µm	39,294
Caudovirales	Interface,	738	0.38	Ga0208904_1000278	3300025669	Nov 2008_L2_0.1 µm	44,769

	Lower			Ga0222637_1000115	3300023435	Nov 2013_L1_0.1 µm	43,214
				Ga0222658_1000155	3300023257	Jul 2014_I_0.1 µm	43,214
				Ga0222667_1000127	3300022890	Aug 2014_I_0.1 µm	43,214
				Ga0222679_1000066	3300022858	Oct 2014_L1_0.1 µm	43,214
				Ga0222697_1000099	3300022868	Dec 2014_L1_0.1 µm	43,214
Caudovirales	Interface, Lower	753	0.34	Ga0222657_1000100	3300023241	Jul 2014_I_0.8 µm	63,847
				Ga0222658_1000072	3300023257	Jul 2014_I_0.1 µm	63,847
				Ga0222667_1000072	3300022890	Aug 2014_I_0.1 µm	63,847
				Ga0222673_1000070	3300022821	Oct 2014_I_0.1 µm	63,847
				Ga0222694_1000013	3300023292	Dec 2014_I_0.1 µm	63,847
				Ga0222697_1000052	3300022868	Dec 2014_L1_0.1 µm	63,847
Caudovirales	Interface, Lower	782	0.31-0.33	Ga0222658_1000118	3300023257	Jul 2014_I_0.1 µm	49,360
				Ga0208647_1000101	3300025362	Nov 2008_I_0.1 µm	37,783
				Ga0208904_1000394	3300025669	Nov 2008_L2_0.1 µm	37,783
				Ga0222673_1000179	3300022821	Oct 2014_I_0.1 µm	37,783
				Ga0222697_1000133	3300022868	Dec 2014_L1_0.1 µm	37,783
Caudovirales	Interface, Lower	1928	0.4	Ga0222628_1000130	3300022871	Nov 2013_I_0.1 µm	37,186
				Ga0222637_1000162	3300023435	Nov 2013_L1_0.1 µm	37,186
				Ga0222667_1000169	3300022890	Aug 2014_I_0.1 µm	37,186
				Ga0222679_1000092	3300022858	Oct 2014_L1_0.1 µm	37,186

				Ga0222697_1000138	3300022868	Dec 2014_L1_0.1 µm	37,186
Caudovirales	Interface, Lower	2172	0.41	Ga0208904_1000242	3300025669	Nov 2008_L2_0.1 µm	47,696
				Ga0222658_1000126	3300023257	Jul 2014_I_0.1 µm	47,696
Caudovirales	Interface, Lower		0.39	Ga0222626_1000161	3300022882	Nov 2013_I_3 µm	47,710
				Ga0222627_1000111	3300023244	Nov 2013_I_0.8 µm	47,710
				Ga0222636_1000113	3300022854	Nov 2013_L1_0.8 µm	47,710
				Ga0222666_1000189	3300024048	Aug 2014_I_0.8 µm	47,710
				Ga0222667_1000103	3300022890	Aug 2014_I_0.1 µm	47,710
				Ga0222696_1000167	3300023233	Dec 2014_L1_0.8 µm	47,710
Caudovirales	Lower	24	0.56	Ga0208769_1000001	3300025697	Nov 2008_L1_0.1 µm	528,260
				Ga0222679_1000001	3300022858	Oct 2014_L1_0.1 µm	528,258
				Ga0222682_1000001	3300023246	Oct 2014_L2_0.1 µm	528,256
Caudovirales	Lower	82	0.51	Ga0222635_1000049	3300023234	Nov 2013_L1_3 µm	65,245
Caudovirales	Lower	113	0.4	Ga0208769_1000003	3300025697	Nov 2008_L1_0.1 µm	185,273
				Ga0208771_1000009	3300025698	Nov 2008_L3_3 µm	185,273
				Ga0208905_1000005	3300025661	Nov 2008_L3_0.8 µm	185,273
Caudovirales	Lower	205	0.4	Ga0207996_1000318	3300025586	Nov 2008_L2_0.8 µm	44,112
				Ga0208648_1000425	3300025642	Nov 2008_L2_3 µm	44,112
Caudovirales	Lower	311	0.44	Ga0208902_1000117	3300025628	Nov 2008_L1_0.8 µm	48,707
				Ga0208279_1000257	3300025649	Nov 2008_L1_3 µm	48,534

Caudovirales	Lower	740	0.36	Ga0208771_1000154	3300025698	Nov 2008_L3_3 µm	43,313
Caudovirales	Lower	866	0.55	Ga0208904_1000201	3300025669	Nov 2008_L2_0.1 µm	51,617
				Ga0222640_1000018	3300023297	Nov 2013_L2_0.1 µm	51,617
				Ga0222679_1000040	3300022858	Oct 2014_L1_0.1 µm	51,617
Caudovirales	Lower	872	0.31	Ga0222696_1000206	3300023233	Dec 2014_L1_0.8 µm	40,169
Caudovirales	Lower	914	0.39	Ga0208904_1000193	3300025669	Nov 2008_L2_0.1 µm	52,303
				Ga0222679_1000038	3300022858	Oct 2014_L1_0.1 µm	52,303
Caudovirales	Lower	1040	0.42	Ga0208769_1000193	3300025697	Nov 2008_L1_0.1 µm	32,656
Caudovirales	Lower	1137	0.35	Ga0207996_1000364	3300025586	Nov 2008_L2_0.8 µm	41,358
Caudovirales	Lower	1152	0.36	Ga0222685_1000017	3300022874	Oct 2014_L3_0.1 µm	36,298
Caudovirales	Lower	1153	0.36	Ga0208769_1000085	3300025697	Nov 2008_L1_0.1 µm	44,334
Caudovirales	Lower	1270	0.31	Ga0222637_1000055	3300023435	Nov 2013_L1_0.1 µm	59,445
Caudovirales	Lower	1274	0.51	Ga0208769_1000038	3300025697	Nov 2008_L1_0.1 µm	68,986
Caudovirales	Lower	1424	0.34	Ga0208905_1000064	3300025661	Nov 2008_L3_0.8 µm	51,499
Caudovirales	Lower	1429	0.34	Ga0208771_1000215	3300025698	Nov 2008_L3_3 µm	36,811
				Ga0208905_1000127	3300025661	Nov 2008_L3_0.8 µm	36,811
Caudovirales	Lower	1640	0.4	Ga0208904_1000412	3300025669	Nov 2008_L2_0.1 µm	36,601
Caudovirales	Lower	1869	0.39	Ga0222637_1000130	3300023435	Nov 2013_L1_0.1 µm	41,872
				Ga0208904_1000324	3300025669	Nov 2008_L2_0.1 µm	41,796
Caudovirales	Lower	1870	0.39	Ga0222637_1000047	3300023435	Nov 2013_L1_0.1 µm	65,207

Caudovirales	Lower	1875	0.41	Ga0208769_1000105	3300025697	Nov 2008_L1_0.1 µm	42,085
Caudovirales	Lower	1882	0.39	Ga0208769_1000053	3300025697	Nov 2008_L1_0.1 µm	58,972
Caudovirales	Lower	2151	0.36	Ga0208771_1001592	3300025698	Nov 2008_L3_3 µm	11,801
				Ga0208905_1001478	3300025661	Nov 2008_L3_0.8 µm	11,801
				Ga0307928_10003616	3300031227	Nov 2013_L3_0.1 µm	11,801
Caudovirales	Lower	2535	0.39	Ga0222679_1000074	3300022858	Oct 2014_L1_0.1 µm	41,354
Caudovirales	Lower	2539	0.32	Ga0222697_1000080	3300022868	Dec 2014_L1_0.1 µm	48,241
Unknown	Lower	2543	0.34	Ga0207997_1000146	3300025736	Nov 2008_L3_0.1 µm	33,354
Caudovirales	Lower	2955	0.42	Ga0208648_1000482	3300025642	Nov 2008_L2_3 µm	40,949
				Ga0207996_1000375	3300025586	Nov 2008_L2_0.8 µm	40,946
Caudovirales	Lower	4777	0.33	Ga0307928_10000216	3300031227	Nov 2013_L3_0.1 µm	40,506
Caudovirales	Lower	6083	0.56	Ga0208904_1000262	3300025669	Nov 2008_L2_0.1 µm	46,043
Caudovirales	Lower	6100	0.42	Ga0208904_1000286	3300025669	Nov 2008_L2_0.1 µm	43,985
Caudovirales	Lower	6184	0.49	Ga0307928_10000238	3300031227	Nov 2013_L3_0.1 µm	39,571
Caudovirales	Lower	6251	0.31	Ga0307928_10000150	3300031227	Nov 2013_L3_0.1 µm	44,919
Caudovirales	Lower	6647	0.36	Ga0307928_10000232	3300031227	Nov 2013_L3_0.1 µm	39,766
Caudovirales	Upper	8417	0.32	Ga0222634_1000217	3300023235	Nov 2013_U3_0.1 µm	33,882
Caudovirales	Lower	8655	0.4	Ga0208902_1000132	3300025628	Nov 2008_L1_0.8 µm	46,752
Caudovirales	Lower	8662	0.48	Ga0208648_1000620	3300025642	Nov 2008_L2_3 µm	34,039
Caudovirales	Lower	9532	0.54	Ga0307928_10000040	3300031227	Nov 2013_L3_0.1 µm	67,356

Caudovirales	Lower	10290	0.34	Ga0222679_1000185	3300022858	Oct 2014_L1_0.1 µm	25,884
				Ga0222637_1000264	3300023435	Nov 2013_L1_0.1 µm	25,839
Unknown	Lower	sg_10366	0.34	Ga0222703_1000045	3300023256	Dec 2014_L3_0.1 µm	33,260
Caudovirales	Lower	sg_11172	0.35	Ga0307928_10003248	3300031227	Nov 2013_L3_0.1 µm	12,412
Caudovirales	Lower	sg_11648	0.56	Ga0307928_10000234	3300031227	Nov 2013_L3_0.1 µm	39,713
Caudovirales	Lower	sg_2	0.6	Ga0222636_1000107	3300022854	Nov 2013_L1_0.8 µm	48,758
Caudovirales	Lower	sg_550	0.39	Ga0208904_1000255	3300025669	Nov 2008_L2_0.1 µm	46,474
Caudovirales	Lower	sg_576	0.39	Ga0208904_1000272	3300025669	Nov 2008_L2_0.1 µm	45,040
Caudovirales	Lower	sg_637	0.31	Ga0208904_1000246	3300025669	Nov 2008_L2_0.1 µm	47,285
Caudovirales	Lower	sg_9367	0.32	Ga0222679_1000111	3300022858	Oct 2014_L1_0.1 µm	34,677
Caudovirales	Lower		0.37	Ga0207997_1000112	3300025736	Nov 2008_L3_0.1 µm	37,866
				Ga0307928_10000272	3300031227	Nov 2013_L3_0.1 µm	37,866
Caudovirales	Lower		0.31	Ga0222640_1000087	3300023297	Nov 2013_L2_0.1 µm	31,376
Caudovirales	Lower		0.35	Ga0207997_1000616	3300025736	Nov 2008_L3_0.1 µm	18,059
Caudovirales	Lower		0.41	Ga0207997_1001313	3300025736	Nov 2008_L3_0.1 µm	12,882
				Ga0307928_10003015	3300031227	Nov 2013_L3_0.1 µm	12,882
Caudovirales	Lower		0.43	Ga0307928_10003459	3300031227	Nov 2013_L3_0.1 µm	12,048
Caudovirales	Lower		0.35	Ga0222697_1000929	3300022868	Dec 2014_L1_0.1 µm	11,901
Caudovirales	Lower		0.44	Ga0207997_1001542	3300025736	Nov 2008_L3_0.1 µm	11,831

[A] A total of 337 contigs representing complete, circular virus genomes were assigned virus clades. Based on their GC content, length, virus cluster and alignment, they were classified into 173 distinct viruses (all shown here). The virus clades are: *Caudovirales* (158); *Microviridae* (1); *Retrovirales* (1); Cress-DNA virus/Parvovirus (6); unknown (7). Viruses belonging to *Microviridae*, *Retrovirales* and Cress-DNA virus/Parvovirus were detected only in the upper

zone, suggesting a wider variety of viruses were supported in the photic zone. [B] Cluster assignments based on clustering with the IMG/VR viral database (see **Methods: Viral analyses**). [C] Ace Lake metagenomes from which virus contigs were identified. Depths: U1, upper 1; U2, upper 2; U3, upper 3; I, interface; L1, lower 1; L2, lower 2; L3, lower 3. Filter fractions: 3, 20–3 µm; 0.8, 3–0.8 µm; 0.1, 0.8–0.1 µm. The viral genome contigs highlighted in red belong to cluster 24 (see Additional file 4: Table S10).

Table S2 Summary of the virus and NCLDV clusters, singletons and contigs associated with *Phycodnaviridae* 1-5 OTUs.

Virus OTUs	Matches to the Ace Lake virus catalog		Matches to the Ace Lake NCLDV catalog		NCLDV clusters unique to an OTU
	Number of contigs	Number of clusters and singletons to which the contigs belong	Number of contigs	Number of clusters and singletons to which the contigs belong	
Phycodnaviridae 1	377	25	231	15	8
Phycodnaviridae 2	1982	141	889	65	54
Phycodnaviridae 3	510	175	80	17	All
Phycodnaviridae 4	315	46	129	12	5
Phycodnaviridae 5	68	16	30	5	None

NCLDV, nucleocytoplasmic large DNA virus; OTU, operational taxonomic unit.

Table S3 Relative abundance-based correlation between *Micromonas* and *Phycodnaviridae* 1-5 OTUs.

Organism 1	Organism 2	**20-3 µm filter fraction**		**3-0.8 µm filter fraction**		**0.8-0.1 µm filter fraction***	
		Coefficient	P-value	Coefficient	P-value	Coefficient	P-value
Micromonas	*Phycodnaviridae* 1	-0.3	0.2	-0.3	0.3	NC	NC
Micromonas	*Phycodnaviridae* 2	-0.1	0.6	-0.4	0.1	NC	NC
Micromonas	*Phycodnaviridae* 3	0.1	0.9	-0.3	0.3	NC	NC
Micromonas	*Phycodnaviridae* 4	-0.2	0.4	-0.2	0.4	NC	NC
Micromonas	*Phycodnaviridae* 5	-0.2	0.5	-0.4	0.1	NC	NC
Phycodnaviridae 1	*Phycodnaviridae* 2	0.8	0.0004	0.6	0.01	0.5	0.03
Phycodnaviridae 1	*Phycodnaviridae* 3	-0.1	0.8	-0.2	0.5	-0.1	0.7
Phycodnaviridae 1	*Phycodnaviridae* 4	0.9	2E-7	0.7	0.002	0.7	0.001
Phycodnaviridae 1	*Phycodnaviridae* 5	0.7	0.0005	0.7	0.003	0.5	0.2
Phycodnaviridae 2	*Phycodnaviridae* 3	0.4	0.1	0.2	0.6	0.5	0.04
Phycodnaviridae 2	*Phycodnaviridae* 4	0.8	4E-5	0.9	3E-7	0.7	0.001
Phycodnaviridae 2	*Phycodnaviridae* 5	0.9	2E-8	0.9	5E-9	0.96	3E-10
Phycodnaviridae 3	*Phycodnaviridae* 4	0.2	0.5	0.1	0.7	0.2	0.6
Phycodnaviridae 3	*Phycodnaviridae* 5	0.4	0.1	-0.01	1	0.5	0.04
Phycodnaviridae 4	*Phycodnaviridae* 5	0.8	3E-5	0.9	4E-8	0.7	0.002

The Pearson product moment correlation coefficient was used to assess the correlation. The significance of the correlation was established with ANOVA (analysis of variance) regression analysis. * The correlation between *Micromonas* and *Phycodnaviridae* 1-5 was not calculated (NC) in the metagenomes from the 0.8-0.1 µm filter fraction because *Micromonas* was not detected in this size fraction. Correlation coefficients that are significant at 99% confidence level have been highlighted with a grey background.

Table S4 Ace Lake cyanophage metadata.

Contig ID	Taxon ID	Given name	Sample collection date	Filter fraction	Ace Lake zone	Length	GC content	Read depth	Taxonomy*
Cyanophage hits from 120 Ace Lake metagenomes									
Ga0222688_1000631	3300023293	CL6727_D14	Dec-14	0.8-0.1 µm	Upper 1	7780	0.27	9	Unclassified
Ga0222688_1000642	3300023293	CL6580_D14	Dec-14	0.8-0.1 µm	Upper 1	7681	0.27	9	Unclassified
Ga0302066_100481	3300028222	CL9495_D6	Dec-06	0.8-0.1 µm	Upper 2	6313	0.29	4	Unclassified
Ga0302071_100018	3300028228	CL6580_D6	Dec-06	0.8-0.1 µm	Upper 1	51665	0.29	9	Unclassified
Ga0302071_100023	3300028228	CL6727_D6	Dec-06	0.8-0.1 µm	Upper 1	43753	0.28	10	Unclassified
Ga0302071_100029	3300028228	SG14971_D6	Dec-06	0.8-0.1 µm	Upper 1	41278	0.28	14	Unclassified
Ga0302071_100139	3300028228	SG15003_D6	Dec-06	0.8-0.1 µm	Upper 1	19294	0.29	10	Unclassified
Ga0302071_100165	3300028228	CL9892_D6	Dec-06	0.8-0.1 µm	Upper 1	17793	0.26	9	Unclassified
Ga0302071_100180	3300028228	SG14969_D6	Dec-06	0.8-0.1 µm	Upper 1	16925	0.28	9	Unclassified
Ga0302071_100224	3300028228	SG14949_D6	Dec-06	0.8-0.1 µm	Upper 1	14834	0.27	10	*Algoriphagus resistens*
Ga0302071_100266	3300028228	SG14929_D6	Dec-06	0.8-0.1 µm	Upper 1	13303	0.3	11	Enterobacteria phage vB_KleM-RaK2
Cyanophage hits from Ace Lake metaBAT2 co-assembly MAGs									
Ga0374944_296679	3300035698	CL6580_bin2037	NA	NA	NA	15125	0.28	52	Bacteria
Ga0374944_296644	3300035698	SG7011_bin2037	NA	NA	NA	18377	0.27	51	Bacteria
Ga0374944_296647	3300035698	CL9892_bin2037	NA	NA	NA	95297	0.28	49	Bacteria
Ga0374944_296655	3300035698	SG7313_bin2037	NA	NA	NA	25303	0.28	53	Bacteria
Ga0374944_296656	3300035698	SG7152_bin2037	NA	NA	NA	23852	0.29	52	Bacteria

Ga0374944_296660	3300035698	SG8115_bin2037	NA	NA	NA	11785	0.27	48	Bacteria
Ga0374944_296661	3300035698	CL6727_bin2037	NA	NA	NA	22803	0.28	54	Bacteria
Ga0374944_296663	3300035698	SG7974_bin2037	NA	NA	NA	23785	0.29	52	Bacteria
Ga0374944_296671	3300035698	CL9495_bin2037	NA	NA	NA	68414	0.28	51	Bacteria

* See **Methods: Metagenome sequencing, assembly, annotation and overview of analyses** for taxonomic classification. NA, not applicable.

Table S5 *Chlorobium* viruses and their potential hosts.

**Host phylum/class**	**Potential host taxonomy***	**SG14554_D6**	**CL1024_D6A**	**CL1024_A14**
Chlorobi	*Chlorobaculum tepidum*	S51, S52, S53, S54, S55, S56, S57, S58, S60 **(1)**		S2-2 **(1)**
	Chlorobium phaeobacteroides		S3_RC **(1)**, S4_RC **(1)**	S1 **(1)**, S3_RC **(1)**, S4_RC **(1)**
	Chlorobium phaeovibrioides	S20 **(1)**, S21 **(1)**, S22 **(1)**	S3 **(1)**, S4 **(1)**, S3_RC **(1)**, S4_RC **(1)**	S1 **(1)**, S2 **(3)**, S3 **(1)**, S4 **(1)**, S1-1 **(1)**, S1_RC **(1)**, S2_RC, S3_RC **(1)**, S4_RC **(1)**
	Prosthecochloris sp. CIB 2401	S62 **(1)**		
Deltaproteobacteria	Deltaproteobacteria			S12
	Desulfuromonadaceae	S61_RC		
	Desulfuromonadales	S61 **(1)**		
	Desulfuromonas	S61 **(1)**		
Gammaproteobacteria	*Acinetobacter* sp. C15	S19		
	Alcanivorax jadensis	S21 **(1)**, S49_RC, S59, S49-1	S11-1	S11-1
	Edwardsiella tarda	S19_RC		
	Halomonas subterranea	S19		
	Klebsiella pneumoniae	S23, S24, S24-1, S25, S26, S27, S28, S29, S30, S31, S32, S33, S34, S35, S35_RC-1, S36, S37, S38, S39, S40, S41, S42, S43, S44, S45	S8, S11-1, S11, S13, S14, S15	S8, S10, S11-1, S11
	Legionella massiliensis	S48 **(1)**, S48_RC **(1)**		
	Legionella pneumophila	S48-2 **(1)**		
	Marinobacter antarcticus	S49, S49_RC	S17, S17_RC	
	Marinobacter sp.	S21 **(1)**, S21_RC, S41, S42, S63, S64, S65	S7 **(1)**, S7_RC **(1)**, S18	S2 **(3)**, S2_RC, S5, S7 **(1)**, S7_RC **(1)**

	Nitrococcus mobilis		S6 **(1)**	S6 **(1)**
	Vibrio cholerae	S50		
Firmicutes	*Lactobacillus namurensis*	S46, S47	S16	
Flavobacteria	*Runella zeae*	S31		
Verrucomicrobia	*Verrucomicrobium* sp. 3C		S9	S9

For designation purposes, of the 83 spacers with matches to the virus contigs (SG14554_D6, CL1024_D6A, and CL1024_A14), 65 were numbered from S1-S65. The other 18 were either reverse complements of S1-S65 (designated by 'RC' after the spacer number) or their sequences were similar to S1-S65 but were shorter by ≥1 nt (designated by '-1' after the spacer number; e.g., S1 is 34 nt and S1-1 is 33 nt). Sequences that were both shorter and reverse complement were designated by 'RC-1' after the spacer number (see Additional file 4: Table S5 for spacer sequences). Numbers in parentheses are the number of host contigs that also had *cas* genes flanking the CRISPR arrays. The spacers highlighted in red had 90-99% identity matches to the respective virus contigs; all other spacer matches were 100% identical. The grey highlighted microbe is the most abundant OTU, *Chlorobium*, in Ace Lake. * See **Methods: Metagenome sequencing, assembly, annotation and overview of analyses** for taxonomic classification.

Table S6 Spacer sequences with matches to two CL1024 and one SG14554 contigs.

Seq no.	Spacer matches	Spacer length	Designation*
Seq1	ATTTCTTCGGTGGCATATCAGAATTTGAGCTTA	33	S1-1
Seq2	CAAGAGTCAACCCTGATCCAGTACTTCTCAGAA	33	S2_RC
Seq3	CCCCGGCCCCTAGGCCATCCGCGTCGTACCGCA	33	S3
Seq4	TTGGGTGTCATGCTGCCATCGTTGCTCGTGTGG	33	S4
Seq5	TAAGCTCAAATTCTGATATGCCACCGAAGAAATA	34	S1_RC
Seq6	CGCTGGCGACTCAGTTATGATACAGTACGCCCC	33	S5
Seq7	CGTATTCTATAAGACTATCTGTCGGCCATGATT	33	S6
Seq8	CTGAACCCAAACTCACGAATGGCGGCTGCCAC	32	S7
Seq9	GAGATCTTGCTGGTCCCTTTGGCCAAGATACTG	33	S8
Seq10	GCGAAGAAGCTGGGCCTTGAGGAAGTGCCCGTG	33	S9
Seq11	GTCAGTCGAATACAACCTAAGCAGCCCGAACTA	33	S10
Seq12	GTGGCAGCCGCCATTCGTGAGTTTGGGTTCAG	32	S7_RC
Seq13	TATTTCTTCGGTGGCATATCAGAATTTGAGCTTA	34	S1
Seq14	CCACACGAGCAACGATGGCAGCATGACACCCAA	33	S4_RC
Seq15	TGCGGTACGACGCGGATGGCCTAGGGGCCGGGG	33	S3_RC
Seq16	TCAGTGTCGCATATCGAGAATGCCTTTTCCACT	33	S11-1
Seq17	TCAGTGTCGCATATCGAGAATGCCTTTTCCACTG	34	S11
Seq18	TGAGTTTGGGTTCAGGGTGCCTATCCTGGCAAAGTC	36	S12
Seq19	TTCTGAGAAGTACTGGATCAGGGTTGACTCT	31	S2-2
Seq20	TTCTGAGAAGTACTGGATCAGGGTTGACTCTTG	33	S2
Seq21	AGCTGTCGGATAAAGCGTAGTAGTGTCTCATGG	33	S13
Seq22	GCTCTTGACACCGGTCAGCCATCGGTATGGGTG	33	S14
Seq23	CAAAGCTCCTCAAGGCCCGTGCGATCACCCGGC	33	S15
Seq24	TCAGCCGTGAAGAGCTTGAAGCCACCGAGGGTT	33	S16
Seq25	GCGATGCGCTGAGTGTGGAGGGCAGAAGCAAGA	33	S17
Seq26	TCTTGCTTCTGCCCTCCACACTCAGCGCATCGC	33	S17_RC
Seq27	TATTGTTTTTGCGTTGACATGTTTTGTCA	29	S18
Seq28	TTGGTCTGCTTTAGCGTCAAGTAGGTTGTTAACCTCC	37	S19
Seq29	TCTACTTTCGTCTGCGTTGGTATCAGCTCCCA	32	S20
Seq30	ACGCAGTTGAGTATCAAGAAATTACATCCGCGA	33	S21
Seq31	CGTCATCGCACCACCAGCCAATCCGGTATAA	31	S22
Seq32	CACCACCATGCTGGGTGCTATATCGTATCCTTTT	34	S23
Seq33	GGCTTTACGGCCTTTGCGCAGATAAGCGAAAA	32	S24-1
Seq34	GGGGTGGGAGTTATACCGCGCACCCGATTGCCG	33	S25
Seq35	GATACACTAAGGATCGATACAGCAATCACAGAA	33	S26
Seq36	AGACTGGCACCTTTTATCTTGGCTGCTGGCGCAG	34	S27
Seq37	CCTTTTCTTTGACAGCAAGGTAAATGGCACCAG	33	S28
Seq38	TGAAGGGATTGCAGGCATCTGACAAGAGGATAA	33	S29
Seq39	CTCATCGTCTTTGCGGTGCTCGTGTATGACAT	32	S30
Seq40	CTGGGCTAGCGGGGCCCTGACAACTGCGGCGCTC	34	S31
Seq41	GGTACCAGTTACCGCGCCGACCATCCCCTTGCC	33	S32
Seq42	CGACGGATAGACGGATACTATGGACTATCAAGT	33	S33
Seq43	TTGAACAGCCGCTGCAAGTCAGGTTGGGCTTGG	33	S34
Seq44	GTGGCTTCGCTGGCAAACGCTTTTAAGTTGCCG	33	S35
Seq45	GGCTTTACGGCCTTTGCGCAGATAAGCGAAAAA	33	S24
Seq46	CGGAGGTAGTCTTTGGCCACCTCTTCGCGGAAGG	34	S36
Seq47	TTGAGATTCTATCCGCGCACACAAATATGGATG	33	S37
Seq48	TCAAGGCCAACGGTCTTTGCGTCGTTGACTTCC	33	S38
Seq49	TGTTGGGTAGTTTGCTGATCTGCGCACCCTCGAA	34	S39

Seq50	TTATCGGATGTTATACAGCAAGGCAGGAAACAGA	34	S40
Seq51	ATCGTATGCCGCTGGATAGGCTCATCTGTTATG	33	S41
Seq52	AGAGACATTGCCCTCAATGCTTATGGTTTTTGG	33	S42
Seq53	GGCAACTTAAAAGCGTTTGCCAGCGAAGCCAC	32	S35_RC-1
Seq54	GCATCGGCTTTATCCATTGCGCTCGTGAAGT	31	S43
Seq55	TGACCACATCATTCGCGAACCAAAAACCATAAG	33	S44
Seq56	TGACTTCACGAGCGCAATGGATAAAGCTGATGC	33	S45
Seq57	TTAACGGCGGTATCGCTACCCCTGACGTAATAG	33	S46
Seq58	TACGATACCGGCTGGCGGACGAAATCGGGCCCTG	34	S47
Seq59	GGACTACGCTCAATGCTATCGATATCCCGCCA	32	S48_RC
Seq60	TGGCGGGATATCGATAGCATTGAGCGTAGTCC	32	S48
Seq61	GCGGGATATCGATAGCATTGAGCGTAGTCC	30	S48-2
Seq62	TCTTCATTTTGCCTACCGACAAGTCCACTATGA	33	S49_RC
Seq63	TCATAGTGGACTTGTCGGTAGGCAAAATGAAGA	33	S49
Seq64	TCGCGGATGTAATTTCTTGATACTCAACTGCGT	33	S21_RC
Seq65	AAACAGGAATGGAGAACTAACATGGCCCAACAA	33	S50
Seq66	CGCTACAATACCTTCGTGATCGGACTGGTGCC	32	S51
Seq67	CTCACAACCGGTTTTAGCCCGCTCGTAACCTC	32	S52
Seq68	CGCTTCGAGGTTAAAACTGATCGCTCGGTTTTG	33	S53
Seq69	TCCTTCGGCCCACGAGTCGATTATTTCCATGAA	33	S54
Seq70	TCCAAGGGTCGATCATAGTGGACTTGTCGGTAG	33	S55
Seq71	TTAGCATTATTTTTTAACTGTTTTGATTGTTG	32	S56
Seq72	ATAGCACCCAGCATGGTGGTGATGAAGATAAC	32	S57
Seq73	GAGCCGCTGCAAGTCAGGTTGGGCTTGGCTGAGG	34	S58
Seq74	GGAGGTTAACAACCTACTTGACGCTAAAGCAGACCAA	37	S19_RC
Seq75	TCATAGTGGACTTGTCGGTAGGCAAAATGAAG	32	S59
Seq76	GACGGCATAAATCCTCCACGGTTTTATTGTGT	32	S49-1
Seq77	CACAAGCCCGAGAGCCGTAGCAAATCCCGCTGTT	34	S60
Seq78	TAATCGCCAACGGCAATCGGGTGCGCGGTATAA	33	S61
Seq79	TTATACCGCGCACCCGATTGCCGTTGGCGATTA	33	S61_RC
Seq80	CGGCTTTGTCCTGATCCTCTTGGCTCCTATGTC	33	S62
Seq81	TCGACGACATGGTAGGGCCGGGCGAAACGTGGT	33	S63
Seq82	TATACATATTCGCAAATGACCCCAAAGAAGAAC	33	S64
Seq83	CTGTGCTTGAGTTCTGGCAGGGGCATACTGCGT	33	S65

* For designations, see Table S4.

Table S7 Host analysis of virus cluster 1024.

**Phylum/class**	**Contig taxonomy ‡**	**Cluster 1024 virus contig matches to spacers from host contigs**													
		**CL1024_D6A**	**CL1024_D6B**	**CL1024_D6C**	**CL1024_N8A**	**CL1024_N8B**	**CL1024_N8C**	**CL1024_N13**	**CL1024_J14**	**CL1024_A14**	**CL1024_D14A**	**CL1024_D14B**	**CL1024_D14C**	**CL1024_D14D**	**CL1024_D14E**
Chlorobi	*Chlorobium phaeobacteroides*	100	100	100	100	100	100	100	100	100	100	100	100	100	100
	Chlorobium phaeovibrioides	100	100	100	100	100	100	100	100	100	100	100	100	100	100
	Chlorobium phaeovibrioides	100	100	100	100	100	100	100	100	100	100	100	100	100	100
	Chlorobium phaeovibrioides	100	100	100	100	100	100	100	100	100	100	100	100	100	100
	Chlorobium phaeovibrioides			90-99		100					100				
	Chlorobium phaeovibrioides			90-99		100					100				
	Chlorobium phaeovibrioides			90-99		100					100				
	Chlorobium phaeovibrioides			90-99		100					100				
	Chlorobium phaeovibrioides			90-99		100					100				
	Chlorobium phaeovibrioides			90-99		100					100				
	Chlorobium phaeovibrioides			90-99		100					100				
	Chlorobium phaeovibrioides			90-99		100					100				
	Chlorobium phaeovibrioides		100			100	90-99	100	100	100	100	100	100	100	100
	Chlorobium phaeovibrioides			90-99		100		90-99		100	100	100	100	100	
	Chlorobium phaeovibrioides			90-99		100		90-99		100	100	100	100	100	
	Chlorobium phaeovibrioides			90-99		100		90-99		100	100	100	100	100	
	Chlorobium phaeovibrioides					100		90-99		100		100	100	100	
	Chlorobium phaeovibrioides					100		90-99		100		100	100	100	
	Chlorobium phaeovibrioides					100		90-99		100		100	100	100	
	*Chlorobaculum tepidum**					100		90-99		100		100	100	100	
	*Chlorobium**			90-99		100					100				

	Chlorobiaceae*			90-99		100					100				
Deltaproteobacteria	Deltaproteobacteria*	100		100	100	100				100			100		
Gammaproteobacteria	*Alcanivorax jadensis*	100	100	100	100	100	100	100	100	100	100	100	90-99	100	100
	Klebsiella pneumoniae	100	100	100	100	100	100	100	100	100	100	100	90-99	100	100
	Klebsiella pneumoniae	100	100	100	100	100	100	100	100	100	100	100	90-99	100	100
	Klebsiella pneumoniae	100	100	100	100	100	100	100	100	100	100	100	90-99	100	100
	Klebsiella pneumoniae	100	100	100	100	100	100	100	100	100	100	100	90-99	100	100
	Klebsiella pneumoniae	90-99	90-99	90-99	90-99	90-99	90-99	90-99	90-99	90-99	90-99	90-99	90-99	90-99	
	Klebsiella pneumoniae		90-99	90-99	90-99	90-99	90-99	90-99	90-99		90-99	90-99	90-99	90-99	90-99
	Klebsiella pneumoniae		90-99	90-99	90-99	90-99	90-99	90-99	90-99		90-99	90-99	90-99	90-99	90-99
	Klebsiella pneumoniae	90-99				90-99						90-99			
	Klebsiella pneumoniae	100	100												
	Klebsiella pneumoniae		90-99				90-99				90-99				
	Klebsiella pneumoniae		90-99				90-99				90-99				
	*Klebsiella pneumoniae**		100			100	100	100	100	100	100	100	100	100	100
	*Klebsiella pneumoniae**		90-99												
	Marinobacter antarcticus	90-99	100	100	90-99	90-99	100		90-99		90-99	90-99			
	Marinobacter antarcticus	90-99	100	100	90-99	90-99	100		90-99		90-99	90-99			
	Marinobacter antarcticus			90-99		100					100				
	Marinobacter antarcticus			90-99		100					100				
	Marinobacter sp.		90-99	90-99	90-99	90-99	90-99	90-99	90-99		90-99	90-99	90-99	90-99	90-99
	Marinobacter sp.			90-99	90-99	90-99	90-99		90-99		90-99	90-99			
	Marinobacter sp.			90-99	90-99	90-99	90-99		90-99		90-99	90-99			
	Marinobacter sp.			90-99	90-99	90-99	90-99		90-99		90-99	90-99			
	Marinobacter sp.			90-99	90-99	90-99	90-99		90-99		90-99	90-99			
	Marinobacter sp.			90-99		100					100				
	Marinobacter sp.			90-99		100					100				
	Marinobacter sp.			90-99		100		90-99		100	100	100	100	100	
	Marinobacter sp.					100		90-99		100		100	100	100	

	Marinobacter sp.					100		90-99		100		100	100	100	
	Marinobacter sp.					100		90-99		100		100	100	100	
	Marinobacter sp.					100		90-99		100		100	100	100	
	Marinobacter sp.					100		90-99		100		100	100	100	
	Marinobacter sp.					100		90-99		100		100	100	100	
	Marinobacter sp.					100		90-99		100		100	100	100	
	Marinobacter sp.*	90-99		90-99	90-99	90-99				90-99			90-99		
	Marinobacter sp.*	90-99		90-99	90-99	90-99				90-99			90-99		
	Marinobacter sp.*		90-99		90-99	90-99	90-99	90-99	90-99	90-99	90-99	90-99	90-99	90-99	90-99
	Marinobacter sp.*			90-99	90-99	90-99	90-99		90-99		90-99	90-99			
	Vibrio cholerae			90-99		100					100				
	*Marinospirillum celere**		90-99	90-99	90-99	90-99	90-99	90-99	90-99		90-99	90-99	90-99	90-99	90-99
	*Nitrococcus mobilis**		90-99				90-99				90-99				
	Gammaproteobacteria*		90-99	90-99	90-99	90-99	90-99	90-99	90-99		90-99	90-99	90-99	90-99	90-99
Betaproteobacteria	*Delftia lacustris**		90-99	90-99	90-99	90-99	90-99	90-99	90-99		90-99	90-99	90-99	90-99	90-99
Verrucomicrobia	*Verrucomicrobium* sp. 3C	100	100	100	100	100	100	100	100	100	100	100	100	100	100
	Bacteria*		90-99	90-99	90-99	90-99	90-99	90-99	90-99		90-99	90-99	90-99	90-99	90-99
	Bacteria*			90-99		100					100				

The values are % identity matches of cluster 1024 virus contigs to host contig spacers. The dark grey-shaded cells indicate 100% identity matches, whereas the light grey-shaded cells indicate 90-99% identity matches. The unshaded cells with no values indicate that the virus contig in the corresponding column had no hits to spacers from the host contig in the corresponding row. ‡ Each row represents a contig with taxonomy assigned as described in **Methods: Metagenome sequencing, assembly, annotation and overview of analyses**. *For specific host contigs the IMG taxonomic assignment was used. For full details of CL1024 contigs see Additional file 4: Table S9.

Table S8 Most abundant virus clusters in Ace Lake.

**Virus cluster**	**Ace Lake zone in which abundant**	**Read depth of a virus cluster compared to the *Chlorobium* peak read depth ‡**	**Probable host †**
20	Upper	2.4	Eukarya
463	Upper	1.6	Bacteria
5	Upper	1	Bacteria
159	Upper	1	Bacteria
9	Upper	0.9	Eukarya
7	Upper	0.8	Eukarya
35	Upper	0.8	Eukarya
32	Upper	0.7	Eukarya
54	Upper	0.7	Unknown
89	Upper	0.7	Unknown; Ace lake-specific
191	Upper	0.7	Unknown; Ace lake-specific
400	Interface and Lower	0.7	Unknown; Ace lake-specific
11	Upper	0.6	Bacteria
37	Upper	0.6	Unknown; Ace lake-specific
66	Upper	0.6	Eukarya
248	Interface and Lower	0.6	Unknown; Ace lake-specific
295	Upper	0.6	Bacteria

‡ The read depth of virus contigs corresponding to a cluster are the sum of the read depths for a sampling time period, incorporating all lake depths and all filter fractions. This value was divided by the peak read depth for *Chlorobium*. Clusters with total read depth >4000 (~0.6 of the *Chlorobium* peak read depth) in at least one time period are shown. No virus singletons had read depth >4000. † The probable hosts of the most abundant virus clusters were identified based on the host assignments of the virus clusters in the IMG/VR viral database, i.e. contigs belonging to the same cluster and without a host assignment were associated to the host predicted for other member(s) of the cluster (if any).

Table S9 Metadata associated with *Chlorobium* viruses.

Contig ID	Taxon ID	Given name	Sample collection date	Filter fraction ‡	Ace Lake zone	Length	GC content	Read depth	Taxonomy*
Ga0302060_10025	3300028201	CL1024_D6A	Dec 2006	0.8	Interface	11188	0.48	34	Unassigned
Ga0302061_10032	3300028203	CL1024_D6B	Dec 2006	3	Interface	8085	0.49	13	Vibrio phage VBM1
Ga0302067_10039	3300028204	CL1024_D6C	Dec 2006	0.1	Interface	6665	0.45	24	Unassigned
Ga0208900_1004295	3300025433	CL1024_N8A	Nov 2008	3	Interface	5426	0.48	613	Unassigned
Ga0208904_1006197	3300025669	CL1024_N8B	Nov 2008	0.1	Lower 2	6239	0.48	37	Unassigned
Ga0208905_1004525	3300025661	CL1024_N8C	Nov 2008	0.8	Lower 3	6310	0.48	46	Unassigned
Ga0222638_1002074	3300023298	CL1024_N13	Nov 2013	3	Lower 2	5603	0.48	28	Unassigned
Ga0222656_1001806	3300022834	CL1024_J14	Jul 2014	3	Interface	5624	0.48	29	Unassigned
Ga0222665_1003383	3300022864	CL1024_A14	Aug 2014	3	Interface	5317	0.47	24	Unassigned
Ga0222689_1000957	3300023231	CL1024_D14A	Dec 2014	3	Upper 3	6117	0.48	27	Unassigned
Ga0222690_1000793	3300023227	CL1024_D14B	Dec 2014	0.8	Upper 3	6492	0.47	50	Unassigned
Ga0222695_1002624	3300023253	CL1024_D14C	Dec 2014	3	Lower 1	5952	0.48	30	Unassigned
Ga0222696_1002166	3300023233	CL1024_D14D	Dec 2014	0.8	Lower 1	5599	0.48	37	Unassigned
Ga0222699_1002408	3300022846	CL1024_D14E	Dec 2014	0.8	Lower 1	5369	0.47	40	Unassigned
Ga0302067_10019	3300028204	SG14554_D6	Dec 2006	0.1	Interface	14104	0.51	146	Vibrio phage VBM1
Ga0302060_10018	3300028201	CL248	Dec 2006	0.8	Interface	17393	0.5	79	*Sphingomonas histidinilytica*
Ga0302061_10026	3300028203	CL248	Dec 2006	3	Interface	10117	0.52	28	*Sphingomonas histidinilytica*
Ga0302067_10021	3300028204	CL248	Dec 2006	0.1	Interface	12817	0.51	66	*Sphingomonas histidinilytica*
Ga0302056_100137	3300028227	CL248	Dec 2006	0.8	Lower 2	7336	0.51	6	*Sphingomonas histidinilytica*
Ga0302055_100061	3300028226	CL248	Dec 2006	0.8	Lower 3	8566	0.51	10	*Sphingomonas histidinilytica*
Ga0302070_100065	3300028296	CL248	Dec 2006	0.1	Lower 3	7664	0.49	6	*Sphingomonas histidinilytica*
Ga0302070_100157	3300028296	CL248	Dec 2006	0.1	Lower 3	5159	0.51	7	*Sphingomonas histidinilytica*

Ga0208279_1004110	3300025649	CL248	Nov 2008	3	Lower 1	6103	0.51	515	*Sphingomonas histidinilytica*
Ga0208902_1002585	3300025628	CL248	Nov 2008	0.8	Lower 1	8161	0.51	136	*Sphingomonas histidinilytica*
Ga0208769_1002379	3300025697	CL248	Nov 2008	0.1	Lower 1	7992	0.51	143	*Sphingomonas histidinilytica*
Ga0207996_1003791	3300025586	CL248	Nov 2008	0.8	Lower 2	7966	0.51	189	*Sphingomonas histidinilytica*
Ga0208904_1004243	3300025669	CL248	Nov 2008	0.1	Lower 2	8059	0.51	284	*Sphingomonas histidinilytica*
Ga0222626_1002761	3300022882	CL248	Nov 2013	3	Interface	6100	0.51	383	*Sphingomonas histidinilytica*
Ga0222627_1001894	3300023244	CL248	Nov 2013	0.8	Interface	6106	0.51	365	*Sphingomonas histidinilytica*
Ga0222628_1002177	3300022871	CL248	Nov 2013	0.1	Interface	6106	0.51	796	*Sphingomonas histidinilytica*
Ga0222635_1001297	3300023234	CL248	Nov 2013	3	Lower 1	7103	0.51	131	*Sphingomonas histidinilytica*
Ga0222636_1002821	3300022854	CL248	Nov 2013	0.8	Lower 1	5518	0.51	104	*Sphingomonas histidinilytica*
Ga0222637_1002828	3300023435	CL248	Nov 2013	0.1	Lower 1	5215	0.51	73	*Sphingomonas histidinilytica*
Ga0222639_1002111	3300023262	CL248	Nov 2013	0.8	Lower 2	8030	0.51	157	*Sphingomonas histidinilytica*
Ga0222640_1001468	3300023297	CL248	Nov 2013	0.1	Lower 2	6934	0.51	138	*Sphingomonas histidinilytica*
Ga0222641_1000777	3300022828	CL248	Nov 2013	3	Lower 3	5218	0.52	91	*Sphingomonas histidinilytica*
Ga0222679_1001284	3300022858	CL248	Oct 2014	0.1	Lower 1	7692	0.51	68	*Sphingomonas histidinilytica*
Ga0222682_1001695	3300023246	CL248	Oct 2014	0.1	Lower 2	5038	0.52	108	*Sphingomonas histidinilytica*

Ga0222686_1001026	3300023501	CL248	Dec 2014	3	Upper 2	6903	0.51	150	*Sphingomonas histidinilytica*
Ga0222689_1000893	3300023231	CL248	Dec 2014	3	Upper 3	6437	0.51	926	*Sphingomonas histidinilytica*
Ga0222689_1001025	3300023231	CL248	Dec 2014	3	Upper 3	5833	0.5	954	Unassigned
Ga0222691_1002182	3300022851	CL248	Dec 2014	0.1	Upper 3	5045	0.52	38	*Sphingomonas histidinilytica*
Ga0222695_1002611	3300023253	CL248	Dec 2014	3	Lower 1	5972	0.5	264	Unassigned
Ga0222696_1001254	3300023233	CL248	Dec 2014	0.8	Lower 1	8225	0.5	132	*Sphingomonas histidinilytica*
Ga0222698_1001226	3300022860	CL248	Dec 2014	3	Lower 2	7682	0.5	354	Unassigned
Ga0222698_1002050	3300022860	CL248	Dec 2014	3	Lower 2	5591	0.51	399	*Sphingomonas histidinilytica*
Ga0222699_1001059	3300022846	CL248	Dec 2014	0.8	Lower 2	8592	0.51	279	*Sphingomonas histidinilytica*
Ga0222700_1001281	3300023061	CL248	Dec 2014	0.1	Lower 2	5063	0.51	82	*Sphingomonas histidinilytica*
Ga0222701_1001644	3300022884	CL248	Dec 2014	3	Lower 3	6103	0.51	265	*Sphingomonas histidinilytica*
Ga0222702_1001705	3300023299	CL248	Dec 2014	0.8	Lower 3	6313	0.51	233	*Sphingomonas histidinilytica*
Ga0208414_1003043	3300025603	CL400	Nov 2008	0.1	Upper 2	10451	0.34	20	Unassigned
Ga0208900_1002555	3300025433	CL400	Nov 2008	3	Interface	8218	0.33	308	Unassigned
Ga0208901_1000990	3300025380	CL400	Nov 2008	0.1	Interface	8545	0.33	452	Unassigned
Ga0208647_1000742	3300025362	CL400	Nov 2008	0.1	Interface	7900	0.33	954	Unassigned
Ga0208279_1002046	3300025649	CL400	Nov 2008	3	Lower 1	10106	0.33	128	Unassigned
Ga0208902_1002371	3300025628	CL400	Nov 2008	0.1	Lower 1	8544	0.33	256	Unassigned
Ga0208769_1002438	3300025697	CL400	Nov 2008	0.1	Lower 1	7877	0.33	341	Unassigned
Ga0207996_1003599	3300025586	CL400	Nov 2008	0.1	Lower 2	8246	0.33	539	Unassigned
Ga0208904_1004387	3300025669	CL400	Nov 2008	0.1	Lower 2	7890	0.33	1030	Unassigned
Ga0208771_1002615	3300025698	CL400	Nov 2008	3	Lower 3	8545	0.33	310	Unassigned
Ga0208905_1003078	3300025661	CL400	Nov 2008	0.1	Lower 3	7889	0.33	336	Unassigned
Ga0207997_1003371	3300025736	CL400	Nov 2008	0.1	Lower 3	7890	0.33	399	Unassigned

Ga0222635_1001119	3300023234	CL400	Nov 2013	3	Lower 1	7890	0.33	112	Unassigned
Ga0222638_1001230	3300023298	CL400	Nov 2013	3	Lower 2	7890	0.33	82	Unassigned
Ga0222656_1001064	3300022834	CL400	Jul 2014	3	Interface	7890	0.33	82	Unassigned
Ga0222658_1003935	3300023257	CL400	Jul 2014	0.1	Interface	5310	0.34	481	Unassigned
Ga0222666_1002834	3300024048	CL400	Aug 2014	0.1	Interface	5311	0.34	279	Unassigned
Ga0222673_1001116	3300022821	CL400	Oct 2014	0.1	Interface	9687	0.33	30	Unassigned
Ga0222677_1001056	3300022855	CL400	Oct 2014	3	Lower 1	7889	0.33	117	Unassigned
Ga0222681_1001368	3300022838	CL400	Oct 2014	0.1	Lower 2	7890	0.33	59	Unassigned
Ga0222682_1001525	3300023246	CL400	Oct 2014	0.1	Lower 2	5311	0.34	146	Unassigned
Ga0222685_1000765	3300022874	CL400	Oct 2014	0.1	Lower 3	7889	0.33	167	Unassigned
Ga0222689_1000515	3300023231	CL400	Dec 2014	3	Upper 2	10005	0.33	31	Unassigned
Ga0222691_1000883	3300022851	CL400	Dec 2014	0.1	Upper 2	9331	0.33	23	Unassigned
Ga0222700_1000514	3300023061	CL400	Dec 2014	0.1	Lower 2	7890	0.33	148	Unassigned
Ga0222701_1002009	3300022884	CL400	Dec 2014	3	Lower 3	5311	0.34	132	Unassigned

‡ Filter fractions: 3, 20–3 µm; 0.8, 3–0.8 µm; 0.1, 0.8–0.1 µm * see **Methods: Metagenome sequencing, assembly, annotation and overview of analyses** for taxonomic classification.

Table S10 Metadata for the cluster 24 'huge' phage.

**Metagenome**[A] (sample collection date, depth, filter fraction)	**Taxon ID**	**Contig ID**	**Contig length (bp)**	**GC content**	**Relative abundance (%)**[B]	**Cas genes identified (number of spacer sequences)**[C]
Nov 2008_L1_0.1 µm	3300025697	Ga0208769_1000001	528,260	0.56	0.9	Yes (14)
Nov 2008_L2_0.1 µm	3300025669	Ga0208904_1000003	447,854	0.56	1	Yes (16)
Nov 2008_L3_3 µm	3300025698	Ga0208771_1000001	528,138	0.56	0.2	Yes (14)
Nov 2013_L1_0.1 µm	3300023435	Ga0222637_1000003	323,923	0.56	1	
		Ga0222637_1000005	204,206	0.56	1	Yes (16)
Nov 2013_L2_0.1 µm	3300023297	Ga0222640_1000001	528,282	0.56	1	Yes (16)
Oct 2014_L1_0.1 µm	3300022858	Ga0222679_1000001	528,258	0.56	2	Yes (16)
Oct 2014_L2_0.1 µm	3300023246	Ga0222682_1000001	528,256	0.56	1	Yes (14)
Dec 2014_L2_0.1 µm	3300023061	Ga0222700_1000001	521,772	0.56	1	Yes (16)
Metabat2 co-assembly MAGs (bin1297)*	3300035698	Ga0374944_093690	42,584	0.57	NA	
		Ga0374944_093691	204,460	0.56	NA	Yes (14)
		Ga0374944_093692	216,989	0.57	NA	

[A] Metagenome data containing complete (or nearly complete) cluster 24 'huge' phage. Depths: L1, lower 1; L2, lower 2; L3, lower 3. Filter fractions: 3, 20–3 µm; 0.1, 0.8–0.1 µm. [B] Relative abundances of individual virus contigs calculated as described in **Methods: Calculations of taxonomic abundance**. [C] Cas gene annotations from IMG were manually verified. * MetaBAT2 co-assembly MAGs generated as described in **Methods: Metagenome sequencing, assembly, annotation and overview of analyses**. The three contigs (from a total of five in bin1297) that were assigned to cluster 24 are listed. The red-highlighted entries are the complete, circular phage shown in Additional file 4: Table S1.
